# Supplementary material for: Total syntheses of Tetrodotoxin and 9-epiTetrodotoxin
Source: Nat Commun. 2024 Jan 23;15:679. doi: 10.1038/s41467-024-45037-0 (PMC10806222; doi:10.1038/s41467-024-45037-0)
Supplement: Supplementary file 1 — Supplementary Information [file 41467_2024_45037_MOESM1_ESM.pdf]

Supplementary information

Total Syntheses of Tetrodotoxin and 9-*epi*Tetrodotoxin

Authors: Peihao Chen†, Jing Wang†, Shuangfeng Zhang, Yan Wang, Yuze Sun, Songlin Bai, Qingcui Wu, Xinyu Cheng, Peng Cao, Xiangbing Qi*

Contents

Supplementary information *1*

1. Supplementary Notes **2**

2. Supplementary Discussion **3**

3. Supplementary Methods **12**

1. Detailed experimental procedures *12*

2. Biological Evaluation *33*

3. NMR spectra and HPLC spectra *35*

4. Computational details *69*

5. Crystallographic information *71*

4. Supplementary References **77**

1. Supplementary Notes

All reactions were carried out under an atmosphere of argon in flame-dried glassware with magnetic stirring unless otherwise indicated. Reagents were purchased from Aldrich Chemical, Alfa Aesar, TCI, Adamas, or J&K at the highest commercial quality and used without further purification. TTX sample was purchased from Tocris Bioscience. Solvents were dried by passage through an activated alumina column under argon. Liquids and solutions were transferred via syringe. Reactions were monitored by GC/MS, UPLC/MS, and thin layer chromatography (TLC) and visualization was accomplished with a 254 nm UV light and by staining with phosphomolybdic acid solution with heating. All Flash silica gel column chromatography was performed using silica gel with particle size of 300-400 mesh. ^{1}H and ^{13}C NMR spectra were recorded on Varian Inova-400 spectrometers. Data for ^{1}H NMR spectra are reported relative to $CDCl_3$ (7.26 ppm), CD_3OD (3.31 ppm), or CD_3CO_2D (2.06 ppm) as an internal standard and are reported as follows: chemical shift (δ ppm), multiplicity (*s* = singlet, *d* = doublet, *t* = triplet, *q* = quartet, *sept* = septet, *m* = multiplet, *br* = broad), coupling constant *J* (Hz), and integration. Data for ^{13}C NMR spectra are reported relative to $CDCl_3$ (77.00 ppm), CD_3OD (49.00 ppm), CD_3CO_2D (22.4 ppm) as an internal standard and are reported in terms of chemical shift (δ ppm). UPLC-MS analyses were performed on a Waters system (Column: BEH C18, 1.7 µm, 2.1*50 mm) with a Photodiode Array (PDA) detector and a Single Quadrupole (SQ) detector. High Resolution Mass spectra were obtained from on an Agilent 1290 LC-6540 QTOF Mass Spectrometer or an Agilent Technologies 7250 GCQTOF. HPLC analyses were carried out on Waters (Column Atlanits® HILIC Silica, 5 µm, 4.6*150 mm and 19*150 mm) with 2998PDA and 3100MS detectors.

2. Supplementary Discussion

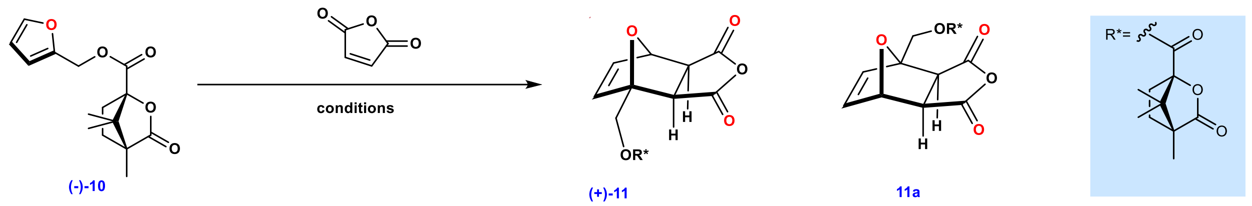

Entry	Maleic anhydride (eq.)	Temperature(°C)	Solvent	Time(h)	(+)-11 [a] [%]	11:11a
1	1.0	55	Neat	24	36.5	5:4
2	1.0	55	Toluene	24	16.8	1:1
3	1.0	55	MTBE	24	38.9	10:1
4	1.0	55	Isopropyl Ether	24	65.5	10:1
5	1.0	55	THF	24	13.8	1:1
6	1.0	55	CHCl3	24	11.4	1:1
7	1.0	55	CH3CN	24	17.1	5:6
8	1.0x2[b]	55	Neat	48	68.5	5:1
9	2.0x2[b]	55	Neat	48	71.1	10:3
10	4.0x2[b]	55	Neat	48	39.7	5:6
11	1.0x2[b]	55	Isopropyl Ether	48	86.8	>20:1
12	1.0x2[b]	55	Isopropyl Ether	48	91.3[c]	>20:1

Supplementary Table 1. Optimization of the stereoselective Diels-Alder reaction. **a,** All optimization reactions were carried out at a 1.0 g scale. The yields were determined by ^{1}H NMR with 1,3,5-trimethoxybenzene as the internal standard. **b,** After 12h, the same eq. maleic anhydride was added. **c,** Scale up to 100 grams, isolated yield with 6% (molar ratio) maleic anhydride. *MTBE:methyl tert-butyl ether.

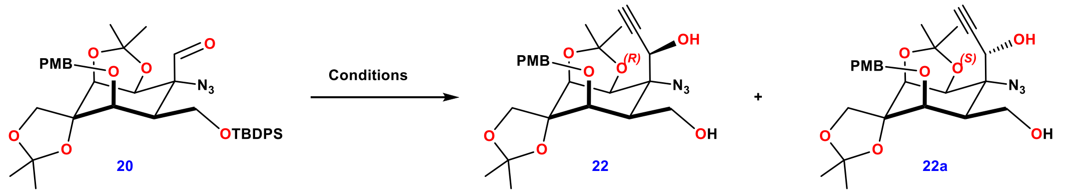

Entry	[b]Conditions	Solvent, temperature (°C)	Time (h)	22a:22[a]	Combined yields[a]
1	TMS—≡—Li	THF, -78 °C	2	>20:1	80%
2	TMS—≡—Li/ HMPA	THF, -78 °C	2	-	<10%
3	TMS—≡—Li/ $ZnBr_2$	THF, 0 °C to r.t.	16	-	<10%
4	TMS—≡—Li	Et_2O, -78 °C	2	>20:1	53%
5	TMS—≡—Li/ $ZnBr_2$	Et_2O, 0 °C to r.t.	16	-	<10%
6	TMS—≡—MgBr	THF, -78 °C to r.t	2	N.D.	N.D.
7	CaC_2	THF, -78 °C	2	-	N.R.
8	≡—Li $NH_2CH_2CH_2NH_2$	THF, -78 °C	2	15:1	70%(66%)
9	≡—MgBr	THF, -78 °C to r.t	2	N.D.	N.D.

Supplementary Table 2. Optimization of 1,2-addition of acetylide anion to **20**. **a,** Combined yields (**22a**+**22**) were determined by ^{1}H NMR with 1,3,5-trimethoxybenzene as the internal standard, the yield in bracket is isolated yield. **b,** Entry 1 to 5 using metallated ethynyltrimethylsilane as nucleophiles were treated with K_2CO_3/ MeOH to deprotect TBDPS and TMS. *N.D., not determined. N.R., no reaction.

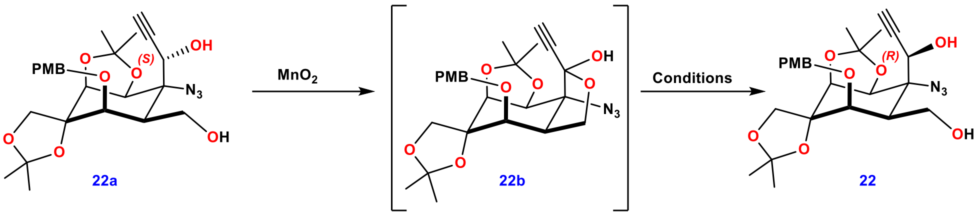

Entry	Conditions	Solvent, temperature (ºC)	Time (h)	22a:22[a]	Combined yields[a]
1	$LiBH_4$	THF, r.t.	0.5h	1:1	87%
2	L-selectride	CH_2Cl_2, -78 ºC to r.t.	16h	-	N.D.
3	L-selectride/$ZnCl_2$	CH_2Cl_2, -78 ºC to r.t.	3h	-	N.D.
4	$LiAlH_4$	THF, 0 ºC to r.t.	1h	-	N.D.
5	DIBAL-H	THF, -78 ºC to r.t.	3h	1:2	25%
6	CBS cat./ BH_3•THF	THF, -15 ºC	16h	-	N.D.
7	$NaBH_4$	Dioxane:H_2O(10:1), r.t.	1.5h	1:2	76%
8	$NaBH_4$	Dioxane:H_2O(10:1), 60ºC	0.5h	1:2	85% (77%[b])

Supplementary Table 3. Optimization of the reduction conditions to epimerize 22a to 22. **a**, Combined yields (22a+22) were determined by ^{1}H NMR with 1,3,5-trimethoxybenzene as the internal standard. **b**, Isolated yields. *N.D., not determined.

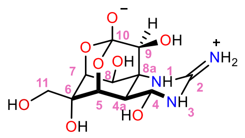

Tetrodotoxin (1)

	Our Synthetic TTX[a]		Natural TTX[b]	
position	δ(C)	δ(H)	δ(C)	δ(H)
2	156.5	—	156.6	—
4	75.1	5.49 (d, J = 9.4Hz)	75.1	5.50 (d, J = 9.4Hz)
4a	40.6	2.34 (d, J = 9.4 Hz)	40.7	2.35 (d, J = 9.4Hz)
5	73.8	4.24 (br s)	73.8	4.25 (br s)
6	71.4	—	71.5	—
7	79.6	4.07 (br s)	79.7	4.08 (t, J = 1.8Hz)
8	72.7	4.28 (br s)	72.8	4.30 (d, J = 1.5Hz)
8a	59.7	—	59.7	—
9	70.8	3.95 (s)	70.9	3.96 (s)
10	110.8	—	110.8	—
11	65.5	4.01(d, J = 12.6Hz)<br>4.04(d, J = 12.6Hz)	65.5	4.02 (d, J = 12.6Hz)<br>4.04 (d, J = 12.6Hz)

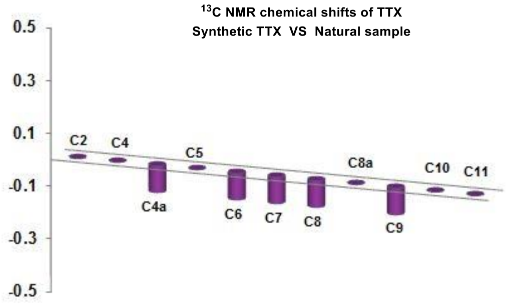

Supplementary Table 4. Comparison of the ^{1}H and ^{13}C NMR data for Natural [1] and our Synthetic Tetrodotoxin. **a**: ^{1}H NMR (600 MHz) and ^{13}C NMR (151 MHz) in 5% CD_3CO_2D/D_2O. Samples were referenced to CHD_2CO_2D = 2.06 ppm, $^{13}CHD_2CO_2D$ = 22.4 ppm. **b:** NMR data for Natural TTX.

Reference: Yasumoto, M. Yotsu, M. Murata, H. Naoki, *J. Am. Chem. Soc.* **1988**, 110, 2344-2345.

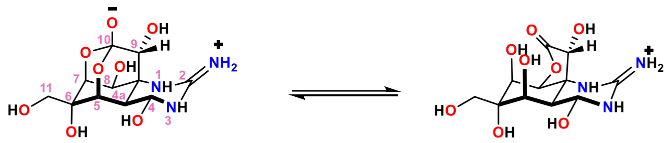

9-*epi*Tetrodotoxin-hemilactal (1a) 9-*epi*Tetrodotoxin-10,8-lactone (1b)

position	δ(H) Our Synthetic 9-*epi*TTX[a] hemilactal	10,8-lactone	Natural 9-*epi*TTX[b] hemilactal	10,8-lactone
2	—	—	—	—
4	5.24 (d,*J* = 9.2 Hz)	5.34 (d,*J* = 10.4Hz)	5.26 (d,*J* = 9.4 Hz)	5.37 (d,*J* = 10.4 Hz)
4a	2.45 (d,*J* = 9.2 Hz)	2.53 (dd,*J* = 10.2 Hz, 2.2 Hz)	2.48 (d,*J* = 9.4 Hz)	2.55 (dd,*J* = 10.4 Hz, 2.8 Hz)
5	4.15 (brs)	4.08 (br s)	4.18 (br s)	4.11 (dd,*J* = 2.8 Hz, 0.9 Hz)
6	—	—	—	—
7	4.24 (br s)	4.18 (d,*J* = 5.2 Hz)	4.26 (br s)	4.21 (dd,*J* = 5.0 Hz, 0.9 Hz)
8	4.22 (br s)	—[c]	4.25 (br s)	4.90
8a	—	—	—	—
9	3.85 (s)	5.44 (s)	3.87 (s)	5.46 (s)
10	—	—	—	—
11	3.99 (d, *J* = 11.6 Hz)<br>4.05 (d, *J* = 11.6 Hz)	3.85 (d, *J* = 12.0Hz)<br>3.98 (d, *J* = 12.0Hz)	4.02 (d, *J* = 11.4 Hz)<br>4.08 (d, *J* = 11.4 Hz)	3.88 (d, *J* = 11.6 Hz)<br>4.00 (d, *J* = 11.6 Hz)

Supplementary Table 5. Comparison of the ^1H NMR data for Natural [2] and Synthetic 9-*epi*Tetrodotoxin. **a**: ^1H NMR (400 MHz) in 5% CD_3CO_2D/D_2O. Samples were referenced to CHD_2CO_2D = 2.06 ppm. **b**: NMR data for Natural 9-*epi*TTX. Reference: *J. Nat. Prod*. **2022**, 85, 9, 2199–2206. **c**: Because of overlap with the DHO signal.

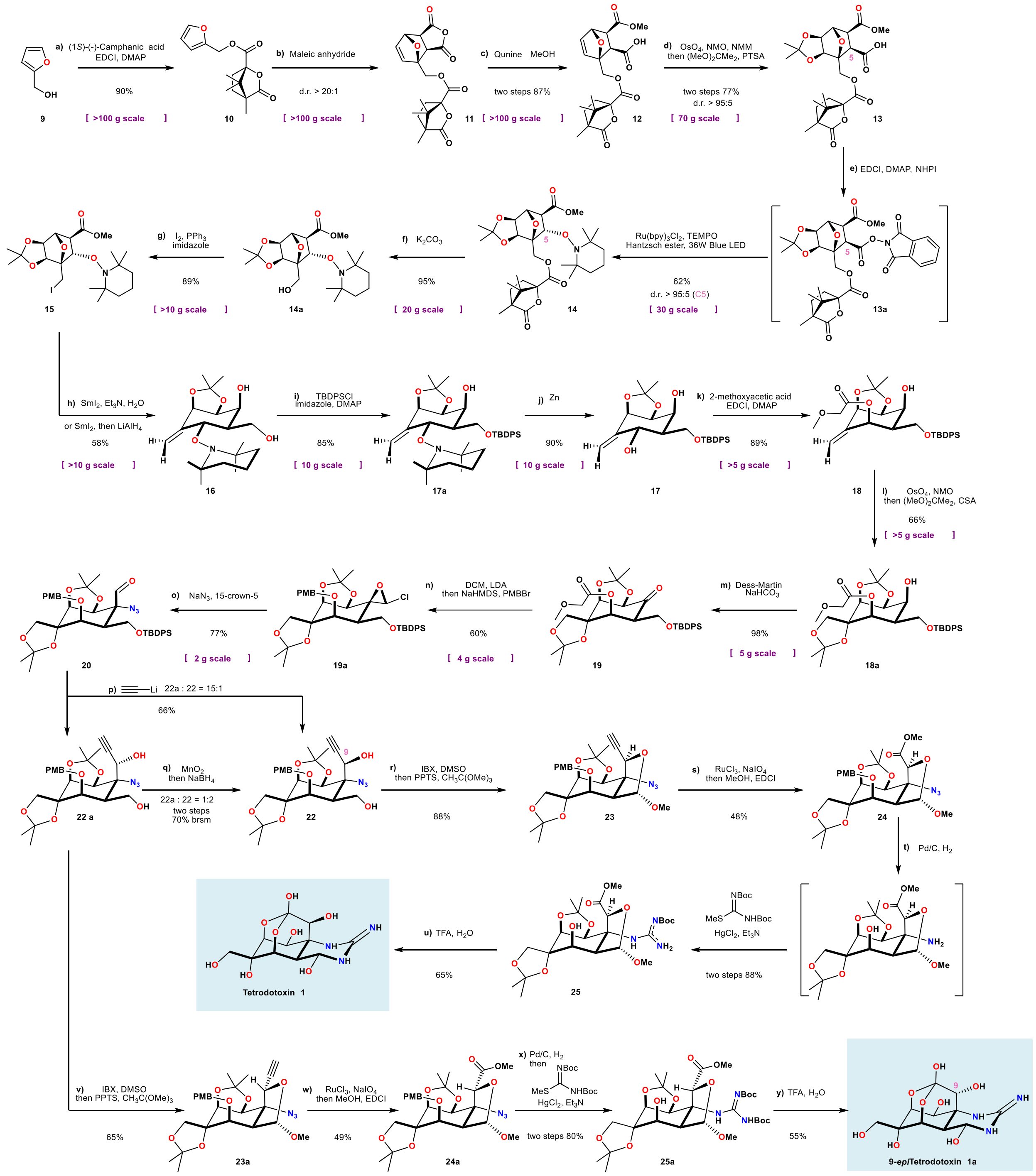

Supplementary Figure 1. Total syntheses of Tetrodotoxin and 9-*epi*Tetrodotoxin. The complete and detailed steps of synthesis.

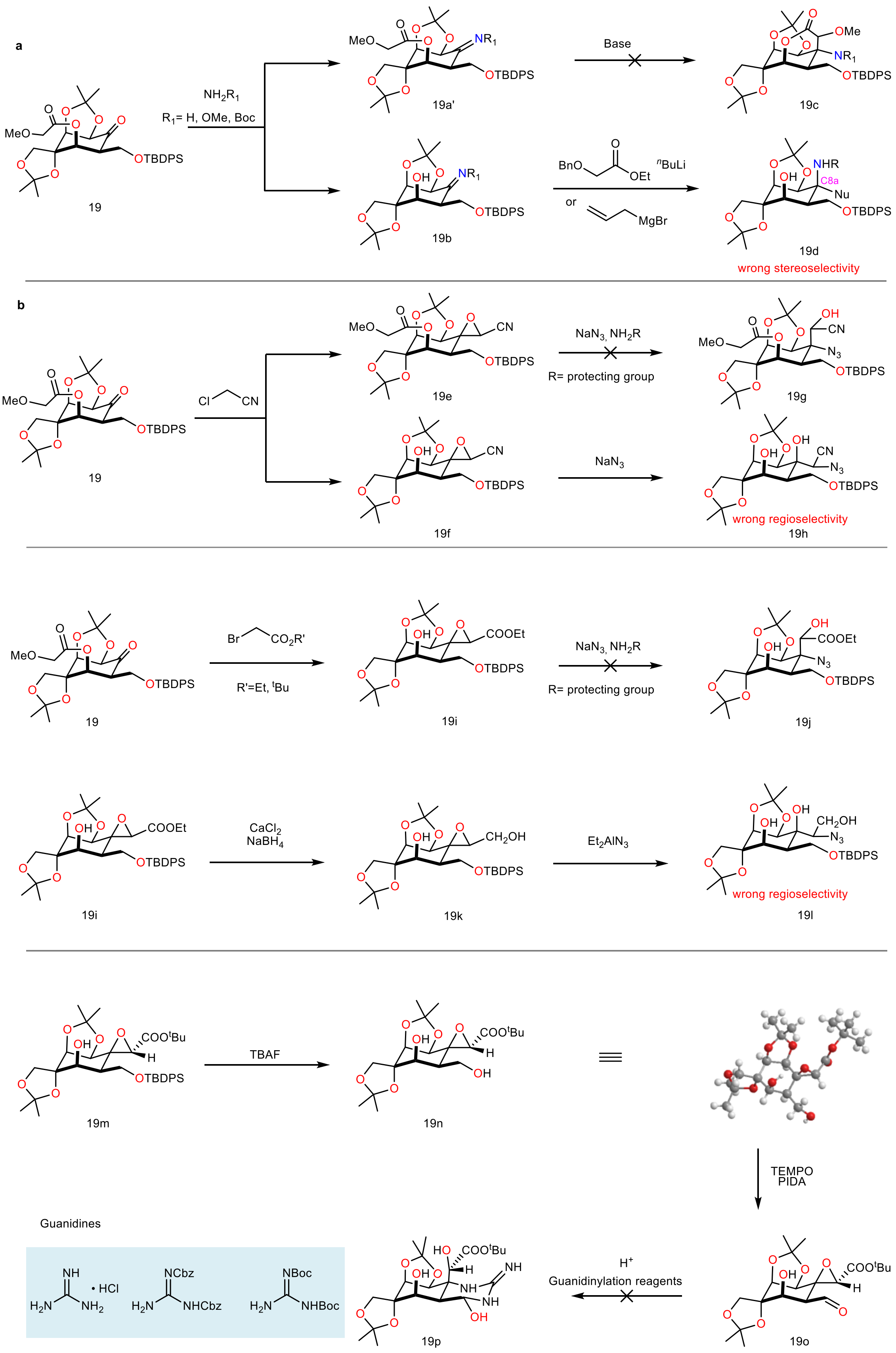

Supplementary Figure 2. Failed attemption in constructing stereocenter at C8a. **a**, Nucleophilic addition to imine produced a diastereomer with undesired configuration at C8a. **b**, Stereoselective aminolysis glycidic ester constructed by Darzens condensation of **19** with *α*-haloester.

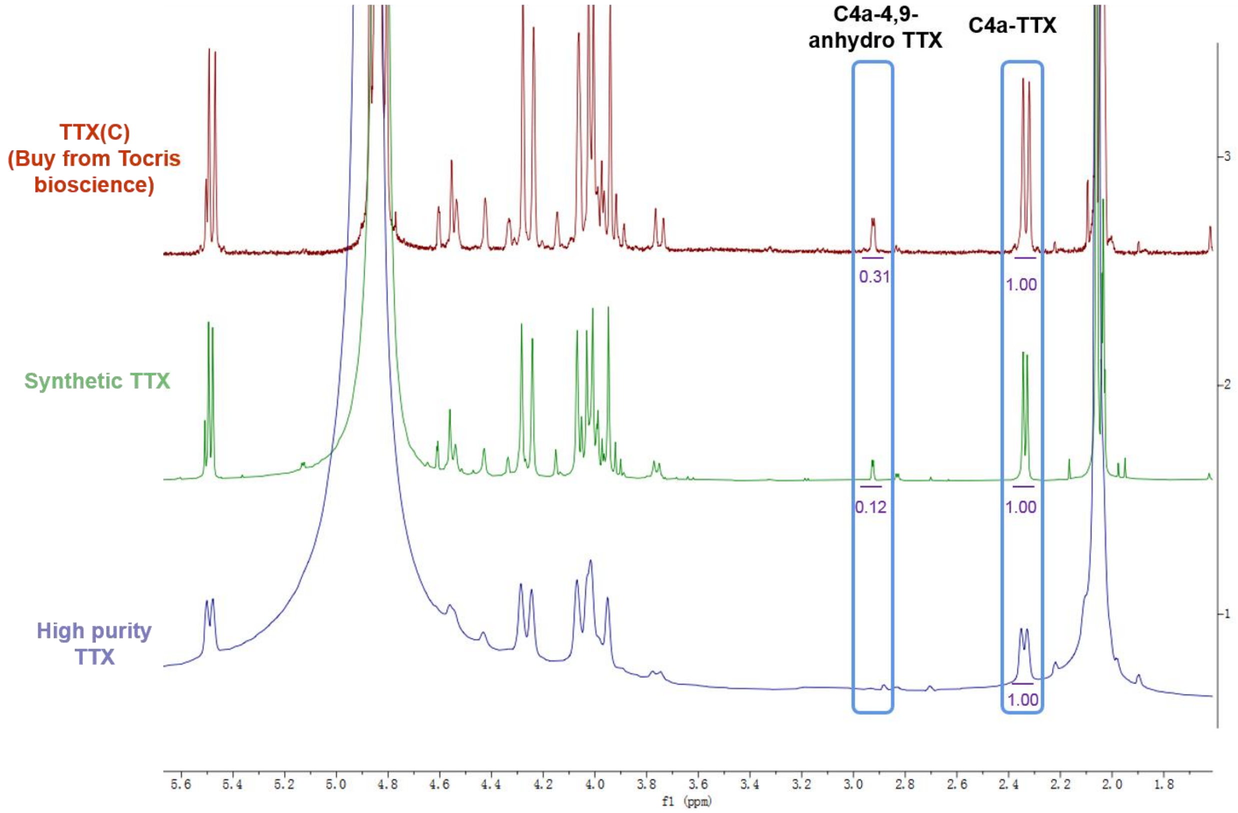

Supplementary Figure 3. Comparison of ^{1}H NMR spectra among the TTX(C) (Buy from Tocris bioscience, TTX:anhydro TTX=10:3), Synthetic TTX (Method A, TTX:anhydro TTX=10:1) and High purity TTX (Method B, TTX:anhydro TTX>20:1).

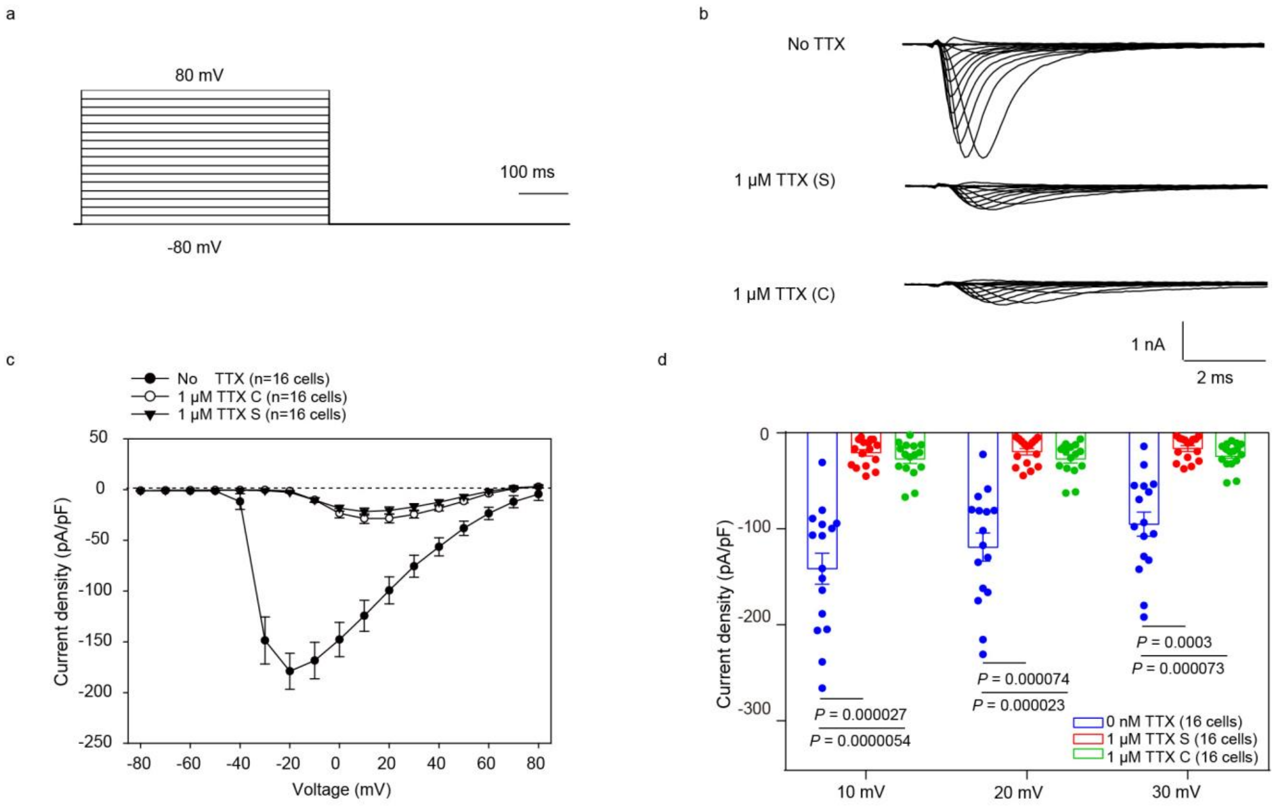

Supplementary Figure 4. The blocking potency between TTX(**S**) and TTX(**C**) on HEK-Na$_v$1.5 cells. **a**, A schematic image represents an evoked step-wise protocol by voltage clamp on HEK-Na$_v$1.5 cells from −80 mV to 80 mV in 10-mV increments. **b**, Representative traces of HEK-Na$_v$1.5 currents under no-TTX condition or 1 μM TTX(**S**) condition or 1 μM TTX(**C**) condition. **c**, The plot graph displays the HEK-Na$_v$1.5 current densities evoked by series of clamped voltage from -80 mV to 80 mV under no-TTX condition or 1 μM TTX(**S**) condition or 1 μM TTX(**C**) condition. Control group, n = 16 cells; 1 μM TTX(**S**) group, n= 16 cells; 1 μM TTX(**C**) group, n = 16 cells. **d**, The bar graph displays quantitative comparison of HEK-Na$_v$1.5 current densities at several clamped voltages under different TTX treatment. Control group, n = 16 cells; 1 μM TTX(**S**) group, n = 16 cells; 1 μM TTX(**C**) group, n = 16 cells. Error bars indicate mean ± SEM. T.Test, the exact p-values are provided on the figures .

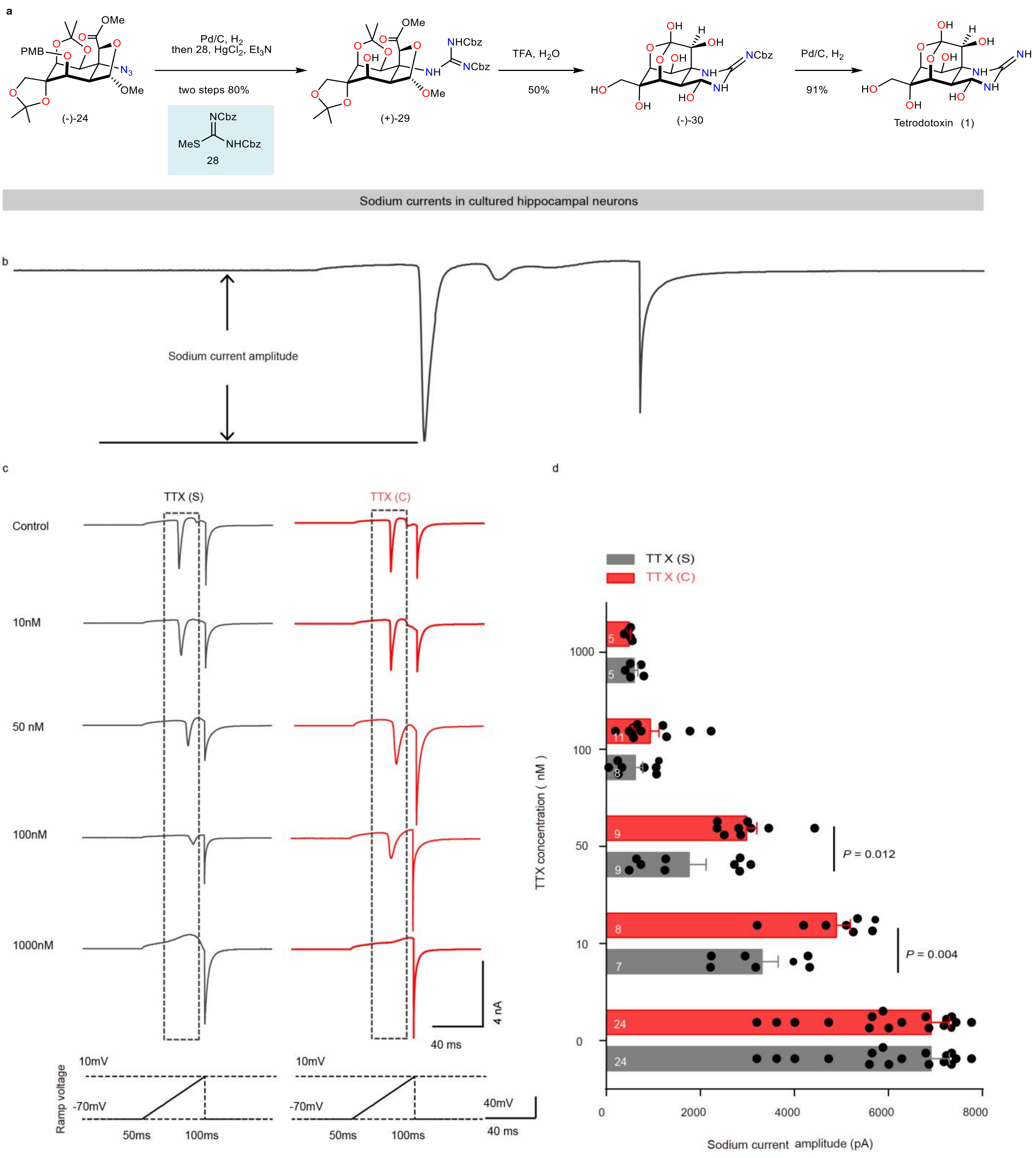

Supplementary Figure 5. Alternative synthesis of pure TTX and effects of TTX (**S**) and TTX (**C**) on depolarization-induced sodium currents. **a**, The procedure of preparing high purity TTX. **b**, Schematic diagram for sodium current evoked by a ramp voltage. **c**, Representative traces for sodium current amplitudes in primary cultured hippocampal neurons (DIV 14) after treatment with various TTX compounds. Black, TTX (**S**); Red, TTX (**C**). Ramp voltage from -70 mV to 10 mV over 50-ms. **d**, Quantitative analyses of sodium current amplitude in neurons treated with TTX (**S**) and TTX (**C**) with various concentrations. Cell numbers are marked on the columns. Error bars represent means ±SEM; two-tailed unpaired t-test, the exact p-values are provided on the figures.

3. Supplementary Methods

1. Detailed experimental procedures

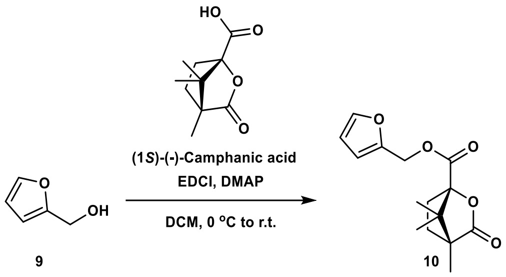

To a stirred solution of furfuryl alcohol **9** (39.6 g, 404.0 mmol, 1.00 eq.), (1*S*)-(-)-Camphanic acid (80.0 g, 404.0 mmol, 1.00 eq.) and DMAP (4.93 g, 40.4 mmol, 0.10 eq.) in CH_2Cl_2 (1400.0 mL), 1-ethyl-3-(3-dimethylaminopropyl) carbodiimide hydrochloride (EDCI·HCl) (116.6 g, 606.0 mmol, 1.50 eq.) was added at 0°C under argon atmosphere. Then the reaction mixture was warmed to room temperature naturally and stirred for 27 h. The reaction mixture was quenched with 0.5 N HCl solution (800 mL) and extracted with CH_2Cl_2 (3 × 800 mL). The combined organic extracts were dried over anhydrous Na_2SO_4, filtered and concentrated under reduced pressure to give a residue, which was recrystallized with EtOH and petroleum ether to afford compound **10** (101.0 g, 90% yield) as a white solid.

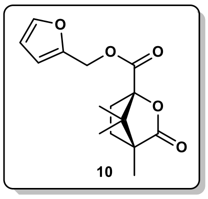

R_f = 0.33 (PE/EtOAc = 4:1)

$[\alpha]_D^{23}$ = -22.4 (*c* = 0.21, $CHCl_3$)

HRMS-ESI (m/z) calc. for $C_{15}H_{18}O_5Na$ $[M+Na]^+$: 301.1052; Found: 301.1043.

^{1}H NMR (400 MHz, $CDCl_3$, 25°C) *δ* 7.42 (dd, *J* = 1.8, 0.8 Hz, 1H), 6.45 (d, *J* = 3.3 Hz, 1H), 6.40 – 6.33 (m, 1H), 5.22 (q, *J* = 13.0 Hz, 2H), 2.43 (ddd, *J* = 14.2, 10.7, 4.2 Hz, 1H), 2.03 (ddd, *J* = 13.7, 9.5, 4.6 Hz, 1H), 1.96 – 1.85 (m, 1H), 1.68 (ddd, *J* = 13.4, 9.3, 4.1 Hz, 1H),1.10 (s, 3H), 1.00 (s, 3H), 0.88 (s, 3H).

^{13}C NMR (101 MHz, $CDCl_3$, 25°C) *δ* 177.96, 167.14, 148.64, 143.44, 111.33, 110.62, 90.96, 58.70, 54.71, 54.29, 30.48, 28.93, 16.67, 16.54, 9.67.

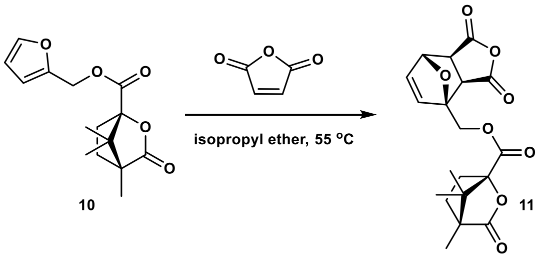

To a stirred solution of compound **10** (100.0 g, 360.0 mmol, 1.00 eq.) in isopropyl ether (290.0 mL), maleic anhydride (35.3 g, 360.0 mmol, 1.00 eq.) was added at room temperature under argon atmosphere. Then the reaction mixture was heated to 55 °C and stirred for 12 h. Additional 1.00 eq. maleic anhydride was added and kept stirred for more 24h until compound **10** was consumed (detected by crude ^{1}H NMR). Then the reaction mixture was filtered, the filter-cake

was washed with isopropyl ether to afford crude compound **11** (131.0 g, with 6% maleic anhydride) as a white solid, which was used in the following reaction without further purification.

Note 1: The compound **11** was sensitive to protic solvents, the whole process should be avoided to contact protic solvents such as water or methanol and so on.

Note 2: The reaction was sensitive to visible light, which should be performed under dark.

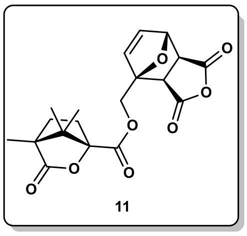

R_f = 0.33 (PE/EtOAc = 1:1)

$[\alpha]_D^{23}$ = +5.0 (*c* = 0.16, $CHCl_3$)

HRMS-ESI (m/z) calc. for $C_{19}H_{24}NO_8$ $[M+NH_4]^+$: 394.1502; Found: 394.1495.

^{1}H NMR (400 MHz, $CDCl_3$, 25°C) *δ* 6.65 (d, *J* = 5.7 Hz, 1H), 6.54 (d, *J* = 5.8 Hz, 1H), 5.46 (s, 1H), 4.97 (d, *J* = 12.4 Hz, 1H), 4.69 (d, *J* = 12.4 Hz, 1H), 3.31 (dd, *J* = 26.4, 6.9 Hz, 2H), 2.44 (ddd, *J* = 14.4, 10.5, 3.8 Hz, 1H), 2.04 (ddd, *J* = 13.7, 9.3, 4.5 Hz, 1H), 1.93 (ddd, *J* = 12.4, 11.0, 4.4 Hz, 1H), 1.69 (ddd, *J* = 13.3, 9.3, 4.1 Hz, 1H), 1.12 (s, 3H), 1.06 (s, 3H), 1.00 (s, 3H).

^{13}C NMR (101 MHz, $CDCl_3$, 25°C) *δ* 178.06, 169.11, 167.81, 166.85, 138.05, 137.34, 90.94, 90.11, 82.34, 61.48, 54.86, 54.50, 51.22, 49.66, 30.75, 28.84, 16.67, 16.62, 9.70.

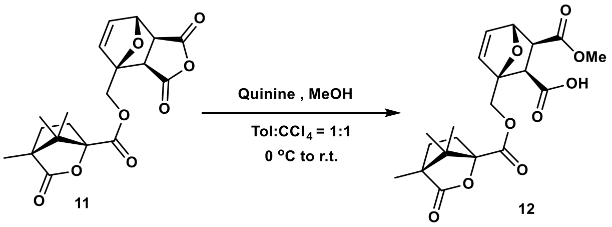

To a stirred solution of compound **11** (131.0 g, 348.0 mmol, 1.00 eq.) in toluene (750.0 mL) and CCl_4 (750.0 mL), quinine (112.0 g, 348.0 mmol, 1.00 eq.) and MeOH (55.7 g, 1740.0 mmol, 5.00 q.) was added at 0 °C under argon atmosphere sequentially. Then the reaction mixture was warmed to room temperature naturally and stirred for 22 h. The reaction mixture was concentrated under reduced pressure to give a residue and then was dissolved in CH_2Cl_2. The mixture was acidified with 1.0 N HCl solution to pH = 2 and then extracted with CH_2Cl_2 until no quinine in organic layer. The combined organic extracts were dried over anhydrous Na_2SO_4, filtered and concentrated under reduced pressure, which was re-crystallization with CH_2Cl_2 and petroleum ether to afford compound **12** (127.5 g, 87% yield for two steps) as a white solid.

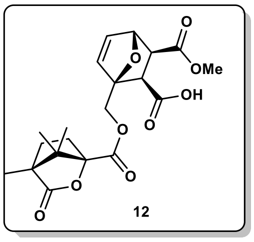

R_f = 0.4 (DCM/MeOH = 10:1)

$[\alpha]_D^{23}$ = +21.7 (*c* = 0.32, $CHCl_3$)

HRMS-ESI (m/z) calc. for $C_{20}H_{28}NO_9$ $[M+NH_4]^+$: 426.1764; Found: 426.1754.

^{1}H NMR (400 MHz, $CDCl_3$, 25°C) *δ* 6.56 (dd, *J* = 5.6, 1.6 Hz, 1H), 6.39 (d, *J* = 5.7 Hz, 1H), 5.46 (d, *J* = 1.7 Hz, 1H), 4.86 (d, *J* = 12.2 Hz, 1H), 4.70 (d, *J* = 12.1 Hz, 1H), 3.71 (s, 3H), 3.07 (d, *J* = 8.9 Hz, 1H), 2.95 (d, *J* = 9.0 Hz, 1H), 2.48 – 2.34 (m, 2H), 2.08 – 1.98 (m, 1H), 1.98 – 1.84 (m, 2H), 1.73 – 1.62 (m, 1H), 1.11 (s, 3H), 1.03 (s, 3H), 0.96 (s, 3H).

^{13}C NMR (101 MHz, CDCl$_3$, 25°C) *δ* 178.09, 175.59, 171.01, 166.85, 137.80, 136.59, 91.07, 89.02, 79.96, 62.30, 54.81, 54.32, 52.46, 49.94, 48.28, 30.65, 28.88, 16.72, 16.61, 9.66.

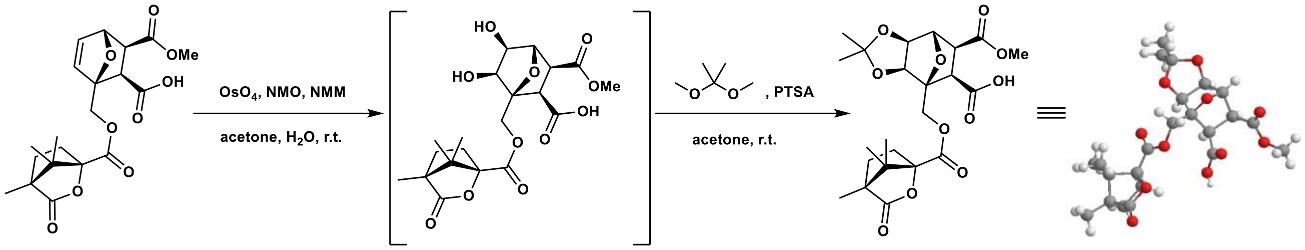

12 12a 13

To a stirred solution of compound **12** (70.0 g, 171.0 mmol, 1.00 eq.), *N*-methylmorpholine *N*-oxide (NMO) (30.0 g, 256.0 mmol, 1.50 eq.) and *N*-methylmorpholine (NMM) (19.0 g, 188.0 mmol, 1.10 eq.) in acetone (900.0 mL) and H_2O (36.0 mL), 0.05 M aq. OsO_4 (34.0 mL, 1.7 mmol, 0.01 eq.) was added slowly at room temperature under argon atmosphere and dark, then the mixture was stirred at room temperature for 3 h under dark. The mixture was quenched with sat. aq. Na_2SO_3 and then concentrated under reduced pressure to give a residue. The residue was acidified with 1.00 N HCl solution to pH = 2 and extracted with ethyl acetate (3 × 500 mL), the remained aqueous layer was extracted with *n*-BuOH until no product in organic layer. All combined organic layer was concentrated under reduced pressure to afford crude compound **12a** as a pale yellow foam, which was used in the following reaction without further purification.

To a stirred solution of compound **12a** in acetone (800.0 mL), 2,2-dimethoxypropane (26.6 g, 256.0 mmol, 1.50 eq.) and PTSA·H_2O (3.3 g, 17.0 mmol, 0.10 eq.) was added sequentially. Then the reaction mixture was stirred at room temperature for 3.5 h. 4.00 g $NaHCO_3$ solid was added to quench reaction and the mixture was concentrated under reduced pressure to give a residue. The residue was directly purified by flash chromatography on silica gel (CH_2Cl_2/MeOH= 20:1) to afford the compound **13** (63.7 g, 77% yield for two steps) as a white foam.

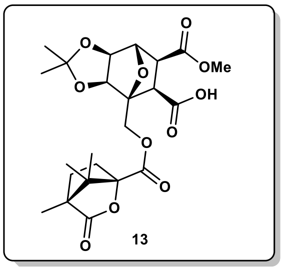

R$_f$ = 0.5 (DCM/MeOH = 10:1)

$[\alpha]_D^{23}$ = +0.8 (*c* = 0.36, CHCl$_3$)

mp: 135.2-136.4 °C

HRMS-ESI (m/z) calc. for $C_{23}H_{34}NO_{11}$ $[M+NH_4]^+$: 500.2132; Found: 500.2123.

^{1}H NMR (400 MHz, CDCl$_3$, 25°C) *δ* 9.67 (brs, 1H), 4.94 (s, 1H), 4.65 (d, *J* = 10.9 Hz, 1H), 4.55 (d, *J* = 10.9 Hz, 1H), 4.34 (dd, *J* = 20.7, 5.5 Hz, 2H), 3.66 (s, 3H), 3.07 (d, *J* = 9.6 Hz, 1H), 2.95 (d, *J* = 9.7 Hz, 1H), 2.42 (ddd, *J* = 14.7, 9.0, 4.1 Hz, 1H), 2.06 (ddd, *J* = 9.3, 6.9, 2.8 Hz, 1H), 1.98 – 1.85 (m, 1H), 1.69 (ddd, *J* = 17.5, 8.9, 4.3 Hz, 2H), 1.43 (s, 3H), 1.28 (s, 3H), 1.10 (s, 3H), 1.05 (s, 3H), 0.94 (s, 3H).

^{13}C NMR (101 MHz, CDCl$_3$, 25°C) *δ* 178.74, 174.08, 169.88, 166.47, 113.27, 91.26, 86.69, 81.78, 81.73, 80.46, 60.63, 54.88, 54.51, 52.47, 48.15, 47.43, 30.69, 28.93, 25.90, 25.61, 16.65, 16.52, 9.62.

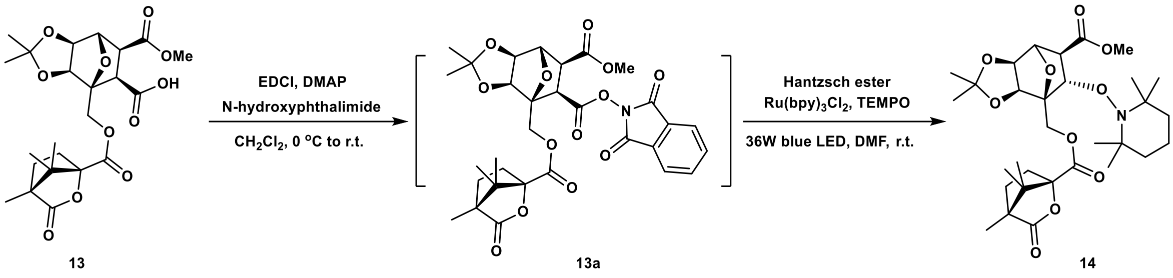

Method A:

To a stirred solution of the compound **13** (1.0 g, 2.1 mmol, 1.00 eq.), N-hydroxyphthalimide (506.0 mg, 3.1 mmol, 1.50 eq.) and DMAP (26.0 mg, 0.2 mmol, 0.10 eq.) in dry DMF (15.0 mL), 1-ethyl-3-(3-dimethylaminopropyl) carbodiimide hydrochloride (EDCI·HCl) (597.0 mg, 3.1 mmol, 1.50 eq.) was added at 0 °C under argon atmosphere. Then the reaction mixture was warmed to room temperature naturally and stirred under dark for 12 h. Then $Ru(bpy)_3Cl_2$ (64.0 mg, 0.1 mmol, 0.05 eq.), TEMPO (498.0 mg, 3.2 mmol, 1.54 eq.) and Hantzsch ester (524.0 mg, 2.1 mmol, 1.00 eq.) in dry DMF (5.0 mL) was added into above solution directly, the result solution was stirred vigorously at room temperature under 36 W Blue LEDs for 24h. Then the reaction mixture was concentrated under reduced pressure to give a residue. The obtained residue was subjected to column chromatography on silica gel (petroleum ether/ CH_2Cl_2, 3:1 to 1:1) to afford compound **14** (800.0 mg, 65% yield, d.r. > 95:5) as a pale yellow solid.

30 Grams Scale: To a stirred solution of the compound **13** (30.0 g, 62.2 mmol, 1.00 eq.), N-hydroxyphthalimide (15.2 g, 93.3 mmol, 1.50 eq.) and DMAP (756.0 mg, 6.2 mmol, 0.10 eq.) in CH_2Cl_2 (600.0 mL), 1-ethyl-3-(3-dimethylaminopropyl) carbodiimide hydrochloride (EDCI·HCl) (17.9 g, 93.3 mmol, 1.50 eq.) was added at 0 °C under argon atmosphere. Then the reaction mixture was warmed to room temperature naturally and stirred under dark for 24 h. The

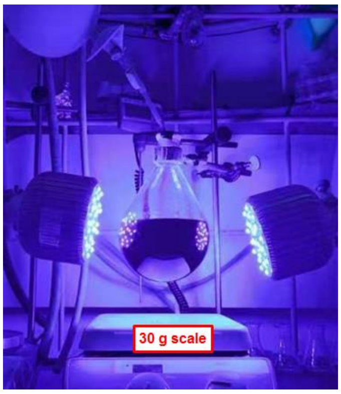

resulting mixture was quenched with sat. aq. NH_4Cl (800 mL) and then extracted with CH_2Cl_2 (3 × 500 mL). The combined organic extracts were dried over anhydrous Na_2SO_4, filtered and concentrated under reduced pressure to afford crude compound **13a**, which was directly used in the next step without further purification. The solution of the above crude NHPI ester **13a**, $Ru(bpy)_3Cl_2$ (1.91 g, 3.2 mmol, 0.05 eq.), TEMPO (14.9 g, 95.8 mmol, 1.54 eq.) and Hantzsch ester (15.7 g, 62.2 mmol, 1.00 eq.) in DMF (degassed, 500.0 mL) was stirred vigorously at room temperature under 36 W Blue LEDs for 24 h. Then the reaction mixture was concentrated under reduced pressure to give a residue. The obtained residue was subjected to column chromatography on silica gel (petroleum ether/CH_2Cl_2, 3:1 to 1:1) to afford compound **14** (22.7 g, 62% yield, d.r. > 95:5) as a pale yellow solid.

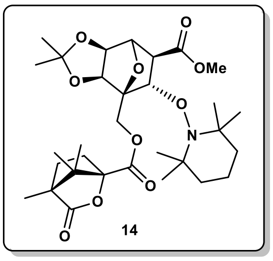

R_f = 0.4 (PE/EA = 4:1)

$[\alpha]_D^{23}$ = +7.3 (*c* = 0.23, $CHCl_3$)

HRMS-ESI (m/z) calc. for $C_{31}H_{48}NO_{10}$ $[M+H]^+$: 594.3278; Found: 594.3265.

^{1}H NMR (400 MHz, $CDCl_3$, 25°C) δ 5.06 – 5.01 (m, 2H), 4.80 (d, J = 5.6 Hz, 1H), 4.52 – 4.48 (m, 3H), 3.71 (s, 3H), 2.84 (d, J = 3.2 Hz, 1H), 2.54 – 2.45 (m, 1H), 2.03 (ddd, J = 13.7, 9.3, 4.5 Hz, 1H), 1.92 (ddd, J = 13.1, 10.8, 4.6 Hz, 1H), 1.67 (ddd, J = 13.4, 9.4, 4.2 Hz, 1H), 1.59 – 1.37 (m, 9H), 1.33 (s, 7H), 1.21 – 1.05 (m, 14H), 0.99 (s, 3H).

^{13}C NMR (101 MHz, $CDCl_3$, 25°C) *δ* 178.03, 171.84, 166.95, 112.84, 91.25, 86.62, 83.10, 82.70, 81.97, 78.62, 60.56, 54.82, 54.10, 52.31, 48.96, 40.09, 39.75, 34.34, 33.93, 30.62, 28.92, 26.03, 25.59, 20.52, 20.44, 16.87, 16.75, 9.71.

Method B (Flow chemistry procedure):

To a stirred solution of the compound **13** (1.5 g, 3.1 mmol, 1.00 eq.), N-hydroxyphthalimide (760.0 mg, 4.7 mmol, 1.50 eq.) and DMAP (37.0 mg, 0.3 mmol, 0.10 eq.) in CH_2Cl_2 (25.0 mL), 1-ethyl-3-(3-dimethylaminopropyl) carbodiimide hydrochloride (EDCI·HCl) (892.0 mg, 4.7 mmol, 1.50 eq.) was added at 0 °C under argon atmosphere. Then the reaction mixture was warmed to room temperature naturally and stirred under dark for 24 h. The resulting mixture was quenched with sat. aq. NH_4Cl (80 mL) and then extracted with CH_2Cl_2 (3 × 20 mL). The combined organic extracts were dried over anhydrous Na_2SO_4, filtered and concentrated under reduced pressure to afford crude compound **13a**, which was directly used in the next step without further purification.

New prepared NHPI ester **13a**, $Ru(bpy)_3Cl_2$ (96.0 mg, 0.16 mmol, 0.05 eq.), TEMPO (750.0 mg, 4.8 mmol, 1.54 eq.) and Hantzsch ester (785.0 mg, 3.1 mmol, 1.00 eq.), DMF (degassed, 40.0 mL) was added to a 100 mL round-bottomed flask and then the flow apparatus was inserted. The system was bubbled with argon for 10 min before reaction began. Then the reaction was proceeded with a 14 rpm flow rate under the irradiation of a 36 W blue LEDs for 24 h. The reaction mixture was concentrated under reduced pressure to give a residue, which was subjected to column chromatography on silica gel (petroleum ether/CH_2Cl_2, 3:1 to 1:1) to afford compound **14** (1.22 g, 66% yield, d.r. > 95:5) as a pale yellow solid.

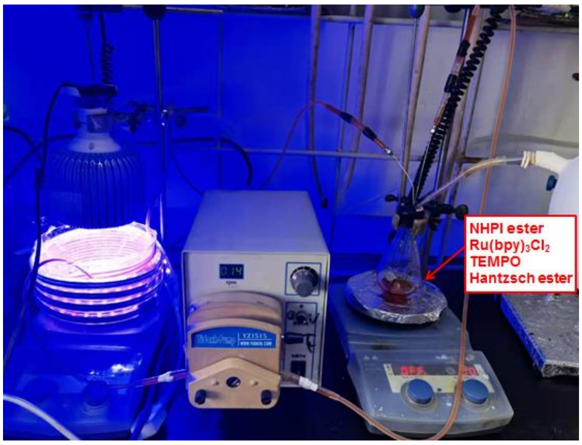

Materials used in flow procedure: Peristaltic pump (BT02-YZ1515), PFA tubing, silicone tubing, 36 W 450 nm LED and electric fan (all materials were purchased on Taobao).

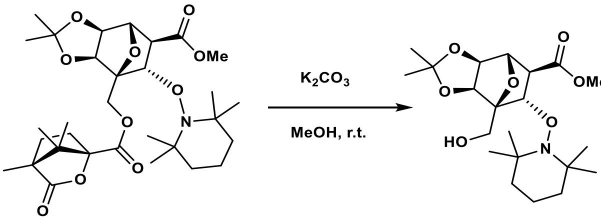

14 14a

To a stirred solution of the compound **14** (20.0 g，33.7 mmol, 1.00 eq.) in MeOH (350.0 mL), K_2CO_3 (6.9 g, 50.5 mmol, 1.50 eq.) was added at room temperature under argon atmosphere and then stirred at room temperature for 1 h. The mixture was quenched with AcOH (10 mL), then concentrated under reduced pressure to give a residue, which was subjected to column chromatography on silica gel (CH_2Cl_2/MeOH, 100:1 to 40:1) to afford compound **14a** (13.2 g, 95% yield) as a colorless oil.

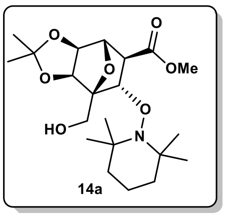

14a

R_f = 0.5 (DCM/MeOH = 20:1)

$[\alpha]_D^{23}$ = +10.9 (*c* = 0.24, $CHCl_3$)

HRMS-ESI (m/z) calc. for $C_{21}H_{36}NO_7$ $[M+H]^+$: 414.2492; Found: 414.2477.

^{1}H NMR (400 MHz, $CDCl_3$, 25°C) δ 4.94 (s, 1H), 4.79 (d, *J* = 6.3 Hz, 1H), 4.51 – 4.43 (m, 2H), 4.21 (d, *J* = 12.5 Hz, 1H), 4.01 (dd, *J* = 12.1, 9.9 Hz, 1H), 3.72 (s, 3H), 2.80 (d, *J* = 3.4 Hz, 1H), 2.33 (br s, 1H), 1.47 – 1.36 (m, 8H), 1.35 – 1.20 (m, 7H), 1.15 – 0.99 (m, 9H).

^{13}C NMR (101 MHz, $CDCl_3$, 25°C) δ 172.40, 112.29, 87.90, 82.89, 82.69, 82.39, 78.86, 61.07, 59.64, 59.22, 52.22, 48.63, 40.45, 40.05, 35.20, 34.19, 25.90, 25.28, 20.39, 20.22, 16.99.

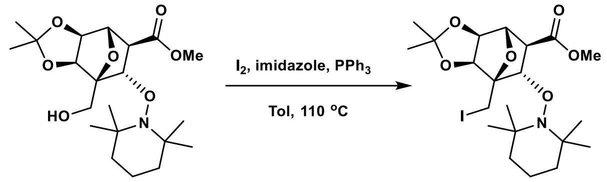

14a **15**

To a stirred solution of the compound **14a** (13.0 g, 31.5 mmol, 1.00 eq.), PPh_3 (30.6 g, 117.8 mmol, 3.74 eq.) and imidazole (6.7 g, 99.5 mmol, 3.16 eq.) in toluene (300.0 mL), I_2 (21.2 g, 83.8 mmol, 2.66 eq.) dissolved in toluene (100.0 mL) was added at room temperature under argon atmosphere. The reaction mixture was heated to 110 °C and then stirred at 110 °C for 2 h. The resulting mixture was cooled to room temperature and concentrated under reduced pressure to give a residue, which was subjected to column chromatography on silica gel (petroleum ether/ethyl acetate, 40:1 to 25:1) to afford compound **15** (14.6 g, 89% yield) as a colorless oil.

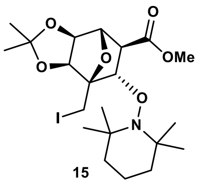

15

R_f = 0.4 (PE/EA = 10:1)

$[\alpha]_D^{23}$ = +21.0 (*c* = 0.26, $CHCl_3$)

HRMS-ESI (m/z) calc. for $C_{21}H_{35}INO_6$ $[M+H]^+$: 524.1509; Found: 524.1503.

^{1}H NMR (400 MHz, $CDCl_3$, 25°C) δ 4.98 (d, *J* = 3.3 Hz, 1H), 4.78 (d, *J* = 5.5 Hz, 1H), 4.63 (d, *J* = 5.5 Hz, 1H), 4.48 (s, 1H), 3.79 (d, *J* = 11.3 Hz, 1H), 3.75 (s, 3H), 3.56 (d, *J* = 11.5 Hz, 1H), 2.92 (d, *J* = 3.3 Hz, 1H), 1.54 (s, 3H), 1.50 – 1.30 (m, 12H), 1.22 – 1.00 (m, 9H).

^{13}C NMR (101 MHz, $CDCl_3$, 25°C) δ 171.92, 112.51, 86.51, 84.36, 83.98, 82.28, 77.92, 60.93, 59.33, 52.23, 48.39, 40.54, 40.03, 35.08, 26.01, 25.64, 20.45, 20.39, 16.96, 1.18.

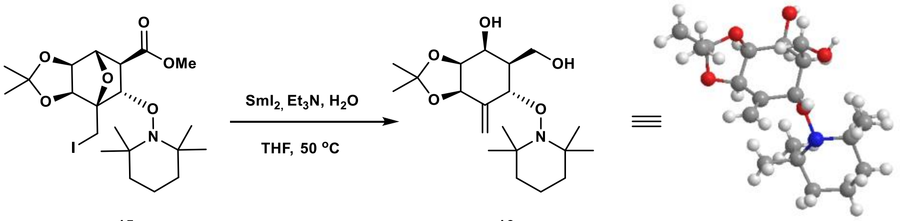

15 **16**

(1) Prepare 0.1 M SmI_2 solution in THF[3]

A flame-dried 1 L round bottomed flask was charged with Samarium metal (12.7 g, 86.0 mmol, 1.00 eq.) and a stir bar, then thoroughly degassed THF (860.0 mL) followed by iodine crystals (22.68 g, 86.0 mmol, 1.00 eq.) was added sequentially at room temperature under argon atmosphere. The reaction mixture was stirred vigorously at room temperature for over 2 h. As SmI_2 was generated, the color of solution changed from orange followed by yellow and green, eventually turned into navy-blue.

Note: In order to ensure full conversion, the solution should be stirred at least 2 h before using.

(2) SmI_2 induced reduction reaction

A flame-dried 500 mL round bottomed flask was charged with compound **15** (500.0 mg, 0.95 mmol, 1.00 eq.), Et_3N (318.0 μL, 23.00 mmol, 24.00 eq.), H_2O (413.0 μL, 23.00 mmol, 24.00 eq.) and THF (2.0 mL) and a stir bar. Above new prepared 0.1 M SmI_2 (115.0 mL, 11.50 mmol, 12.00 eq.) solution was transferred into the mixture using a double-ended needle. The whole progress needed to avoid air and carried out under argon atmosphere. Then the mixture was stirred at room temperature for 1.5 h. After the reaction was completed, the color changed to yellow. Then the reaction mixture was quenched with sat. aq. NH_4Cl (10 mL), washed with 0.1 N aq. HCl (2 mL) and extracted with ethyl acetate (3 × 10 mL). The combined organic extracts were dried over anhydrous Na_2SO_4, filtered and concentrated under reduced pressure to give a residue, which was purified with column chromatography on silica gel (CH_2Cl_2/MeOH, 80:1) to afford compound **16** (229.0 mg, 65% yield) as a white solid.

(3) Scalable preparation of compound 16

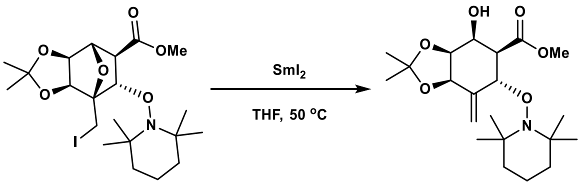

15 16a

SmI_2 solution in THF was prepared following the same process as mentioned before. New prepared SmI_2 solution in THF (860.0 mL) was transferred into a stirred solution of compound **15** (15.0 g, 28.7 mmol, 1.00 eq.) dissolved in THF (20.0 mL) using a double-ended needle. The whole progress needed to avoid air and carried out under argon atmosphere. Then the mixture was heated to 55 °C and stirred at 55 °C for 2 h. After the reaction was completed, the color changed to yellow. Then the reaction mixture was quenched with sat. aq. NH_4Cl (500 mL) and filtered to remove samarium salts. The resulting mixture was extracted with ethyl acetate (3 × 800 mL). The combined organic extracts were dried over anhydrous Na_2SO_4, filtered and concentrated under reduced pressure to afford crude compound **16a**, which was directly used in the next step without further purification.

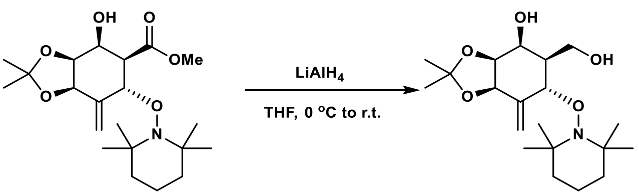

16a 16

To a stirred solution of the compound **16a** in THF (150.0 mL), the $LiAlH_4$ **(**1.1 g, 28.7 mmol, 1.00 eq.) was added slowly at 0 °C under argon atmosphere.

The mixture was warmed to room temperature naturally and stirred totally for 12 h. Excess $Na_2SO_4 \cdot 10H_2O$ was added slowly to quench reaction and then the mixture was filtered and concentrated under reduced pressure. The crude product compound was purified with column chromatography on silica gel (CH_2Cl_2/MeOH, 80:1) to afford compound **16** (6.1 g, 58% yield for two steps) as a white solid.

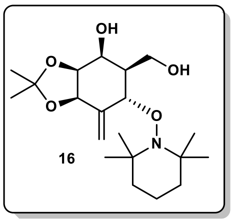

R_f = 0.2 (PE/EA = 2:1)

$[\alpha]_D^{23}$ = +57.5 (*c* = 0.30, $CHCl_3$)

mp: 57.8-58.9 °C

HRMS-ESI (m/z) calc. for $C_{20}H_{36}NO_5$ $[M+H]^+$: 370.2593; Found: 370.2585.

^{1}H NMR (400 MHz, $CDCl_3$, 25°C) *δ* 5.40 (s, 1H), 5.23 (s, 1H), 4.75 (d, *J* = 6.5 Hz, 1H), 4.51 (s, 1H), 4.45 – 4.38 (m, 1H), 4.30 (d, *J* = 3.9 Hz, 1H), 3.95 (dd, *J* = 10.7, 6.5 Hz, 1H), 3.76 (dd, *J* = 10.6, 6.8 Hz, 1H), 2.92 (d, *J* = 4.3 Hz, 1H), 2.56 – 2.46 (m, 1H), 1.57 – 0.96 (m, 24H).

^{13}C NMR (101 MHz, $CDCl_3$, 25°C) *δ* 142.43, 115.10, 109.62, 84.93, 76.78, 74.39, 67.62, 62.88, 60.48, 59.94, 45.20, 39.73, 33.82, 33.51, 27.09, 25.32, 20.17, 20.08, 16.61.

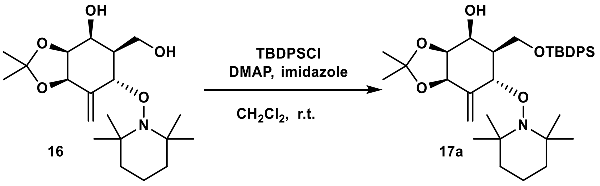

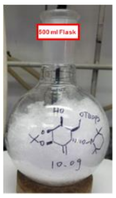

To a stirred solution of the compound **16** (7.2 g, 19.5 mmol, 1.00 eq.), imidazole (2.6 g, 39.0 mmol, 2.00 eq.) and DMAP (475.0 mg, 3.9 mmol, 0.20 eq.) in CH_2Cl_2 (100.0 mL), TBDPSCl (6.9 g, 25.3 mmol, 1.30 eq.) was added slowly at 0 °C under argon atmosphere. The mixture was stirred at room temperature for 2 h. MeOH (20 mL) was added to quench reaction, the resulting mixture was concentrated under reduced pressure and then subjected to column chromatography on silica gel (petroleum ether/ethyl acetate, 20:1 to 12:1) to afford compound **17a** (10.0 g, 85% yield) as a white foam.

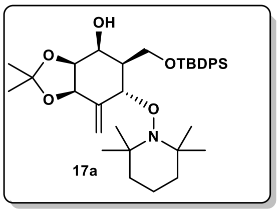

R_f = 0.4 (PE/EA = 10:1)

$[\alpha]_D^{23}$ = +23.3 (*c* = 0.22, $CHCl_3$)

HRMS-ESI (m/z) calc. for $C_{36}H_{54}NO_5Si$ $[M+H]^+$: 608.3771; Found: 608.3748.

^{1}H NMR (400 MHz, $CDCl_3$, 25°C) *δ* 7.70 – 7.63 (m, 4H), 7.43 – 7.30 (m, 6H), 5.36 (t, *J* = 2.1 Hz, 1H), 5.09 (t, *J* = 2.0 Hz, 1H), 4.76 – 4.69 (m, 1H), 4.51 – 4.41 (m, 2H), 4.33 (d, *J* = 3.8 Hz, 1H), 4.00 (dd, *J* = 10.1, 7.1 Hz, 1H), 3.62 (dd, *J* = 10.1, 7.7 Hz, 1H), 2.87 (d, *J* = 7.5 Hz, 1H), 2.82 – 2.75 (m, 1H), 1.58 – 0.91 (m, 33H).

^{13}C NMR (101 MHz, $CDCl_3$, 25°C) *δ* 142.58, 135.47, 133.15, 129.58, 129.55, 127.60, 116.38, 109.88, 86.31, 75.25, 66.86, 62.51, 60.26, 58.71, 46.55, 40.31, 34.59, 34.12, 27.69, 26.67, 26.19, 20.15, 20.08, 19.03, 17.00.

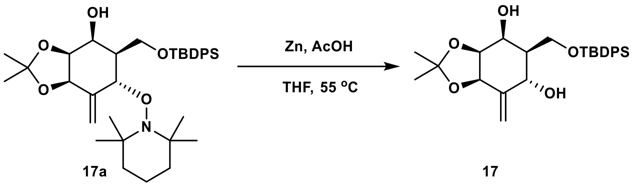

To a stirred solution of the compound **17a** (9.5 g, 15.6 mmol, 1.00 eq.) in THF (100.0 mL) and AcOH (130.0 mL), Zinc powder (41.0 g, 624.0 mmol, 40.00 eq.) was added at room temperature under argon atmosphere. The mixture was heated to 55 °C and stirred at 55 °C for 4.5 h. The resulting mixture was concentrated under reduced pressure and then subjected to column chromatography on silica gel (petroleum ether/ethyl acetate, 4:1) to afford compound **17** (6.5 g, 90% yield) as a white foam.

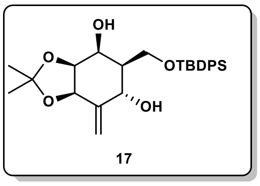

R_f = 0.2 (PE/EA = 4:1)

$[\alpha]_D^{23}$ = +32.2 (*c* = 1.4, $CHCl_3$)

HRMS-ESI (m/z) calc. for $C_{27}H_{36}O_5SiNa$ $[M+Na]^+$: 491.2230; Found: 491.2216.

^{1}H NMR (400 MHz, $CDCl_3$, 25°C) *δ* 7.76 – 7.61 (m, 4H), 7.49 – 7.34 (m, 6H), 5.42 – 5.27 (m, 2H), 4.80 (d, *J* = 6.9 Hz, 1H), 4.51 (d, *J* = 7.6 Hz, 1H), 4.31 (dd, *J* = 6.9, 4.3 Hz, 1H), 4.13 (t, *J* = 3.9 Hz, 1H), 4.06 – 3.94 (m, 2H), 2.66 (s, 2H), 2.08 – 1.95 (m, 1H), 1.47 (s, 3H), 1.41 (s, 3H), 1.07 (s, 9H).

^{13}C NMR (101 MHz, $CDCl_3$, 25°C) *δ* 145.09, 135.54, 132.79, 129.82, 127.79, 127.76, 115.07, 109.81, 76.76, 75.66, 70.10, 66.82, 65.13, 47.43, 26.79, 26.69, 25.17, 19.08.

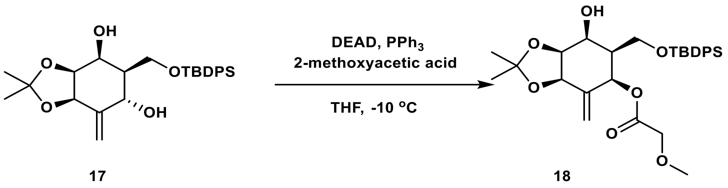

To a stirred solution of the compound **17** (6.4 g, 13.7 mmol, 1.00 eq.), 2-methoxyacetic acid (1.8 g, 20.5 mmol, 1.50 eq.) and PPh_3 (7.2 g, 27.4 mmol, 2.00 eq.) in dry THF (137.0 mL), diethyl azodicarboxylate (DEAD) (3.7 g, 20.5 mmol, 1.50 eq.) was added dropwise at -10 °C under argon atmosphere. The mixture was stirred at -10 °C for 12 h. The resulting mixture was concentrated under reduced pressure and subjected to column chromatography on silica gel (petroleum ether/ethyl acetate, 8:1 to 5:1) to afford compound **18** (6.6 g, 89% yield) as a colorless oil.

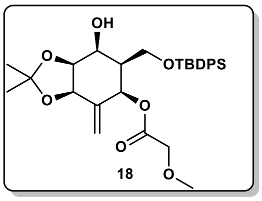

R_f = 0.4 (PE/EA = 4:1)

$[\alpha]_D^{23}$ = +18.4 (*c* = 0.35, $CHCl_3$)

HRMS-ESI (m/z) calc. for $C_{30}H_{40}O_7SiNa$ $[M+Na]^+$: 563.2441; Found: 563.2430.

^{1}H NMR (400 MHz, $CDCl_3$, 25°C) *δ* 7.71 – 7.63 (m, 4H), 7.47 – 7.34 (m, 6H), 5.43 (d, *J* = 4.1 Hz, 1H), 5.37 (d, *J* = 1.4 Hz, 1H), 5.10 (d, *J* = 1.4 Hz, 1H), 4.54 (d, *J* = 5.2 Hz, 1H), 4.44 – 4.38 (m, 1H), 4.35 – 4.25 (m, 1H), 4.17 (t, *J* = 3.9 Hz, 1H), 3.93 – 3.75 (m, 3H), 3.33 (s, 3H), 2.55 (s, 1H), 1.48 (s, 3H), 1.44 (s, 3H), 1.03 (s, 9H).

^{13}C NMR (101 MHz, CDCl$_3$, 25°C) δ 168.80, 140.30, 135.43, 132.72, 132.59, 129.85, 129.76, 127.72, 127.69, 113.10, 110.19, 77.58, 74.67, 70.36, 69.36, 68.26, 62.41, 59.27, 43.63, 27.49, 26.63, 26.00, 18.94.

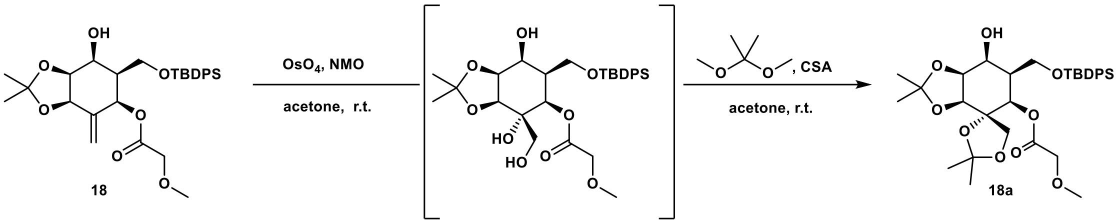

To a stirred solution of compound **18** (54.0 mg, 0.10 mmol, 1.00 eq.), 4-methylmorpholine N-oxide (14.0 mg, 0.12 mmol, 1.20 eq.) in acetone (2.0 mL), 0.05 M aq. OsO_4 (0.04 mL, 0.002 mmol, 0.02 eq.) was added at room temperature under argon atmosphere and dark, then the mixture was stirred for 24 h under dark. Then camphorsulfonic acid (CSA) (35.0 mg, 0.15 mmol, 1.50 eq.) and 2,2-dimethoxypropane (52.0 mg, 0.50 mmol, 5.00 eq.) was added, the mixture was stirred for 1 h at room temperature. The resulting mixture was quenched with sat. aq. Na_2SO_3 (0.2 mL) and then extracted with CH_2Cl_2 (3 × 2 mL). The combined organic layer was dried with Na_2SO_4, filtered and concentrated under reduced pressure to give a residue. The residue was purified by flash chromatography on silica gel (petroleum ether/ethyl acetate, 5:1) to afford compound **18a** (34.0 mg, 56% yield) as a white foam.

Scalable preparation of compound 18a: To a stirred solution of compound **18** (6.6 g, 12.2 mmol, 1.00 eq.), 4-methylmorpholine N-oxide (2.85 g, 24.4 mmol, 2.00 eq.) in acetone (70.0 mL), 0.05 M aq. OsO_4 (35.0 mL, 0.7 mmol, 0.06 eq.) was added slowly at room temperature under argon atmosphere and dark, then the mixture was stirred for 12 h. The resulting mixture was quenched with sat. aq. NH_4Cl (50 mL) and then extracted with CH_2Cl_2 (3 × 200 mL). The combined organic layer was dried with Na_2SO_4, filtered and concentrated under reduced pressure to afford crude compound as a pale yellow solid, which was used in the following reaction without further purification.

To a stirred solution of crude compound, 2,2-dimethoxypropane (2.54 g, 24.4 mmol, 2.00 eq.) in CH_2Cl_2 (80.0 mL), camphorsulfonic acid (CSA) (566.0 mg, 2.44 mmol, 0.20 eq.) was added at room temperature under argon atmosphere. Then the reaction mixture was stirred for 0.5 h. The resulting mixture was quenched with sat. aq. NH_4Cl (50 mL) and then extracted with CH_2Cl_2 (3 × 20 mL). The combined organic layer was dried with Na_2SO_4, filtered and concentrated under reduced pressure to give a residue. The residue was purified by flash chromatography on silica gel (petroleum ether/ethyl acetate, 5:1) to afford compound **18a** (4.9 g, 66% yield) as a white foam.

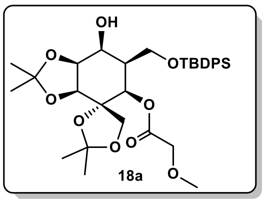

R_f = 0.4 (PE/EA = 4:1)

$[\alpha]_D^{23}$ = -4.1 (c = 0.43, CHCl$_3$)

HRMS-ESI (m/z) calc. for $C_{33}H_{46}O_9SiNa$ [M+Na]$^+$: 637.2809; Found: 637.2799.

^{1}H NMR (400 MHz, CDCl$_3$, 25°C) δ 7.70 – 7.59 (m, 4H), 7.48 – 7.29 (m, 6H), 5.52 (s, 1H), 4.26 – 4.19 (m, 2H), 4.15 (d, J = 5.5 Hz, 1H), 4.06 (dd, J = 9.4, 1.4 Hz, 1H), 3.90 – 3.95 (m, 3H), 3.84 – 3.72 (m, 2H), 3.33 (s, 3H), 2.52 (brs, 1H), 2.28 (t, J = 6.5 Hz, 1H), 1.56 (s, 3H), 1.44 (s, 6H), 1.41 (s, 3H), 1.05 (s, 9H).

^{13}C NMR (101 MHz, CDCl$_3$, 25°C) δ 168.86, 135.58, 133.25, 129.63, 129.59, 127.62, 127.60, 110.96, 109.65, 79.73, 77.81, 74.81, 71.89, 69.60, 69.10, 66.34,

61.50, 59.37, 40.45, 26.74, 26.68, 26.50, 25.74, 25.52, 19.10.

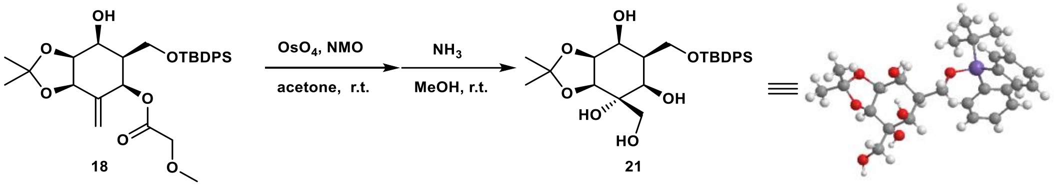

To a stirred solution of compound **18** (54.0 mg, 0.10 mmol, 1.00 eq.), 4-methylmorpholine N-oxide (23.4 mg, 0.20 mmol, 2.00 eq.) in acetone (1.0 mL), 0.05 M aq. OsO_4 (0.12 mL, 0.006 mmol, 0.06 eq.) was added slowly at room temperature under argon atmosphere and dark, then the mixture was stirred at room temperature for 13h. The resulting mixture was quenched with sat. aq. NH_4Cl (2 mL) and then extracted with CH_2Cl_2 (3 × 10 mL). The combined organic layer was dried with Na_2SO_4, filtered and concentrated under reduced pressure to afford crude compound as a pale yellow solid, which was used in the following reaction without further purification.

To a stirred solution of above crude product in MeOH (1.0 mL), 7 N NH_3 in MeOH (0.2 mL) was added at room temperature under argon atmosphere. Then the mixture was stirred for 2 h. The resulting mixture was quenched with H_2O (10 mL) and extracted with CH_2Cl_2 (3 × 10 mL). The combined organic layer was dried with Na_2SO_4, filtered and concentrated under reduced pressure to give a residue. The residue was purified by flash chromatography on silica gel (petroleum ether/ethyl acetate, 1:1) to afford compound **21** (37.5 mg, 75% yield) as a white solid.

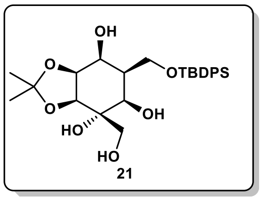

R_f = 0.2 (PE/EA = 1:1)

$[\alpha]_D^{23}$ = -14.3 (*c* = 0.22, $CHCl_3$)

mp:124.3-124.8 °C

HRMS-ESI (m/z) calc. for $C_{27}H_{38}O_7SiNa$ $[M+Na]^+$: 525.2285; Found: 525.2278.

^{1}H NMR (400 MHz, $CDCl_3$, 25°C) δ 7.69 (m, 4H), 7.45 – 7.34 (m, 6H), 4.28 (dd, *J* = 6.1, 5.0 Hz, 1H), 4.20 (d, *J* = 5.7 Hz, 2H), 4.13 – 3.99 (m, 3H), 3.97 (d, *J* = 10.7 Hz, 1H), 3.88 (d, *J* = 11.5 Hz, 1H), 3.70 (d, *J* = 10.6 Hz, 1H), 2.93 (s, 1H), 2.73 (s, 1H), 2.51 (s, 1H), 2.17 (t, *J* = 7.3 Hz, 1H), 1.50 (s, 3H), 1.39 (s, 3H), 1.06 (s, 9H).

^{13}C NMR (101 MHz, $CDCl_3$, 25°C) δ 135.57, 133.63, 129.60, 127.64, 109.24, 74.53, 73.33, 70.98, 67.25, 64.83, 62.54, 38.60, 26.85, 26.18, 24.72, 19.26.

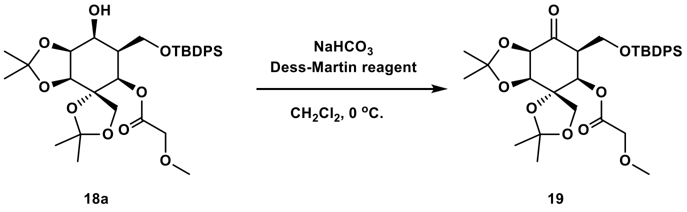

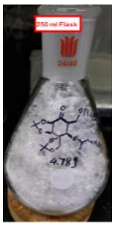

To a stirred solution of compound **18a** (4.89 g, 8.0 mmol, 1.00 eq.), $NaHCO_3$ (2.69 g, 32.0 mmol, 4.00 eq.) in CH_2Cl_2 (22.0 mL), Dess-Martin reagent (5.38 g, 12.7 mmol, 1.60 eq.) was added at 0 °C under argon atmosphere. The solution was stirred at room temperature for 3 h. The resulting mixture was directly

purified by flash chromatography on silica gel (petroleum ether/ethyl acetate, 6:1) to afford compound **19** (4.78 g, 98% yield) as a white foam.

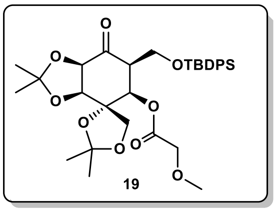

19

R$_f$ = 0.5 (PE/EA = 4:1)

[α]$_D^{23}$ = +13.0 (*c* = 0.24, $CHCl_3$)

HRMS-ESI (m/z) calc. for $C_{33}H_{44}O_9SiNa$ $[M+H]^+$: 635.2653; Found: 635.2632.

^{1}H NMR (400 MHz, $CDCl_3$, 25°C) *δ* 7.67 – 7.57 (m, 4H), 7.46 – 7.31 (m, 6H), 5.91 (t, *J* = 2.3 Hz, 1H), 4.47 (d, *J* = 5.9 Hz, 1H), 4.41 (dd, *J* = 5.8, 2.1 Hz, 1H), 4.23 (s, 2H), 3.96 (dd, *J* = 10.9, 4.8 Hz, 1H), 3.91 – 3.76 (m, 2H), 3.62 (dd, *J* = 10.9, 10.0 Hz, 1H), 3.45 – 3.39 (m, 1H), 3.37 (s, 3H), 1.55 (s, 3H), 1.51 (s, 3H), 1.36 (s, 3H), 1.35 (s, 3H), 1.04 (s, 9H).

^{13}C NMR (101 MHz, $CDCl_3$, 25°C) *δ* 205.28, 169.14, 135.62, 135.52, 133.04, 132.81, 129.75, 129.71, 127.69, 111.47, 111.00, 82.03, 78.77, 78.52, 74.47, 69.31, 69.01, 59.28, 57.62, 50.18, 27.02, 26.72, 26.53, 26.09, 19.07.

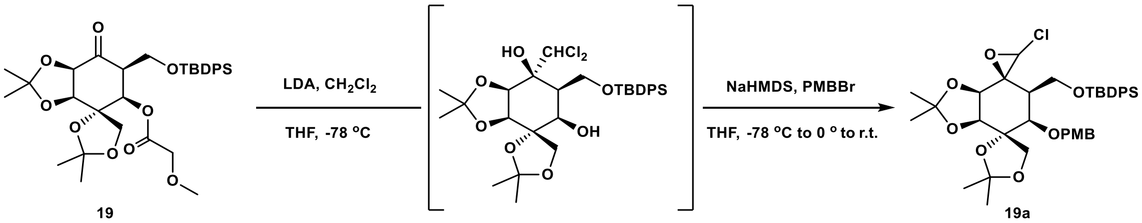

To a stirred solution of diisopropylamine (4.2 g, 41.6 mmol, 6.40 eq.) in dry THF (42.0 mL), *n*-butyllithium (2.5 M solution in hexane) (16.6 ml, 41.6 mmol, 6.40 eq.) was added dropwise at -78 °C under argon atmosphere. The reaction mixture was stirred at -78 °C for 1 h and then dry CH_2Cl_2 (11.6 g, 136.5 mmol, 21.00 eq.), a solution of compound **19** (4.0 g, 6.5 mmol, 1.00 eq.) in dry THF (50.0 mL) was added dropwise at -78 °C under argon atmosphere. Then the mixture was stirred at -78 °C until the disappearance of **19** on TLC with 4:1 PE/EtOAc. The resulting mixture was quenched by sat. aq. NH_4Cl (20 mL), extracted with EtOAc (3 × 50 mL). The combined organic layer was dried over anhydrous $MgSO_4$, concentrated under reduced pressure to afford crude compound as a pale yellow oil, which was used in the following reaction without further purification.

To a stirred solution of crude compound in dry THF (15.0 mL), NaHMDS (2.0 M in THF, 8.1 mL, 16.2 mmol, 2.50 eq.) was added dropwise at -78 °C under argon atmosphere. The reaction mixture was stirred at -78 °C for 1 h, then warmed to 0 °C and stirred at 0 °C for 1 h, then the mixture was cooled to -78 °C again and PMBBr (3.3 g, 16.2 mmol, 2.50 eq.) was added dropwise. The mixture was stirred at -78 °C for 1 h and then warmed to 0 °C, stirred at 0 °C for 1 h, then warmed to room temperature and stirred at room temperature for 12 h. The resulting mixture was quenched with sat. aq. NH_4Cl (50 mL), extracted with EtOAc (3 × 10 mL). The combined organic layer was dried over anhydrous $MgSO_4$, filtered and concentrated under reduced pressure to give a residue, which was purified by flash chromatography on silica gel (PE/EtOAc, 10:1) to give compound **19a** (2.76 g, 60% yield) as a colorless oil.

NOTE: The one pot synthesis procedure: To a stirred solution of diisopropylamine (51.7 mg, 0.51 mmol, 6.40 eq.) in dry THF (1.0 mL), *n*-butyllithium (2.5 M solution in hexane) (0.2 ml, 0.51 mmol, 6.40 eq.) was added dropwise at -78 °C under argon atmosphere. The reaction mixture was stirred at -78 °C for 40 min and then dry CH_2Cl_2 (142.8 mg, 1.68 mmol, 21.00 eq.), a solution of compound **19** (50.0 mg, 0.08 mmol, 1.00 eq.) in dry THF (5.0 mL) was added dropwise at -78 °C under argon atmosphere. Then the mixture was stirred at -78 °C for 3 h. Then NaHMDS (2.0 M in THF, 0.1 mL, 0.2 mmol, 2.50 eq.) was

added dropwise at -78 °C under argon atmosphere. The reaction mixture was stirred at -78 °C for 1 h, then warmed to 0 °C and stirred at 0 °C for 1 h, then the mixture was cooled to -78 °C again and PMBBr (40.2 mg, 0.2 mmol, 2.50 eq.) was added dropwise. The mixture was stirred at -78 °C for 1 h and then warmed to 0 °C, stirred at 0 °C for 1 h, then warmed to room temperature and stirred at room temperature for 12h. The resulting mixture was quenched with sat. aq. NH_4Cl (10.0 mL), extracted with EtOAc (3 × 10 mL). The combined organic layer was dried over anhydrous $MgSO_4$, filtered and concentrated under reduced pressure to give a residue, which was purified by flash chromatography on silica gel (PE/EtOAc, 10:1) to give compound **19a** (32.4 mg, 56% yield).

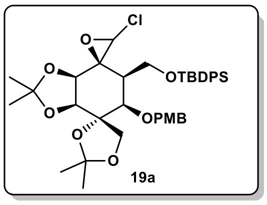

R_f = 0.6 (PE/EA = 2:1)

$[\alpha]_D^{22}$ = +1.54 (*c* = 0.50, $CHCl_3$)

HRMS-ESI (m/z) calc. for $C_{39}H_{49}ClO_8SiNa$ $[M+Na]^+$: 731.2783; Found: 731.2773.

^{1}H NMR (400 MHz, $CDCl_3$, 25°C) δ 7.65 – 7.57 (m, 4H), 7.39 (dddd, *J* = 9.3, 8.1, 4.2, 1.8 Hz, 6H), 7.20 (d, *J* = 8.7 Hz, 2H), 6.80 (d, *J* = 8.7 Hz, 2H), 4.91 (s, 1H), 4.62 (d, *J* = 11.1 Hz, 1H), 4.50 (d, *J* = 11.1 Hz, 1H), 4.44 (d, *J* = 6.4 Hz, 1H), 4.27 – 4.23 (m, 1H), 4.22 (s, 1H), 4.11 (d, *J* = 9.5 Hz, 1H), 3.89 (dd, *J* = 10.4, 7.7 Hz, 1H), 3.80 (dd, *J* = 4.2, 1.5 Hz, 1H), 3.78 (s, 3H), 3.69 (dd, *J* = 10.4, 5.8 Hz, 1H), 2.41 (ddd, *J* = 7.6, 5.7, 4.2 Hz, 1H), 1.42 (s, 3H), 1.37 (s, 3H), 1.35 (s, 3H), 1.30 (s, 3H), 1.05 (s, 9H).

^{13}C NMR (101 MHz, $CDCl_3$, 25°C) δ 158.89, 135.51, 135.46, 133.17, 130.55, 129.85, 129.78, 128.73, 127.78, 127.75, 113.49, 110.54, 110.40, 79.84, 78.85, 78.72, 74.59, 72.41, 70.55, 70.06, 60.74, 59.81, 55.22, 41.65, 29.70, 27.07, 26.87, 26.63, 25.52, 19.11.

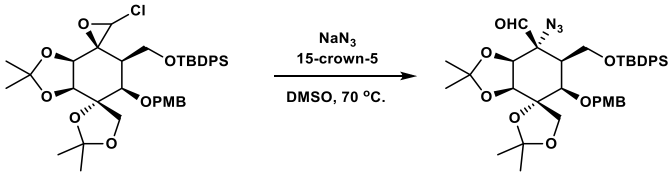

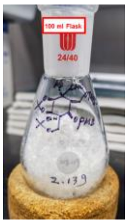

19a 20

To a stirred solution of compound **19a** (2.7 g, 3.8 mmol, 1.00 eq.), sodium azide (1.48 g, 22.8 mmol, 6.00 eq.) in dry dimethylsulfoxide (38.0 mL), 15-crown-5 ether (2.5 g, 11.4 mmol, 3.00 eq.) was added at room temperature under argon atmosphere. The reaction mixture was heated to 70 °C and stirred for 21 h. After the disappearance of **19a** on TLC with 4:1 petroleum ether: ethyl acetate, the reaction mixture was quenched with sat. aq. NH_4Cl (30 mL), extracted with CH_2Cl_2 (3 × 10 mL). The combined organic layer was dried over anhydrous $MgSO_4$, filtered and concentrated under reduced pressure to give a residue, which was purified by flash chromatography on silica gel (petroleum ether/ethyl acetate, 20:1) to give compound **20** (2.13 g, 77% yield) as a white foam.

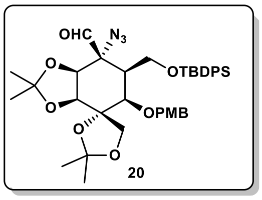

R_f = 0.5 (PE/EA = 2:1)

$[\alpha]_D^{22}$ = -46.6 (*c* = 0.13, $CHCl_3$)

HRMS-ESI (m/z) calc. for $C_{39}H_{49}N_3O_8SiNa$ $[M+Na]^+$: 738.3187; Found: 738.3173.

^{1}H NMR (400 MHz, $CDCl_3$, 25°C) δ 9.67 (s, 1H), 7.65 – 7.58 (m, 4H), 7.48 – 7.30 (m, 6H), 7.10 (d, *J* = 8.3 Hz, 2H), 6.83 (d, *J* = 7.9 Hz, 2H), 4.61 (q, *J* = 11.2 Hz, 2H), 4.50 (d, *J* = 6.4 Hz, 1H), 4.38 (d, *J* = 6.4 Hz, 1H), 4.29 – 4.14 (m, 3H), 3.95 (dd, *J* = 10.4, 3.9 Hz, 1H), 3.80 (s, 3H),

3.52 (t, J = 10.8 Hz, 1H), 2.34 (d, J = 10.3 Hz, 1H), 1.47 (s, 3H), 1.46 (s, 3H), 1.38 (s, 3H), 1.36 (s, 3H), 1.08 (s, 9H).

^{13}C NMR (101 MHz, CDCl$_3$, 25°C) δ 197.96, 159.21, 135.54, 135.46, 133.12, 132.82, 129.83, 128.72, 127.79, 127.75, 113.77, 111.13, 110.26, 80.18, 79.72, 79.06, 76.53, 75.89, 74.35, 69.41, 58.77, 55.25, 45.80, 26.99, 26.93, 26.57, 25.51, 24.63, 19.22.

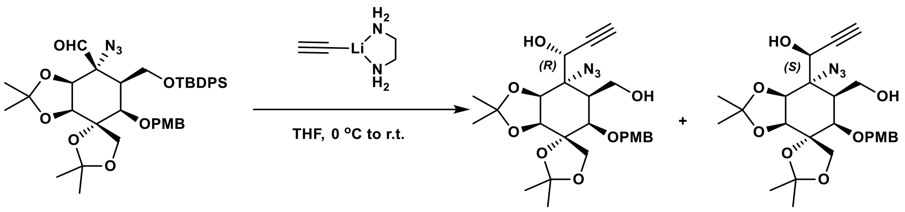

20 **22** **22a**

To a stirred solution of lithium acetylide ethylenediamine complex (180.0 mg, 1.96 mmol, 10.00 eq.) in THF (2.5 mL), compound **20** (140.0 mg, 0.20 mmol, 1.00 eq.) dissolved in THF (2.5 mL) was added dropwise at 0 °C under argon atmosphere. Then the mixture was stirred at 0 °C for 1 h and quenched with sat. aq. NH_4Cl (2 mL), extracted with EtOAc (3 × 10 mL). The combined organic layer was dried over anhydrous $MgSO_4$, filtered and concentrated under reduced pressure to give a residue, which was purified by flash chromatography on silica gel (petroleum ether/ethyl acetate, 2:1) to afford compound **22a** (65.0 mg, 66% yield) as colorless oil and its diastereomer **22** (**22a:22** = 15:1, the ratio was determined through crude ^{1}H NMR).

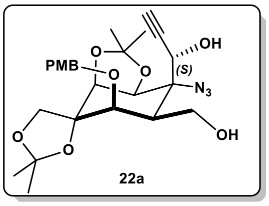

22a

R_f = 0.2 (PE/EA = 2:1)

$[\alpha]_D^{22}$ = +35.4 (c = 0.03, $CHCl_3$)

HRMS-ESI (m/z) calc. for $C_{25}H_{33}N_3O_8Na$ $[M+Na]^+$: 526.2166; Found: 526.2154.

^{1}H NMR (400 MHz, CDCl$_3$, 25°C) δ 7.31 (d, J = 8.4 Hz, 2H), 6.89 (d, J = 8.0 Hz, 2H), 5.00 (d, J = 1.3 Hz, 1H), 4.76 (d, J = 11.1 Hz, 1H), 4.62 (d, J = 11.2 Hz, 1H), 4.26 (d, J = 6.3 Hz, 1H), 4.13 (d, J = 6.4 Hz, 1H), 4.01 – 3.90 (m, 5H), 3.82 – 3.75 (m, 4H), 2.68 – 2.60 (m, 1H), 2.53 (d, J = 2.2 Hz, 1H), 1.56 (s, 3H), 1.47 (s, 3H), 1.39 (s, 3H), 1.33 (s, 3H).

^{13}C NMR (101 MHz, CDCl$_3$, 25°C) δ 159.40, 129.80, 129.56, 113.90, 109.23, 108.64, 85.91, 81.59, 79.81, 75.11, 74.62, 74.27, 66.75, 66.30, 64.89, 58.13, 55.27, 43.29, 29.69, 27.04, 26.26, 26.07, 24.43.

Epimerization of 22a to 22

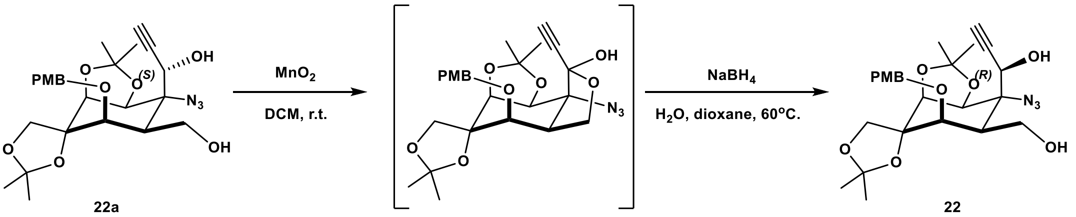

22a **22**

To a stirred solution of compound **22a** (148.0 mg, 0.29 mmol, 1.00 eq.) in CH_2Cl_2 (5.0 mL), MnO_2 (121.0 mg, 1.40 mmol, 10.00 eq.) was added at room temperature under argon atmosphere. Then the reaction mixture was stirred at room temperature for 2 h. The resulting mixture was filtered and concentrated

under reduced pressure to give a crude product, which was used in the following reaction without further purification.

To a stirred solution of the above resulted oxidation product in dioxane (2.0 mL) and H_2O (200.0 uL), $NaBH_4$ (421.0 mg, 1.11 mmol, 4.00 eq.) was added at room temperature under argon atmosphere. Then the reaction mixture was stirred at 60 °C for 0.5 h. The resulting mixture was quenched with sat. aq. NH_4Cl (2 mL), extracted three times (3 × 10 mL) with CH_2Cl_2. The combined organic layer was dried over anhydrous $MgSO_4$, filtered and concentrated under reduced pressure to give a residue, which was purified by flash chromatography on silica gel (petroleum ether/ethyl acetate, 2:1) to afford compound **22** (76.4 mg, 51%, 70% brsm yield for two steps) as a colorless oil and **22a** (39.1 mg, 26% yield) as a white foam. (**22:22a** = 2:1, the ratio was determined through crude ^1H NMR).

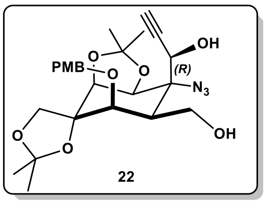

22

R_f = 0.25 (PE/EA = 2:1)

$[\alpha]_D^{22}$ = +22.8 (*c* = 0.18, $CHCl_3$)

mp: 48.3-49.6 °C

HRMS-ESI (m/z) calc. for $C_{25}H_{33}N_3O_8Na$ $[M+Na]^+$: 526.2166; Found: 526.2156.

^1H NMR (400 MHz, $CDCl_3$, 25ºC) δ 7.30 (d, *J* = 8.5 Hz, 2H), 6.88 (d, *J* = 8.4 Hz, 2H), 4.83 (d, *J* = 1.9 Hz, 1H), 4.73 (d, *J* = 10.7 Hz, 1H), 4.64 (d, *J* = 10.7 Hz, 1H), 4.36 (dd, *J* = 16.6, 6.8 Hz, 2H), 4.20 (dd, *J* = 21.0, 8.2 Hz, 2H), 4.13 (d, *J* = 4.4 Hz, 1H), 3.80 (s, 3H), 3.78 – 3.74 (m, 1H), 3.69 (dd, *J* = 11.8, 5.7 Hz, 1H), 2.65 – 2.63 (m, 1H), 2.55 (dd, *J* = 12.3, 5.5 Hz, 2H), 1.56 (s, 3H), 1.48 (s, 3H), 1.40 (s, 3H), 1.37 (s, 3H).

^{13}C NMR (101 MHz, $CDCl_3$, 25ºC) δ 159.49, 129.78, 129.30, 113.91, 109.56, 108.50, 85.45, 81.54, 79.63, 78.40, 76.76, 74.89, 74.75, 65.85, 65.74, 65.51, 59.22, 55.25, 43.89, 29.68, 26.87, 26.26, 26.09, 24.76.

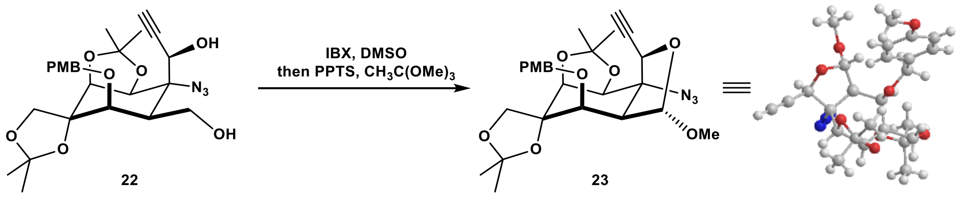

22 23

To a stirred solution of compound **22** (120.0 mg, 0.24 mmol, 1.00 eq.) in DMSO (5.0 mL), IBX (70.0 mg, 0.25 mmol, 1.05 eq.) was added at room temperature under argon atmosphere. Then the reaction mixture was stirred at room temperature for 2 h. After the disappearance of **22** on TLC with 4:1 petroleum ether/EtOAc, MeOH (5.0 mL), pyridinium *p*-toluenesulfonate (PPTS) (90.0 mg, 0.36 mmol, 1.50 eq.) and trimethyl orthoacetate (1.4 mL) was added into the above solution and then the solution was stirred at room temperature for 12 h. The mixture was quenched with sat. aq. $NaHCO_3$ (10 mL), extracted with CH_2Cl_2 (3 × 20 mL). The combined organic layer was dried over anhydrous $MgSO_4$, filtered and concentrated under reduced pressure to give a residue, which was purified by flash chromatography on silica gel (petroleum ether/ethyl acetate, 8:1) to afford compound **23** (108.0 mg, 88% yield) as a white foam.

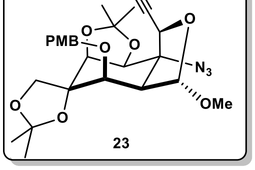

23

R_f = 0.4 (PE/EA = 4:1)

$[\alpha]_D^{22}$ = +9.5 (*c* = 0.05, $CHCl_3$)

HRMS-ESI (m/z) calc. for $C_{26}H_{33}N_3O_8Na$ $[M+Na]^+$: 538.2166; Found: 538.2158.

^{1}H NMR (400 MHz, CDCl$_3$, 25°C) δ 7.23 (d, J = 8.7 Hz, 2H), 6.84 (d, J = 8.7 Hz, 2H), 5.37 (d, J = 4.2 Hz, 1H), 4.85 (d, J = 2.2 Hz, 1H), 4.51 – 4.40 (m, 3H), 4.23 – 4.09 (m, 3H), 3.88 (dd, J = 8.6, 1.4 Hz, 1H), 3.79 (s, 3H), 3.40 (s, 3H), 2.80 (ddd, J = 8.5, 4.2, 0.9 Hz, 1H), 2.61 (d, J = 2.3 Hz, 1H), 1.39 (s, 3H), 1.37 (s, 3H),1.26 (s, 3H), 1.26 (s, 3H).

^{13}C NMR (101 MHz, CDCl$_3$, 25°C) δ 159.52, 130.29, 129.10, 113.72, 111.08, 110.07, 107.08, 79.55, 77.38, 76.81, 76.62, 75.79, 75.17, 74.95, 73.62, 69.17, 67.74, 56.03, 55.23, 47.35, 27.13, 26.34, 24.73, 24.18.

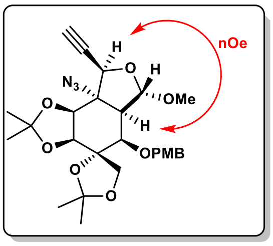

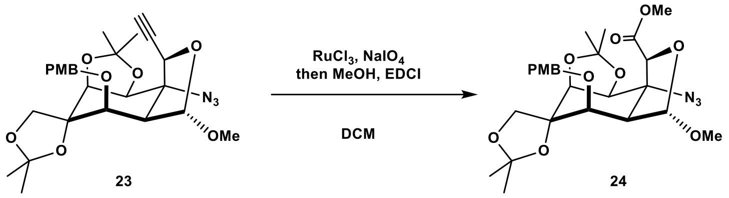

To a stirred solution of compound **23** (27.0 mg, 0.05 mmol, 1.00 eq.), $NaIO_4$ (45.0 mg, 0.21 mmol, 4.00 eq.) in CCl_4 (1.0 mL), MeCN (1.0 mL) and H_2O (1.5 mL), $RuCl_3{\cdot}xH_2O$ (2.2 mg, 0.011 mmol, 0.20 eq.) was added at room temperature under argon atmosphere. Then the reaction mixture was stirred at room temperature for 2 h. Then H_2O (2.0 mL) and CH_2Cl_2 (2.0 mL) was added into above solution. The residue was partitioned, the separated organic layer was used directly, EDCI·HCl (20.0 mg, 0.10 mmol, 2.00 eq.), DMAP (1.2 mg, 0.009 mmol, 0.20 eq.), MeOH (16.8 mg, 0.52 mmol, 10.00 eq.) was added into above organic solution at room temperature under argon atmosphere. The reaction mixture was stirred at room temperature for 2 h and then concentrated under reduced pressure to give a residue, which was purified by flash chromatography on silica gel (PE/EtOAc, 8/1 to 4/1) to afford compound **24** (13.2 mg, 48% yield) as a white foam.

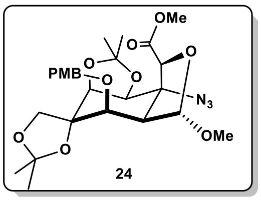

R_f = 0.25 (PE/EA = 4:1)

$[\alpha]_D^{22}$ = -8.0 (c = 0.05, CHCl$_3$)

HRMS-ESI (m/z) calc. for $C_{26}H_{39}N_4O_{10}$ $[M+NH_4]^+$: 567.2666; Found: 567.2658

^{1}H NMR (400 MHz, CDCl$_3$, 25°C) δ 7.23 (d, J = 8.4 Hz, 2H), 6.86 (d, J = 8.6 Hz, 2H), 5.31 (d, J = 3.3 Hz, 1H), 4.72 (s, 1H), 4.49 (d, J = 7.2 Hz, 3H), 4.20 (dd, J = 18.2, 7.9 Hz, 2H), 4.04 (d, J = 9.6 Hz, 1H), 3.89 (d, J = 6.4 Hz, 1H), 3.80 (d, J = 1.8 Hz, 6H), 3.43 (s, 3H), 2.83 (dd, J = 7.5, 3.3 Hz, 1H), 1.39 (s, 3H), 1.37 (s, 3H), 1.22 (s, 3H), 1.19 (s, 3H).

^{13}C NMR (101 MHz, CDCl$_3$, 25°C) δ 168.60, 159.46, 130.05, 129.29, 113.78, 110.76, 109.19, 106.14, 83.08, 79.15, 76.19, 75.30, 73.57, 69.24, 67.71, 55.99, 55.26, 52.12, 50.72, 27.02, 26.47, 24.73, 24.08.

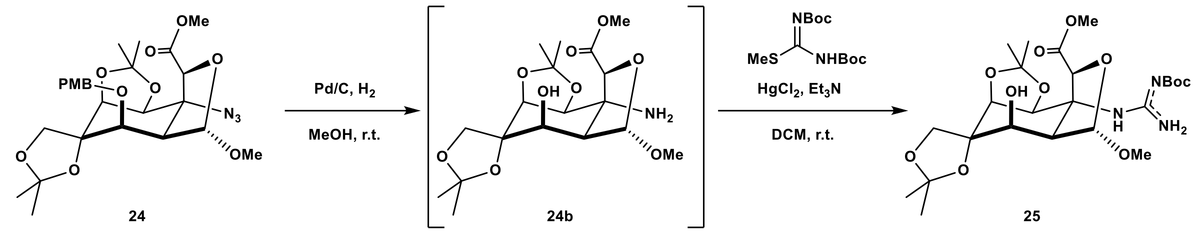

A suspension of Compound **24** (46.0 mg, 0.084 mmol, 1.00 eq.) and Pd-C (10 mg, 20 wt%) in MeOH (4.0 mL) was stirred at room temperature for 24 h under H_2 atmosphere (balloon). The mixture was passed through a Celite® pad, the filtrate was evaporated to give the crude product **24b**, which was subjected to the next step without further purification.

To a stirred solution of the crude product **24b**, 1,3-bis(tert-butoxycarbonyl)-2-methyl-2-thiopseudourea (48.7 mg, 0.168 mmol, 2.00 eq.) and mercury (II) chloride (45.7 mg, 0.168 mmol, 2.00 eq.) in dry dichloromethane (5.0 mL), Et_3N (42.0 mg, 0.42 mmol, 5.00 eq.) was added at room temperature under argon atmosphere. The reaction mixture was stirred at room temperature for 2 h and then was quenched with water (10 mL) and extracted with CH_2Cl_2 (3 × 10 mL). The combined organic layer was dried over anhydrous $MgSO_4$, filtered and concentrated under reduced pressure to give a residue, which was purified by flash chromatography on silica gel (petroleum ether/ethyl acetate, 1:1) to afford compound **25** (40.2 mg, 88% yield, for 2 steps) as a white foam.

Note: One Boc protection group was removed during purification of silica gel.

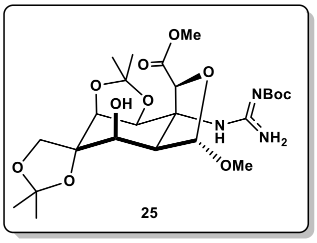

$\mathbf{R_f}$ = 0.2 (PE/EtOAc = 2:1)

$[\alpha]_D^{22}$ = +24.2 (*c* = 0.16, $CHCl_3$)

HRMS-ESI (m/z) calc. for $C_{24}H_{40}N_3O_{11}$ $[M+H]^+$: 546.2663; Found: 546.2650.

^{1}H NMR (400 MHz, $CDCl_3$, 25ºC) δ 11.35 (brs, 1H), 9.02 (brs, 1H), 5.46 (s, 1H), 5.28 (d, *J* = 5.8 Hz, 1H), 5.24 (s, 1H), 4.29 (dd, *J* = 16.4, 7.7 Hz, 2H), 4.19 (d, *J* = 9.6 Hz, 1H), 3.92 (dd, *J* = 12.0, 6.8 Hz, 1H), 3.80 (s, 3H), 3.48 (s, 3H), 3.35 (d, *J* = 12.3 Hz, 1H), 2.99 – 2.90 (m, 1H), 1.47 (s, 12H), 1.43 (s, 3H), 1.39 (s, 3H), 1.31 (s, 3H).

^{13}C NMR (101 MHz, $CDCl_3$, 25ºC) δ 168.44, 153.94, 152.21, 111.21, 109.59, 104.50, 83.07, 80.70, 79.47, 73.13, 68.49, 68.03, 62.48, 55.82, 54.21, 52.14, 29.69, 28.31, 28.07, 27.27, 25.29.

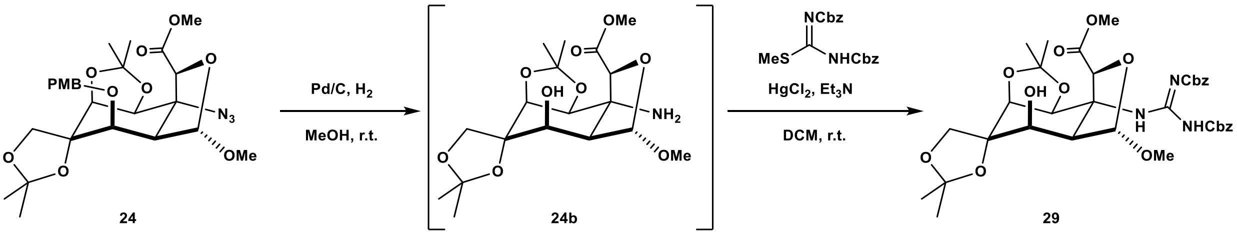

A suspension of Compound **24** (13.0 mg, 0.024 mmol, 1.00 eq.) and Pd-C (2.6 mg, 20 wt%) in MeOH (2.0 mL) was stirred at room temperature for 19 h under H_2 atmosphere (balloon). The mixture was passed through a Celite® pad, the filtrate was evaporated to give the crude product **24b**, which was subjected to the next step without further purification.

To a stirred solution of the crude product **24b**, 1,3-bis(benzyloxycarbonyl)-2-methyl-2-thiopseudourea (17.2 mg, 0.048 mmol, 2.00 eq.) and mercury (II)

chloride (13.0 mg, 0.048 mmol, 2.00 eq.) in dry dichloromethane (2.5 mL), Et_3N (12.1 mg, 0.12 mmol, 5.00 eq.) was added at room temperature under argon atmosphere. The reaction mixture was stirred at room temperature for 3 h and then was quenched with water (10 mL) and extracted with CH_2Cl_2 (3 × 10 mL). The combined organic layer was dried over anhydrous $MgSO_4$, filtered and concentrated under reduced pressure to give a residue, which was purified by flash chromatography on silica gel (petroleum ether/ethyl acetate, 2:1) to afford compound **29** (13.4 mg, 80% yield, for 2 steps) as a white foam.

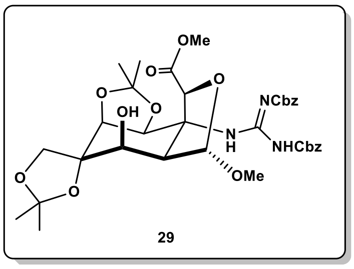

R_f = 0.65 (PE/EtOAc = 2:1)

$[\alpha]_D^{22}$ = +21.1 (*c* = 0.22, $CHCl_3$)

HRMS-ESI (m/z) calc. for $C_{35}H_{44}N_3O_{13}$ $[M+H]^+$: 714.2874; Found: 714.2863.

^{1}H NMR (400 MHz, $CDCl_3$, 25ºC) *δ* 11.64 (brs, 1H), 9.03 (brs, 1H), 7.41 – 7.28 (m, 10H), 5.45 (d, *J* = 2.8 Hz, 1H), 5.26 – 5.21 (m, 1H), 5.18 – 5.05 (m, 5H), 4.31 (d, *J* = 9.7 Hz, 1H), 4.22 – 4.16 (m, 2H), 3.93 (dd, *J* = 12.0, 6.7 Hz, 1H), 3.61 (s, 3H), 3.45 (s, 3H), 3.36 (d, *J* = 12.5 Hz, 1H), 2.98 (dd, *J* = 6.8, 2.9 Hz, 1H), 1.48 (s, 3H), 1.42 (s, 3H), 1.40 (s, 3H), 1.20 (s, 3H).

^{13}C NMR (101 MHz, $CDCl_3$, 25ºC) *δ* 168.07, 163.03, 154.15, 152.89, 136.58, 134.61, 128.80, 128.67, 128.40, 127.90, 127.75, 111.35, 109.80, 104.45, 80.58, 79.47, 72.96, 68.51, 68.14, 67.90, 67.01, 62.67, 55.81, 54.15, 52.01, 29.69, 27.22, 25.36, 25.29, 24.11.

Method A: One step synthesis of TTX

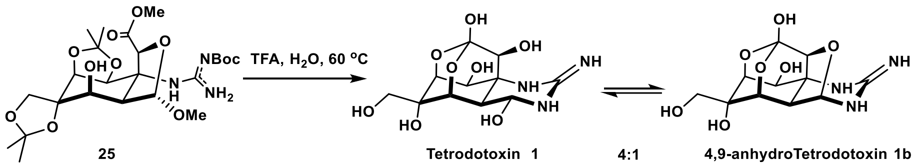

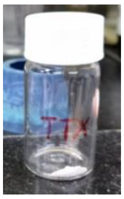

Compound **25** (40.0 mg, 0.073 mmol, 1.00 eq.) was dissolved in trifluoroacetic acid (1.0 mL) and water (1.0 mL) at room temperature under argon atmosphere, then the reaction mixture was heated to 60 °C and stirred at 60 °C for 24 h. The resulting mixture was concentrated in vacuo to give a residue, which was detected by ^{1}H NMR to give a mixture of tetrodotoxin and 4,9-anhydrotetrodotoxin (1:1). Then the mixture was re-dissolved in TFA-*d* (20.0 µL) and deuterium oxide (1.0 mL) and then stirred at room temperature for 5 days. The resulting mixture was concentrated in vacuo to give the residue, which was purified by HPLC (samples preparation and purity analysis were conducted on Waters HPLC (Column Atlanits® HILIC Silica, 5 µm, 4.6*150 mm and 19*150 mm) with 2998PDA and 3100MS detectors, mobile phase: H_2O (0.1% AcOH)/ Acetonitrile (0.1% AcOH) (65%-50% in 10min)). The residual solution was lyophilized and gave a mixture of tetrodotoxin **1** and 4,9-anhydrotetrodotoxin **1b** in a 4:1 ratio (detected by ^{1}H NMR) (15.2 mg, total 65% yield) as white solid.

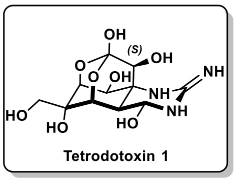

$[\alpha]_D^{25}$ = +1.8 (*c* = 0.06, 0.05 M AcOH)

HRMS-ESI (m/z) calc. for $C_{11}H_{18}N_3O_8$ $[M+H]^+$: 320.1094; Found: 320.1085.

^{1}H NMR (600 MHz, 5% CD_3CO_2D/D_2O, 25ºC) *δ* 5.49 (d, *J* = 9.4 Hz, 1H), 4.28 (brs, 1H), 4.24 (brs, 1H), 4.07 (brs, 1H), 4.04 (d, *J* = 12.6 Hz, 1H), 4.00 (d, *J* = 12.6 Hz, 1H), 3.95 (s, 1H), 2.34 (d, *J* = 9.4 Hz, 1H).

^{13}C NMR (151 MHz, 5% CD_3CO_2D/D_2O, 25ºC) *δ* 156.52, 110.76, 79.59, 75.06, 73.77, 72.72, 71.39, 70.80, 65.45, 59.65, 40.62.

Method B: Two steps synthesis of TTX

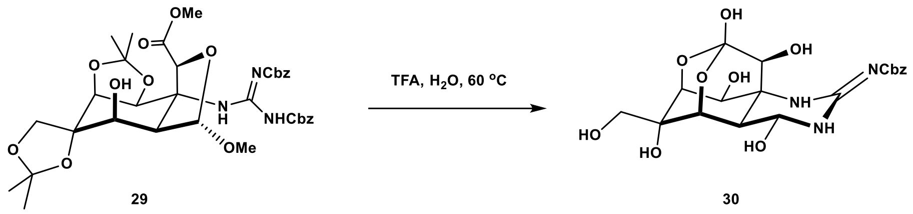

29 **30**

Compound **29** (13.0 mg, 0.018 mmol, 1.00 eq.) was dissolved in trifluoroacetic acid (500.0 µL) and water (500.0 µL) at room temperature under argon atmosphere. Then the reaction mixture was heated to 60 °C and stirred at 60 °C for 24 h. The resulting mixture was concentrated in vacuo to give a residue, which was purified by preparative TLC to give hemiaminal **30** (4.1 mg, 49.6% yield) as a white foam.

NOTE: Hemiaminal **30** and its anhydrous form could be separated by PTLC (DCM: MeOH = 4:1) following process described by Fukuyama.[4]

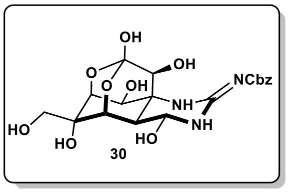

$\mathbf{R_f}$ = 0.15 (DCM/MeOH = 4:1)

$[\alpha]_D^{22}$ = -52.9 (*c* = 0.08, MeOH)

HRMS-ESI (m/z) calc. for $C_{19}H_{24}N_3O_{10}$ $[M+H]^+$: 454.1461; Found: 454.1455.

^{1}H NMR (400 MHz, CD_3OD, 25°C) *δ* 7.51 – 7.17 (m, 5H), 5.63 (d, *J* = 8.8 Hz, 1H), 5.18 (s, 2H), 4.19 (d, *J* = 14.8 Hz, 2H), 3.88 – 4.10 (m, 4H), 2.31 (d, *J* = 9.0 Hz, 1H).

^{13}C NMR (151 MHz, CD_3OD, 25°C) *δ* 169.65, 156.11, 137.49, 129.54, 129.31, 129.25, 109.77, 78.97, 74.80, 73.03, 71.91, 70.03, 68.61, 64.79, 58.81, 39.96.

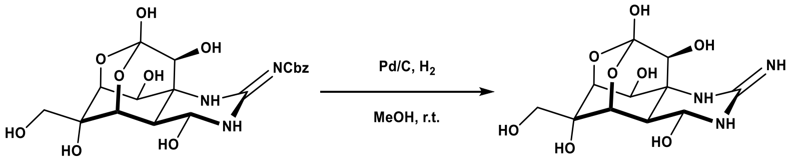

30 **Tetrodotoxin 1**

A suspension of Compound **30** (4.0 mg, 0.009 mmol, 1.00 eq.) and Pd-C (2.0 mg, 50 wt%) in MeOH (2.0 mL) was stirred at room temperature for 6 h under H_2 atmosphere (balloon). The mixture was passed through a Celite® pad, the filtrate was evaporated to give a residue, which was purified by HPLC (samples preparation and purity analysis were conducted on Waters HPLC (Column Atlanits® HILIC Silica, 5 µm, 4.6*150 mm and 19*150 mm) with 2998PDA and 3100MS detectors, mobile phase: H_2O (0.1% AcOH)/ Acetonitrile (0.1% AcOH) (65%-50% in 10 min)). The residual solution was lyophilized to give tetrodotoxin **1** as a white solid (2.5 mg, 91% yield).

Synthesis of 9-*epi*TTX

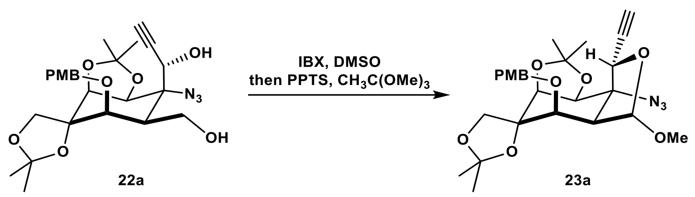

To a stirred solution of compound **22a** (50.0 mg, 0.10 mmol, 1.00 eq.) in DMSO (2.0 mL), IBX (29.0 mg, 0.10 mmol, 1.00 eq.) was added at room temperature under argon atmosphere. Then the reaction mixture was stirred at room temperature for 2 h. After the disappearance of **22a** on TLC with 4:1 petroleum ether/EtOAc, MeOH (2 mL), pyridinium *p*-toluenesulfonate (PPTS) (37.8 mg, 0.15 mmol, 1.50 eq.) and trimethyl orthoacetate (0.55 mL) was added into the above solution and then the solution was stirred at room temperature for 12 h. The mixture was quenched with sat. aq. $NaHCO_3$ (2 mL), extracted with CH_2Cl_2 (3 × 10 mL). The combined organic layer was dried over anhydrous $MgSO_4$, filtered and concentrated under reduced pressure to give a residue, which was purified by flash chromatography on silica gel (petroleum ether/ethyl acetate, 8:1) to afford compound **23a** (33.3 mg, 65% yield) as a white foam.

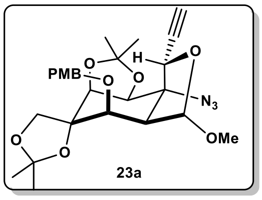

R_f = 0.35 (PE/EA = 4:1)

$[\alpha]_D^{22}$ = -4.5 (*c* = 0.108, $CHCl_3$)

HRMS-ESI (m/z) calc. for $C_{26}H_{33}N_3O_8Na$ $[M+Na]^+$: 538.2166; Found: 538.2150.

^{1}H NMR (400 MHz, $CDCl_3$, 25°C) *δ* 7.23 (d, *J* = 8.7 Hz, 2H), 6.87 (d, *J* = 8.7 Hz, 2H), 5.09 (d, *J* = 3.2 Hz, 1H), 4.99 (d, *J* = 2.2 Hz, 1H), 4.53 (dd, *J* = 30.7, 11.1 Hz, 2H), 4.39 (d, *J* = 7.7 Hz, 1H), 4.30 – 4.25 (m, 2H), 4.09 (d, *J* = 9.5 Hz, 1H), 3.80 (s, 3H), 3.79 (d, *J* = 0.9 Hz, 1H), 3.47 (s, 3H), 2.78 (dd, *J* = 5.9, 3.2 Hz, 1H), 2.68 (d, *J* = 2.2 Hz, 1H), 1.42 (s, 3H), 1.39 (s, 3H), 1.31 (s, 3H), 1.29 (s, 3H).

^{13}C NMR (101 MHz, $CDCl_3$, 25°C) *δ* 159.36, 129.39, 113.80, 110.23, 109.55, 106.48, 80.49, 78.71, 78.10, 76.47, 76.33, 76.00, 75.00, 74.64, 69.76, 68.46, 56.33, 55.25, 51.23, 29.69, 26.81, 26.60, 25.25, 23.97.

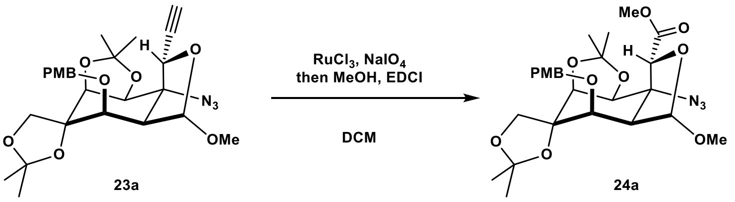

To a stirred solution of compound **23a** (20.0 mg, 0.04 mmol, 1.00 eq.), $NaIO_4$ (34.2 mg, 0.16 mmol, 4.00 eq.) in CCl_4 (0.7 mL), MeCN (0.7 mL) and H_2O (1.1 mL), $RuCl_3 \cdot xH_2O$ (1.7 mg, 0.008 mmol, 0.20 eq.) was added at room temperature under argon atmosphere. Then the reaction mixture was stirred at room temperature for 0.5 h. Then H_2O (2.0 mL) and CH_2Cl_2 (2.0 mL) was added into above solution. The residue was partitioned, the separated organic layer was used directly, EDCI·HCl (15.4 mg, 0.08 mmol, 2.00 eq.), DMAP (1.0 mg, 0.008 mmol, 0.20 eq.), MeOH (12.8 mg, 0.40 mmol, 10.00 eq.) was added into above organic solution at room temperature under argon atmosphere. The reaction mixture was stirred at room temperature for 2 h and then concentrated under reduced pressure to give a residue, which was purified by flash chromatography on silica gel (PE/EtOAc, 8/1 to 4/1) to afford compound **24a** (10.4 mg, 49% yield) as a white foam.

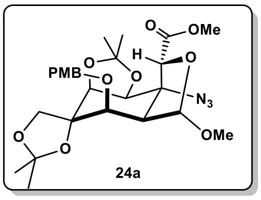

R_f = 0.35 (PE/EA = 4:1)

$[\alpha]_D^{22}$ = -10.8 (*c* = 0.042, $CHCl_3$)

HRMS-ESI (m/z) calc. for $C_{26}H_{35}N_3O_{10}Na$ $[M+Na]^+$: 572.2220; Found: 572.2214.

^{1}H NMR (400 MHz, $CDCl_3$, 25ºC) *δ* 7.23 (d, *J* = 8.6 Hz, 2H), 6.87 (d, *J* = 8.7 Hz, 2H), 5.14 (d, *J* = 6.1 Hz, 1H), 4.87 (s, 1H), 4.50 (q, *J* = 11.0 Hz, 2H), 4.37 (d, *J* = 7.4 Hz, 1H), 4.26 – 4.17 (m, 2H), 4.01 (d, *J* = 9.6 Hz, 1H), 3.87 – 3.84 (m, 1H), 3.84 (s, 3H), 3.80 (s, 3H), 3.58 (s, 3H), 2.72 (t, *J* = 6.4 Hz, 1H), 1.37 (s, 6H), 1.25 (s, 6H).

^{13}C NMR (101 MHz, $CDCl_3$, 25ºC) *δ* 168.61, 159.43, 129.88, 129.48, 113.75, 110.61, 109.11, 106.30, 80.97, 79.99, 75.26, 75.14, 75.07, 68.67, 68.29, 57.41, 55.25, 52.28, 49.03, 29.70, 26.94, 26.41, 24.83, 24.17.

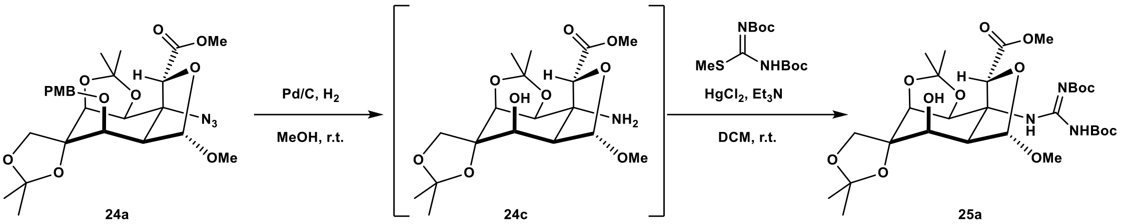

A suspension of compound **24a** (8.0 mg, 0.015 mmol, 1.00 eq.) and Pd/C (1.6 mg, 20 wt%) in MeOH (1.0 mL) was stirred at room temperature for 24 h under H_2 atmosphere (balloon). The mixture was passed through a Celite® pad. The filtrate was evaporated to give the crude product **24c**, which was subjected to the next step without further purification.

To a stirred solution of the crude product **24c**, 1,3-bis(tert-butoxycarbonyl)-2-methyl-2-thiopseudo urea (8.7 mg, 0.030 mmol, 2.00 eq.) and mercury (II) chloride (8.2 mg, 0.030 mmol, 2.00 eq.) in dry dichloromethane (1.5 mL), Et_3N (7.6 mg, 0.075 mmol, 5.00 eq.) was added at room temperature under argon atmosphere. The reaction mixture was stirred at room temperature for 2 h and then was quenched with water (10.0 mL) and extracted with CH_2Cl_2 (3 × 10 mL). The combined organic layer was dried over anhydrous $MgSO_4$, filtered and concentrated under reduced pressure to give a residue, which was purified by flash chromatography on silica gel (PE/EtOAc, 2:1) to afford compound **25a** (7.5 mg, 80% yield, for 2 steps) as a white foam.

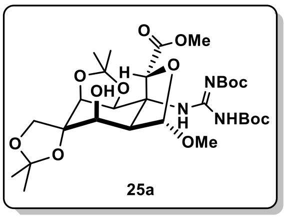

R_f = 0. 4 (PE/EtOAc = 2:1)

$[\alpha]_D^{22}$ = +13.6 (*c* = 0.120, $CHCl_3$)

HRMS-ESI (m/z) calc. for $C_{35}H_{44}N_3O_{13}$ $[M+H]^+$: 646.3187; Found: 646.3174.

^{1}H NMR (400 MHz, $CDCl_3$, 25ºC) *δ* 11.08 (brs, 1H), 9.14 (brs, 1H), 5.44 (d, *J* = 2.1 Hz, 1H), 5.27 (s, 1H), 5.13 (d, *J* = 6.8 Hz, 1H), 4.35 (d, *J* = 9.6 Hz, 1H), 4.24 (d, *J* = 6.5 Hz, 1H), 4.15 (d, *J* = 9.6 Hz, 1H), 3.97 (dd, *J* = 11.7, 7.0 Hz, 1H), 3.65 (s, 3H), 3.32 (s, 3H), 3.10 (d, *J* = 5.0 Hz, 1H), 3.04 (d, *J* = 12.0 Hz, 1H), 1.50 – 1.40 (m, 24H), 1.37 (s, 3H), 1.31 (s, 3H).

^{13}C NMR (101 MHz, $CDCl_3$, 25ºC) *δ* 169.12, 162.69, 154.29, 151.96, 111.28, 109.32, 106.71, 82.62, 82.15, 78.78, 78.47, 77.10, 74.03, 68.89, 68.03, 61.79, 55.15, 54.23, 51.54, 28.26, 28.07, 27.24, 25.46, 25.37, 23.67.

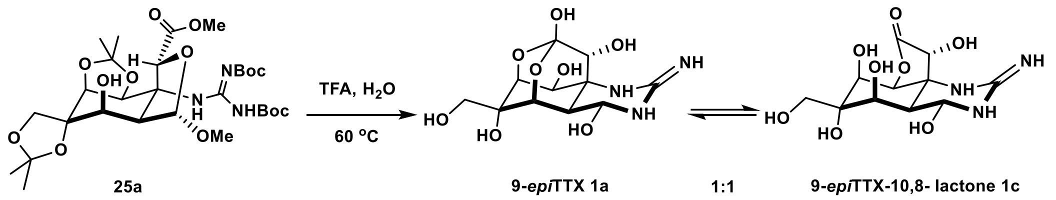

25a **9-*epi*TTX 1a** **9-*epi*TTX-10,8- lactone 1c**

Compound **25a** (7.0 mg, 0.011 mmol, 1.00 eq.) was dissolved in trifluoroacetic acid (500 µL) and water (500 µL) at room temperature under argon atmosphere. Then the reaction mixture was heated to 60 °C and stirred at 60 °C for 12 h. The resulting mixture was concentrated in vacuo to give a residue, which was purified by HPLC (samples preparation and purity analysis were conducted on Waters HPLC (Column Atlanits® HILIC Silica, 5 µm, 4.6*150 mm and 19*150 mm) with 2998PDA and 3100MS detectors, mobile phase: H_2O (0.1% AcOH)/ Acetonitrile (0.1% AcOH) (65%-50% in 10 min)). The residual solution was lyophilized to give 9-*epi*TTX-hemilactal **1a** and 9-*epi*TTX-10,8-lactone **1c** (1:1, detected by ^{1}H NMR) as a white solid (1.9 mg, 55% yield).

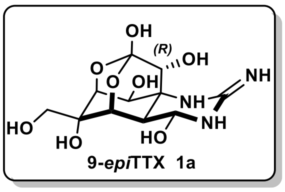

HRMS-ESI (m/z) calc. for $C_{11}H_{18}N_3O_8$ $[M+H]^+$: 320.1094; Found: 320.1086.

9-*epi*TTX(hemilactal) ^{1}H NMR (400 MHz, 5% CD_3CO_2D/D_2O)

δ 5.24 (d, *J* = 9.2 Hz, 2H), 4.24 (s, 1H), 4.22 (s, 1H), 4.15 (brs, 1H), 4.05 (d, *J* = 11.6 Hz, 1H), 3.99 (d, *J* = 11.6 Hz, 1H), 3.85 (s, 1H), 2.45 (d, *J* = 9.2 Hz, 1H).

9-*epi*TTX(10,8-lactone) ^{1}H NMR (400 MHz, 5% CD_3CO_2D/D_2O)

δ 5.44 (s, 1H), 5.34 (d, *J* = 10.4 Hz, 1H), 4.18 (d, *J* = 5.2 Hz, 1H), 4.08 (brs, 1H), 3.98 (d, *J* = 12.0 Hz, 1H), 3.85 (d, *J* = 12.0 Hz, 1H), 2.53 (dd, *J* = 10.2, 2.2 Hz, 1H).

9-*epi*TTX (hemilactal and 10,8-lactone) ^{13}C NMR (151 MHz, 5% CD_3CO_2D/D_2O) *δ* 180.03, 156.80, 155.14, 110.74, 81.06, 79.64, 76.78, 75.33, 74.25, 73.68, 73.27, 72.99, 71.03, 70.14, 69.42, 68.50, 66.00, 65.30, 63.40, 56.18, 43.90, 43.34.

2. Biological Evaluation

Culture of primary hippocampal neurons[5,6,7].

C57BL/6J mice strain were maintained in an animal facility with 12h light/12h dark cycles, temperature (22-24 °C), humidity (40-60%) at the National Institute of Biological Sciences, Beijing. PO pups was used for dissociating primary hippocampal neuron. Sex was not considered in the study design because there was no sex correlation involved in this experiment.

Hippocampi were isolated from P0 wildtype mice of either sex (C57BL/6J) and kept in ice-cold Hank's balanced salt solution (Sigma) and incubated with papain (10 U/ml in 1 mM $CaCl_2$ and 0.5 mM EGTA) at 37 ℃ for 20 min. Cells were dissociated by 1 mL pipette. Dissociated cells were plated on Matrigel-coated circular glass coverslips (12 mm diameter) in 24-well dishes and cultured for 14–16 days in vitro (DIV) in 1ml of MEM (Invitrogen) supplemented with B27 (Invitrogen), glucose, transferrin, fetal bovine serum, and AraC (Sigma).

Animal experimentation: Animal care and use followed the institutional guidelines of the National Institute of Biological Sciences (NIBS), Beijing (Approval ID: NIBSLuoM15C), and the Regulations for the Administration of Affairs Concerning Experimental Animals of China.

Electrophysiological recording of cultured hippocampal neurons.

The bath solution contained the following (in mM): 140 NaCl, 5 KCl, 2 $MgCl_2$, 2 $CaCl_2$, 10 HEPES, and 10 glucose, adjusted to pH 7.4 with NaOH. The whole-cell intracellular pipette solution contained the following (in mM): 126 $CsMeSO_3$, 10 HEPES, 1 EGTA, 0.1 $CaCl_2$, 3 GTP-Na3, 4 ATP-Mg, 8 Na2-phosphocreatine, adjusted to pH 7.4 with CsOH. The current signals were recorded with MultiClamp 700B and Clampex 10 data acquisition software (Molecular Devices). The resistance of pipettes varied between 4.0–7.0 MΩ. Recordings with series resistances of > 15 MΩ were rejected. Neurons were clamp at -70 mV in presence of TTX (S) or TTX (C). Sodium currents are evoked by a 50-ms width ramp voltage from -70 mV to 10 mV. Leakage currents of > 400 pA were rejected. Data were sampled at 10 kHz. The data were analyzed using Clampfit 10.5 (software) and Prism 6.02 (GraphPad Software). Sodium currents amplitudes were analyzed after normalized to the sodium current amplitude of 1 μM TTX Compounds group.

Voltage-gated sodium channel assay

HEK-293 cells stably expressing Na_v 1.5 (human) or Na_v 1.7 (human) were donated by Fan Zhang lab in Hebei Medical University and cultured in Dulbecco's Modified Eagle Medium (DMEM, Gibco) containing 4.5 mg/ml glucose, 10% fetal bovine serum (FBS, Gibco), 100 U/ml penicillin, 100 μg/mL streptomycin and incubated at 37 °C with 5% CO_2. When cell confluency reached 70%, the cells were treated with 0.05% trypsin (Gibco) and put on poly-D-lysine (Sigma)-coated 12 mm cover slips for whole cell electrophysiological characterization.

Sodium currents signals were recorded using MultiClamp 700B, Clampex 10 data acquisition software (Molecular Devices), and glass micropipettes (4-7 MΩ) in stably expressing Na_v 1.5 (human) or Na_v 1.7 (human) HEK-293 cells. The data were analyzed using Clampfit 10.5 (software) and Prism 6.02 (GraphPad Software). For recording the voltage-dependent currents, the electrodes were filled with the internal solution composed of (in mM) 40 CsCl, 10 NaCl, 10 EGTA, 105 CsF, 10 HEPES, pH 7.3 with CsOH. The extracellular solution was composed of (in mM) 130 NaCl, 4 KCl, 1 $MgCl_2$, 1.5 $CaCl_2$, 5 D-Glucose monohydrate, 5 HEPES, pH=7.4 with NaOH. The voltage dependence of ion current was elicited using a protocol consisting of steps from a holding potential of -80 mV to voltages ranging from -80 to 80 mV for 500 ms in 10 mV increment. The current density is obtained by dividing the current amplitude by the membrane capacitance and plotted against the voltage. Data were sampled at 10 kHz. Recordings with series resistances of > 15 MΩ or leakage currents of > 400 pA were rejected. The function (The formula: *xb*=IC50*10^((1/Hillslope)*log(2^(1/*s*) - 1)); *f1* = min+(max-min)/(1 + (*xb*/*x*)^Hillslope)^*s*; *f* = if(*x*<=0, if(Hillslope>0,min,max), *f1*)) in SigmaPlot 12.0 software were used for curve fitting and calculation of IC50.

3. NMR spectra and HPLC spectra

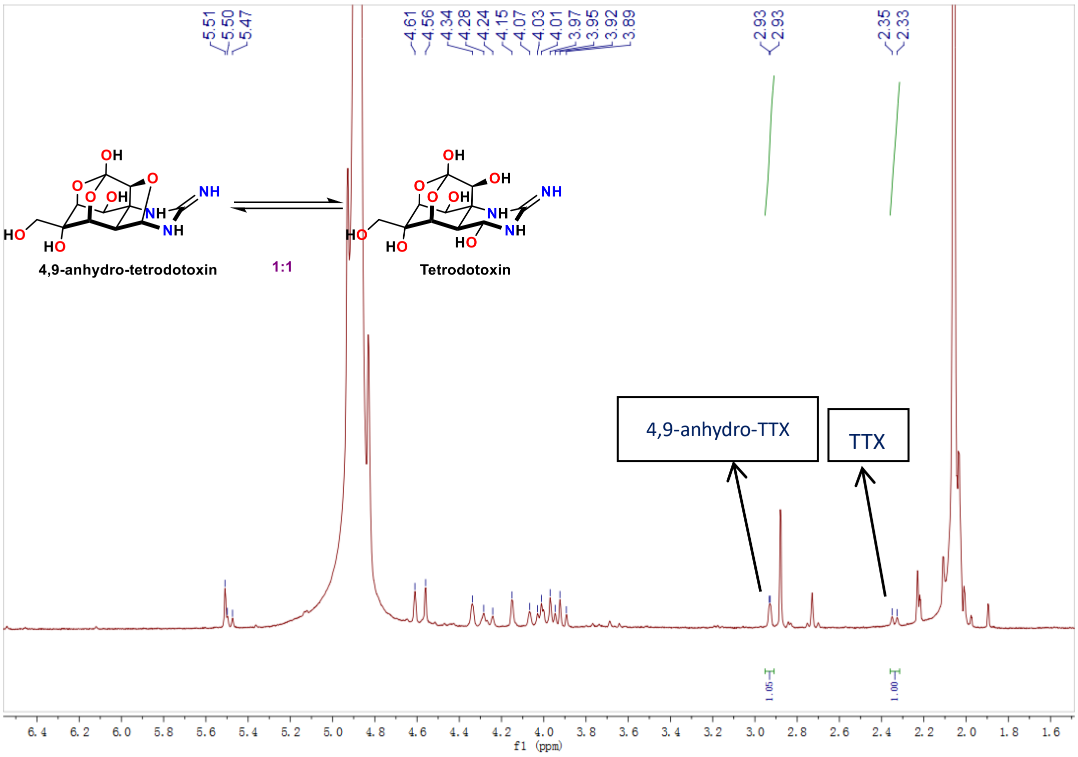

Supplementary Figure 6. ^{1}H NMR spectra for Synthetic Tetrodotoxin and 4,9-anhydrotetrodotoxin (Method A), (5% CD_3CO_2D/D_2O, 400 MHz,25°C).The ratio of Tetrodotoxin and 4,9-anhydrotetrodotoxin is 1:1.

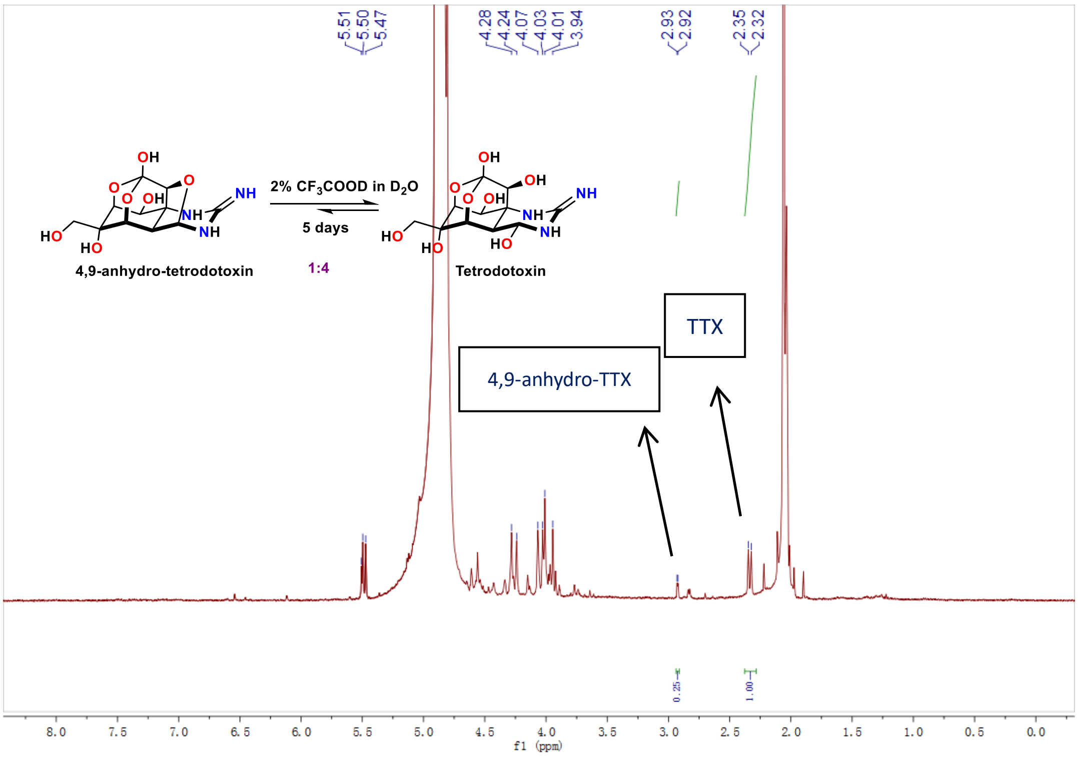

Supplementary Figure 7. ^{1}H NMR Spectra for Synthetic Tetrodotoxin and 4,9-anhydrotetrodotoxin (Method A) after stirring in d-TFA for 5 days (5%

CD3CO2D/D2O, 400 MHz, 25ºC). The ratio of Tetrodotoxin and 4,9-anhydrotetrodotoxin is 4:1.

^{1}H NMR (400 MHz, $CDCl_3$, 25ºC) of compound **10**

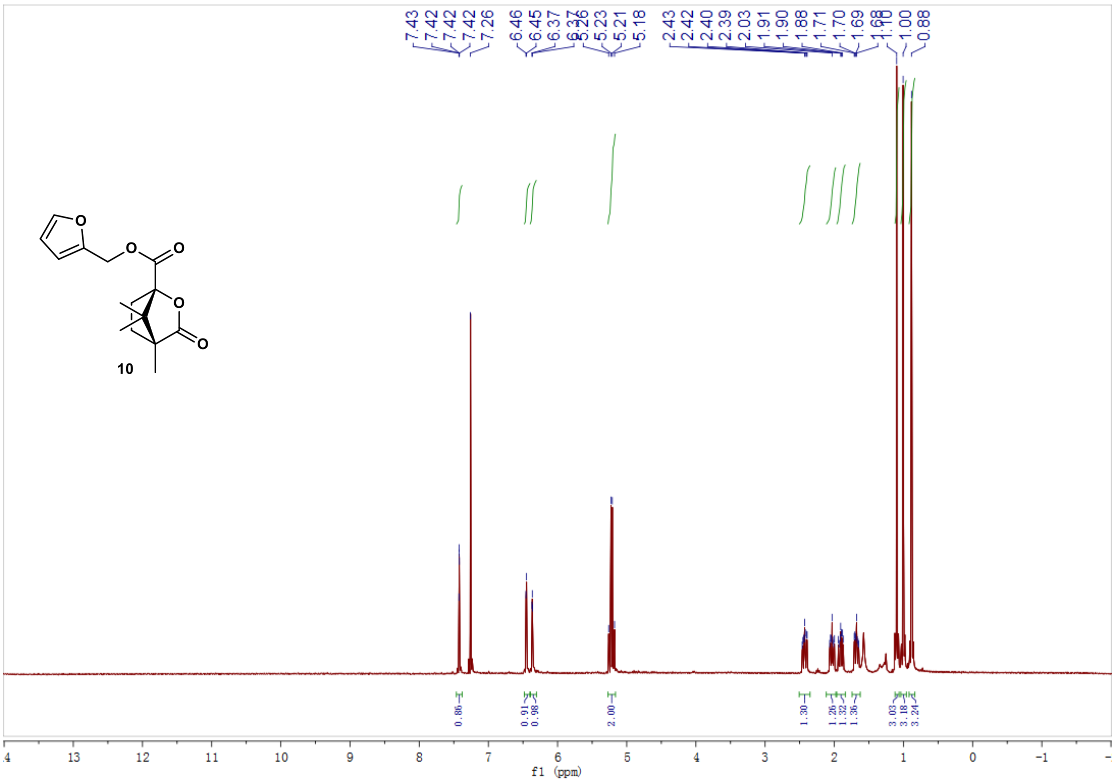

^{13}C NMR (101 MHz, $CDCl_3$, 25ºC) of compound **10**

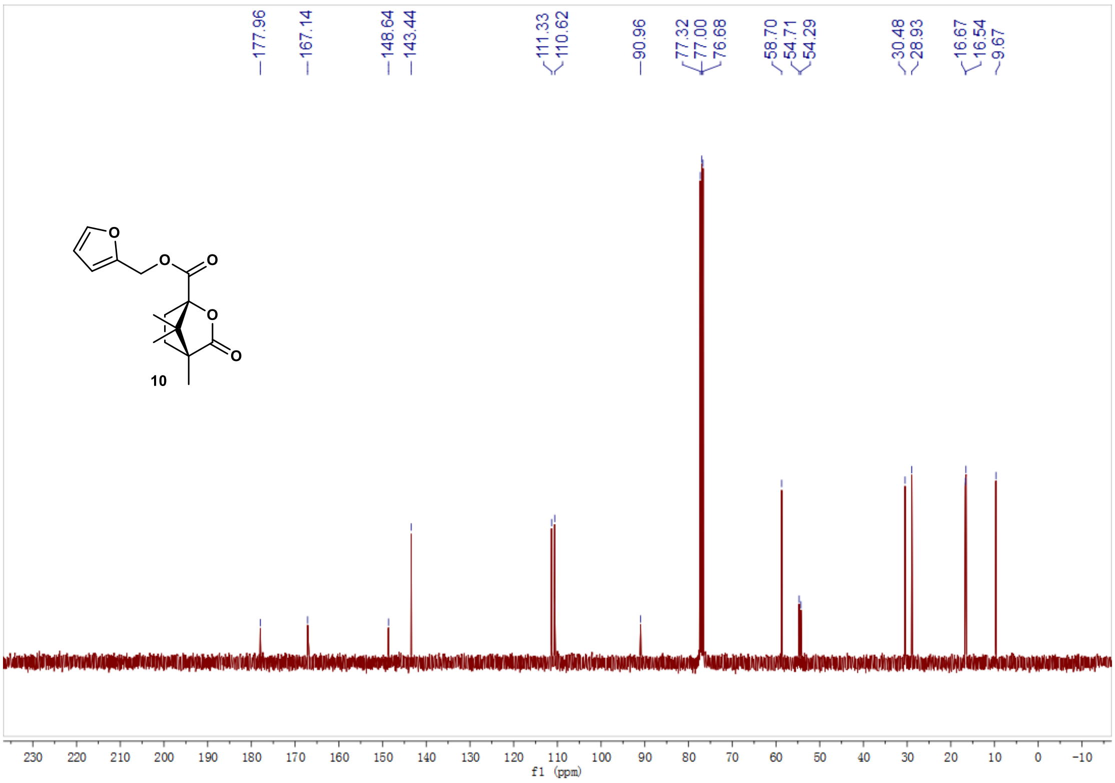

Supplementary Figure 8. NMR spectra of compound 10

^{1}H NMR (400 MHz, $CDCl_3$, 25°C) of compound **11**

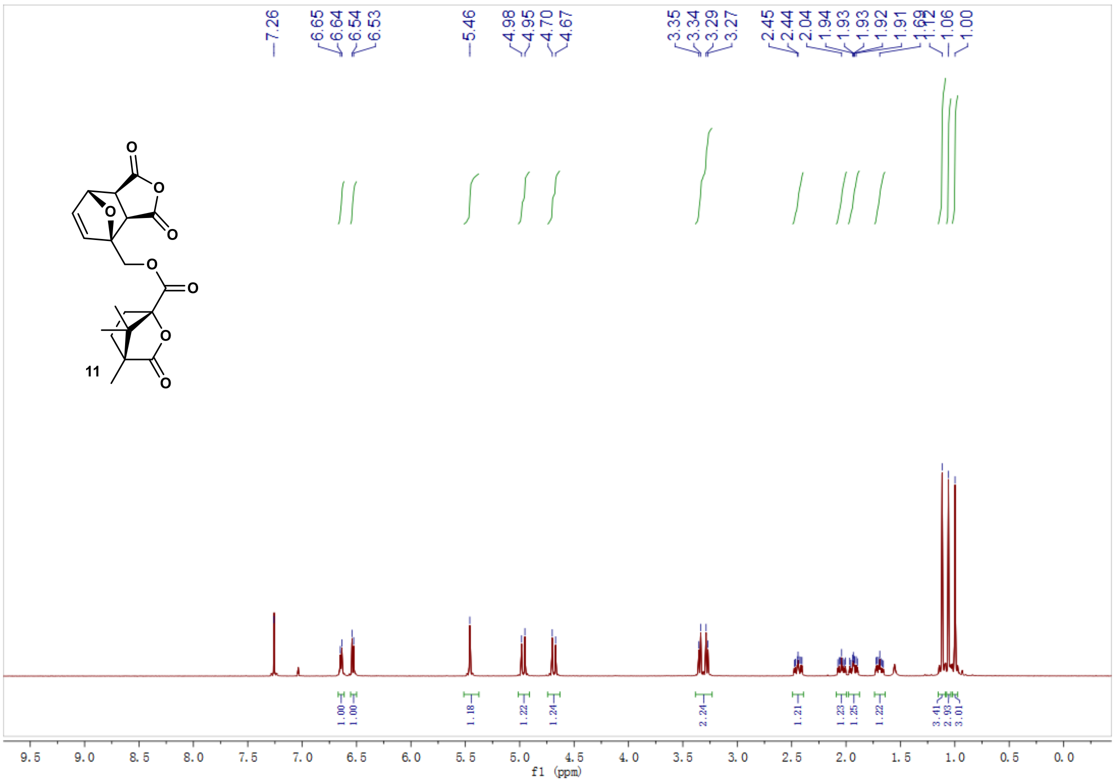

^{13}C NMR (101 MHz, $CDCl_3$, 25°C) of compound **11**

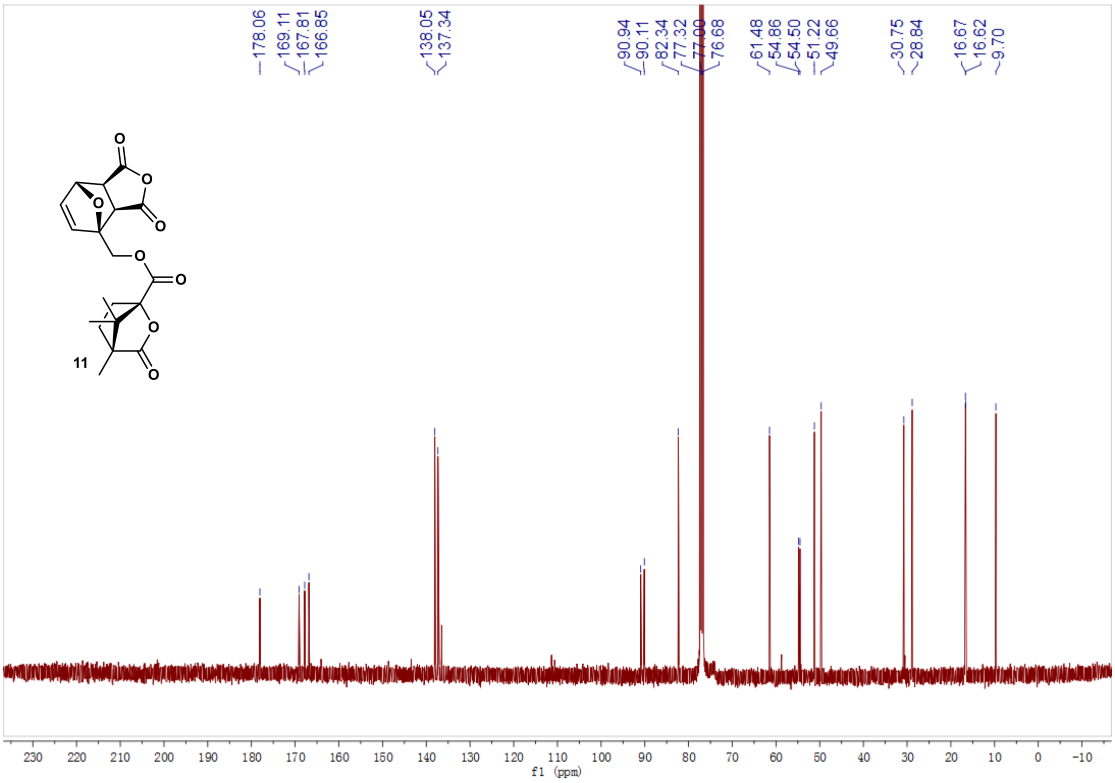

Supplementary Figure 9. NMR spectra of compound 11

^{1}H NMR (400 MHz, $CDCl_3$, 25°C) of compound **12**

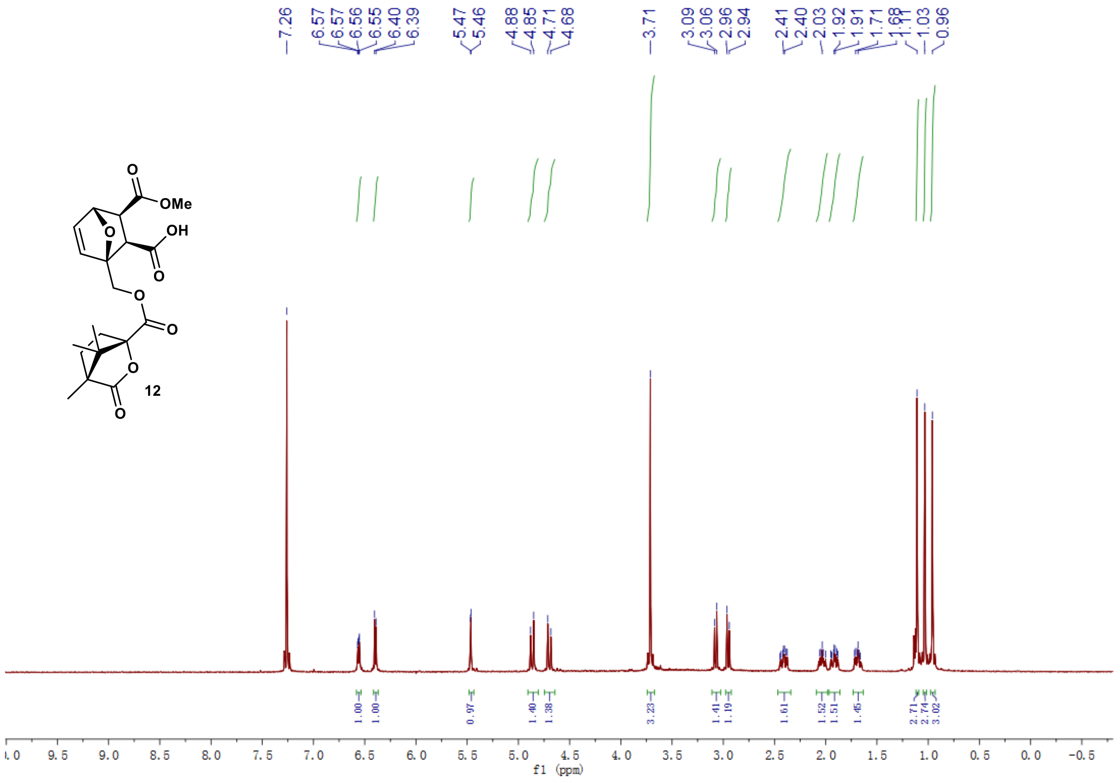

^{13}C NMR (101 MHz, $CDCl_3$, 25°C) of compound **12**

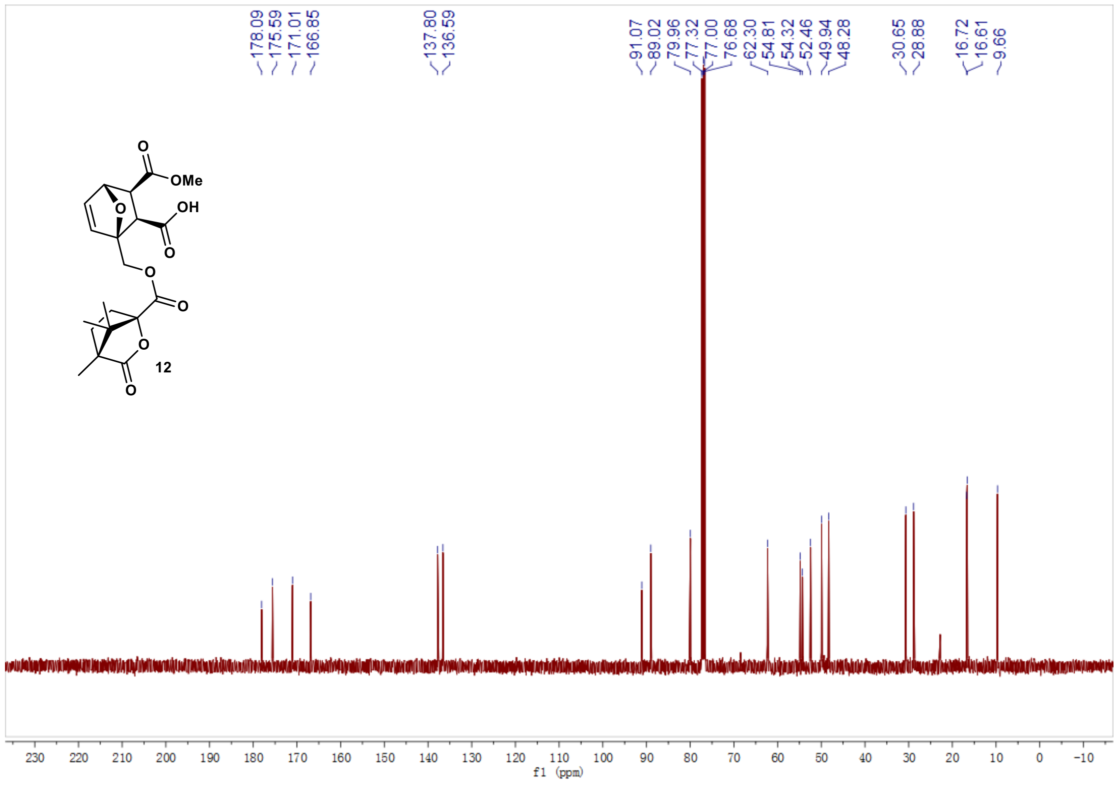

Supplementary Figure 10. NMR spectra of compound 12

^{1}H NMR (400 MHz, $CDCl_3$, 25°C) of compound **13**

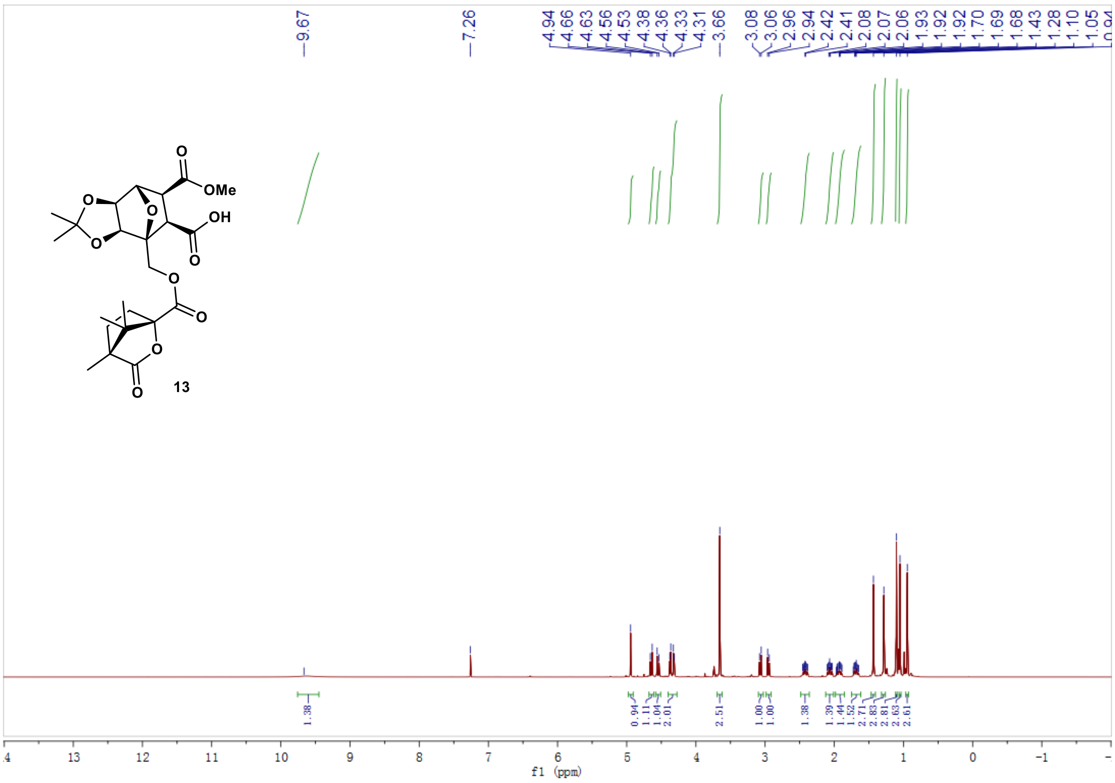

^{13}C NMR (101 MHz, $CDCl_3$, 25°C) of compound **13**

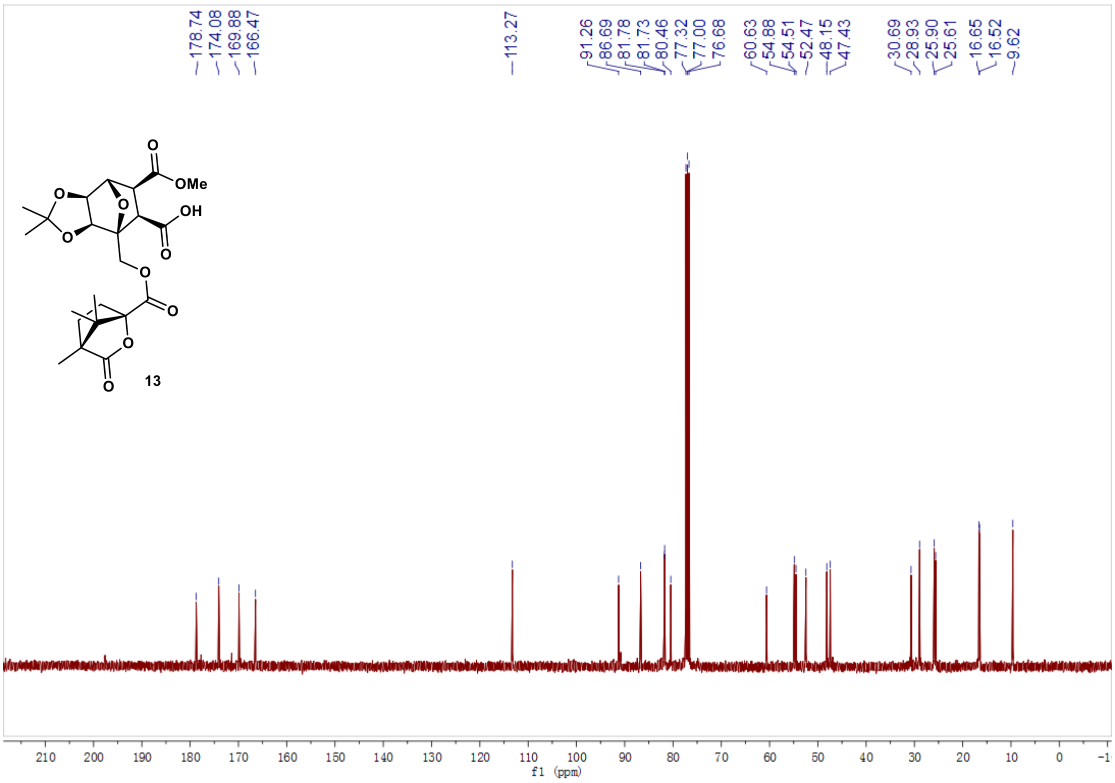

Supplementary Figure 11. NMR spectra of compound 13

^{1}H NMR (400 MHz, $CDCl_3$, 25°C) of compound **14**

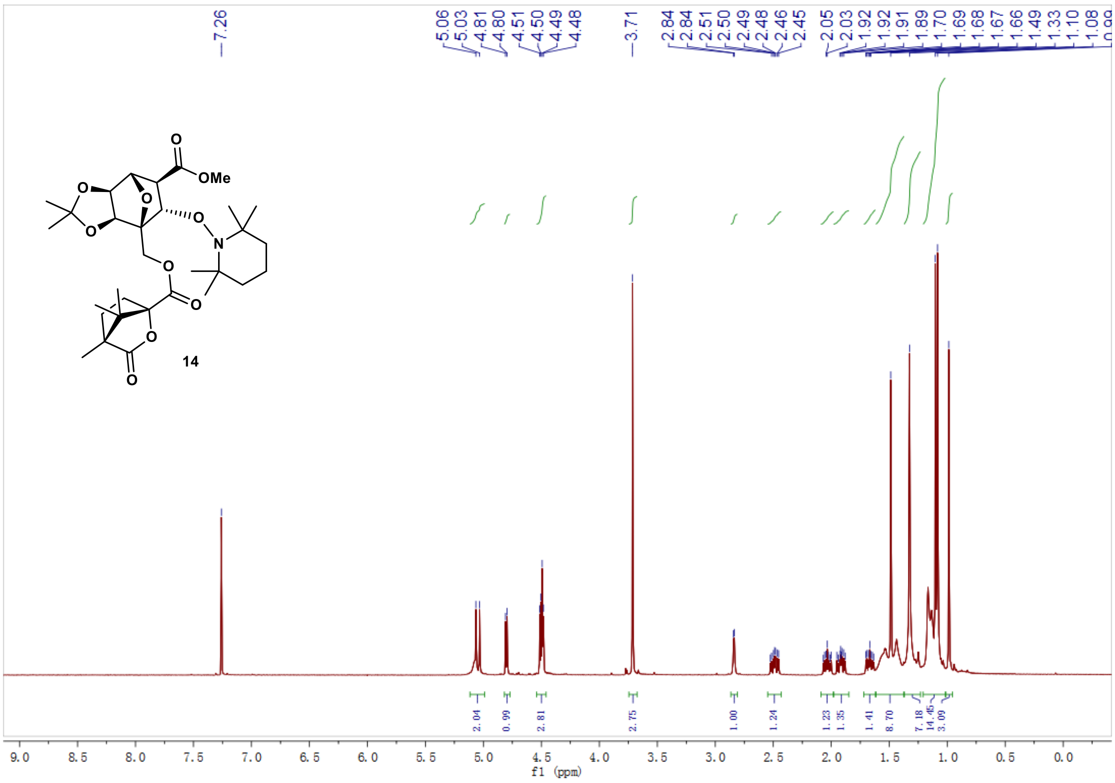

^{13}C NMR (101 MHz, $CDCl_3$, 25°C) of compound **14**

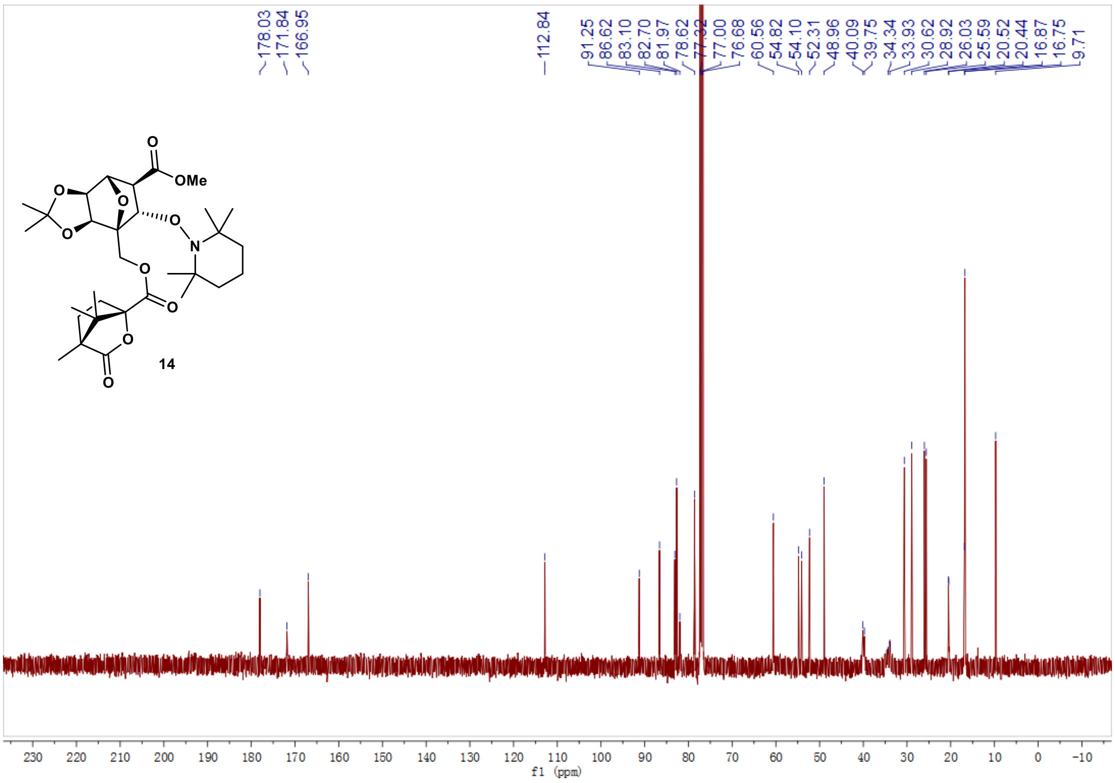

Supplementary Figure 12. NMR spectra of compound 14

^{1}H NMR (400 MHz, CDCl3, 25°C) of compound **14a**

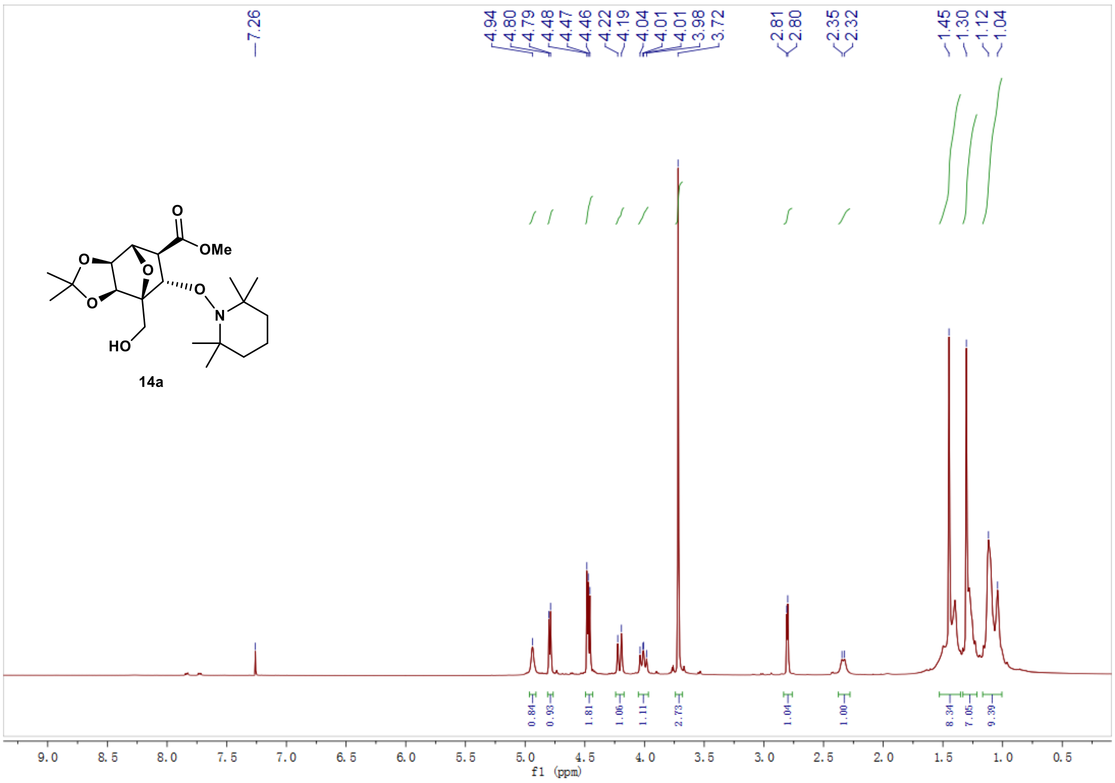

^{13}C NMR (101 MHz, CDCl3, 25°C) of compound **14a**

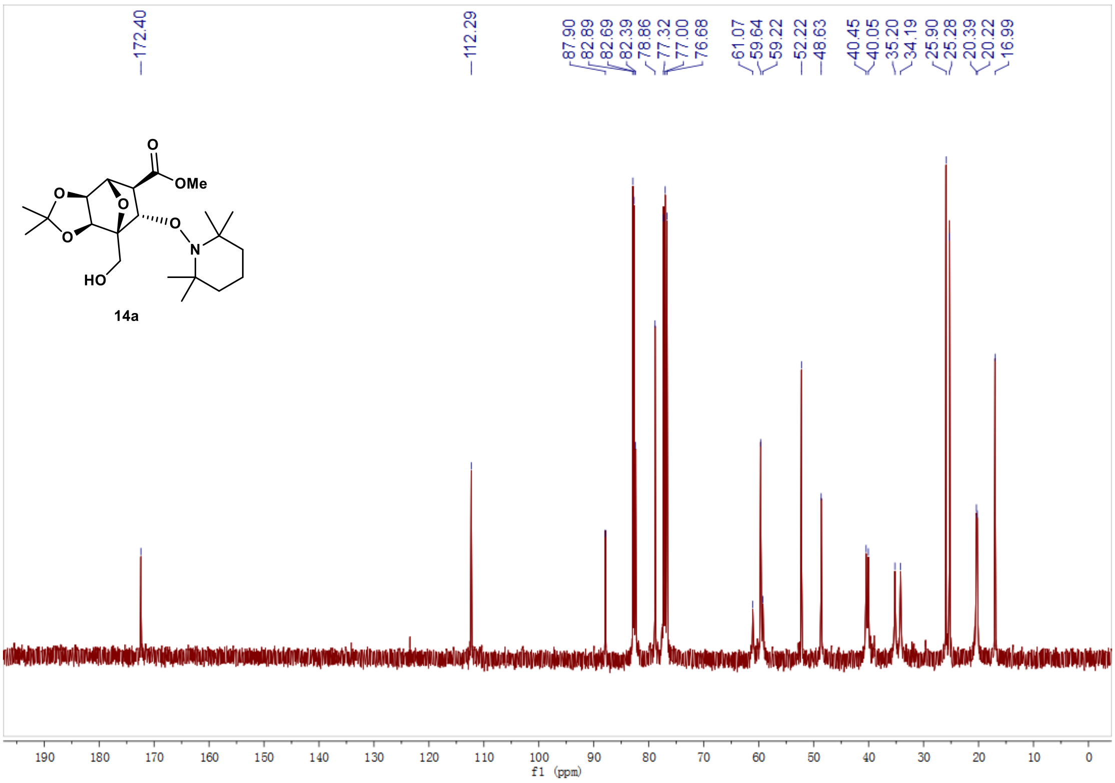

Supplementary Figure 13. NMR spectra of compound 14a

^{1}H NMR (400 MHz, $CDCl_3$, 25^oC) of compound **15**

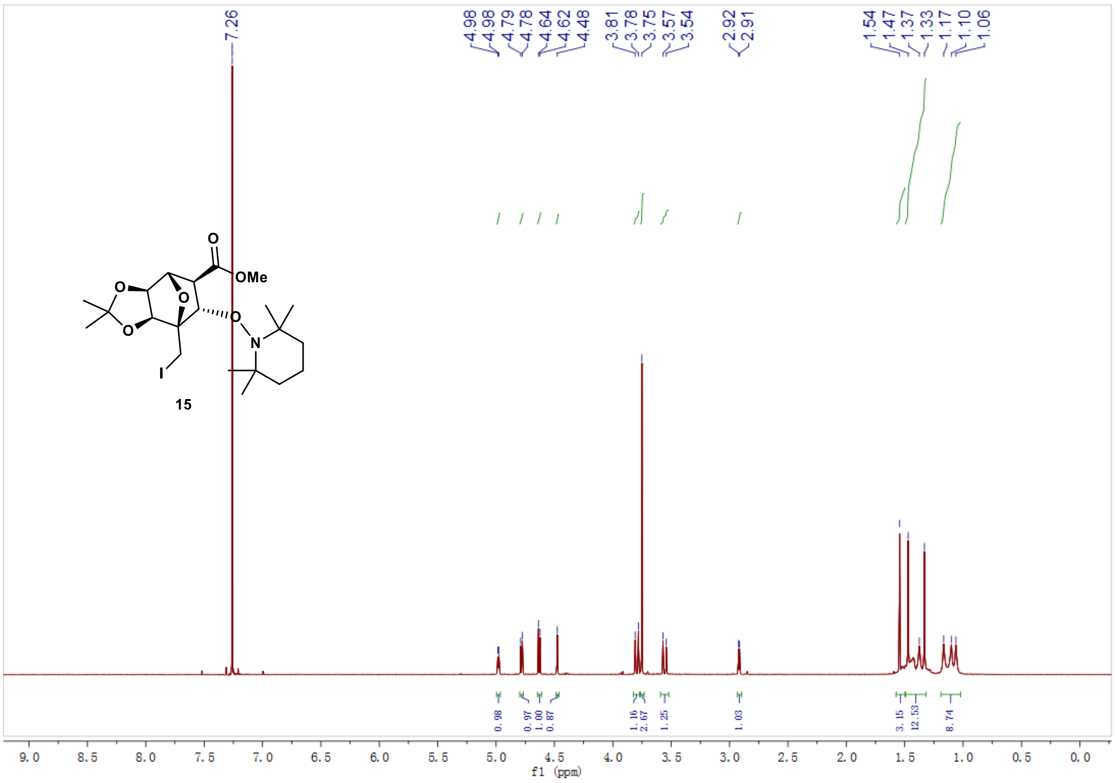

^{13}C NMR (101 MHz, $CDCl_3$, 25^oC) of compound **15**

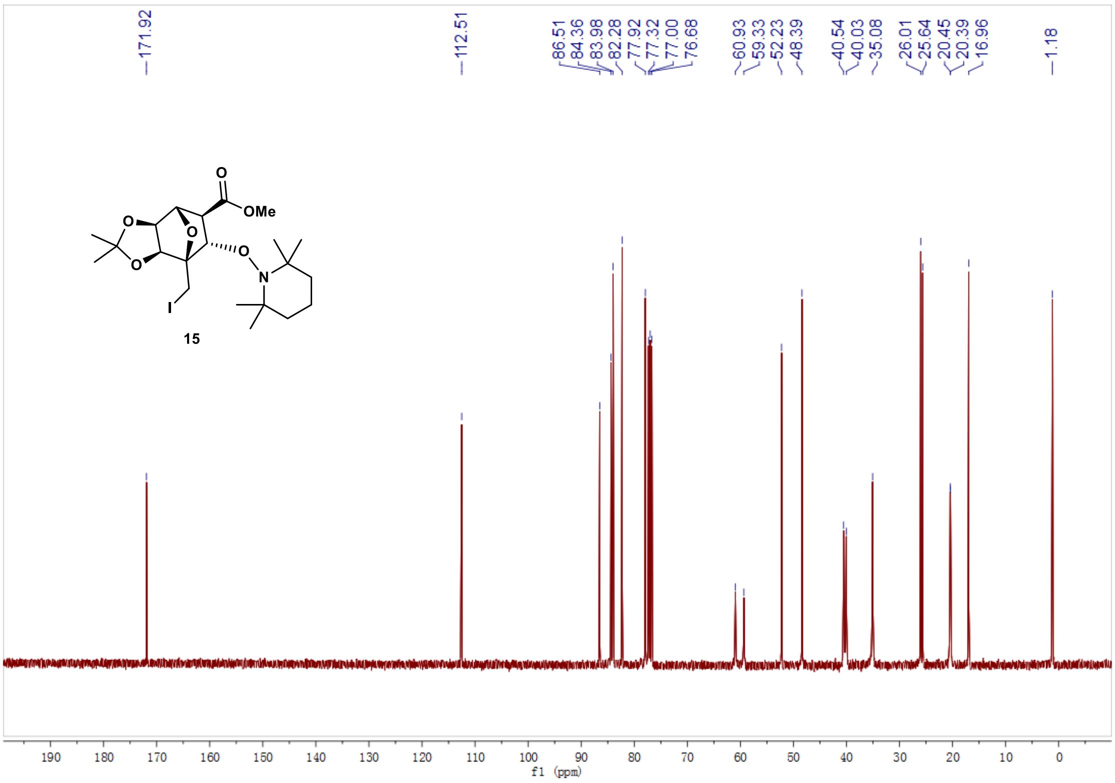

Supplementary Figure 14. NMR spectra of compound 15

^{1}H NMR (400 MHz, $CDCl_3$, 25°C) of compound **16**

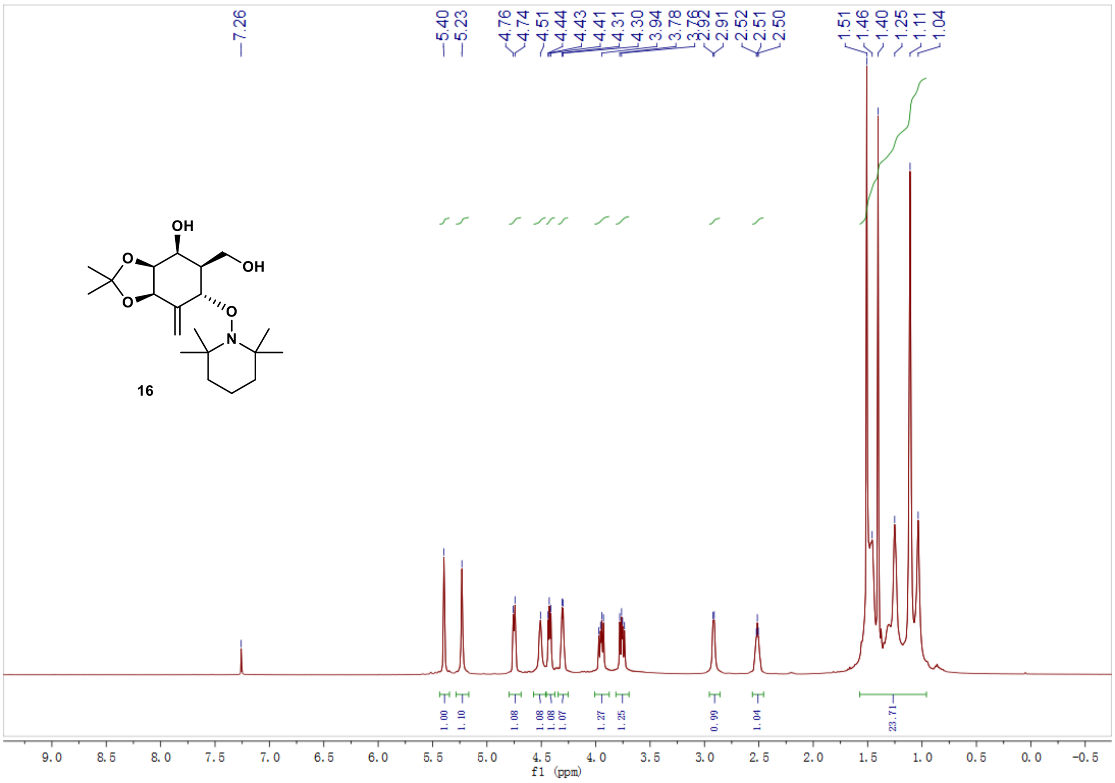

^{13}C NMR (101 MHz, $CDCl_3$, 25°C) of compound **16**

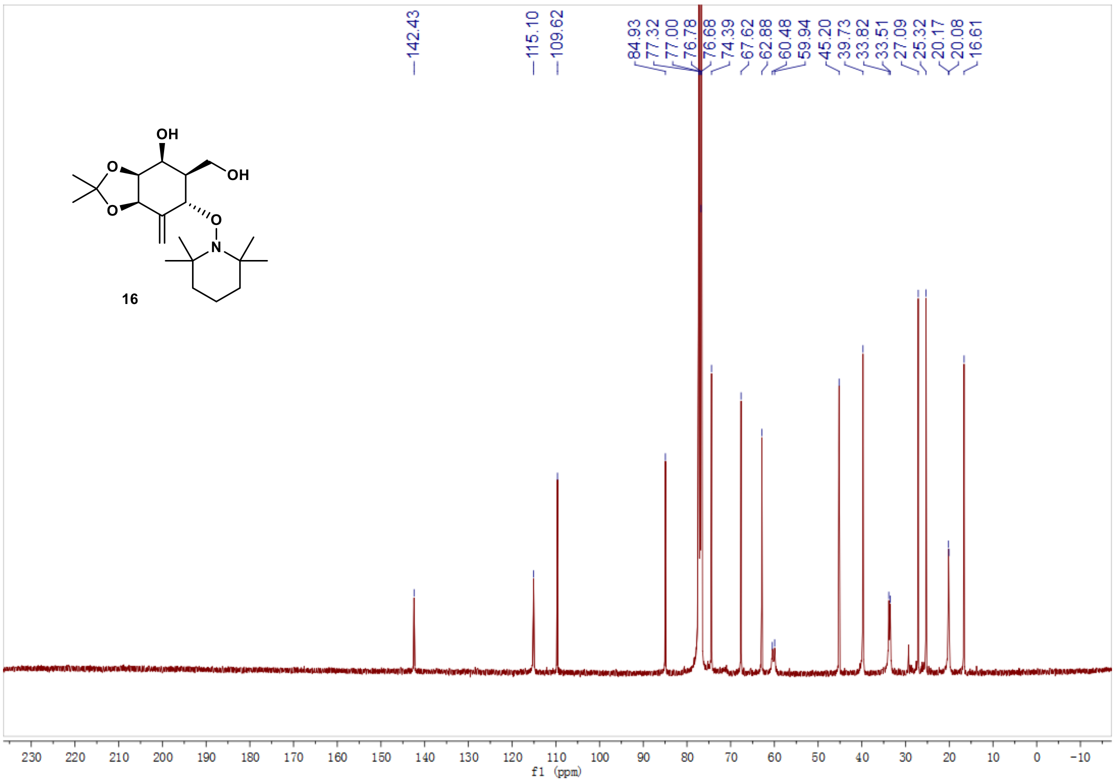

Supplementary Figure 15. NMR spectra of compound 16

^{1}H NMR (400 MHz, $CDCl_3$, 25°C) of compound **17a**

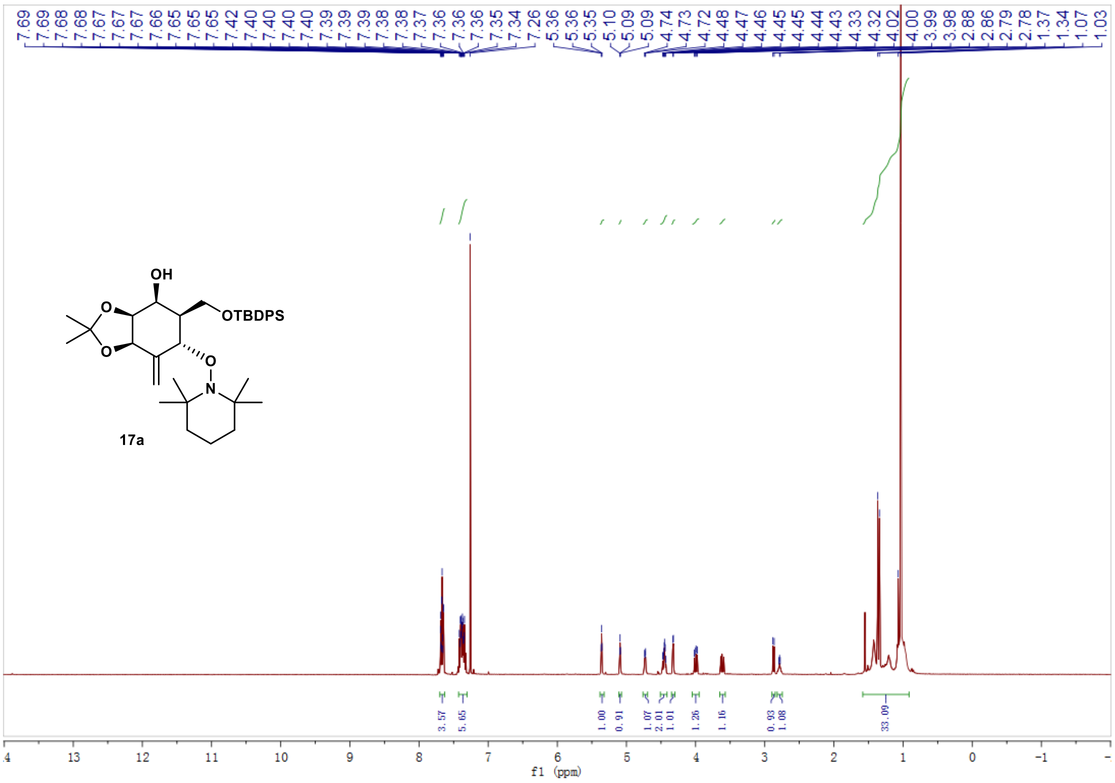

^{13}C NMR (101 MHz, $CDCl_3$, 25°C) of compound **17a**

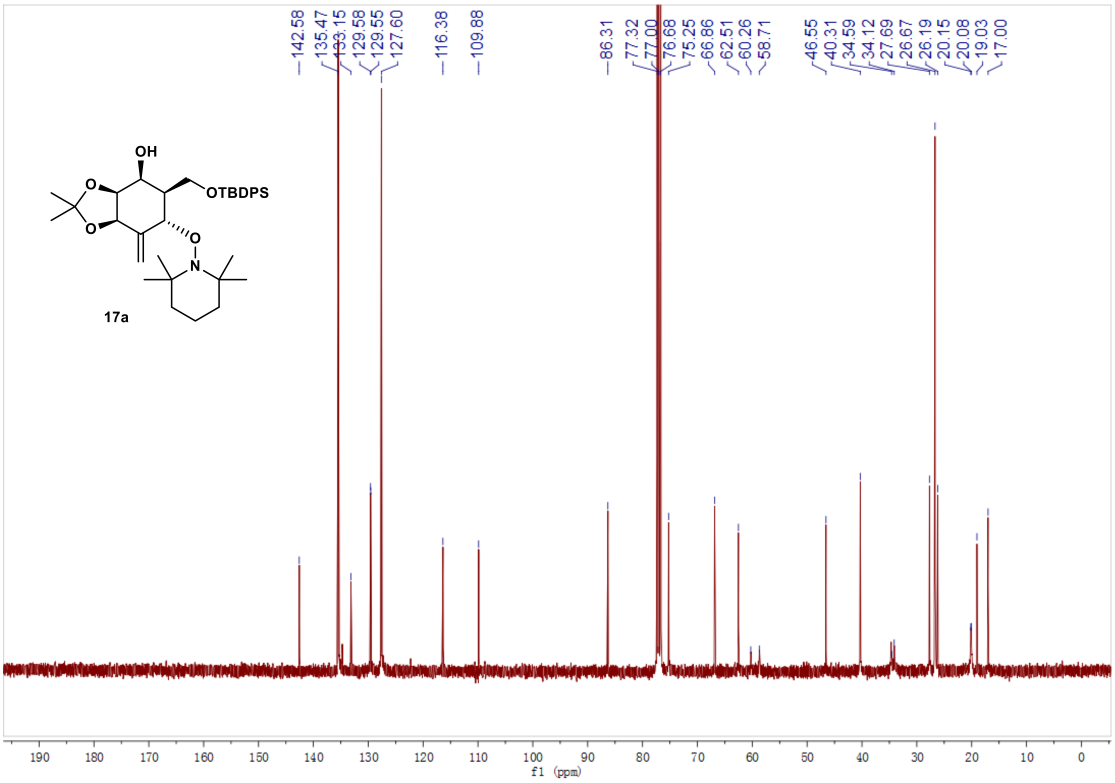

Supplementary Figure 16. NMR spectra of compound 17a

^{1}H NMR (400 MHz, $CDCl_3$, 25°C) of compound **17**

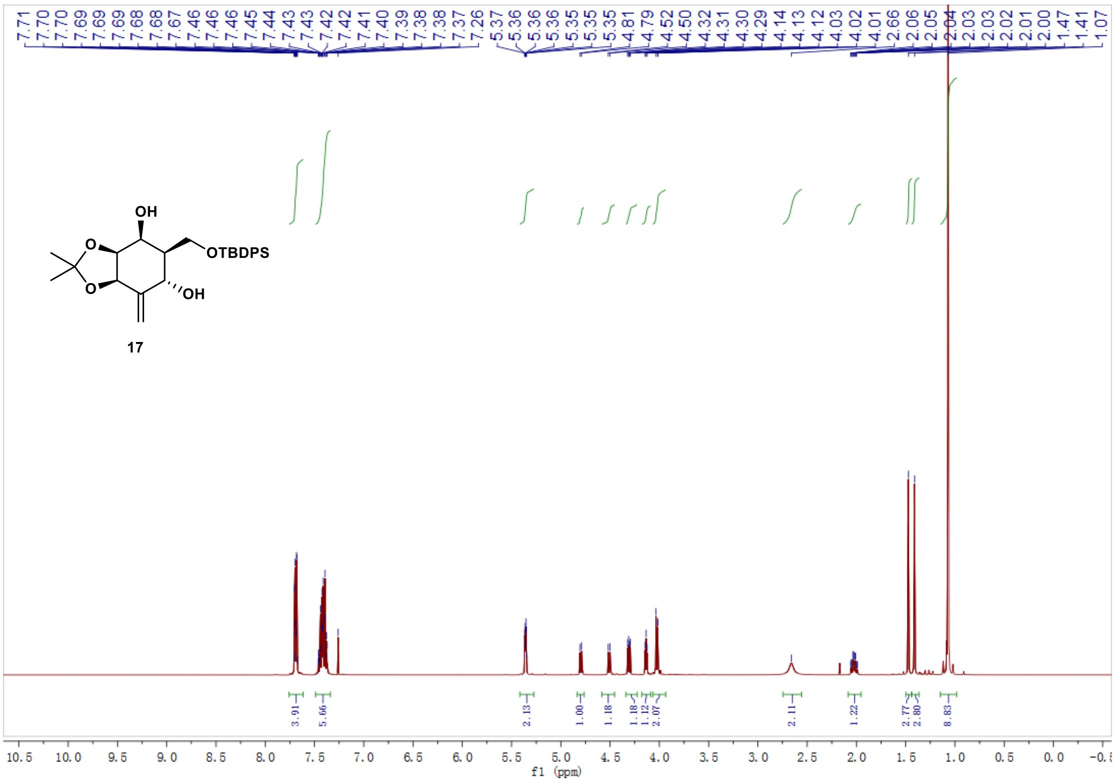

^{13}C NMR (101 MHz, $CDCl_3$, 25°C) of compound **17**

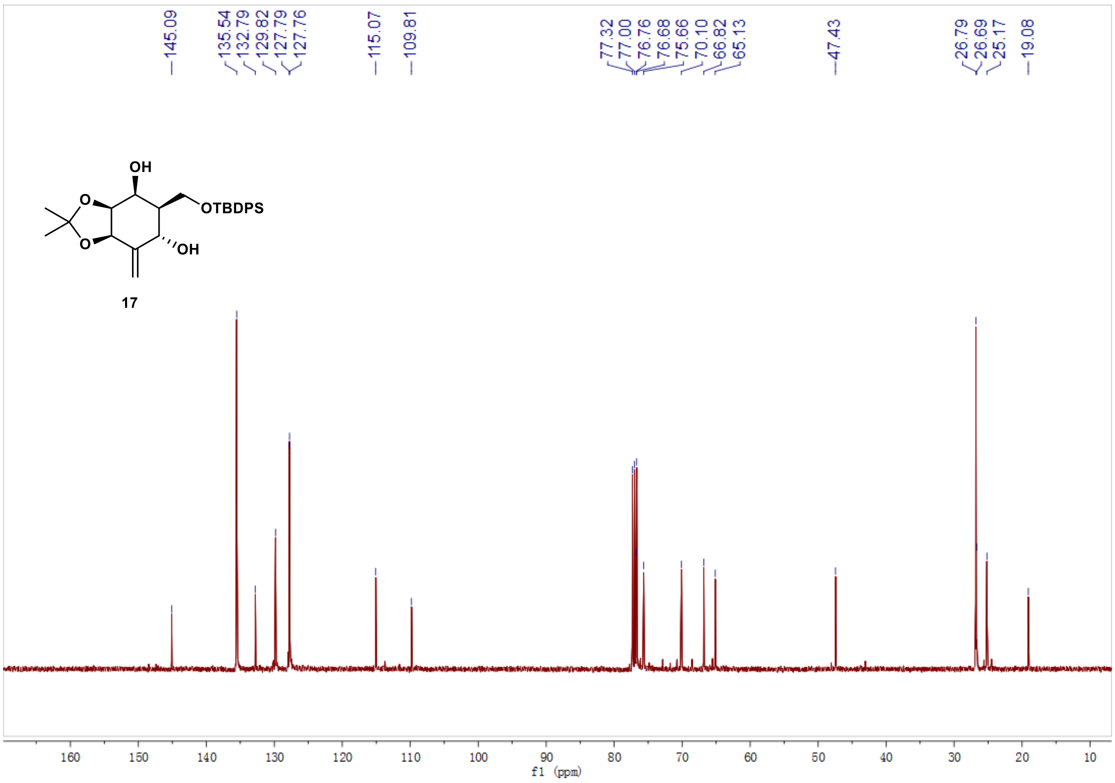

Supplementary Figure 17. NMR spectra of compound 17

^{1}H NMR (400 MHz, $CDCl_3$, 25°C) of compound **18**

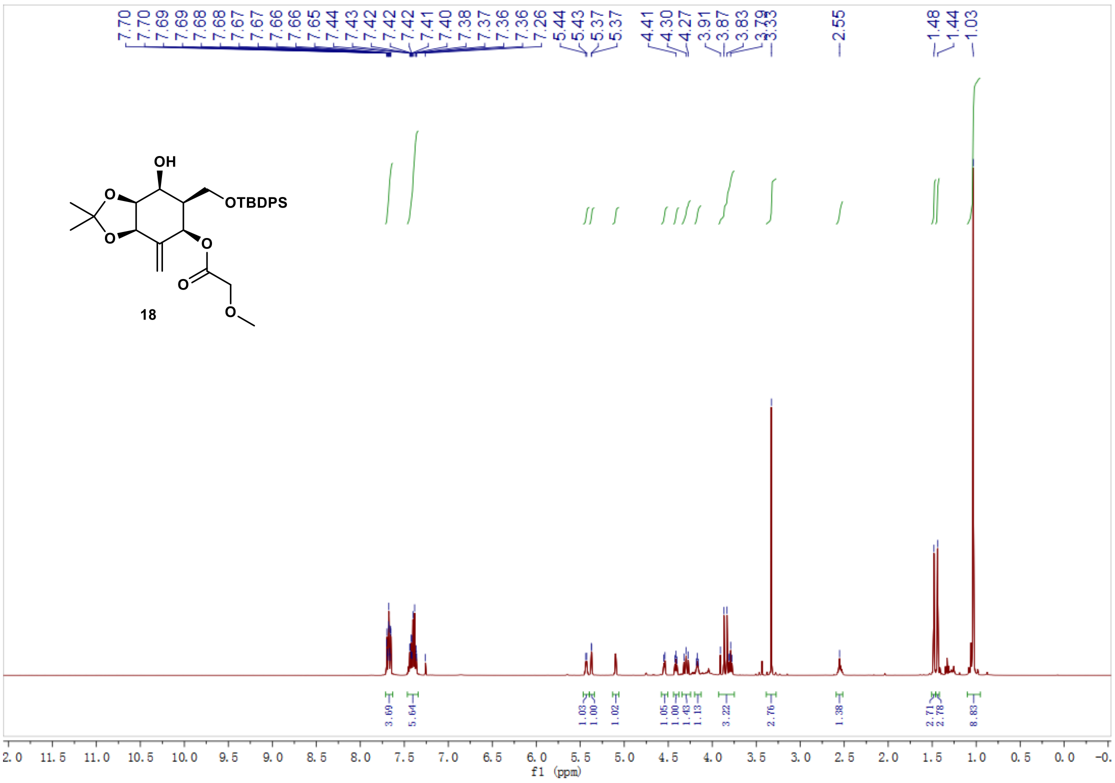

^{13}C NMR (101 MHz, $CDCl_3$, 25°C) of compound **18**

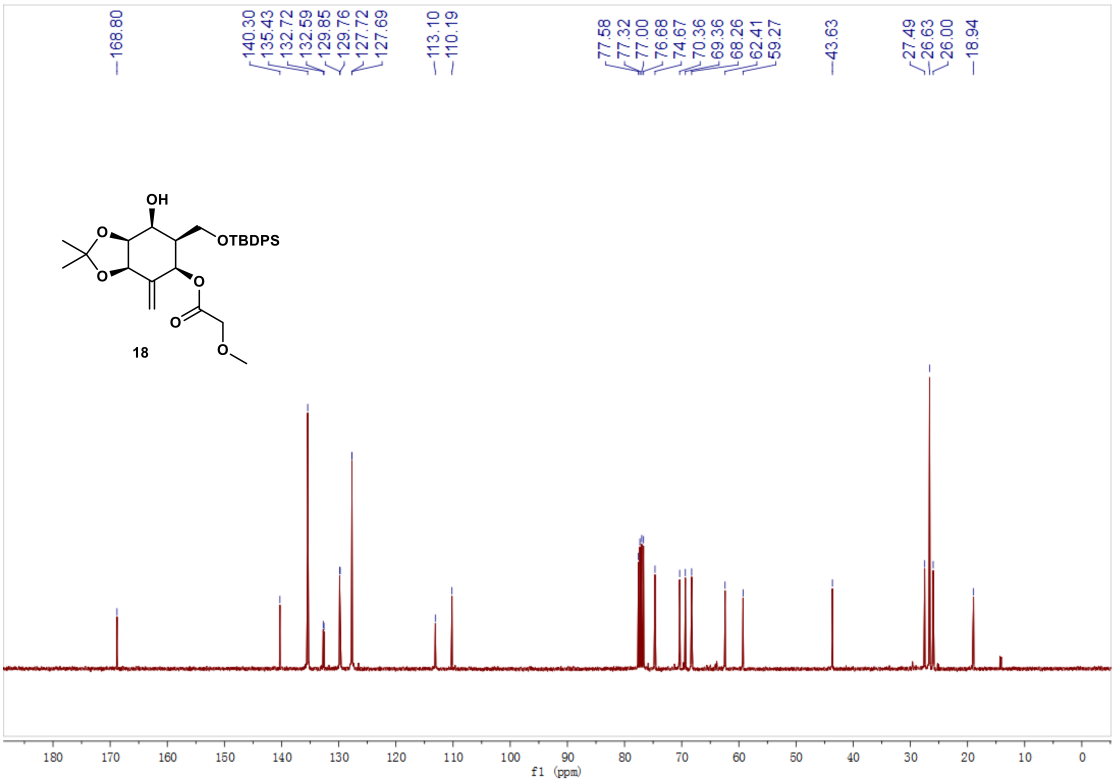

Supplementary Figure 18. NMR spectra of compound 18

^{1}H NMR (400 MHz, $CDCl_3$, 25°C) of compound **21**

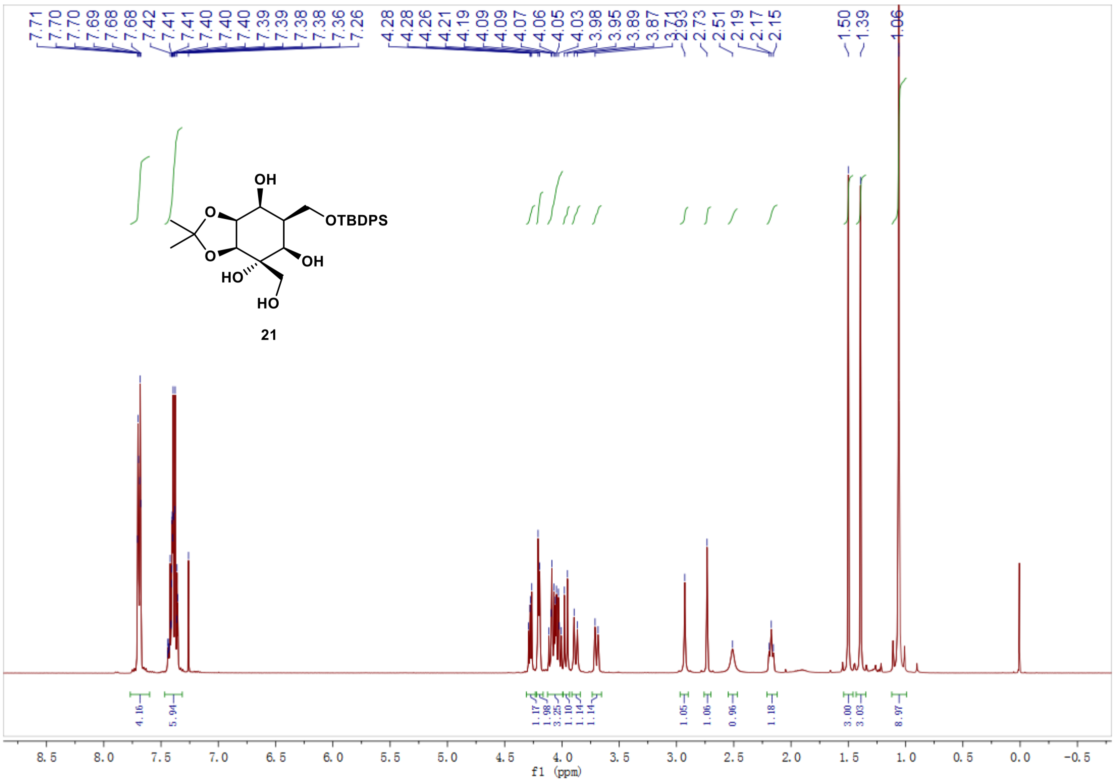

^{13}C NMR (101 MHz, $CDCl_3$, 25°C) of compound **21**

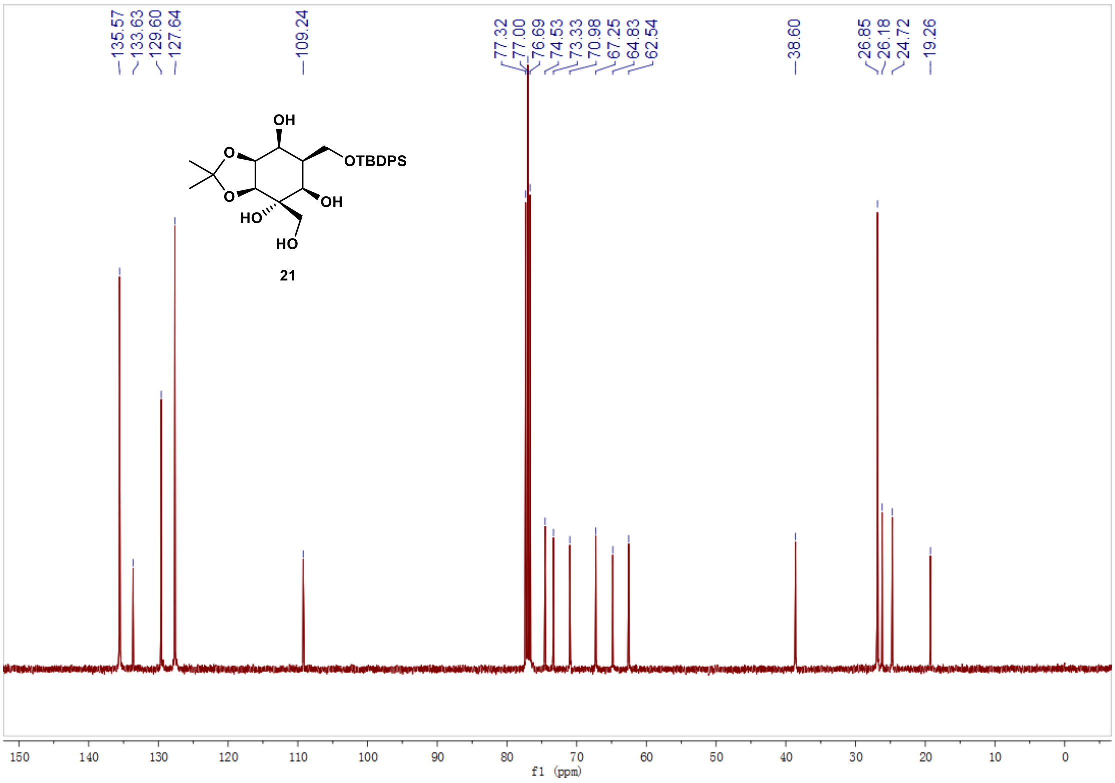

Supplementary Figure 19. NMR spectra of compound 21

^{1}H NMR (400 MHz, $CDCl_3$, 25°C) of compound **18a**

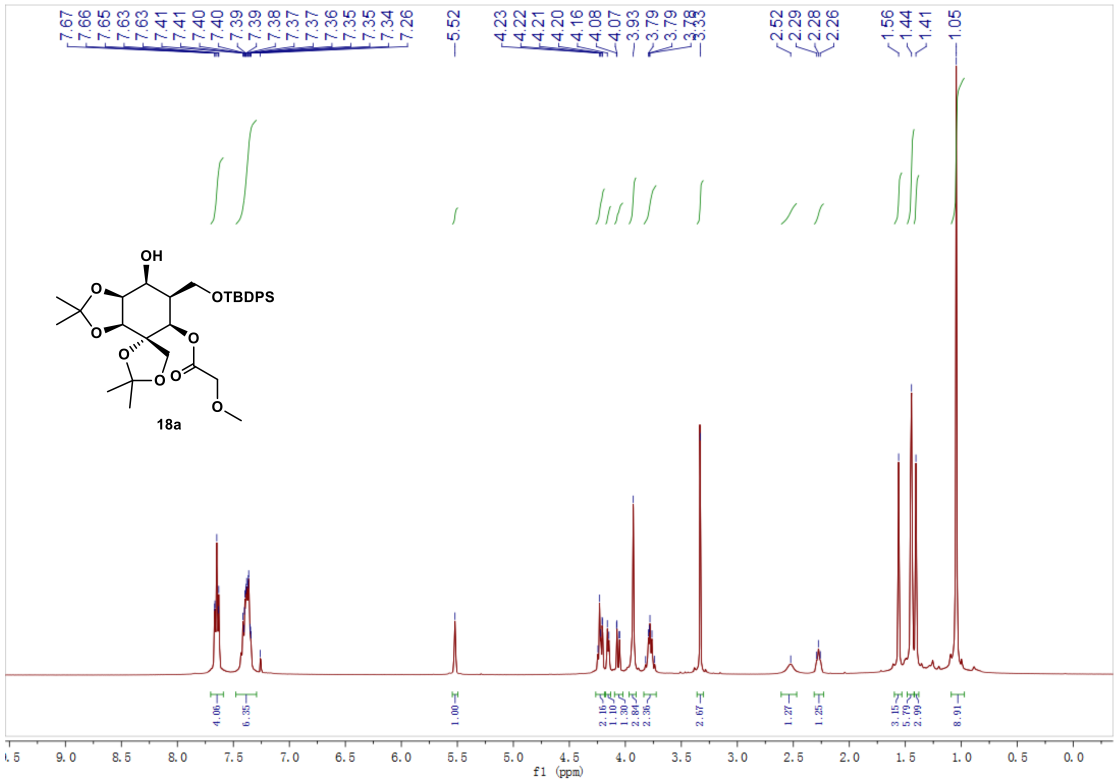

^{13}C NMR (101 MHz, $CDCl_3$, 25°C) of compound **18a**

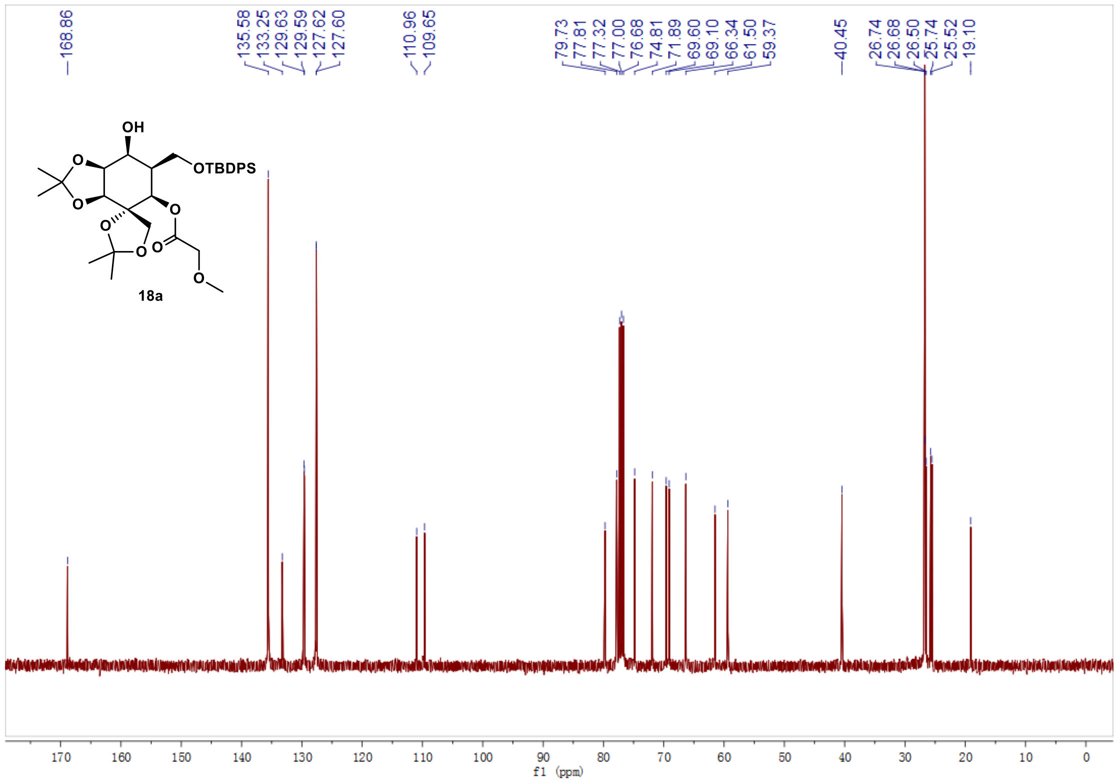

Supplementary Figure 20. NMR spectra of compound 18a

^{1}H NMR (400 MHz, $CDCl_3$, 25°C) of compound **19**

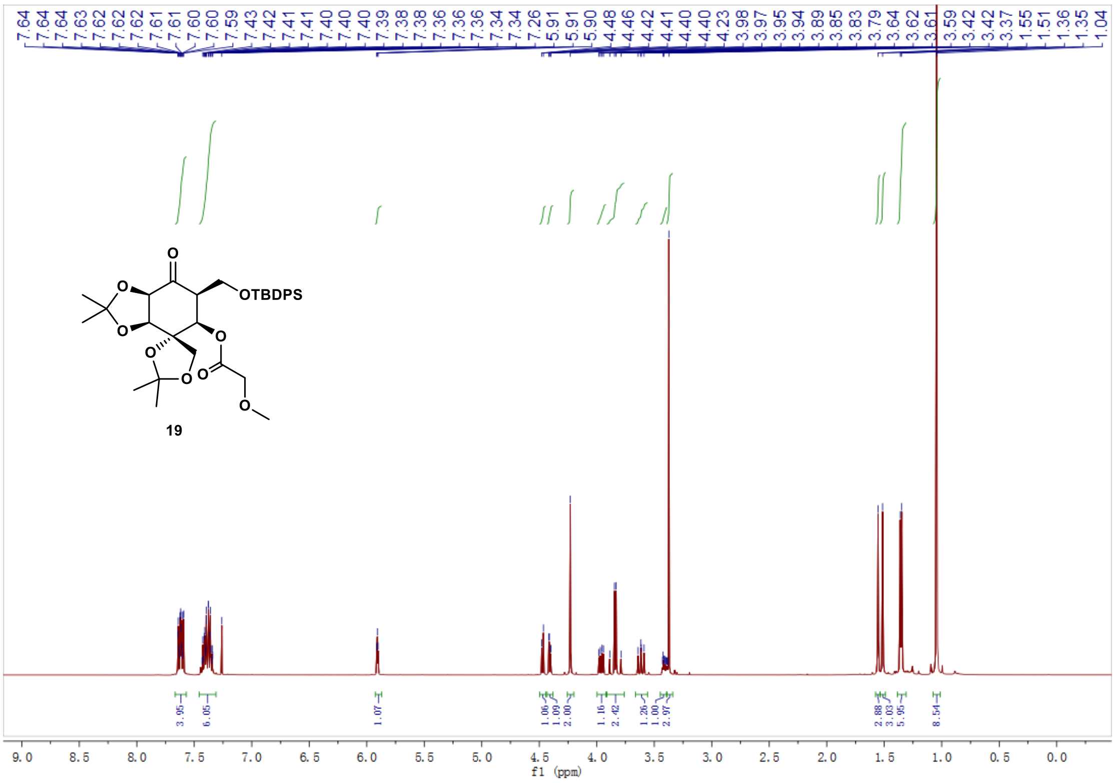

^{13}C NMR (101 MHz, $CDCl_3$, 25°C) of compound **19**

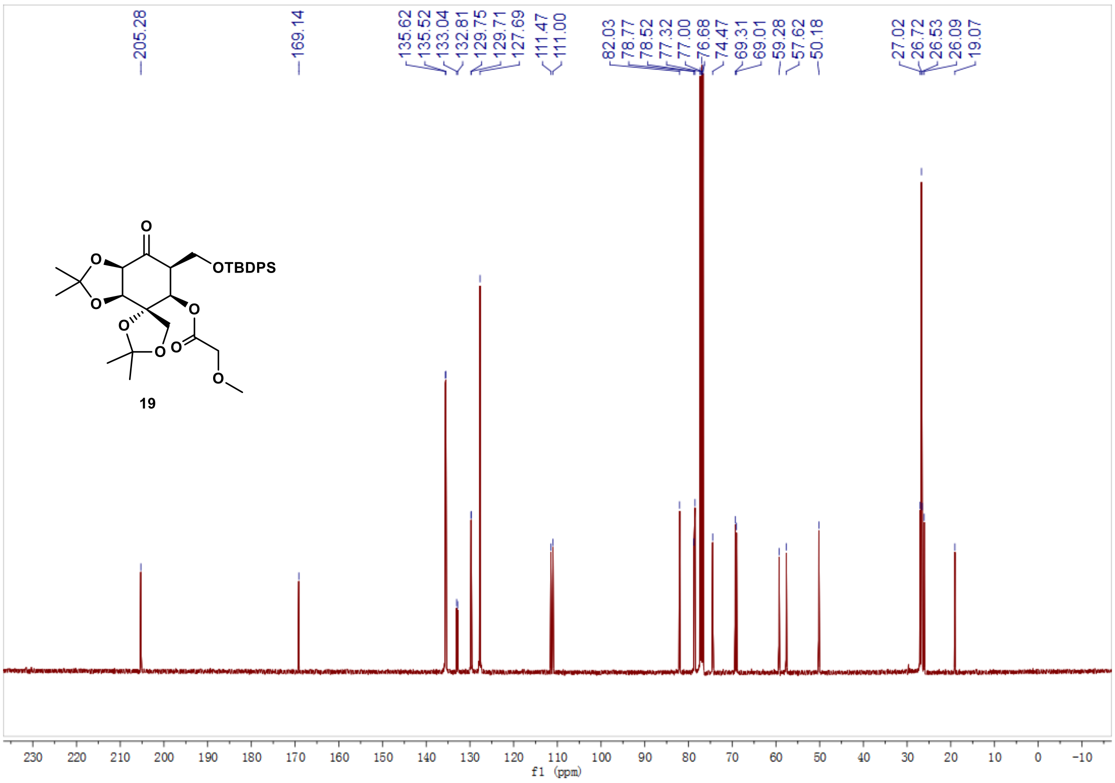

Supplementary Figure 21. NMR spectra of compound 19

^{1}H NMR (400 MHz, $CDCl_3$, 25°C) of compound **19a**

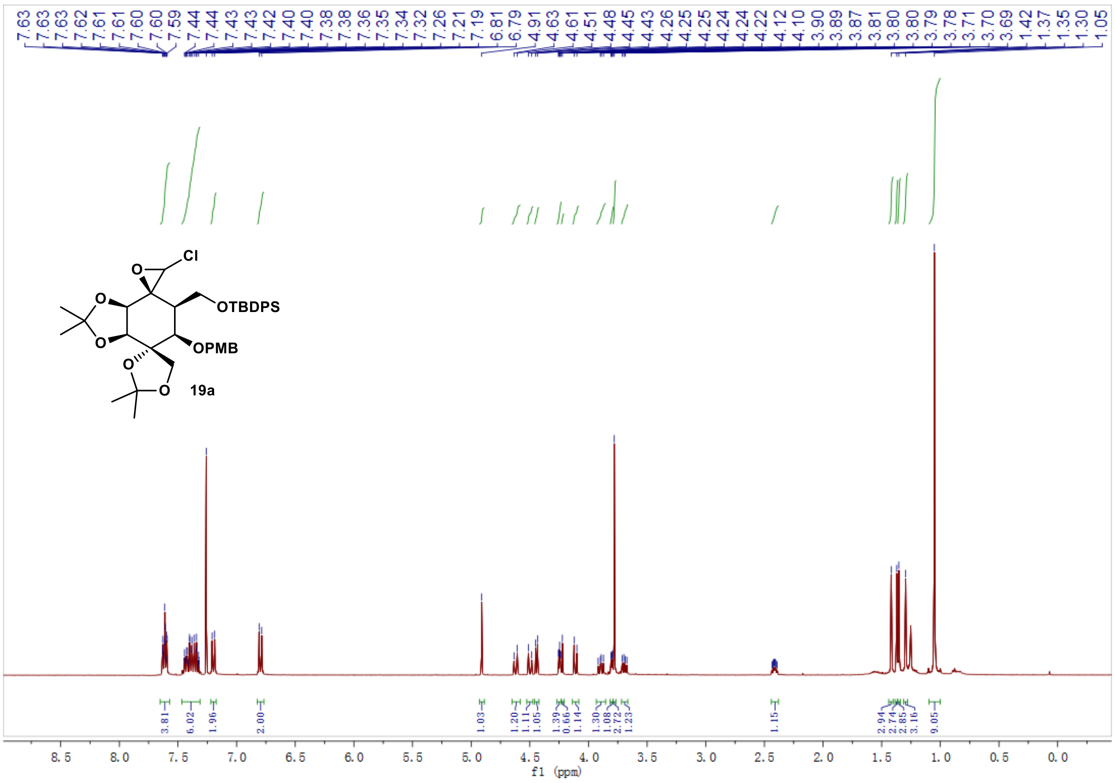

^{13}C NMR (101 MHz, $CDCl_3$, 25°C) of compound **19a**

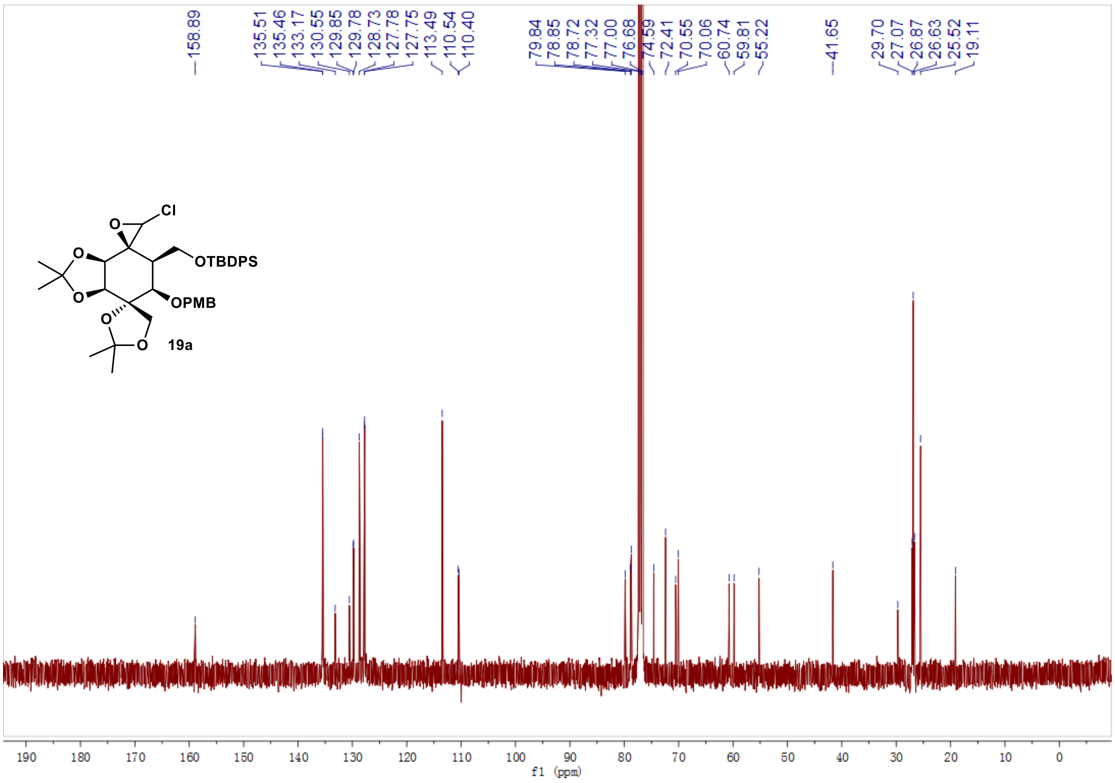

Supplementary Figure 22. NMR spectra of compound 19a

^{1}H NMR (400 MHz, $CDCl_3$, 25°C) of compound **20**

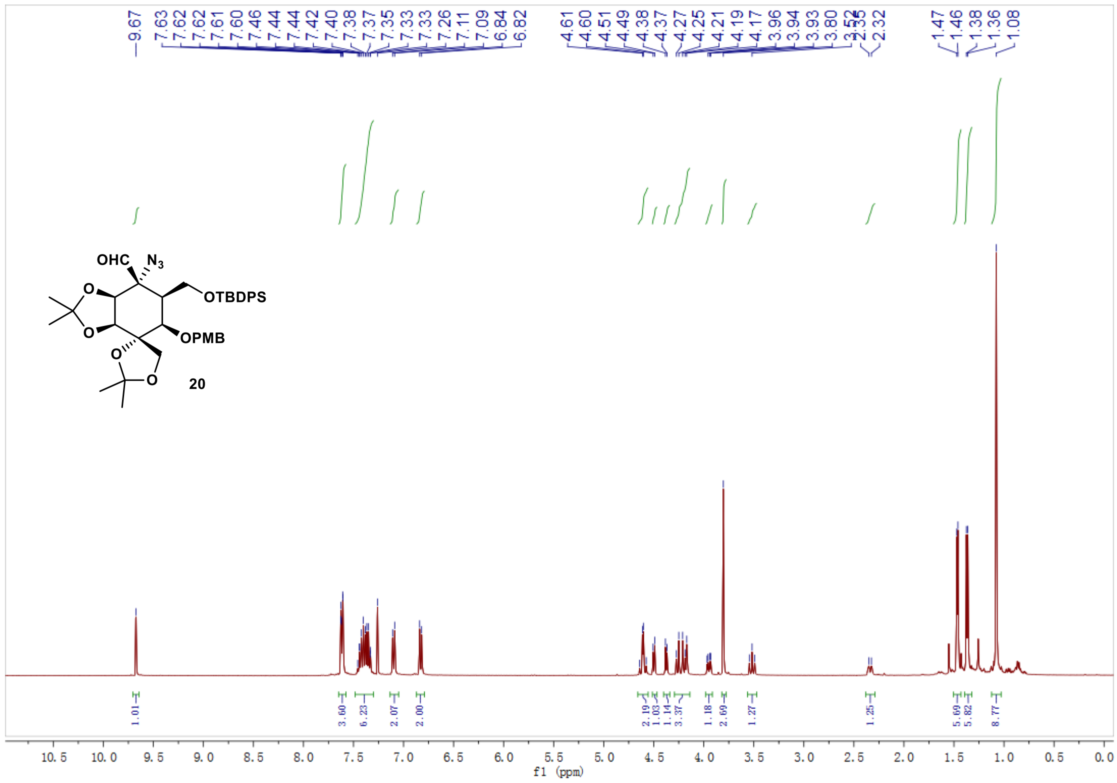

^{13}C NMR (101 MHz, $CDCl_3$, 25°C) of compound **20**

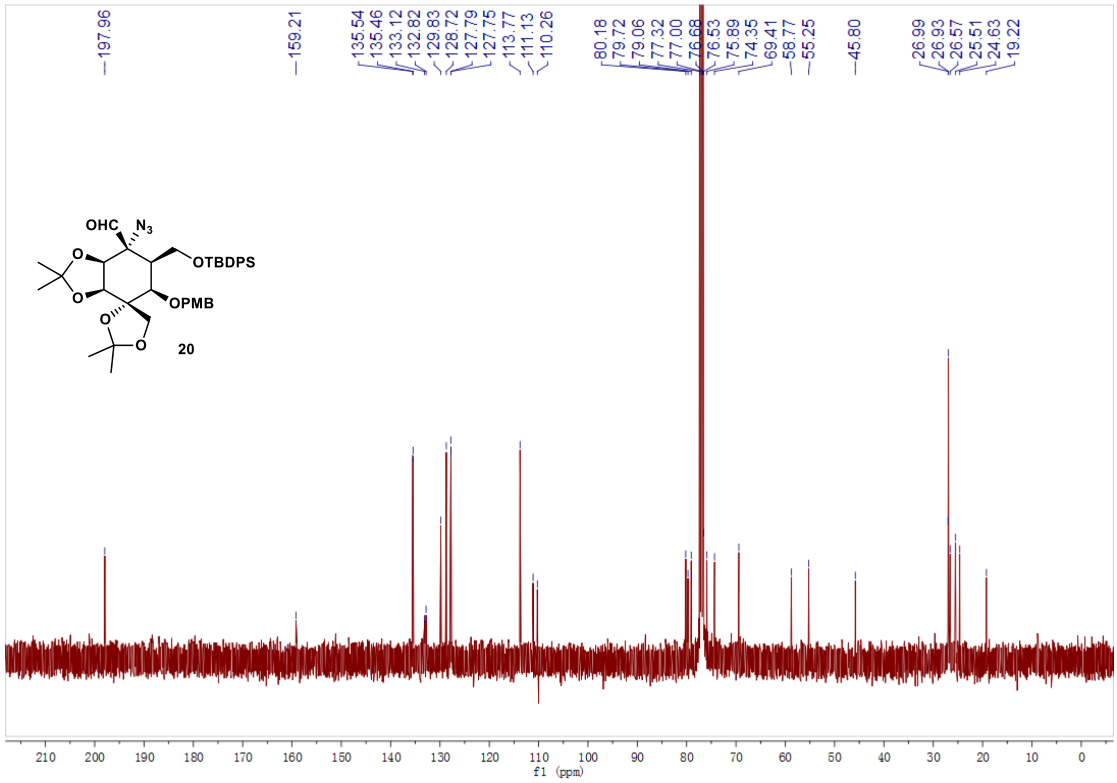

Supplementary Figure 23. NMR spectra of compound 20

^{1}H NMR (400 MHz, $CDCl_3$, 25°C) of compound **22a**

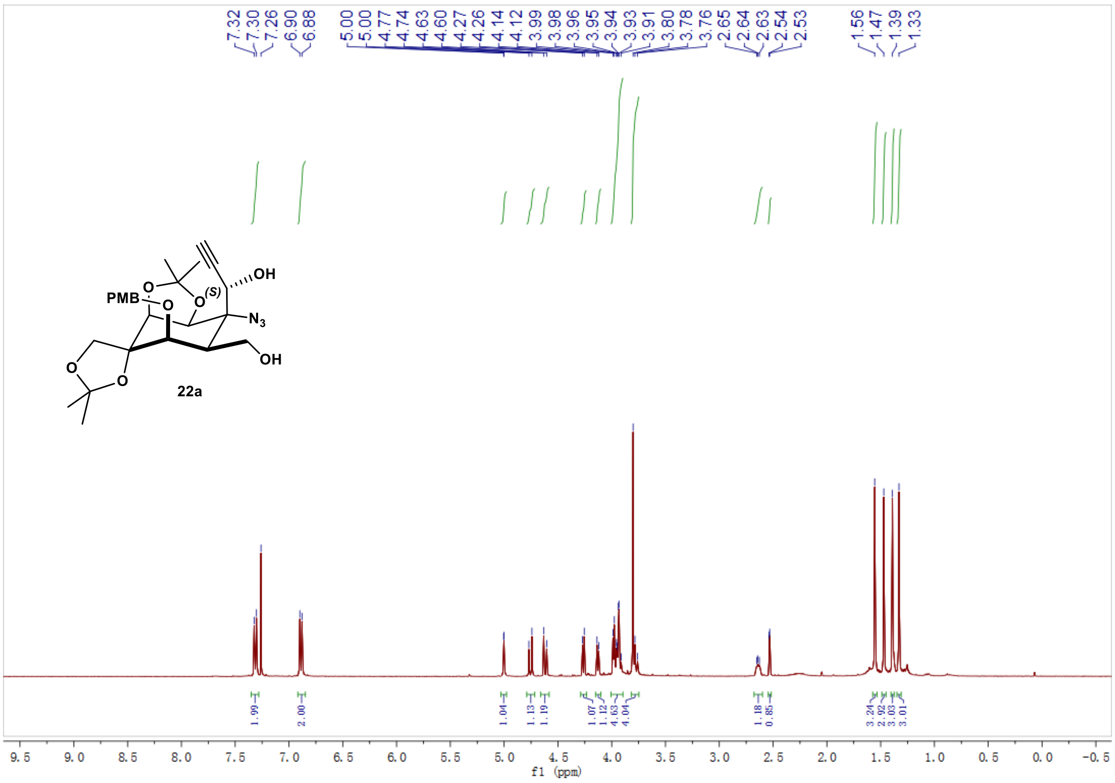

^{13}C NMR (101 MHz, $CDCl_3$, 25°C) of compound **22a**

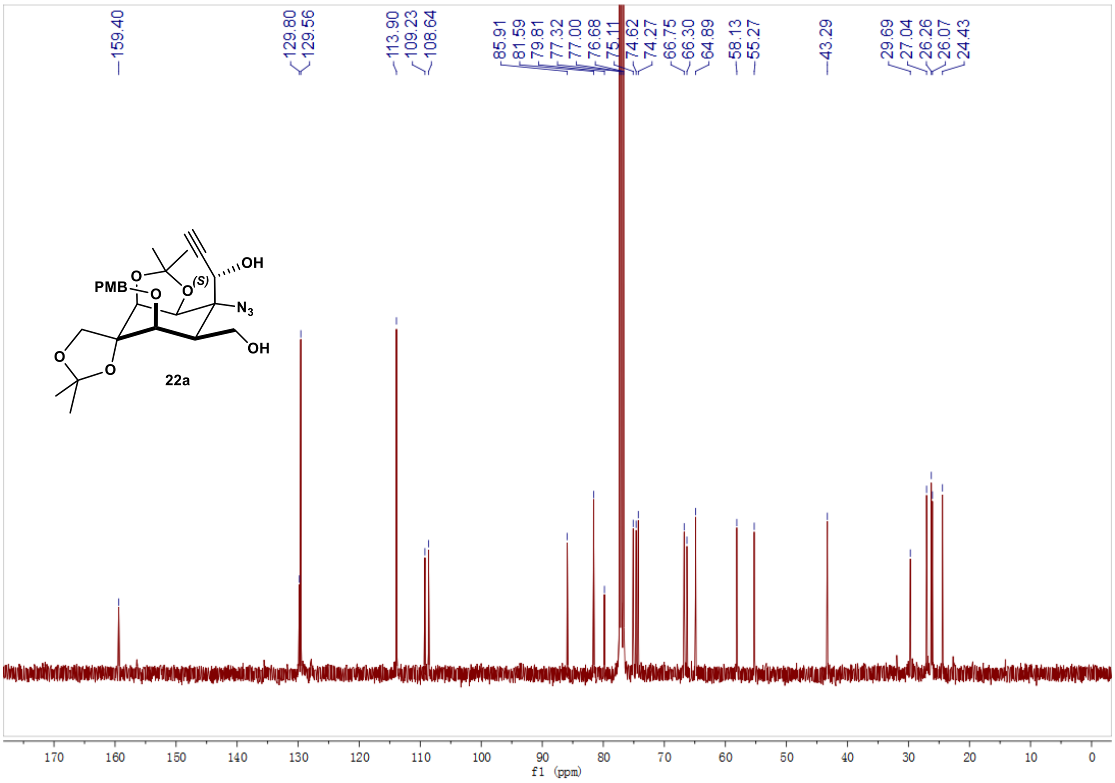

Supplementary Figure 24. NMR spectra of compound 22a

^{1}H NMR (400 MHz, $CDCl_3$, 25°C) of compound **22**

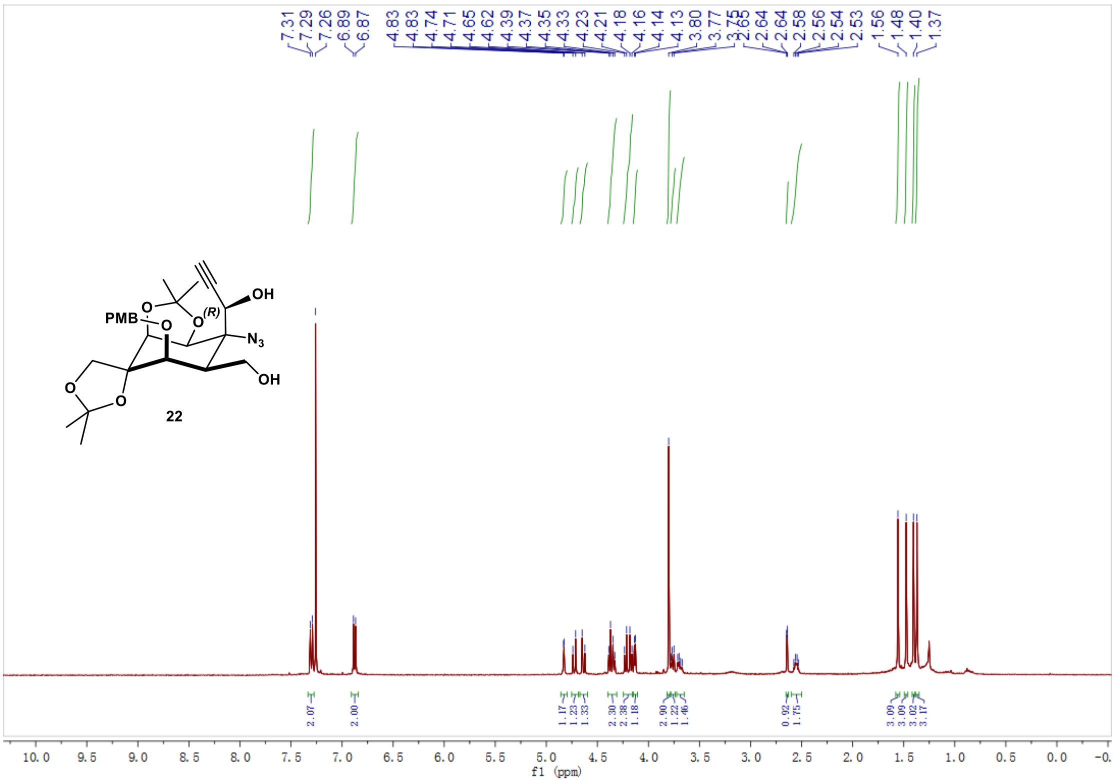

^{13}C NMR (101 MHz, $CDCl_3$, 25°C) of compound **22**

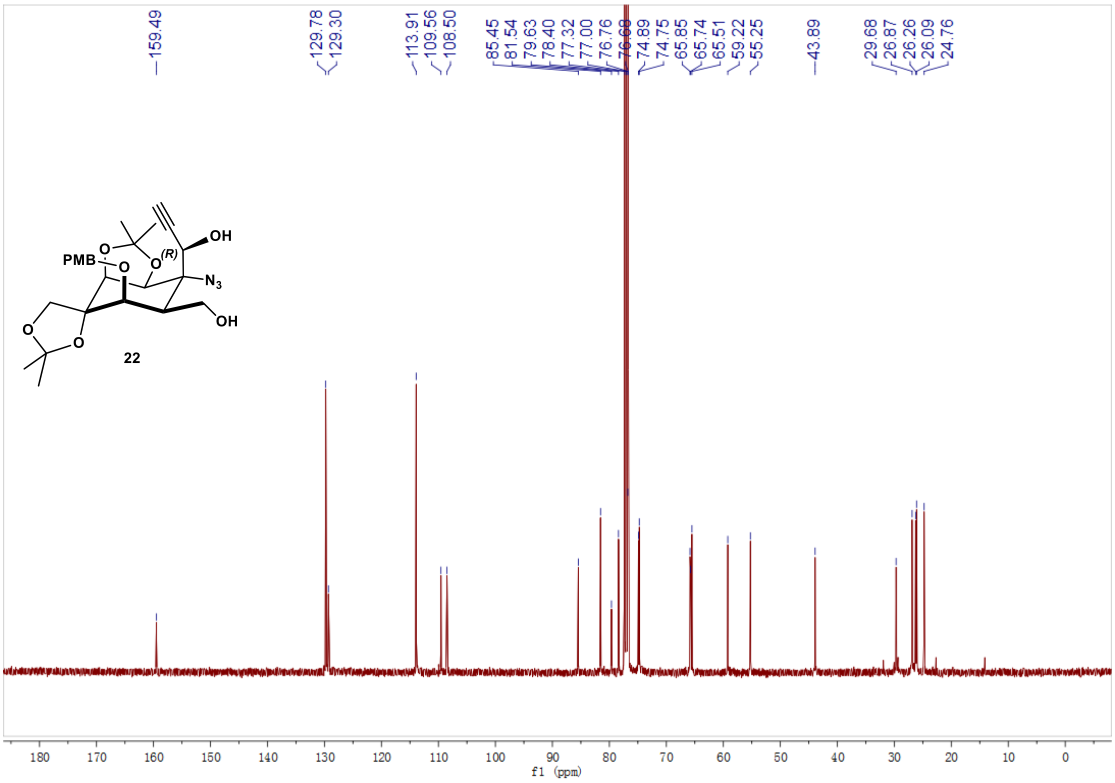

Supplementary Figure 25. NMR spectra of compound 22

^{1}H NMR (400 MHz, $CDCl_3$, 25°C) of compound **23**

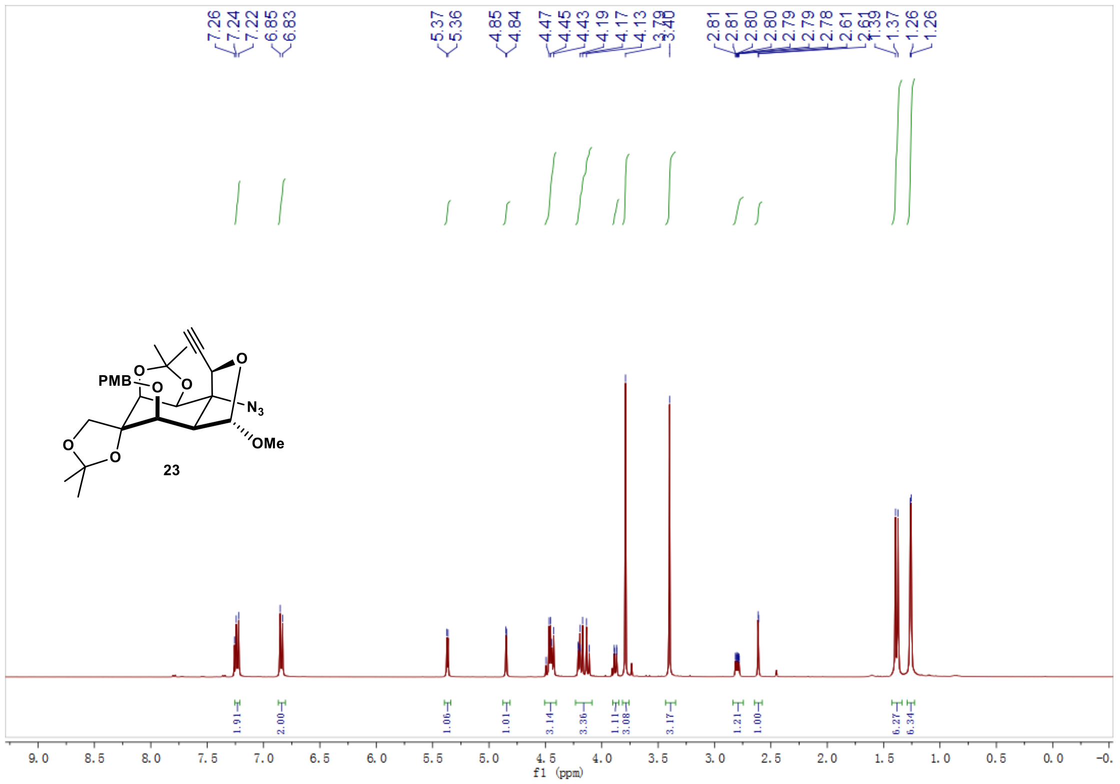

^{13}C NMR (101 MHz, $CDCl_3$, 25°C) of compound **23**

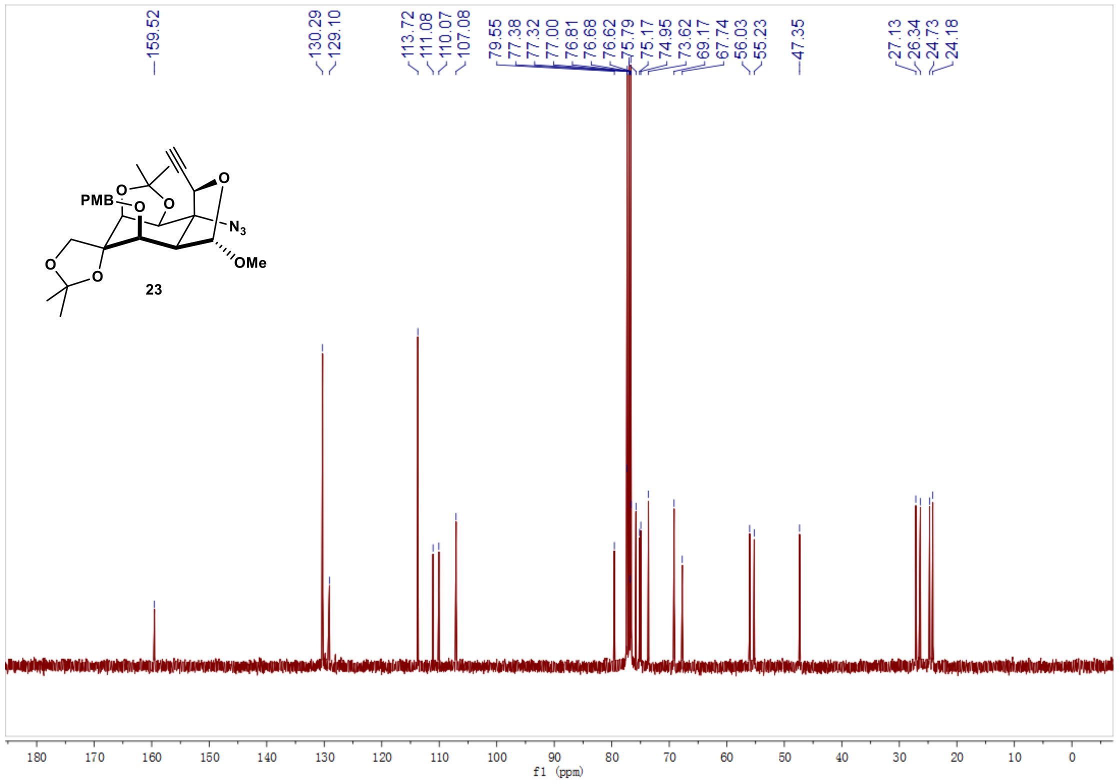

COSY of compound **23**

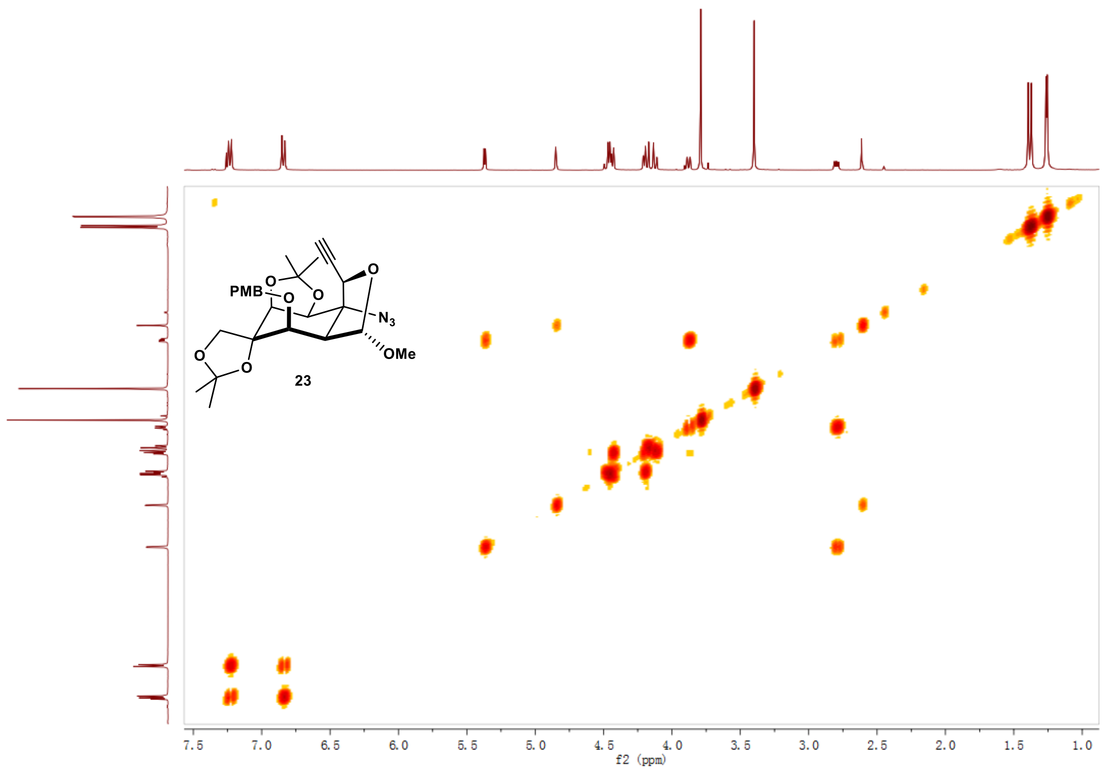

HSQC of compound **23**

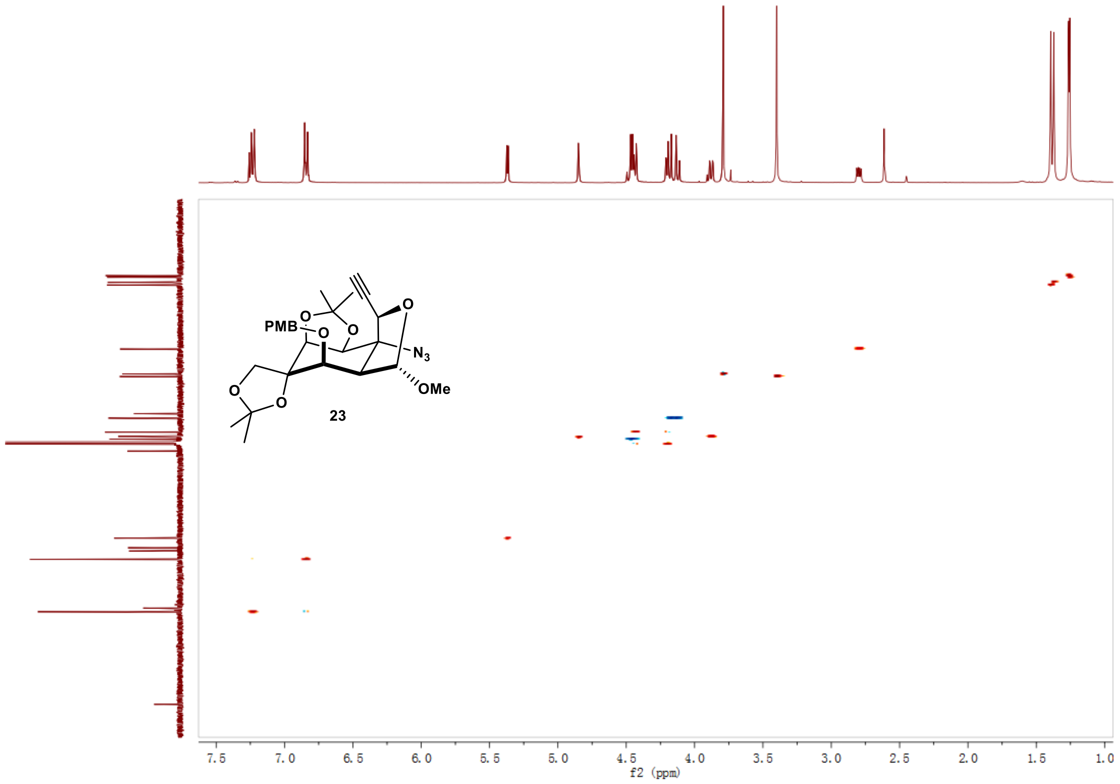

NOESY of compound **23**

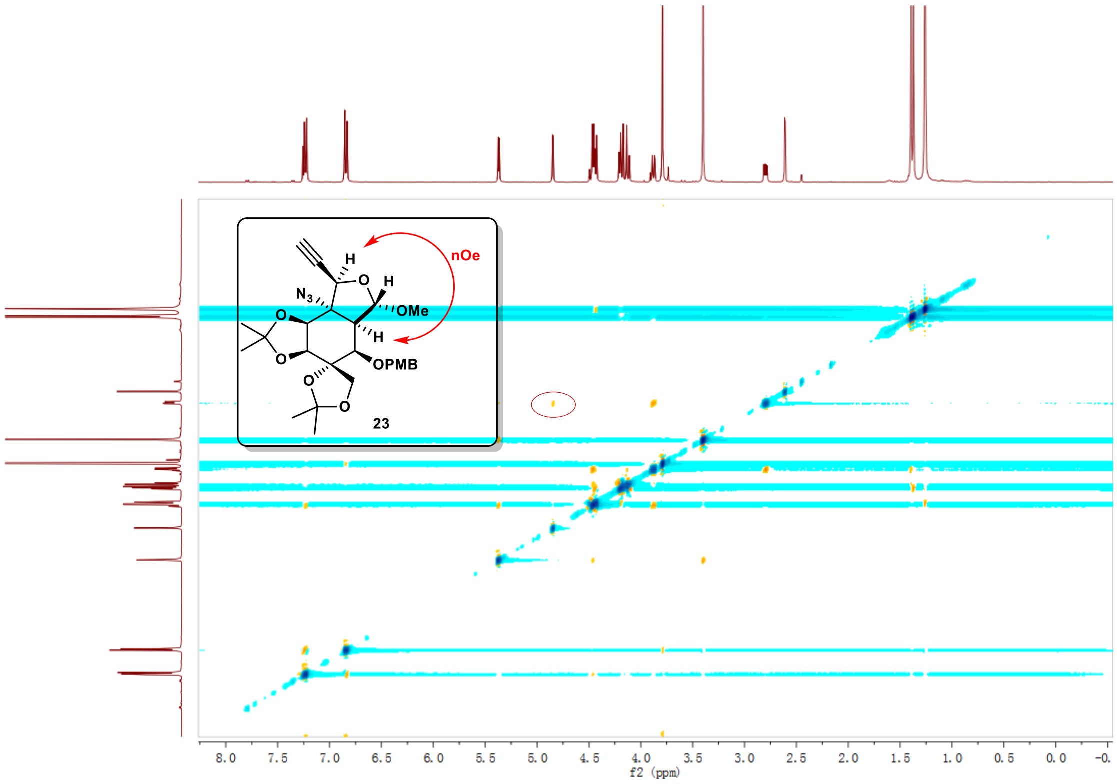

Supplementary Figure 26. NMR spectra of compound 23

^{1}H NMR (400 MHz, $CDCl_3$, 25ºC) of compound **24**

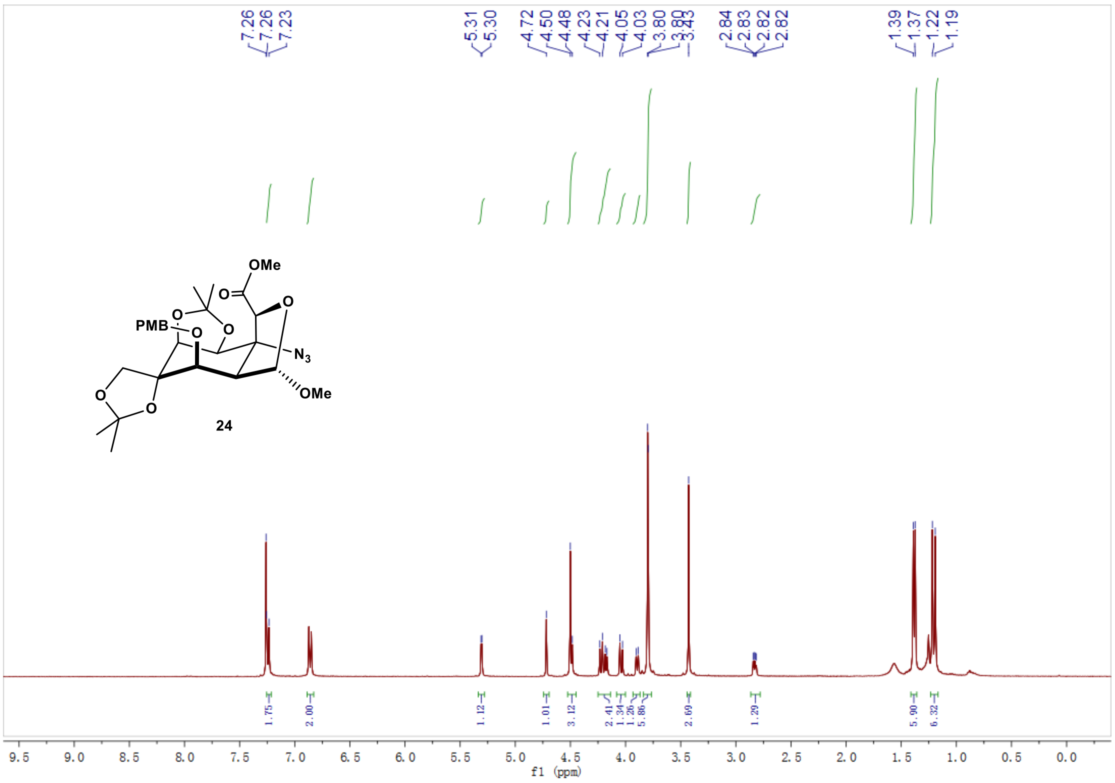

^{13}C NMR (101 MHz, $CDCl_3$, 25°C) of compound **24**

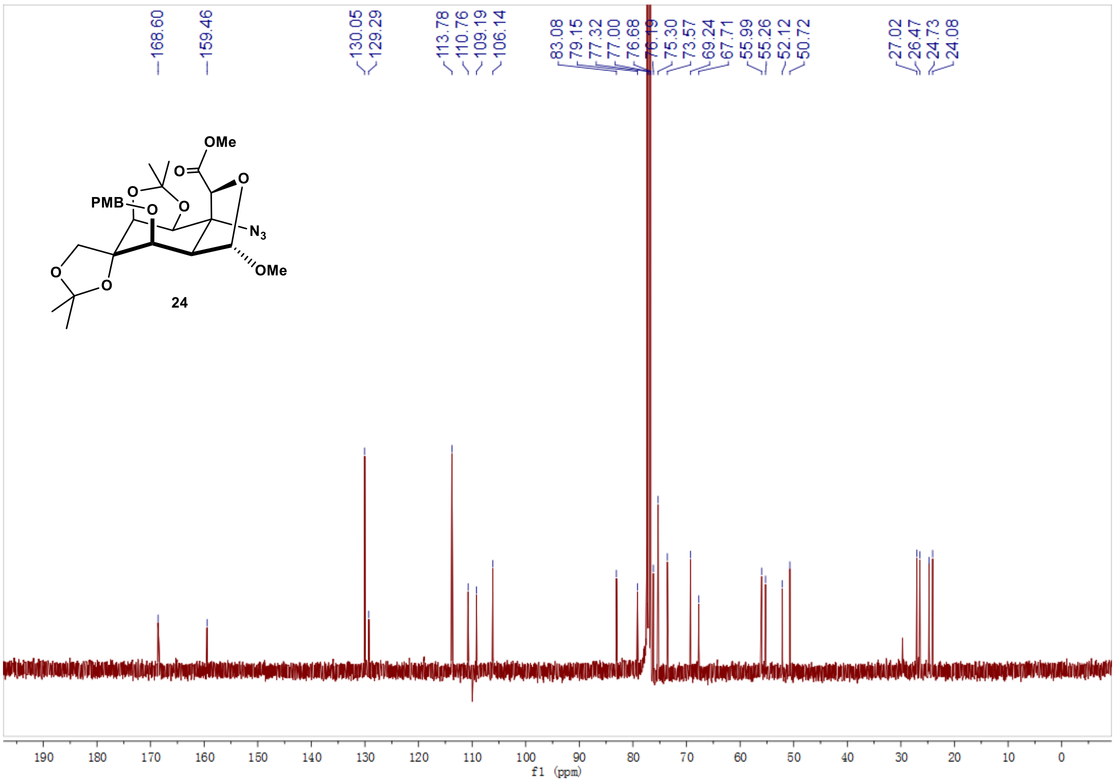

Supplementary Figure 27. NMR spectra of compound 24

^{1}H NMR (400 MHz, $CDCl_3$, 25°C) of compound **25**

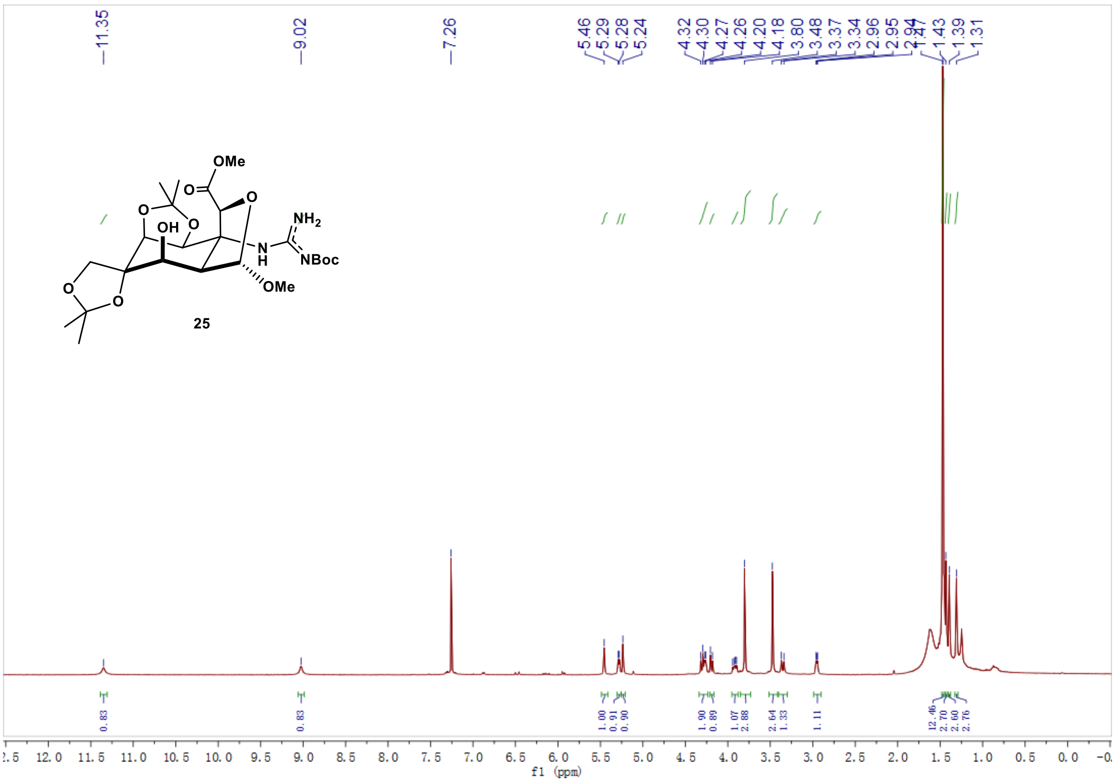

^{13}C NMR (101 MHz, $CDCl_3$, 25°C) of compound **25**

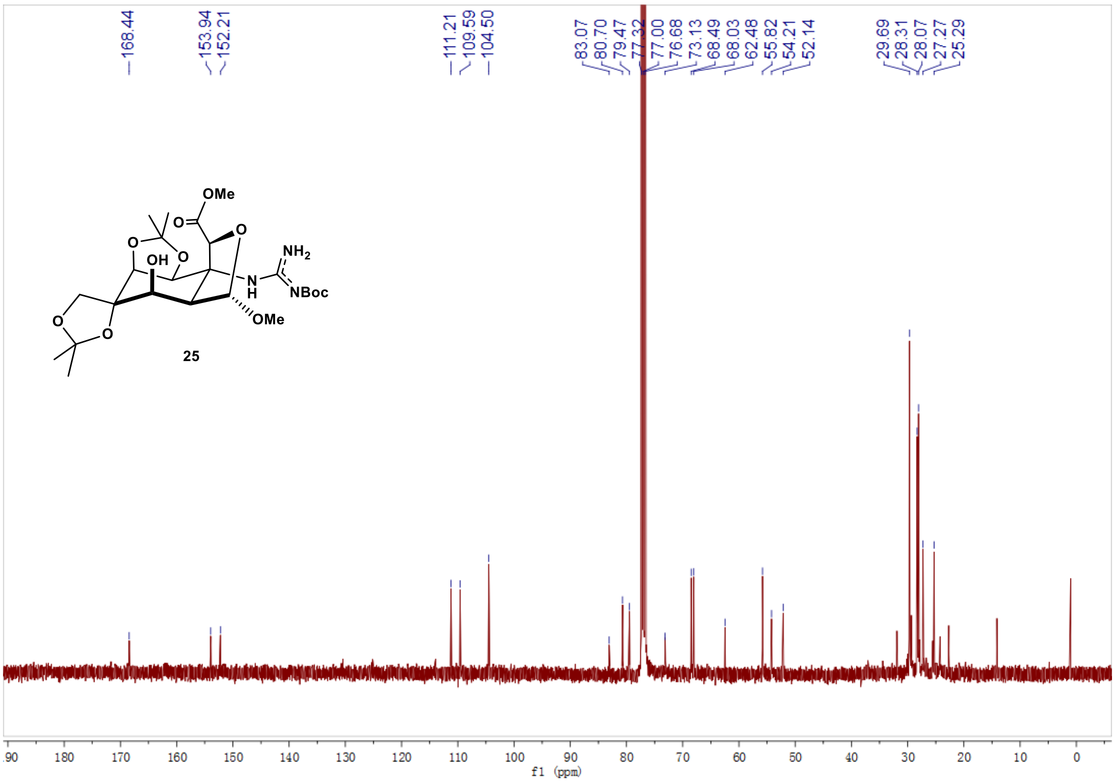

Supplementary Figure 28. NMR spectra of compound 25

^{1}H NMR (400 MHz, $CDCl_3$, 25°C) of compound **29**

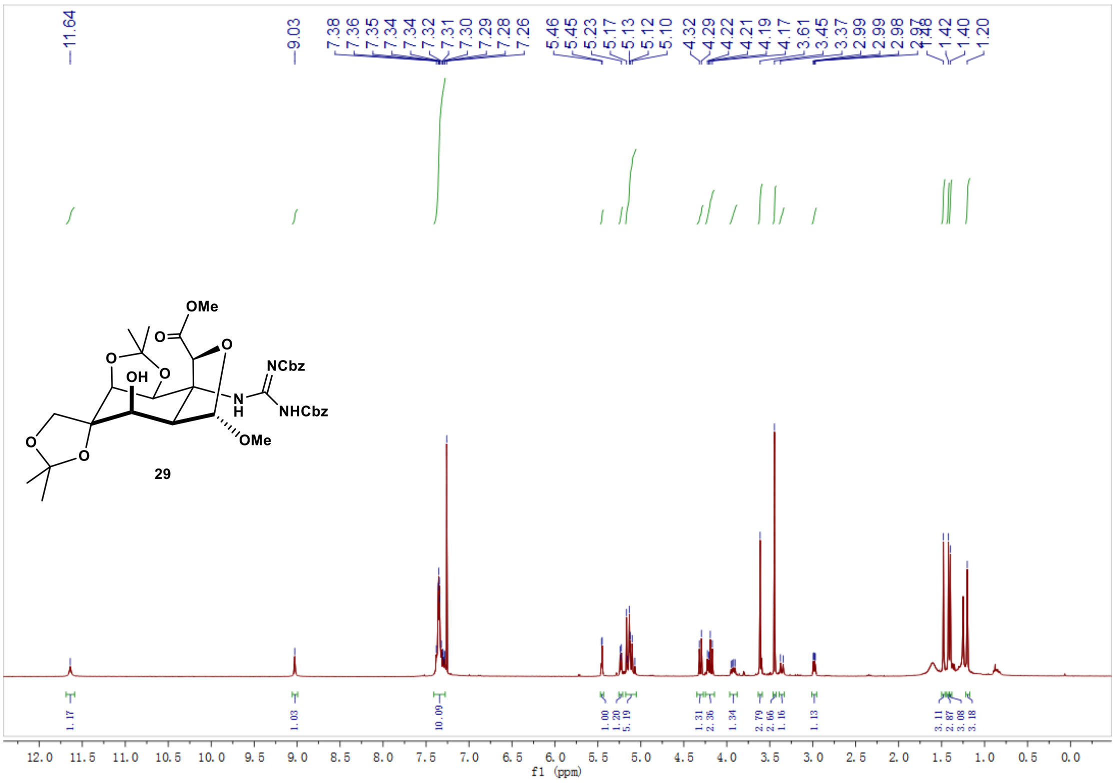

^{13}C NMR (101 MHz, $CDCl_3$, 25°C) of compound **29**

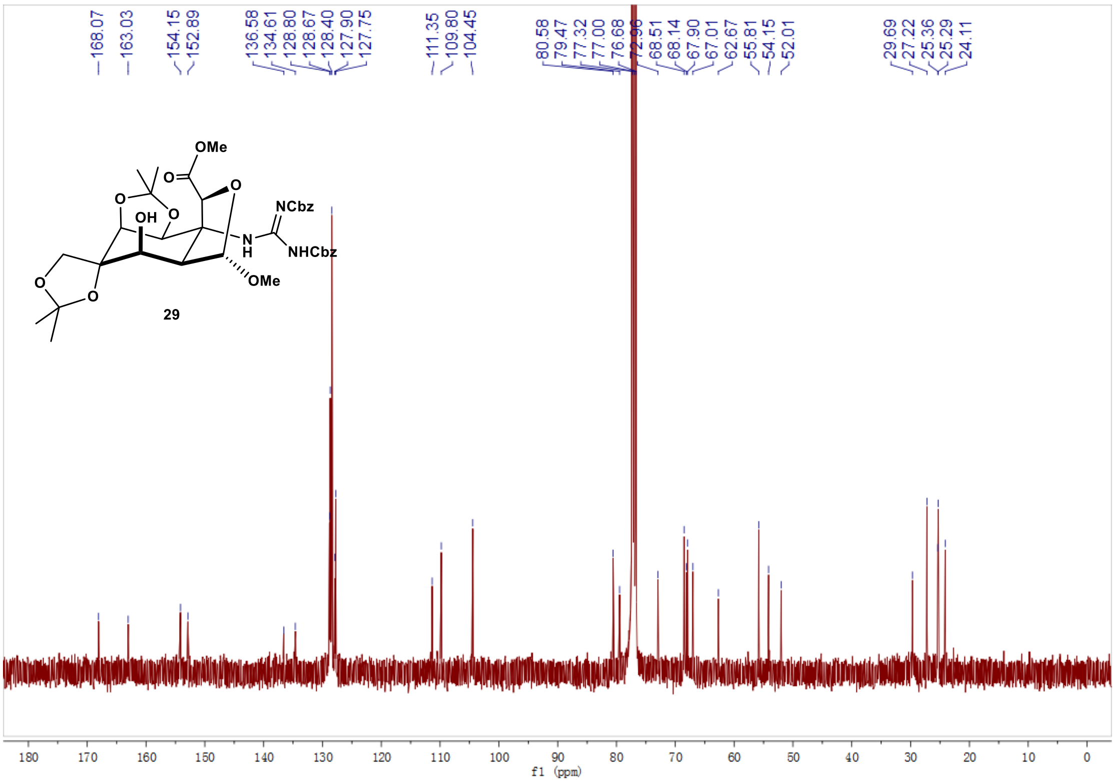

Supplementary Figure 29. NMR spectra of compound 29

^{1}H NMR (400 MHz, CD_3OD, 25°C) of compound **30**

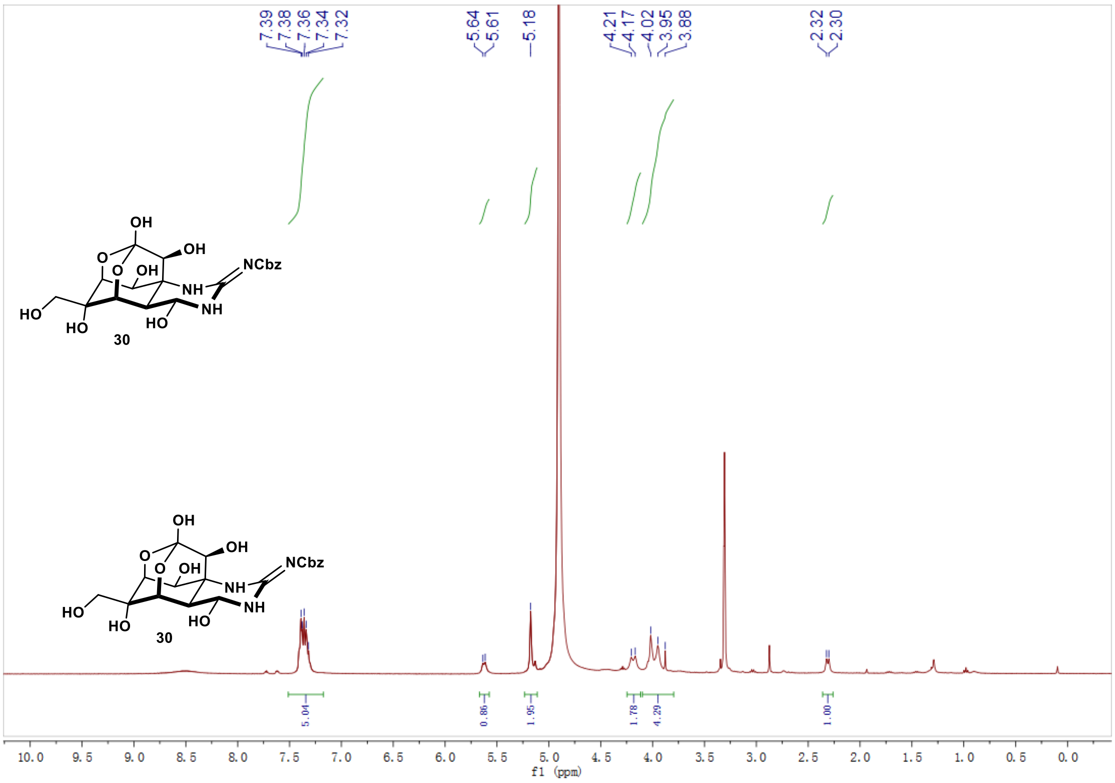

^{13}C NMR (151 MHz, CD_3OD, 25ºC) of compound **30**

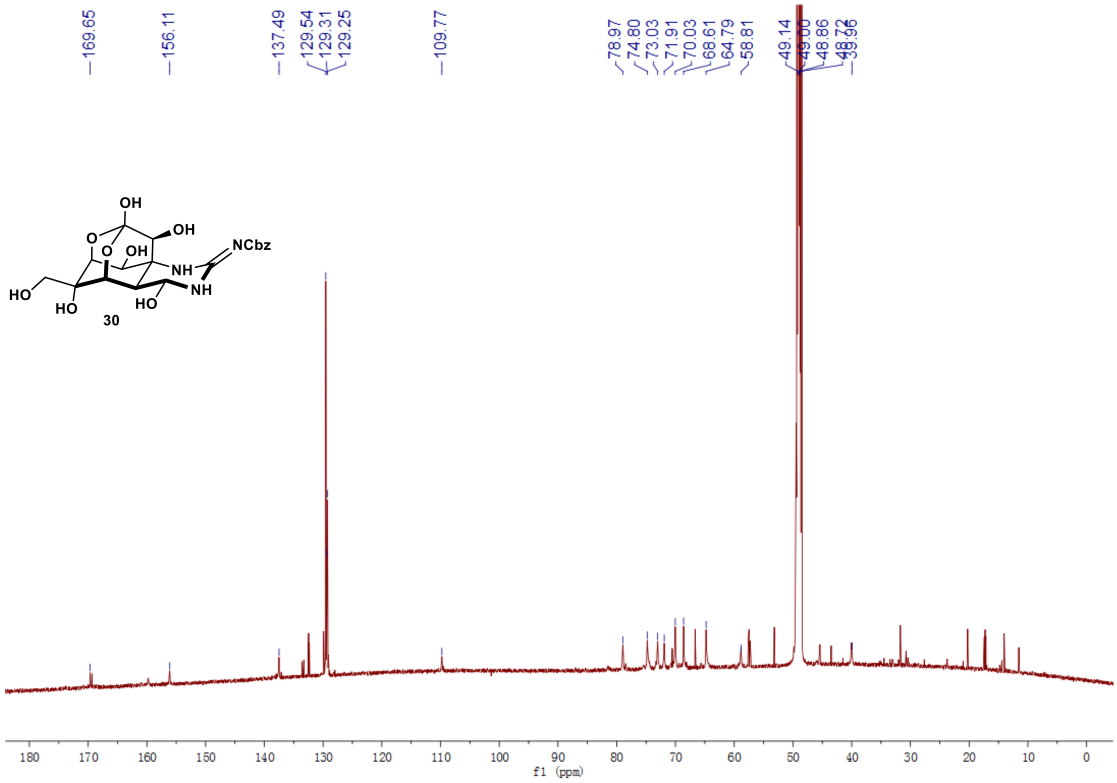

Supplementary Figure 30. NMR spectra of compound 30

^{1}H NMR (600 MHz, 5% CD_3CO_2D/D_2O, 25ºC) of Tetrodotoxin **1 (Method A)**

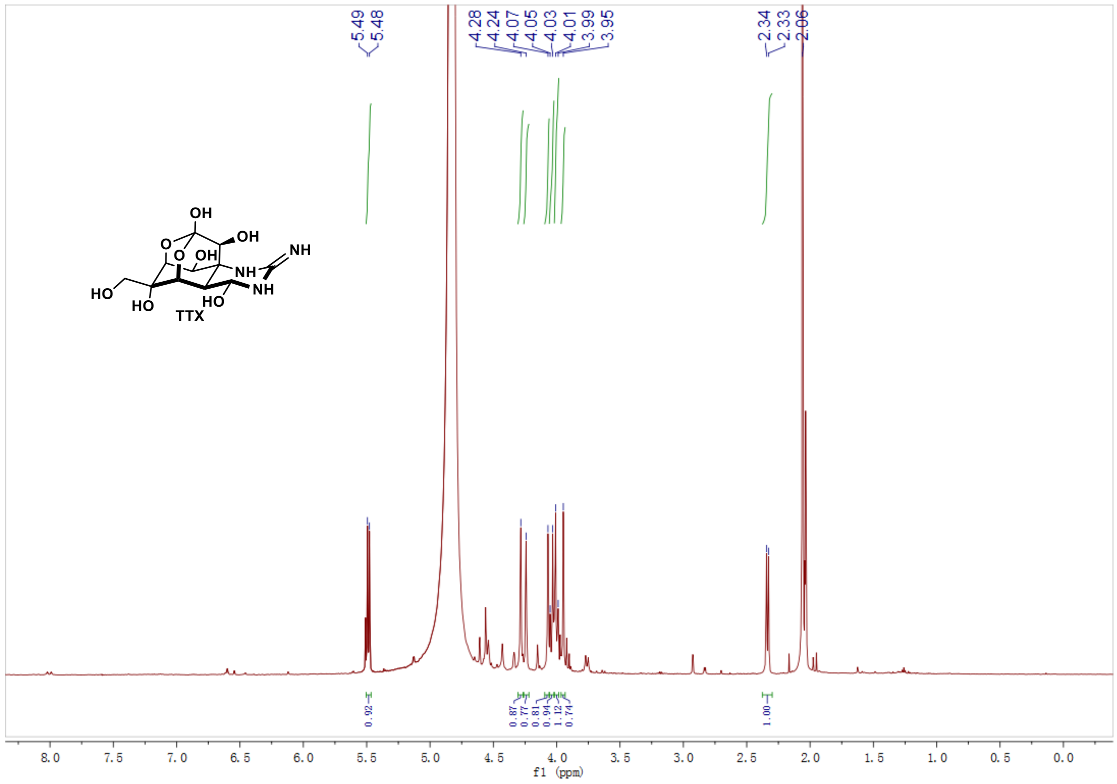

^{13}C NMR (151 MHz, 5% CD_3CO_2D/D_2O, 25^oC) of Tetrodotoxin **1 (Method A)**

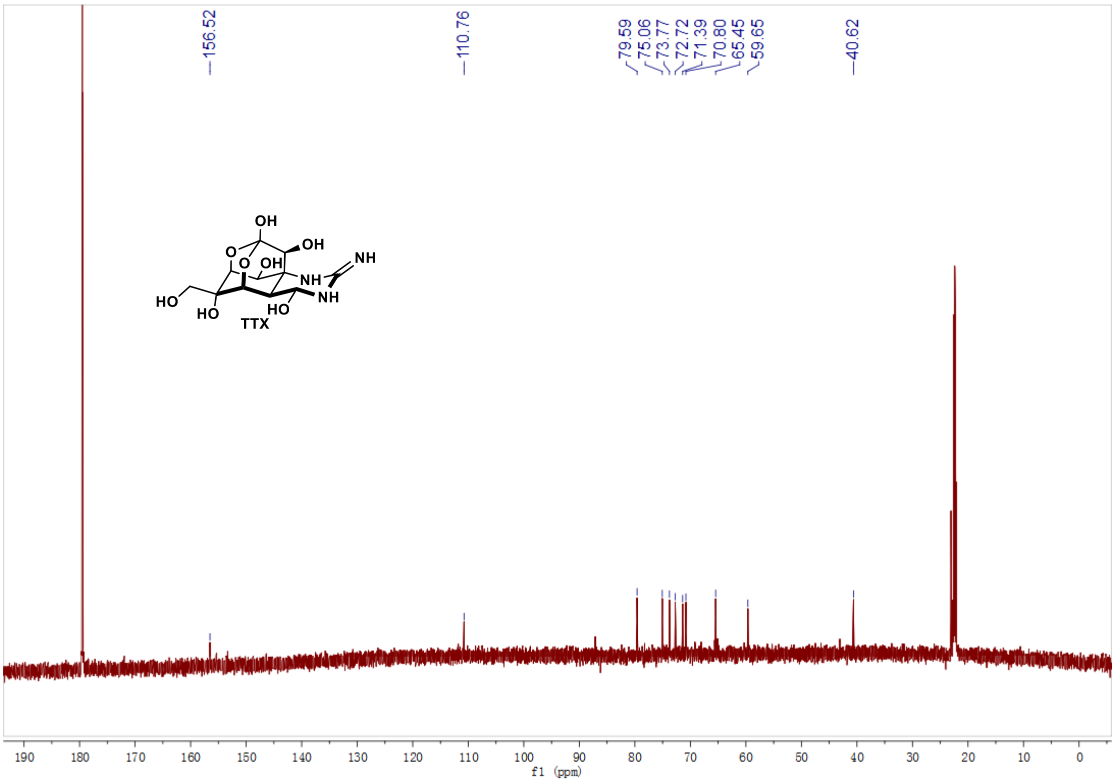

Supplementary Figure 31. NMR spectra of compound 1 Tetrodotoxin, (Method A)

^1H NMR (400 MHz, 5% CD_3CO_2D/D_2O, 25^oC) of Tetrodotoxin **1 (Method B)**

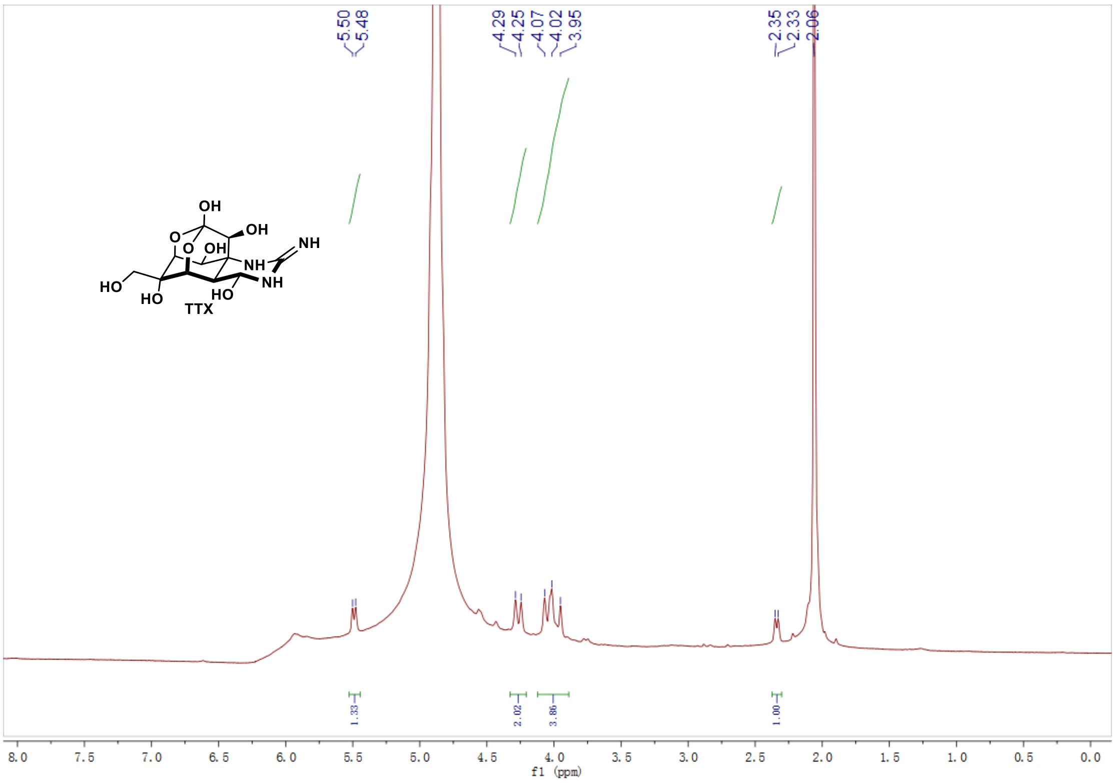

Supplementary Figure 32. NMR spectra of compound 1 Tetrodotoxin, (Method B)

^{1}H NMR (400 MHz, 5% CD_3CO_2D/D_2O, 25°C) of Tetrodotoxin (**C**, Buy from Tocris bioscience)

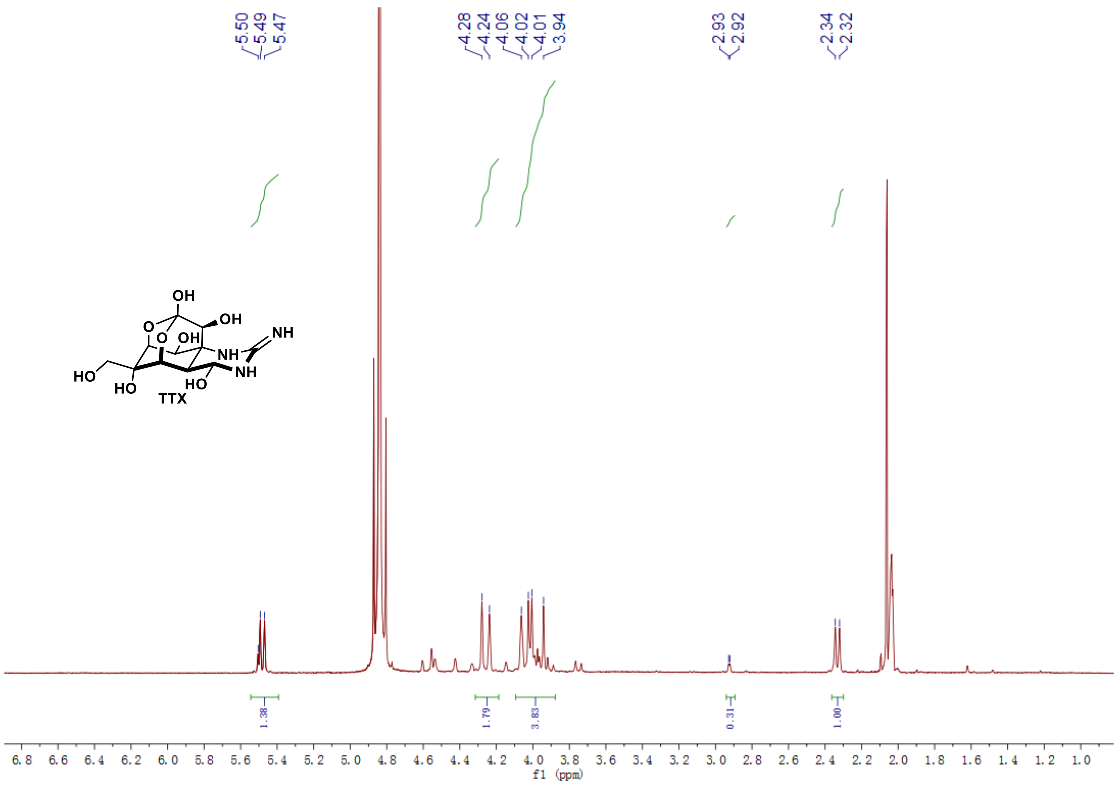

Supplementary Figure 33. NMR spectra of compound 1 Tetrodotoxin, (**C**, Buy from Tocris bioscience)

^{1}H NMR (400 MHz, $CDCl_3$, 25°C) of compound **23a**

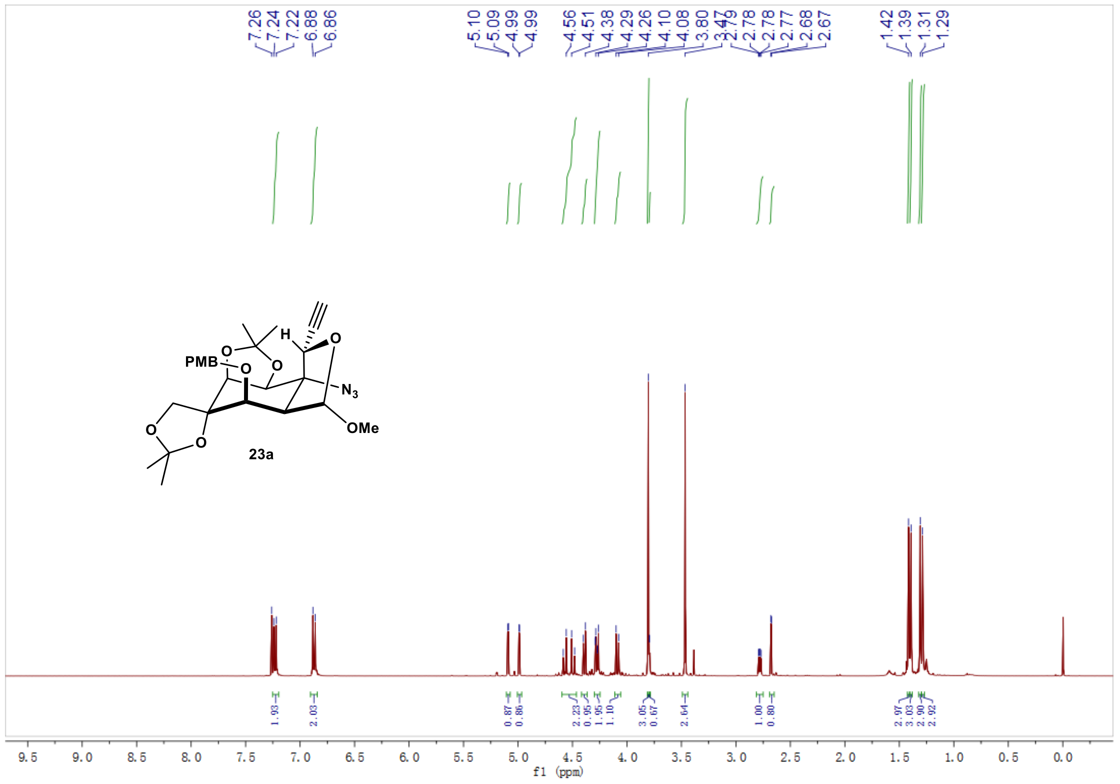

^{13}C NMR (101 MHz, $CDCl_3$, 25°C) of compound **23a**

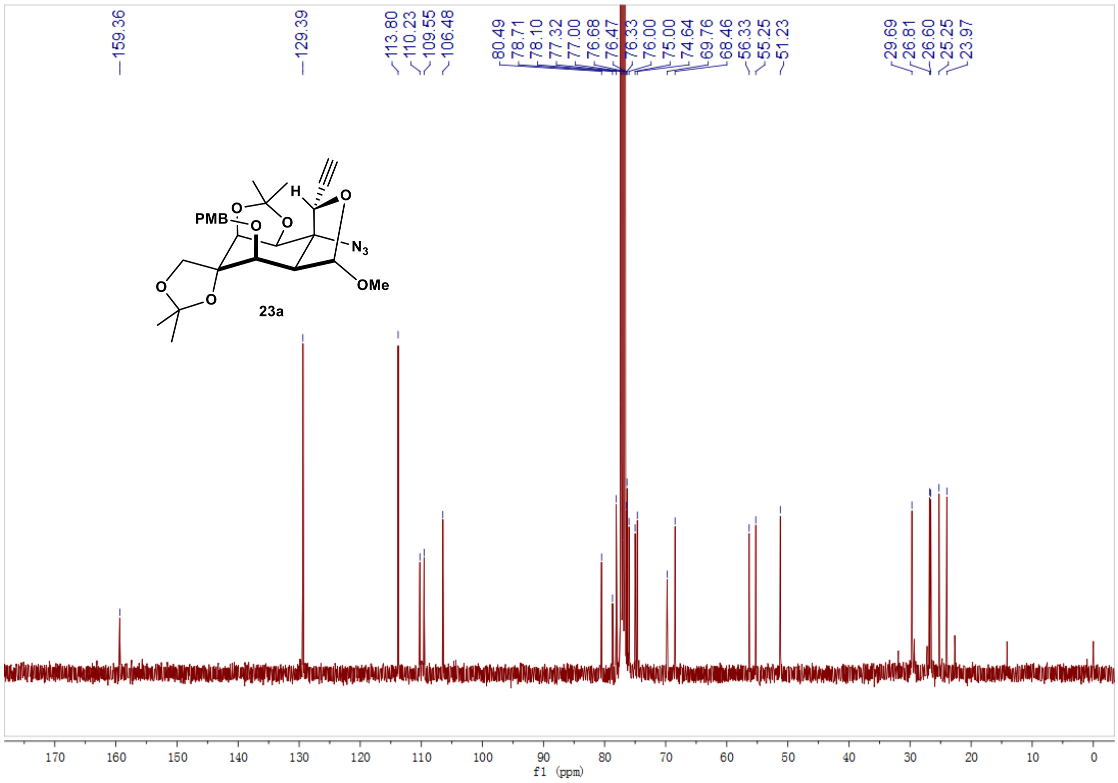

COSY of compound **23a**

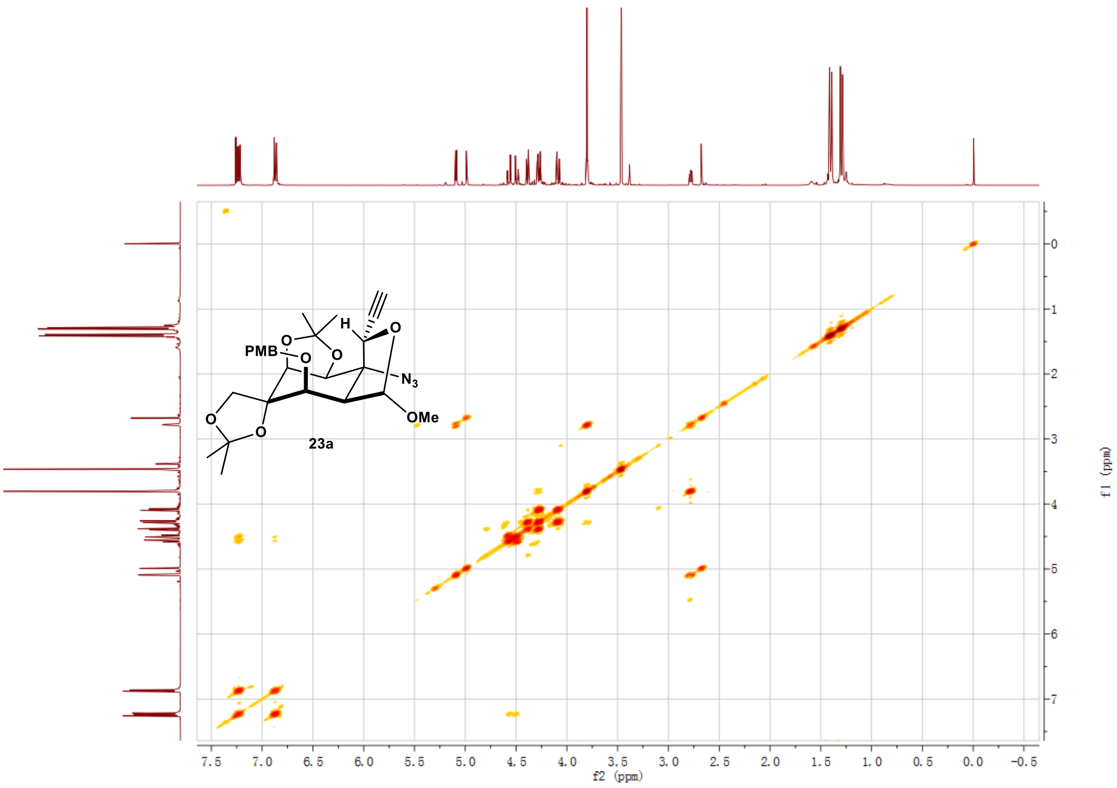

HSQC of compound **23a**

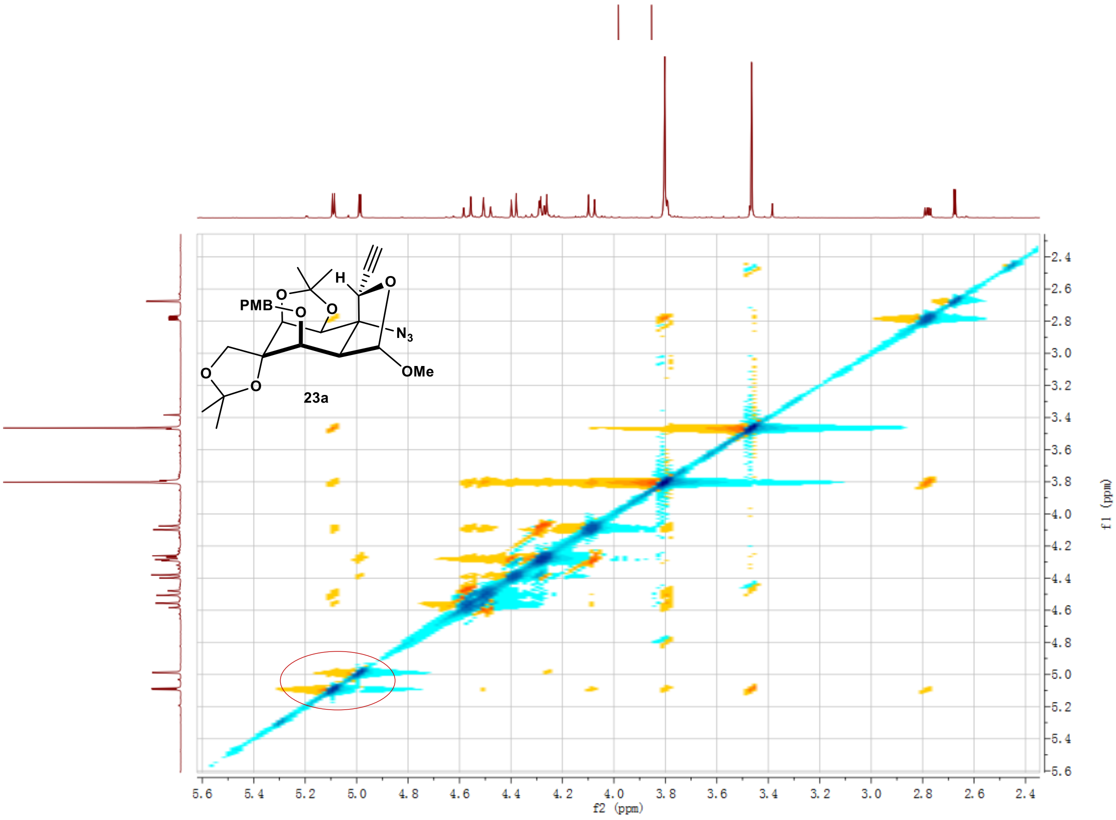

NOESY of compound **23a**

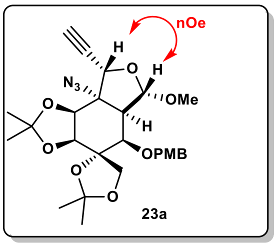

Supplementary Figure 34. NMR spectra of compound 23a

^{1}H NMR (400 MHz, $CDCl_3$, 25°C) of compound **24a**

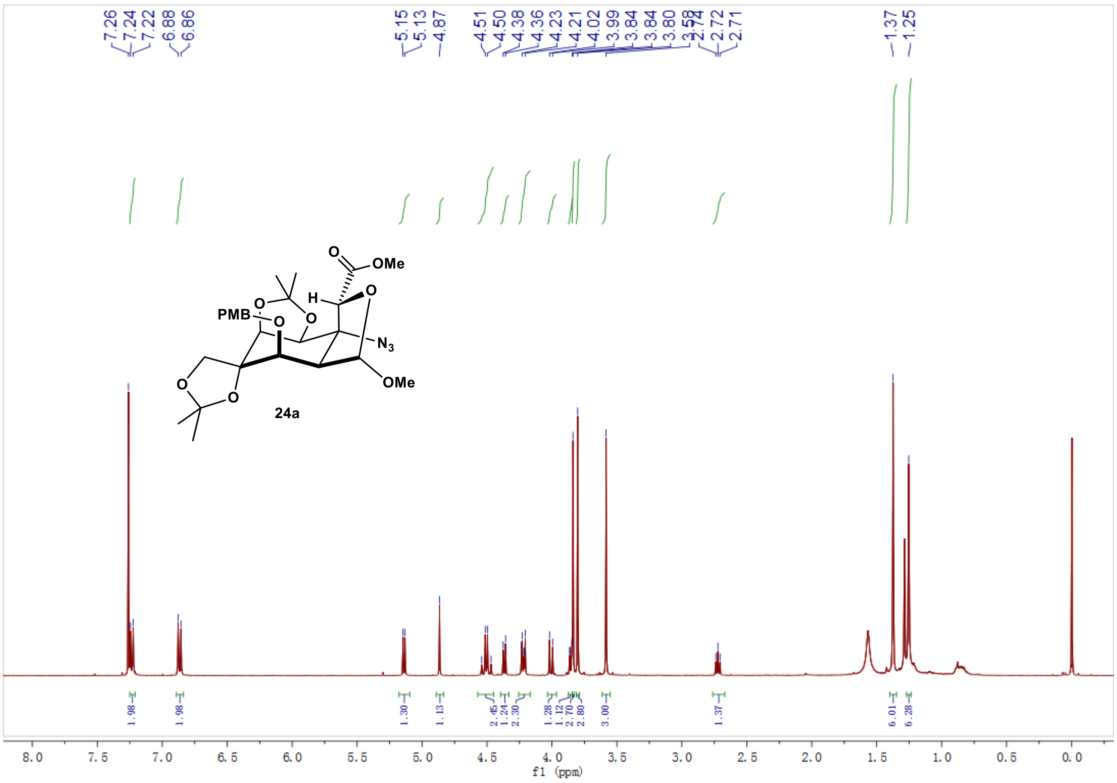

^{13}C NMR (101 MHz, $CDCl_3$, 25°C) of compound **24a**

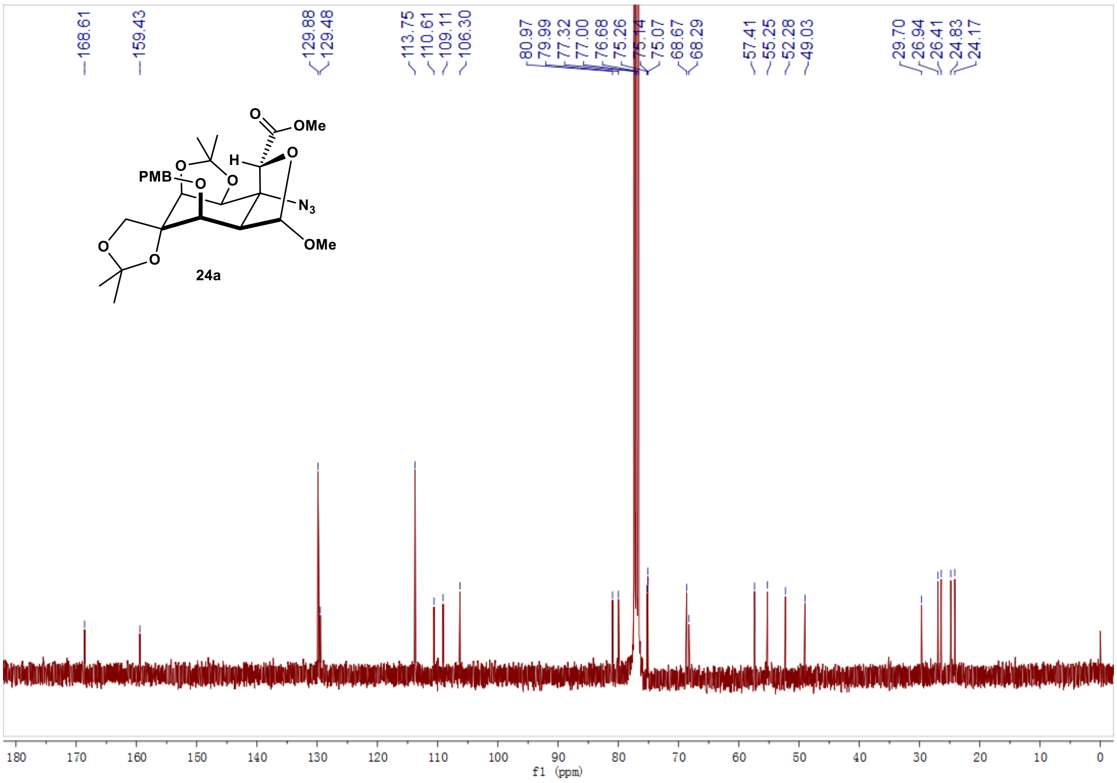

Supplementary Figure 35. NMR spectra of compound 24a

^{1}H NMR (400 MHz, $CDCl_3$, 25°C) of compound **25a**

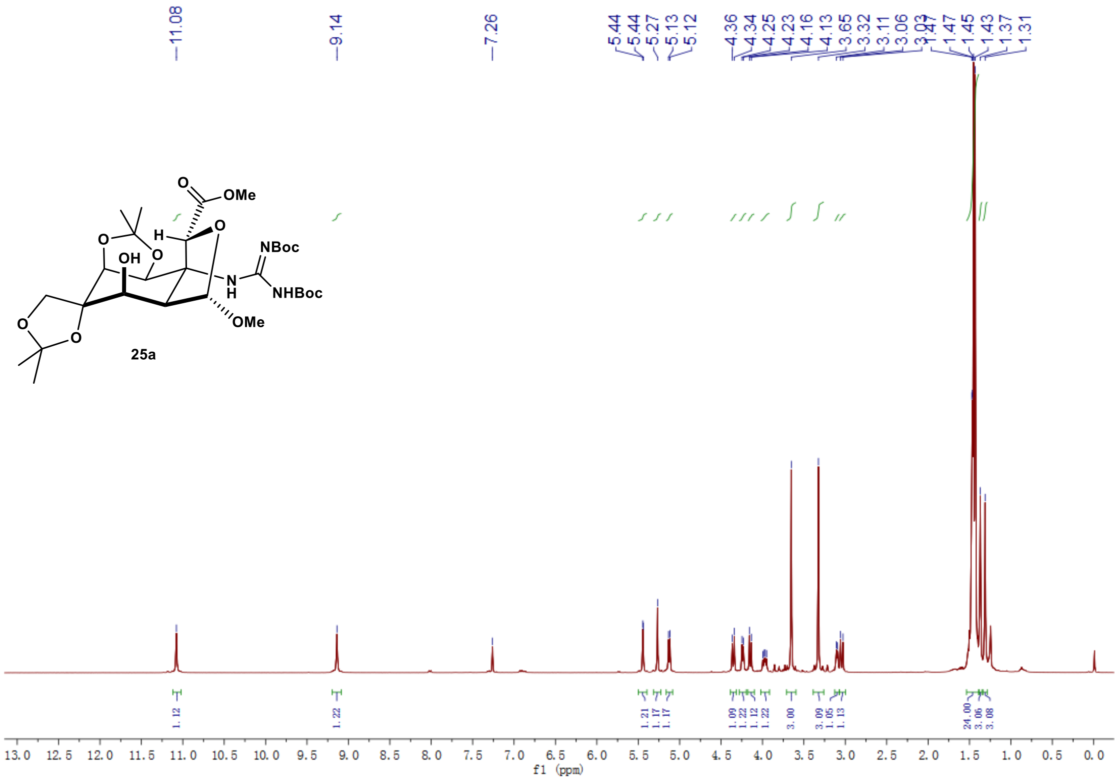

^{13}C NMR (101 MHz, $CDCl_3$, 25°C) of compound **25a**

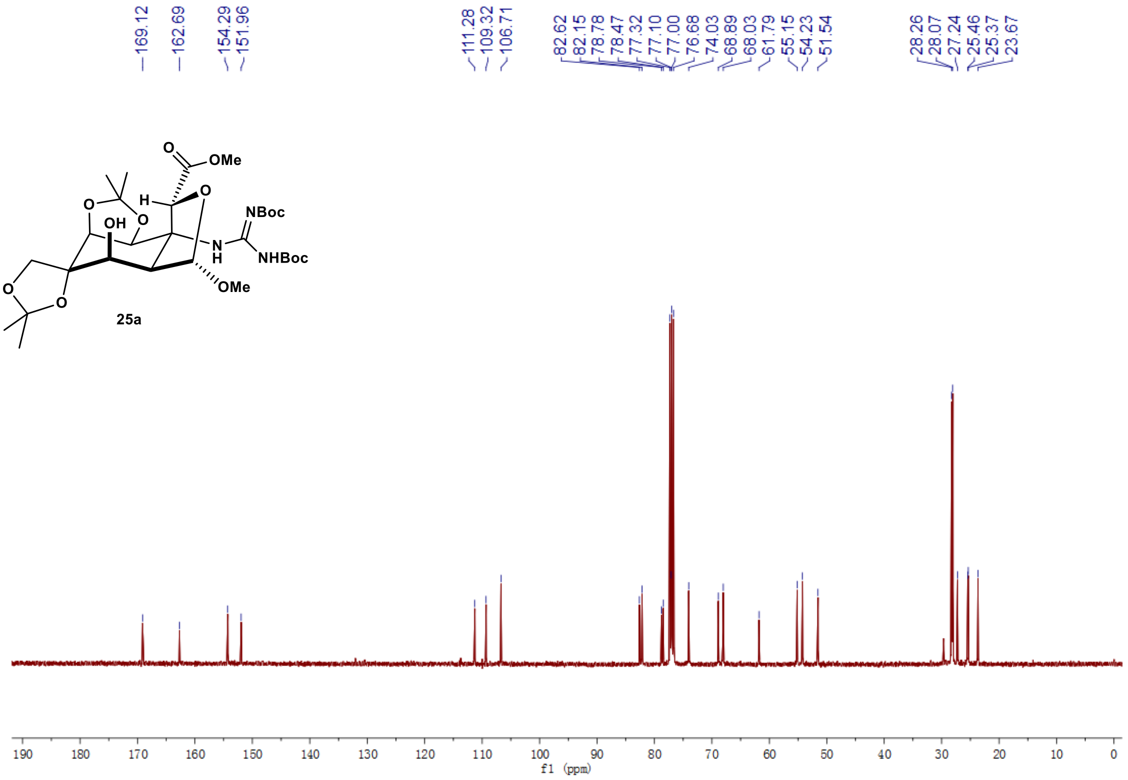

Supplementary Figure 36. NMR spectra of compound 25a

^{1}H NMR (400 MHz, 5% CD_3CO_2D/D_2O, 25°C) of compound 9-*epi*TTX **1a** and 9-*epi*TTX-8,10-lactone **1c**

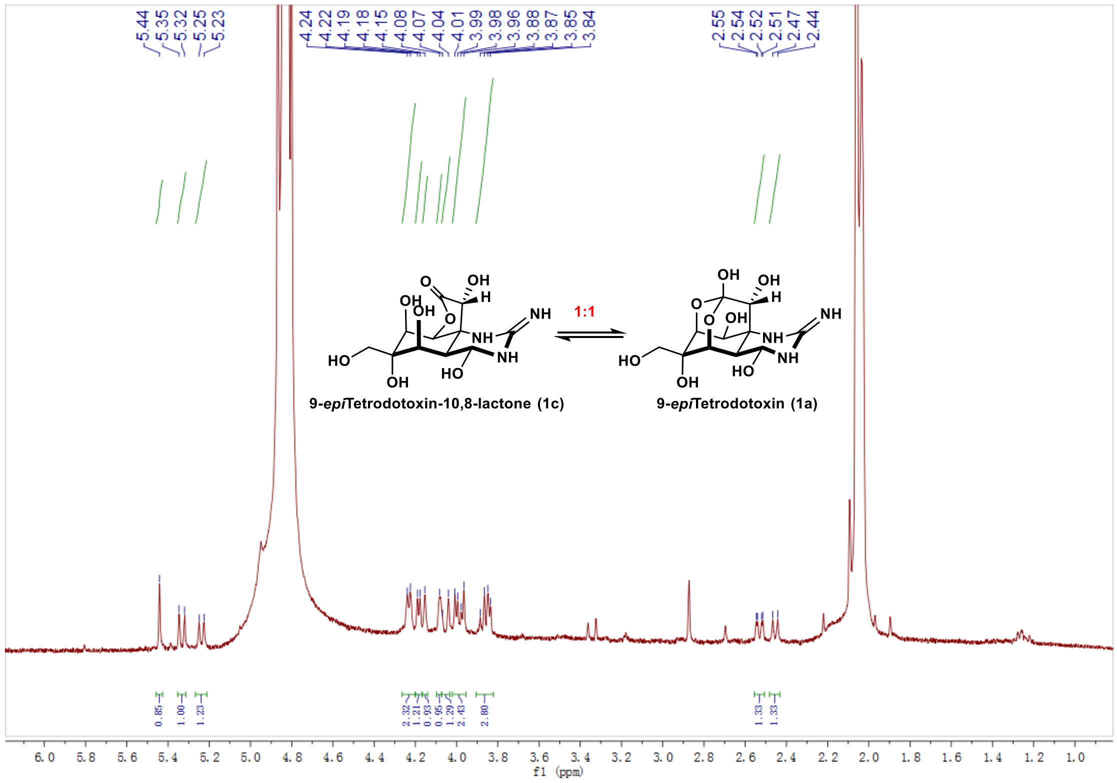

^{13}C NMR (151 MHz, 5% CD_3CO_2D/D_2O, 25°C) of compound 9-*epi*TTX **1a** and 9-*epi*TTX-8,10-lactone **1c**

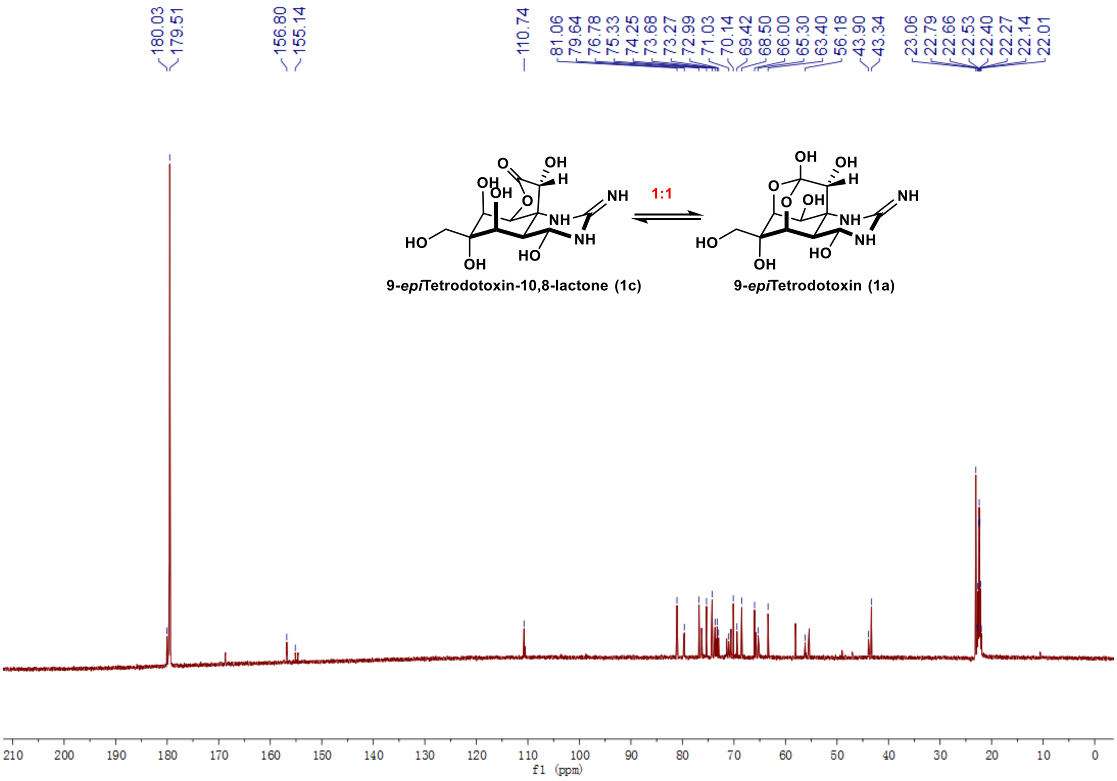

Supplementary Figure 37. NMR spectra of compound 9-*epi*TTX **1a** and 9-*epi*TTX-8,10-lactone **1c**

HPLC spectra

Synthetic TTX

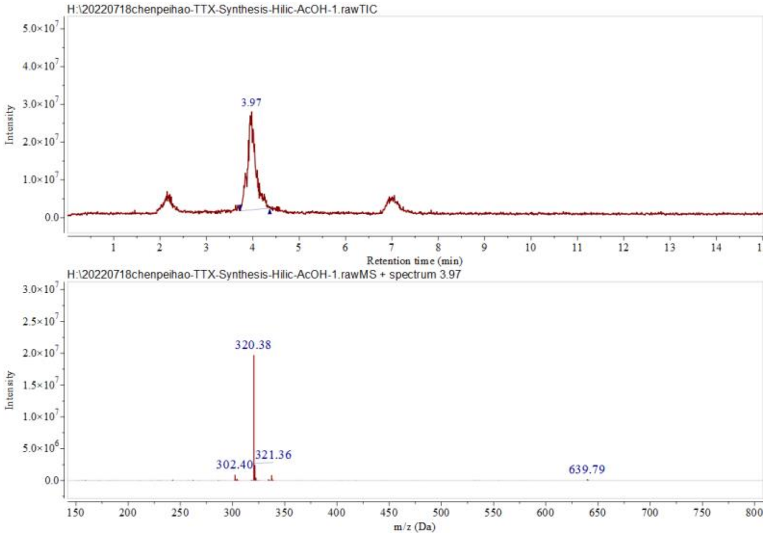

Supplementary Figure 38. HPLC spectra of Synthetic TTX

Commercial TTX (Buy from Tocris bioscience)

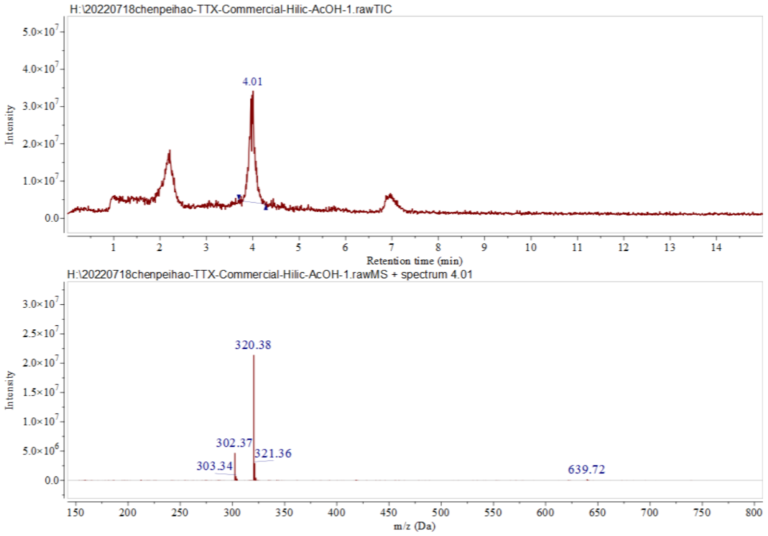

Supplementary Figure 39. HPLC spectra of Commercial TTX (Buy from Tocris bioscience)

Synthetic 9-*epi*TTX

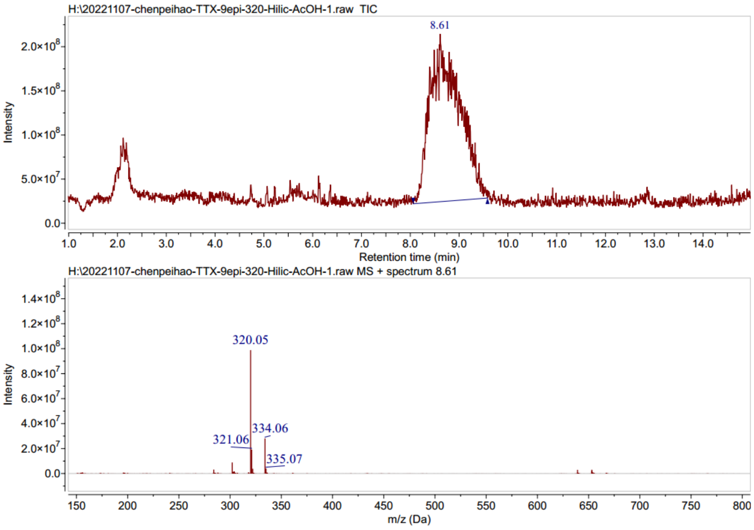

Supplementary Figure 40. HPLC spectra of Synthetic 9-*epi*TTX

Acquisition parameters:

Column Atlanits® HILIC Silica, 5 µm, 4.6*150 mm) with 2998PDA and 3100MS detectors

Mobile phase: H_2O (0.1% AcOH)/ Acetonitrile (0.1% AcOH) (65%-50% in 10 min).

4. Computational details

In our calculations, Gaussian 16 program[8] was used to carry out total density functional theory (DFT) calculations. Geometry optimizations were performed in gas phase by B3LYP[9] hybrid functional with dispersion correction of D3[10](BJ)[11] and using Def2-SVP[12,13] basis set for all elements. The vibrational frequencies calculations were conducted at the same level of theory to be sure whether every optimized stationary point is an energy minimum or a transition state and evaluate their thermal corrections at 298 K. The single point energies were calculated based on the gas-phase optimized structures by M06-2x[14] hybrid functional with D3[10] dispersion correction and def2-TZVPP[12,13] basis set was used in calculations. The solvation free energies were evaluated by SMD implicit solvent model[15] in single point calculations. In order to adjust the Gibbs free energies from 1 atm to 1 mol/L, a correction of $RT\ln(Csol/Cgas)$ (1.89 kcal/mol) is added to energies of all species. Csol represents the standard molar concentration in solution (1 mol/L), Cgas represents the standard molar concentration in gas phase (0.0446 mol/L), and *R* represents the gas constant.

	ZPE	TCH	TCG	EE	H	G	Im. Freq.
**13**	0.492018	0.523054	0.428490	-1532.809591	-1532.286537	-1532.378091	
**TEMPO**	0.261405	0.274203	0.224938	-483.691997	-483.417794	-483.464049	
**Cis_TS**	0.757973	0.800627	0.685693	-2016.498049	-2015.697422	-2015.809346	158.73***i***
**Trans_TS**	0.756439	0.799563	0.682964	-2016.500741	-2015.701178	-2015.814767	118.62***i***
**14-r**	0.762183	0.804493	0.690386	-2016.577852	-2015.773359	-2015.884456	
**14**	0.761967	0.804464	0.688249	-2016.579619	-2015.775155	-2015.888360	

Supplementary Table 6: Zero-point correction (ZPE), thermal correction to enthalpy (TCH), thermal correction to Gibbs free energy (TCG), electronic energies (EE), enthalpies (H), and Gibbs free energies(G) (in Hartree) of the structures calculated in corresponding level.

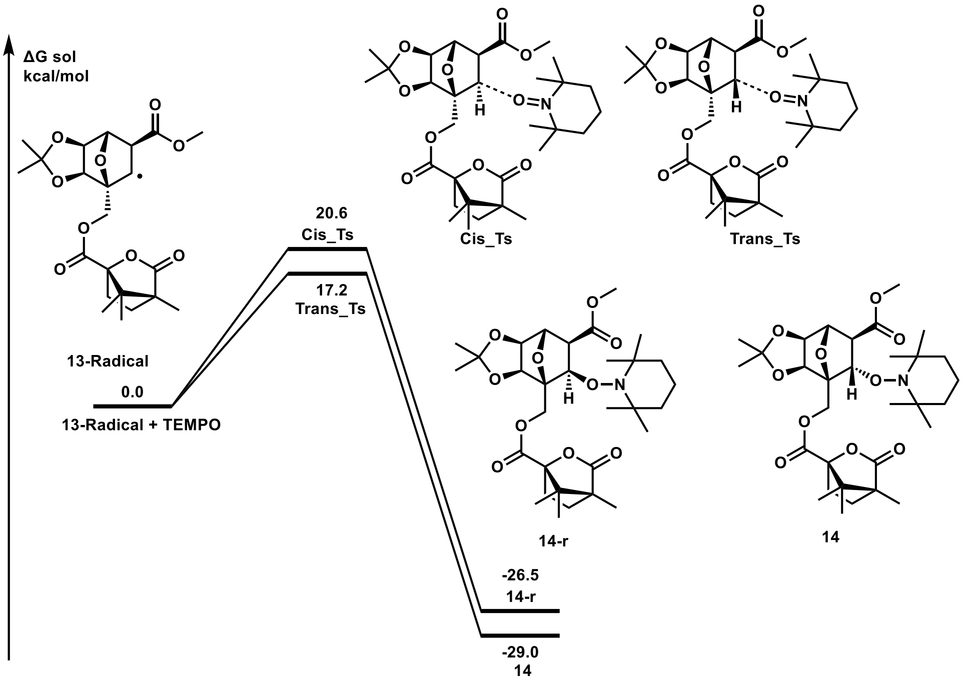

Supplementary Figure 41. Free energy diagram of radical coupling process. The DFT calculations support a clear radical addition preference for the experimentally observed stereoisomer at C5 that stems from the radical addition from the convex face of the oxo-bridge ring. (ΔΔG=3.4 kcal/mol and predicted dr > 99:1).

5. Crystallographic information

The crystal data of compound **(+)-13** has been deposited in CCDC with number **2184304**.

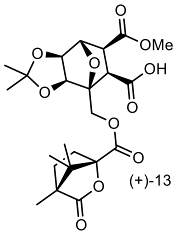

(+)-13

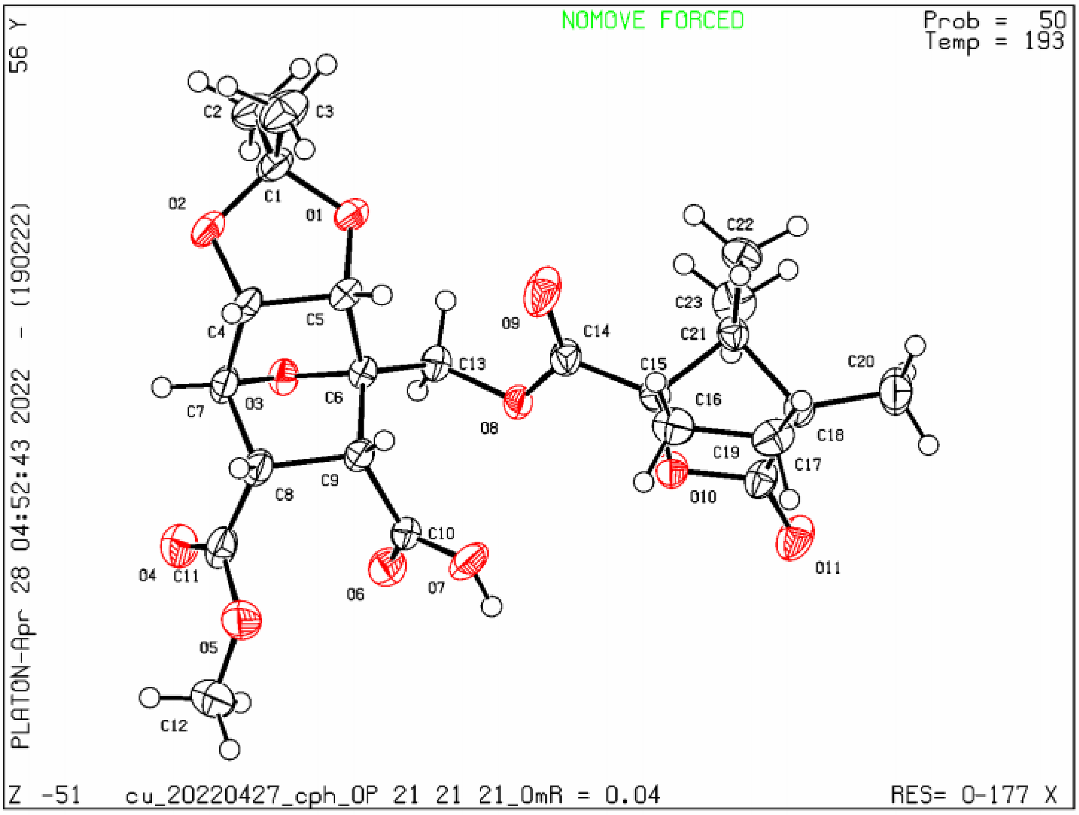

Supplementary Figure 42. X-ray crystal structure of compound 13

Bond precision: C-C = 0.0039 A		Wavelength=1.54178
Cell:	a=6.0147(1) b=17.5833(3) c=23.3786(4)	
	alpha=90 beta=90 gamma=90	
Temperature:	193 K	
	Calculated	Reported
Volume	2472.48(7)	2472.48(7)
Space group	P 21 21 21	P 21 21 21
Hall group	P 2ac 2ab	P 2ac 2ab
Moiety formula	C23 H30 O11 [+ solvent]	C23 H30 O11
Sum formula	C23 H30 O11 [+ solvent]	C23 H30 O11
Mr	482.47	482.47

Dx,g cm-3	1.296	1.296
Z	4	4
Mu (mm-1)	0.879	0.879
F000	1024.0	1024.0
F000'	1027.73	
h,k,lmax	7,21,28	7,21,28
Nref	4525[2624]	4521
Tmin,Tmax	0.869,0.892	0.353,0.467
Tmin'	0.869	
Correction method= # Reported T Limits: Tmin=0.353 Tmax=0.467		
AbsCorr = MULTI-SCAN		
Data completeness= 1.72/1.00	Theta(max)= 68.172	
R(reflections)= 0.0421(4310)	wR2(reflections)=0.1130(4521)	
S = 1.118	Npar= 315	

Supplementary Table 7. Crystal data and structure refinement for compound 13.

The crystal data of compound **(+)-16** has been deposited in CCDC with number **2182018**.

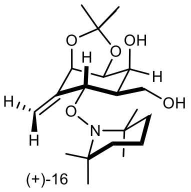

(+)-16

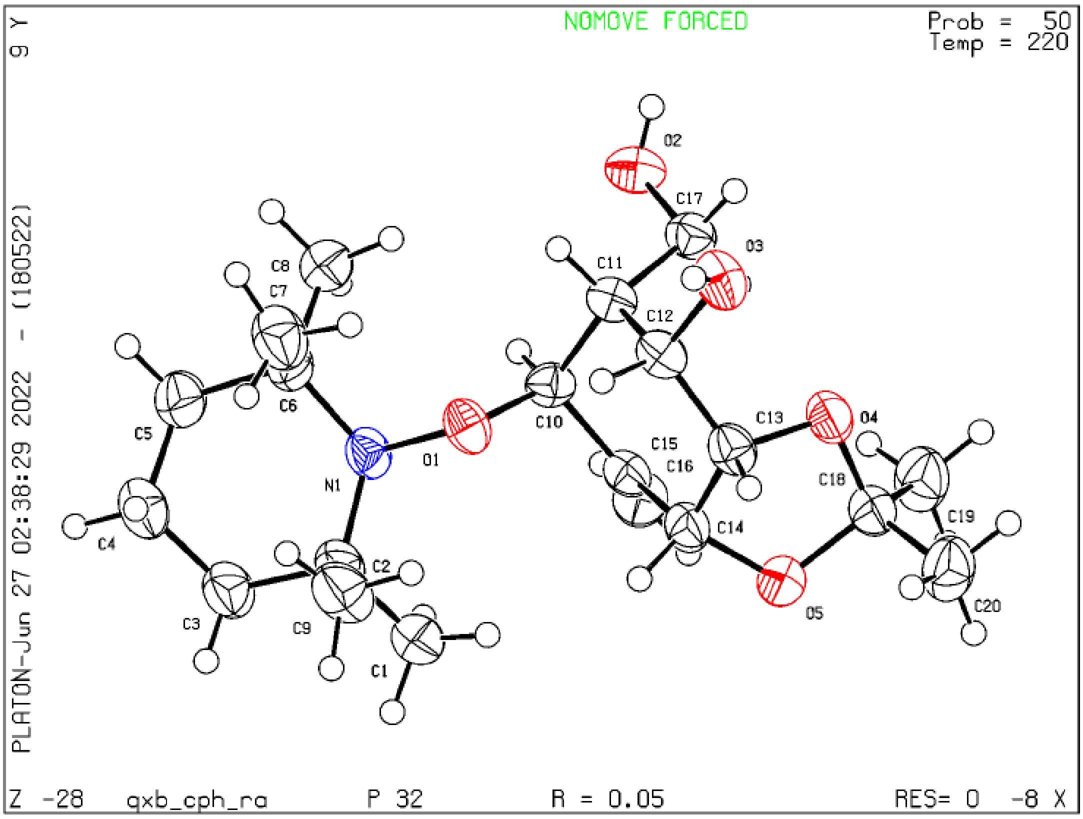

Supplementary Figure 43. X-ray crystal structure of compound 16

Note: Alert level B

PLAT029_ALERT_3_B _diffrn_measured_fraction_theta_full value Low . 0.954 Why?

The current B level ALERTS might due to the incomplete scans, possibly based on erroneously assumed higher than actual symmetry.

Bond precision: C-C = 0.0074 A		Wavelength=1.54184
Cell:	a=17.6413(14) b=17.6413(14) c=6.2712(6)	
	alpha=90 beta=90 gamma=120	
Temperature:	220K	
	Calculated	Reported
Volume	1690.2(3)	1690.2(3)
Space group	P 32	P 32
Hall group	P 32	P 32
Moiety formula	C20 H35 N O5 [+ solvent]	C20 H35 N O5
Sum formula	C20 H35 N O5 [+ solvent]	C20 H35 N O5
Mr	369.49	369.49
Dx,g cm-3	1.089	1.089
Z	3	3
Mu (mm-1)	0.624	0.624
F000	606.0	606.0
F000’	607.84	
h,k,lmax	21,21,7	21,21,7
Nref	4468[2234]	3347
Tmin,Tmax	0.963,0.969	0.479,1.000
Tmin’	0.940	
Correction method= # Reported T Limits: Tmin=0.479 Tmax=1.000		
AbsCorr = MULTI-SCAN		
Data completeness= 1.50/0.75	Theta(max)= 72.548	
R(reflections)= 0.0475(2416)	wR2(reflections)=0.1205(3347)	
S = 0.946	Npar= 243	

Supplementary Table 8. Crystal data and structure refinement for compound 16.

The crystal data of compound **(-)-21** has been deposited in CCDC with number **2184298**.

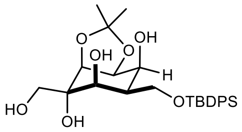

(-)-21

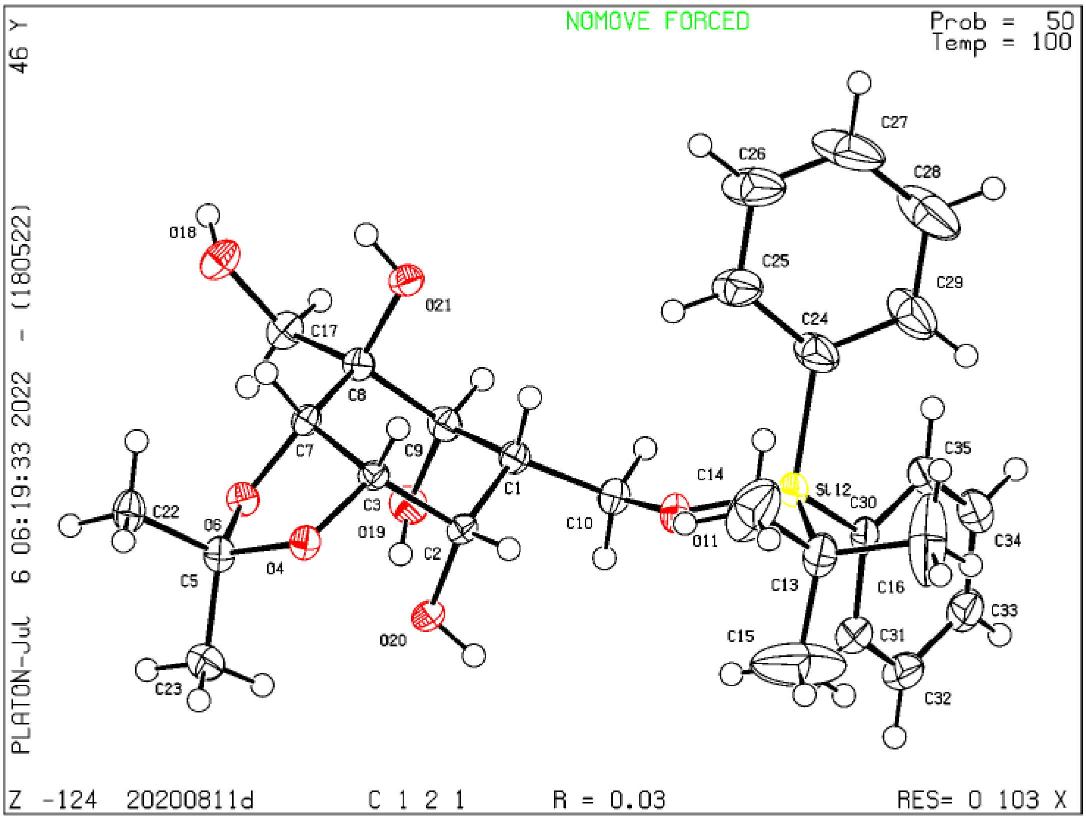

Supplementary Figure 44. X-ray crystal structure of compound 21

Bond precision: C-C = 0.0039 A		Wavelength=1.54184
Cell:	a=21.9882(2) b=7.80324(9) c=16.22556(19)	
	alpha=90 beta=91.3178(11) gamma=90	
Temperature:	100K	
	Calculated	Reported
Volume	2783.23(5)	2783.23(5)
Space group	C 2	C 1 2 1
Hall group	C 2y	C 2y
Moiety formula	C27 H38 O7 Si [+ solvent]	C27 H38 O7 Si
Sum formula	C27 H38 O7 Si [+ solvent]	C27 H38 O7 Si
Mr	502.66	502.66
Dx,g cm-3	1.200	1.200
Z	4	4
Mu (mm-1)	1.085	1.085
F000	1080.0	1080.0
F000’	1084.23	

h,k,lmax	27,9,20	27,9,20
Nref	5791[3116]	5558
Tmin,Tmax	0.878,0.979	0.923,1.000
Tmin'	0.805	
Correction method= # Reported T Limits: Tmin=0.923 Tmax=1.000		
AbsCorr = MULTI-SCAN		
Data completeness= 1.78/0.96	Theta(max)= 75.646	
R(reflections)= 0.0330(5430)	wR2(reflections)=0.0821(5558)	
S = 1.057	Npar= 326	

Supplementary Table 9. Crystal data and structure refinement for compound 21.

The crystal data of compound **(+)-23** has been deposited in CCDC with number **2184305**.

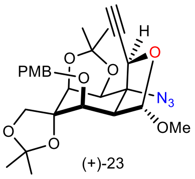

(+)-23

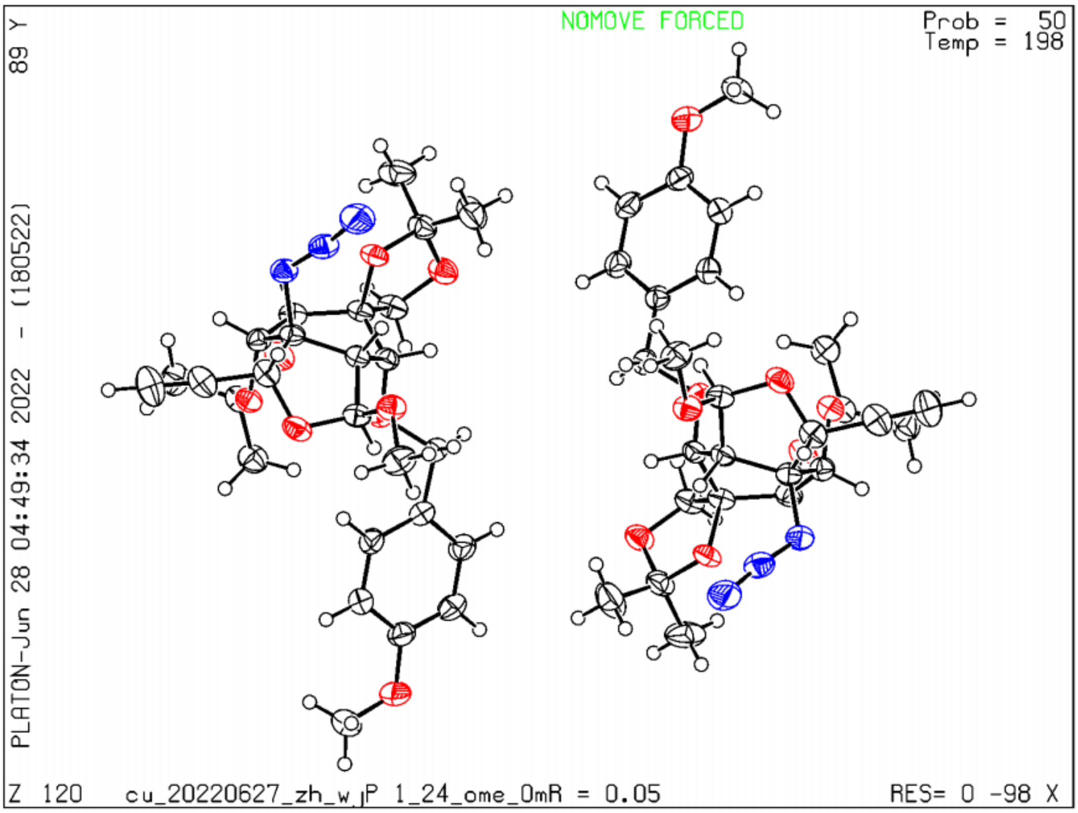

Supplementary Figure 45. X-ray crystal structure of compound 23

Bond precision: C-C = 0.0070 A		Wavelength=1.54178
Cell:	a=8.3171(2) b=10.4531(3) c=16.3414(5)	
	alpha=84.815(2) beta=83.036(2) gamma=67.740(1)	
Temperature:	198K	
	Calculated	Reported
Volume	1303.61(6)	1303.61(6)

Space group	P 1	P 1
Hall group	P 1	P 1
Moiety formula	C26 H33 N3 O8	C26 H33 N3 O8
Sum formula	C26 H33 N3 O8	C26 H33 N3 O8
Mr	515.55	515.55
Dx,g cm-3	1.313	1.313
Z	2	2
Mu (mm-1)	0.815	0.815
F000	548.0	548.0
F000’	549.83	
h,k,lmax	10,12,19	10,12,19
Nref	9580[4790]	28420
Tmin,Tmax	0.889,0.922	0.662,0.753
Tmin’	0.885	
Correction method= # Reported T Limits: Tmin=0.662 Tmax=0.753		
AbsCorr = MULTI-SCAN		
Data completeness= 5.93/2.97	Theta(max)= 68.383	
R(reflections)= 0.0476(24581)	wR2(reflections)=0.1245(28420)	
S = 1.039	Npar= 680	

Supplementary Table10. Crystal data and structure refinement for compound 23.

4. Supplementary References

1 Yasumoto, T., Yotsu, M., Murata, M. & Naoki, H. New tetrodotoxin analogs from the newt Cynops ensicauda. *J. Am. Chem. Soc.* **110**, 2344-2345 (1988).

2 Yaegashi, Y. *et al.* Isolation and Biological Activity of 9-*epi*Tetrodotoxin and Isolation of Tb-242B, Possible Biosynthetic Shunt Products of Tetrodotoxin from Pufferfish. *J. Nat. Prod.* **85**, 2199-2206 (2022).

3 Sadasivam, D. V., Choquette, K. A. & Flowers, R. A. n. Preparation and Use of Samarium Diiodide (SmI_2) in Organic Synthesis: The Mechanistic Role of HMPA and Ni(II) Salts in the Samarium Barbier Reaction. *J Vis Exp.* **72**, e4323 (2013).

4 Maehara, T., Motoyama, K., Toma, T., Yokoshima, S. & Fukuyama, T. Total Synthesis of (−)-Tetrodotoxin and 11-norTTX-6(*R*)-ol. *Angew. Chem. Int. Ed.* **56**, 1549-1552 (2017).

5 Cao, P., Yang, X. & Sudhof, T. C. Complexin activates exocytosis of distinct secretory vesicles controlled by different synaptotagmins. *The Journal of neuroscience : the official journal of the Society for Neuroscience* **33**, 1714-1727 (2013).

6 Roseberry, T.K. et al. Cell-Type-Specific Control of Brainstem Locomotor Circuits by Basal Ganglia. Cell **164**, 526-537 (2016).

7 Liu, Z. *et al.* IGF1-Dependent Synaptic Plasticity of Mitral Cells in Olfactory Memory during Social Learning. *Neuron* **95**, 106-122.e105 (2017).

8 Gaussian 16 Rev. C.01 (Wallingford, CT, 2016).

9 Stephens, P. J., Devlin, F. J., Chabalowski, C. F. & Frisch, M. J. Ab Initio Calculation of Vibrational Absorption and Circular Dichroism Spectra Using Density Functional Force Fields. *The Journal of Physical Chemistry* **98**, 11623-11627 (1994).

10 Grimme, S., Antony, J., Ehrlich, S. & Krieg, H. A consistent and accurate ab initio parametrization of density functional dispersion correction (DFT-D) for the 94 elements H-Pu. *J Chem Phys* **132**, 154104 (2010).

11 Grimme, S., Ehrlich, S. & Goerigk, L. Effect of the damping function in dispersion corrected density functional theory. *J Comput Chem* **32**, 1456-1465 (2011).

12 Weigend, F. & Ahlrichs, R. Balanced basis sets of split valence, triple zeta valence and quadruple zeta valence quality for H to Rn: Design and assessment of accuracy. *Phys Chem Chem Phys* **7**, 3297-3305 (2005).

13 Andrae, D., Häußermann, U., Dolg, M., Stoll, H. & Preuß, H. Energy-adjustedab initio pseudopotentials for the second and third row transition elements. *Theoretica chimica acta* **77**, 123-141 (1990).

14 Zhao, Y. & Truhlar, D. G. The M06 suite of density functionals for main group thermochemistry, thermochemical kinetics, noncovalent interactions, excited states, and transition elements: two new functionals and systematic testing of four M06-class functionals and 12 other functionals. *Theoretical Chemistry Accounts* **120**, 215-241 (2008).

15 Marenich, A. V., Cramer, C. J. & Truhlar, D. G. Universal solvation model based on solute electron density and on a continuum model of the solvent defined by the bulk dielectric constant and atomic surface tensions. *J Phys Chem B* **113**, 6378-6396 (2009).
