# Supplementary material for: Total syntheses of Tetrodotoxin and 9-epiTetrodotoxin
Source: Nat Commun. 2024 Jan 23;15:679. doi: 10.1038/s41467-024-45037-0 (PMC10806222; doi:10.1038/s41467-024-45037-0)
Supplement: Supplementary file 3 — Reporting Summary [file 41467_2024_45037_MOESM3_ESM.pdf]

nature portfolio

Corresponding author(s): XIANGBING QI

Last updated by author(s): Dec 22, 2023

Reporting Summary

Nature Portfolio wishes to improve the reproducibility of the work that we publish. This form provides structure for consistency and transparency in reporting. For further information on Nature Portfolio policies, see our Editorial Policies and the Editorial Policy Checklist.

Statistics

For all statistical analyses, confirm that the following items are present in the figure legend, table legend, main text, or Methods section.

n/a	Confirmed	
☐	☒	The exact sample size (*n*) for each experimental group/condition, given as a discrete number and unit of measurement
☐	☒	A statement on whether measurements were taken from distinct samples or whether the same sample was measured repeatedly
☐	☒	The statistical test(s) used AND whether they are one- or two-sided<br>*Only common tests should be described solely by name; describe more complex techniques in the Methods section.*
☒	☐	A description of all covariates tested
☒	☐	A description of any assumptions or corrections, such as tests of normality and adjustment for multiple comparisons
☐	☒	A full description of the statistical parameters including central tendency (e.g. means) or other basic estimates (e.g. regression coefficient) AND variation (e.g. standard deviation) or associated estimates of uncertainty (e.g. confidence intervals)
☐	☒	For null hypothesis testing, the test statistic (e.g. *F*, *t*, *r*) with confidence intervals, effect sizes, degrees of freedom and *P* value noted<br>*Give P values as exact values whenever suitable.*
☒	☐	For Bayesian analysis, information on the choice of priors and Markov chain Monte Carlo settings
☒	☐	For hierarchical and complex designs, identification of the appropriate level for tests and full reporting of outcomes
☒	☐	Estimates of effect sizes (e.g. Cohen's *d*, Pearson's *r*), indicating how they were calculated

Our web collection on statistics for biologists contains articles on many of the points above.

Software and code

Policy information about availability of computer code

Data collection	We used MultiClamp 700B, Clampex 10.5 data acquisition software (Molecular Devices) for electrophysiological data acquisition.
Data analysis	The electrophysiological data were analyzed using Clampfit 10.5 (software) and Prism 6.02 (GraphPad Software). SigmaPlot 12.0 software were used for curve fitting and calculation of IC50.

For manuscripts utilizing custom algorithms or software that are central to the research but not yet described in published literature, software must be made available to editors and reviewers. We strongly encourage code deposition in a community repository (e.g. GitHub). See the Nature Portfolio guidelines for submitting code & software for further information.

Data

Policy information about availability of data

All manuscripts must include a data availability statement. This statement should provide the following information, where applicable:

- Accession codes, unique identifiers, or web links for publicly available datasets
- A description of any restrictions on data availability
- For clinical datasets or third party data, please ensure that the statement adheres to our policy

The X-ray crystallographic coordinates for structures reported in this study have been deposited at the Cambridge Crystallographic Data Centre (CCDC), under deposition numbers 2184304 (13) (https://dx.doi.org/10.5517/ccdc.csd.cc2c9yf2), 2182018 (16) (https://dx.doi.org/10.5517/ccdc.csd.cc2c7kpw), 2184298 (21) (https://dx.doi.org/10.5517/ccdc.csd.cc2c9y7w), 2184305 (23) (https://dx.doi.org/10.5517/ccdc.csd.cc2c9yg3). Copies of the data can be obtained free of charge via https://www.ccdc.cam.ac.uk/structures/. All other data supporting the findings of this study, including experimental procedures and compound characterization,

NMR, and HPLC are available within the Article and its Supplementary Information or from the corresponding author upon request. Source data are provided with this paper. The raw NMR data and HPLC traces, optimized coordination data for the calculated structures, data of electrophysiological experiments are available at figshare under accession code https://doi.org/10.6084/m9.figshare.23291687 under the Creative Commons Attribution 4.0 International license.

Research involving human participants, their data, or biological material

Policy information about studies with human participants or human data. See also policy information about sex, gender (identity/presentation), and sexual orientation and race, ethnicity and racism.

Reporting on sex and gender	This information has not been collected.
Reporting on race, ethnicity, or other socially relevant groupings	This information has not been collected.
Population characteristics	This information has not been collected.
Recruitment	This information has not been collected.
Ethics oversight	This information has not been collected.

Note that full information on the approval of the study protocol must also be provided in the manuscript.

Field-specific reporting

Please select the one below that is the best fit for your research. If you are not sure, read the appropriate sections before making your selection.

[x] Life sciences [ ] Behavioural & social sciences [ ] Ecological, evolutionary & environmental sciences

For a reference copy of the document with all sections, see nature.com/documents/nr-reporting-summary-flat.pdf

Life sciences study design

All studies must disclose on these points even when the disclosure is negative.

Sample size	Primary hippocampal neurons<br>0 nM TTX group, N = 24; 10 nM TTX (S) group, N = 7; 10 nM TTX (C) group, N = 8; 50 nM TTX (S) group, N = 9; 50 nM TTX (C) group, N = 9; 100 nM TTX (S) group, N = 8; 100 nM TTX (C) group, N = 11; 1000 nM TTX (S) group, N = 5; 1000 nM TTX (C) group, N = 5.<br>HEK-Nav1.5 assay<br>0 nM TTX group, N = 24; 1 μM TTX (S) group, N = 16; 1 μM TTX (C) group, N = 16.<br>HEK-Nav1.7 assay<br>0 nM TTX group, N = 28; 2.5 nM TTX (S) group, N = 19; 2.5 nM TTX (C) group, N = 22; 5 nM TTX (S) group, N = 22; 5 nM TTX (C) group, N = 21; 10 nM TTX (S) group, N = 19; 10 nM TTX (C) group, N = 20; 100 nM TTX (S) group, N = 15; 100 nM TTX (C) group, N = 18.<br>No statistical methods were used to pre-determine sample size. We used sample sizes similar to literature in the field.
Data exclusions	For quality control, electrophysiological recordings with series resistances of > 15 MΩ were rejected. Leakage currents of > 400 pA were rejected.
Replication	Experiments that led to quantitative conclusions were repeated (as indicated in figure legends).
Randomization	Samples were allocated random.
Blinding	Data collection and analysis was not performed blind. Due to the limitation of the number of electrophysiological researchers.

Reporting for specific materials, systems and methods

We require information from authors about some types of materials, experimental systems and methods used in many studies. Here, indicate whether each material, system or method listed is relevant to your study. If you are not sure if a list item applies to your research, read the appropriate section before selecting a response.

Materials & experimental systems

n/a	Involved in the study
☒	☐ Antibodies
☐	☒ Eukaryotic cell lines
☒	☐ Palaeontology and archaeology
☐	☒ Animals and other organisms
☒	☐ Clinical data
☒	☐ Dual use research of concern
☒	☐ Plants

Methods

n/a	Involved in the study
☒	☐ ChIP-seq
☒	☐ Flow cytometry
☒	☐ MRI-based neuroimaging

Eukaryotic cell lines

Policy information about cell lines and Sex and Gender in Research

Cell line source(s)	HEK-293 cells stably expressing Nav 1.5 (human) or Nav 1.7 (human) were donated by Fan Zhang lab in Hebei Medical University
Authentication	These cell lines can be authenticated by RT-PCR.
Mycoplasma contamination	Cell lines used in this study were negative to mycoplasma by detection of PCR.
Commonly misidentified lines (See ICLAC register)	No commonly misidentified cell lines were used in this study.

Animals and other research organisms

Policy information about studies involving animals; ARRIVE guidelines recommended for reporting animal research, and Sex and Gender in Research

Laboratory animals	P0 C57BL/6J mice used for isolation of primary hippocampal neurons. C57BL/6J mice strain were maintained in an animal facility with 12h light/12h dark cycles, temperature (22-24 oC), humidity (40-60%) at the National Institute of Biological Sciences, Beijing
Wild animals	The study did not involve wild animals.
Reporting on sex	This information has not been collected.
Field-collected samples	The study did not involve samples collected from the field.
Ethics oversight	Animal experimentation : Animal care and use followed the institutional guidelines of the National Institute of Biological Sciences (NIBS), Beijing (Approval ID:NIBSLuoM15C), and the Regulations for the Administration of Affairs Concerning Experimental Animals of China.

Note that full information on the approval of the study protocol must also be provided in the manuscript.

Plants

Seed stocks	This information has not been collected.
Novel plant genotypes	This information has not been collected.
Authentication	This information has not been collected.
