# Supplementary material for: Total syntheses of Tetrodotoxin and 9-epiTetrodotoxin
Source: Nat Commun. 2024 Jan 23;15:679. doi: 10.1038/s41467-024-45037-0 (PMC10806222; doi:10.1038/s41467-024-45037-0)

# checkCIF/PLATON report

Structure factors have been supplied for datablock(s) qxb_cph_ra

THIS REPORT IS FOR GUIDANCE ONLY. IF USED AS PART OF A REVIEW PROCEDURE FOR PUBLICATION, IT SHOULD NOT REPLACE THE EXPERTISE OF AN EXPERIENCED CRYSTALLOGRAPHIC REFEREE.

No syntax errors found.  CIF dictionary  Interpreting this report

## Datablock: qxb_cph_ra

---

| | | | |
|---|---|---|---|
| Bond precision: | C-C = 0.0074 A | | Wavelength=1.54184 |
| Cell: | a=17.6413(14) | b=17.6413(14) | c=6.2712(6) |
| | alpha=90 | beta=90 | gamma=120 |
| Temperature: | 220 K | | |

| | Calculated | Reported |
|---|---|---|
| Volume | 1690.2(3) | 1690.2(3) |
| Space group | P 32 | P 32 |
| Hall group | P 32 | P 32 |
| Moiety formula | C20 H35 N O5 [+ solvent] | C20 H35 N O5 |
| Sum formula | C20 H35 N O5 [+ solvent] | C20 H35 N O5 |
| Mr | 369.49 | 369.49 |
| Dx,g cm-3 | 1.089 | 1.089 |
| Z | 3 | 3 |
| Mu (mm-1) | 0.624 | 0.624 |
| F000 | 606.0 | 606.0 |
| F000’ | 607.84 | |
| h,k,lmax | 21,21,7 | 21,21,7 |
| Nref | 4468[ 2234] | 3347 |
| Tmin,Tmax | 0.963,0.969 | 0.479,1.000 |
| Tmin’ | 0.940 | |

Correction method= # Reported T Limits: Tmin=0.479 Tmax=1.000
AbsCorr = MULTI-SCAN

Data completeness= 1.50/0.75  Theta(max)= 72.548

R(reflections)= 0.0475( 2416)  wR2(reflections)= 0.1205( 3347)

S = 0.946  Npar= 243

The following ALERTS were generated. Each ALERT has the format
**test-name_ALERT_alert-type_alert-level.**
Click on the hyperlinks for more details of the test.

### Alert level B

```
PLAT029_ALERT_3_B _diffrn_measured_fraction_theta_full value Low .      0.954 Why?
```

### Alert level C

```
STRVA01_ALERT_4_C           Flack parameter is too small
           From the CIF: _refine_ls_abs_structure_Flack   -0.300
           From the CIF: _refine_ls_abs_structure_Flack_su    0.300
PLAT340_ALERT_3_C Low Bond Precision on  C-C Bonds ...............    0.00744 Ang.
PLAT414_ALERT_2_C Short Intra D-H..H-X       H3       ..H12      .       1.99 Ang.
                                                   x,y,z  =      1_555 Check
PLAT417_ALERT_2_C Short Inter D-H..H-D       H2       ..H3       .       2.12 Ang.
                                                x,y,-1+z  =      1_554 Check
PLAT911_ALERT_3_C Missing FCF Refl Between Thmin & STh/L=    0.600          94 Report
PLAT915_ALERT_3_C No Flack x Check Done: Low Friedel Pair Coverage          57 %
```

### Alert level G

```
PLAT007_ALERT_5_G Number of Unrefined Donor-H Atoms ..............           2 Report
PLAT032_ALERT_4_G Std. Uncertainty on Flack Parameter Value High .       0.300 Report
PLAT398_ALERT_2_G Deviating  C-O-C   Angle From 120 for O4       .       106.6 Degree
PLAT398_ALERT_2_G Deviating  C-O-C   Angle From 120 for O5       .       108.1 Degree
PLAT605_ALERT_4_G Largest Solvent Accessible VOID in the Structure        217 A**3
PLAT791_ALERT_4_G Model has Chirality at C10         (Sohnke SpGr)           S Verify
PLAT791_ALERT_4_G Model has Chirality at C11         (Sohnke SpGr)           S Verify
PLAT791_ALERT_4_G Model has Chirality at C12         (Sohnke SpGr)           S Verify
PLAT791_ALERT_4_G Model has Chirality at C13         (Sohnke SpGr)           S Verify
PLAT791_ALERT_4_G Model has Chirality at C14         (Sohnke SpGr)           R Verify
PLAT912_ALERT_4_G Missing # of FCF Reflections Above STh/L=  0.600          65 Note
PLAT916_ALERT_2_G Hooft y and Flack x Parameter Values Differ by .        0.10 Check
PLAT941_ALERT_3_G Average HKL Measurement Multiplicity ...........         2.5 Low
PLAT978_ALERT_2_G Number C-C Bonds with Positive Residual Density.           1 Info
```

 0 **ALERT level A** = Most likely a serious problem - resolve or explain
 1 **ALERT level B** = A potentially serious problem, consider carefully
 6 **ALERT level C** = Check. Ensure it is not caused by an omission or oversight
14 **ALERT level G** = General information/check it is not something unexpected

 0 ALERT type 1 CIF construction/syntax error, inconsistent or missing data
 6 ALERT type 2 Indicator that the structure model may be wrong or deficient
 5 ALERT type 3 Indicator that the structure quality may be low
 9 ALERT type 4 Improvement, methodology, query or suggestion
 1 ALERT type 5 Informative message, check

## Validation response form

Please find below a validation response form (VRF) that can be filled in and pasted into your CIF.

```
# start Validation Reply Form
_vrf_STRVA01_qxb_cph_ra
;
PROBLEM: Flack parameter is too small
RESPONSE: ...
;
_vrf_PLAT029_qxb_cph_ra
;
PROBLEM: _diffrn_measured_fraction_theta_full value Low .      0.954 Why?
RESPONSE: ...
;
_vrf_PLAT340_qxb_cph_ra
;
PROBLEM: Low Bond Precision on  C-C Bonds ..............    0.00744 Ang.
RESPONSE: ...
;
_vrf_PLAT414_qxb_cph_ra
;
PROBLEM: Short Intra D-H..H-X       H3       ..H12      .       1.99 Ang.
RESPONSE: ...
;
_vrf_PLAT417_qxb_cph_ra
;
PROBLEM: Short Inter D-H..H-D       H2       ..H3       .       2.12 Ang.
RESPONSE: ...
;
_vrf_PLAT911_qxb_cph_ra
;
PROBLEM: Missing FCF Refl Between Thmin & STh/L=    0.600         94 Report
RESPONSE: ...
;
_vrf_PLAT915_qxb_cph_ra
;
PROBLEM: No Flack x Check Done: Low Friedel Pair Coverage         57 %
RESPONSE: ...
;
# end Validation Reply Form
```

It is advisable to attempt to resolve as many as possible of the alerts in all categories. Often the minor alerts point to easily fixed oversights, errors and omissions in your CIF or refinement strategy, so attention to these fine details can be worthwhile. In order to resolve some of the more serious problems it may be necessary to carry out additional measurements or structure refinements. However, the purpose of your study may justify the reported deviations and the more serious of these should normally be commented upon in the discussion or experimental section of a paper or in the "special_details" fields of the CIF. checkCIF was carefully designed to identify outliers and unusual parameters, but every test has its limitations and alerts that are not important in a particular case may appear. Conversely, the absence of alerts does not guarantee there are no aspects of the results needing attention. It is up to the individual to critically assess their own results and, if necessary, seek expert advice.

**Publication of your CIF in IUCr journals**

A basic structural check has been run on your CIF. These basic checks will be run on all CIFs submitted for publication in IUCr journals (*Acta Crystallographica*, *Journal of Applied Crystallography*, *Journal of Synchrotron Radiation*); however, if you intend to submit to *Acta Crystallographica Section C* or *E* or *IUCrData*, you should make sure that full publication checks are run on the final version of your CIF prior to submission.

**Publication of your CIF in other journals**

Please refer to the *Notes for Authors* of the relevant journal for any special instructions relating to CIF submission.

---

**PLATON version of 18/05/2022; check.def file version of 17/05/2022**

**Datablock qxb_cph_ra** - ellipsoid plot

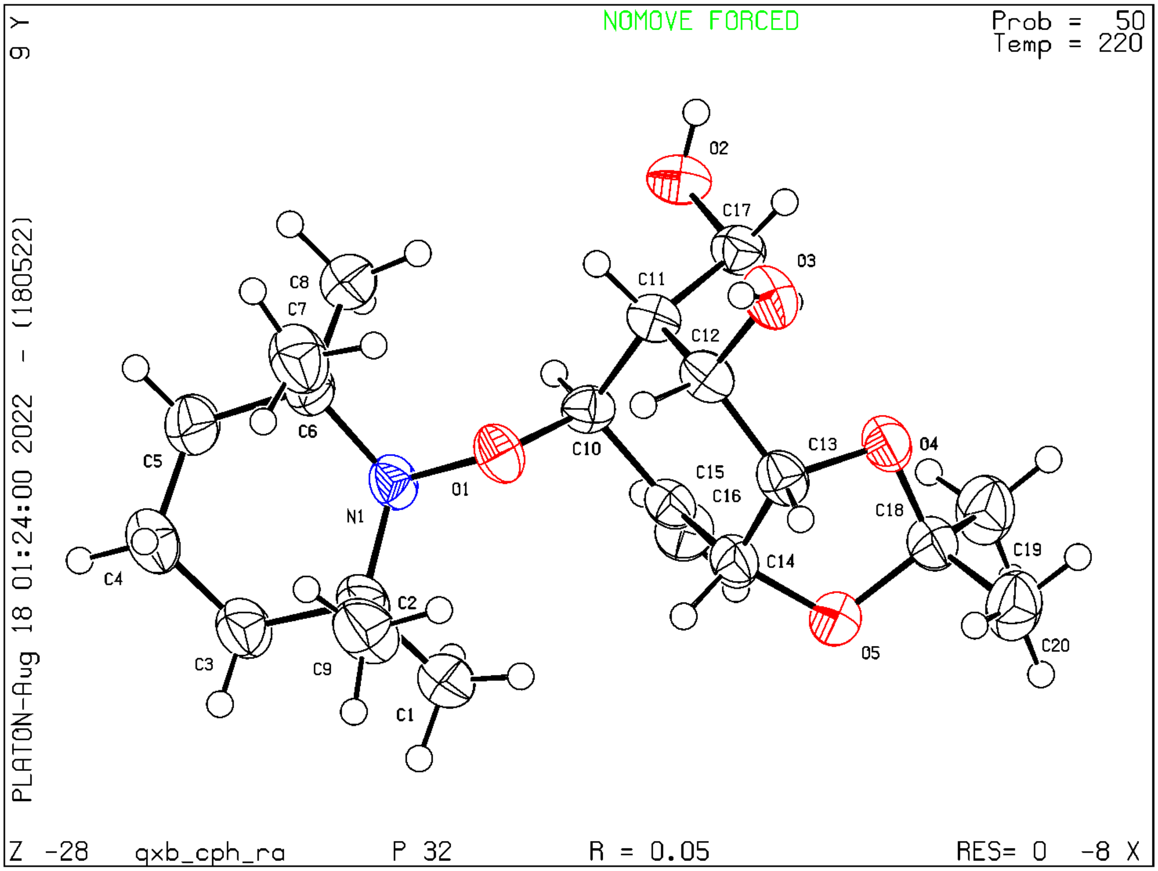

Supplement: Supplementary file 4 — Source Data [file 41467_2024_45037_MOESM4_ESM.zip › Surce Data 20231213/Surce Data New/Crystal Structure Source Data/CCDC-2182018/checkcif .pdf]
