# Supplementary material for: Total syntheses of Tetrodotoxin and 9-epiTetrodotoxin
Source: Nat Commun. 2024 Jan 23;15:679. doi: 10.1038/s41467-024-45037-0 (PMC10806222; doi:10.1038/s41467-024-45037-0)
Supplement: Supplementary file 4 — Source Data [file 41467_2024_45037_MOESM4_ESM.zip › Surce Data 20231213/Surce Data New/Crystal Structure Source Data/CCDC-2182018/QXB_CPH_Ra-2182018_tables.html]

QXB\_CPH\_Ra-2182018


# QXB\_CPH\_Ra-2182018

Table 1 Crystal data and structure refinement for QXB\_CPH\_Ra-2182018.

| Identification code | QXB\_CPH\_Ra-2182018 |
| Empirical formula | C20H35NO5 |
| Formula weight | 369.49 |
| Temperature/K | 220.00(10) |
| Crystal system | trigonal |
| Space group | P32 |
| a/Å | 17.6413(14) |
| b/Å | 17.6413(14) |
| c/Å | 6.2712(6) |
| α/° | 90 |
| β/° | 90 |
| γ/° | 120 |
| Volume/Å3 | 1690.2(3) |
| Z | 3 |
| ρcalcg/cm3 | 1.089 |
| μ/mm‑1 | 0.624 |
| F(000) | 606.0 |
| Crystal size/mm3 | 0.1 × 0.05 × 0.05 |
| Radiation | CuKα (λ = 1.54184) |
| 2Θ range for data collection/° | 5.784 to 145.096 |
| Index ranges | -18 ≤ h ≤ 21, -21 ≤ k ≤ 16, -7 ≤ l ≤ 6 |
| Reflections collected | 5103 |
| Independent reflections | 3347 [Rint = 0.0478, Rsigma = 0.0866] |
| Data/restraints/parameters | 3347/1/243 |
| Goodness-of-fit on F2 | 0.946 |
| Final R indexes [I>=2σ (I)] | R1 = 0.0475, wR2 = 0.1106 |
| Final R indexes [all data] | R1 = 0.0664, wR2 = 0.1205 |
| Largest diff. peak/hole / e Å-3 | 0.13/-0.19 || Flack parameter | -0.3(3) |

Table 2 Fractional Atomic Coordinates (×104) and Equivalent Isotropic Displacement Parameters (Å2×103) for QXB\_CPH\_Ra-2182018. Ueq is defined as 1/3 of the trace of the orthogonalised UIJ tensor.

| Atom | *x* | *y* | *z* | U(eq) |
| --- | --- | --- | --- | --- |
| O(1) | 3146.8(19) | 2453(2) | 5738(4) | 39.4(7) |
| O(3) | 5465.6(19) | 2672(2) | 7988(5) | 44.8(8) |
| O(2) | 5359(2) | 3425(2) | 1698(5) | 46.1(8) |
| O(5) | 3337(2) | 66(2) | 5025(6) | 46.1(8) |
| O(4) | 4763.1(19) | 1111(2) | 5629(5) | 43.5(7) |
| N(1) | 2453(2) | 2585(2) | 4830(6) | 38.3(8) |
| C(12) | 4610(3) | 2307(3) | 7078(7) | 36.9(9) |
| C(18) | 4228(3) | 226(3) | 4981(8) | 44.4(11) |
| C(17) | 5269(3) | 2832(3) | 3369(7) | 40.2(10) |
| C(11) | 4612(3) | 2772(3) | 5026(7) | 37.0(9) |
| C(10) | 3667(3) | 2336(3) | 4148(7) | 34.5(9) |
| C(6) | 2761(3) | 3543(3) | 5054(7) | 41.1(11) |
| C(13) | 4220(3) | 1328(3) | 6881(7) | 38.8(10) |
| C(5) | 1994(3) | 3680(3) | 4438(9) | 52.5(13) |
| C(3) | 907(3) | 2152(3) | 5205(9) | 50.0(12) |
| C(15) | 3331(3) | 1369(3) | 3738(7) | 37.2(9) |
| C(2) | 1617(3) | 1938(3) | 5898(9) | 48.4(12) |
| C(16) | 3048(3) | 1019(3) | 1859(8) | 46.6(11) |
| C(14) | 3366(3) | 861(3) | 5634(8) | 40.0(10) |
| C(4) | 1159(3) | 3101(4) | 5654(9) | 54.0(13) |
| C(19) | 4475(4) | 129(4) | 2744(9) | 58.8(14) |
| C(9) | 1640(4) | 1879(4) | 8310(9) | 63.1(16) |
| C(7) | 3099(3) | 3915(4) | 7263(9) | 58.5(14) |
| C(8) | 3493(3) | 4030(3) | 3452(9) | 50.1(12) |
| C(1) | 1349(3) | 1045(3) | 4932(12) | 68.0(17) |
| C(20) | 4274(4) | -398(4) | 6565(9) | 58.3(14) |

Table 3 Anisotropic Displacement Parameters (Å2×103) for QXB\_CPH\_Ra-2182018. The Anisotropic displacement factor exponent takes the form: -2π2[h2a\*2U11+2hka\*b\*U12+…].

| Atom | U11 | U22 | U33 | U23 | U13 | U12 |
| --- | --- | --- | --- | --- | --- | --- |
| O(1) | 36.5(15) | 54.5(18) | 33.0(17) | -1.5(14) | -3.6(13) | 27.2(14) |
| O(3) | 41.0(17) | 60(2) | 37.0(19) | -12.4(16) | -10.9(14) | 28.3(15) |
| O(2) | 40.1(16) | 44.0(17) | 42.5(19) | 4.5(14) | 4.2(14) | 12.2(14) |
| O(5) | 44.4(18) | 39.9(16) | 56(2) | 4.1(15) | 6.9(15) | 22.9(14) |
| O(4) | 37.7(16) | 49.8(18) | 49(2) | 4.7(15) | 5.5(14) | 26.1(15) |
| N(1) | 34.3(19) | 46(2) | 39(2) | 3.0(17) | -4.6(16) | 23.7(16) |
| C(12) | 34(2) | 45(2) | 30(2) | -0.8(19) | 0.6(18) | 18.3(19) |
| C(18) | 40(2) | 45(2) | 50(3) | 5(2) | 7(2) | 24(2) |
| C(17) | 36(2) | 40(2) | 40(3) | 2(2) | 5.0(19) | 16.5(19) |
| C(11) | 33(2) | 38(2) | 36(3) | 1.2(19) | 1.9(19) | 14.7(18) |
| C(10) | 36(2) | 35(2) | 30(2) | 5.1(18) | 1.0(17) | 15.3(18) |
| C(6) | 39(2) | 41(2) | 46(3) | -2(2) | -5(2) | 22.3(19) |
| C(13) | 39(2) | 50(3) | 32(2) | 5(2) | 5.6(19) | 25(2) |
| C(5) | 47(3) | 51(3) | 65(4) | 1(2) | -7(2) | 28(2) |
| C(3) | 41(2) | 57(3) | 56(3) | 3(2) | -3(2) | 27(2) |
| C(15) | 31(2) | 42(2) | 37(2) | 0(2) | 5.0(18) | 17.5(19) |
| C(2) | 38(2) | 55(3) | 58(3) | 17(2) | 6(2) | 27(2) |
| C(16) | 47(3) | 46(3) | 45(3) | -9(2) | -11(2) | 22(2) |
| C(14) | 38(2) | 44(2) | 42(3) | 3(2) | 8(2) | 24(2) |
| C(4) | 43(3) | 65(3) | 62(4) | -2(3) | -3(2) | 33(3) |
| C(19) | 70(3) | 64(3) | 53(3) | 1(3) | 12(3) | 42(3) |
| C(9) | 53(3) | 84(4) | 57(4) | 33(3) | 15(3) | 38(3) |
| C(7) | 50(3) | 69(4) | 63(4) | -14(3) | -11(3) | 34(3) |
| C(8) | 46(3) | 43(3) | 60(3) | 5(2) | 1(2) | 22(2) |
| C(1) | 37(3) | 49(3) | 115(5) | 12(3) | 5(3) | 20(2) |
| C(20) | 71(3) | 63(3) | 57(3) | 12(3) | 7(3) | 45(3) |

Table 4 Bond Lengths for QXB\_CPH\_Ra-2182018.

| Atom | Atom | Length/Å |  | Atom | Atom | Length/Å |
| --- | --- | --- | --- | --- | --- | --- |
| O(1) | N(1) | 1.469(4) |  | C(17) | C(11) | 1.520(6) |
| O(1) | C(10) | 1.438(5) |  | C(11) | C(10) | 1.548(5) |
| O(3) | C(12) | 1.430(5) |  | C(10) | C(15) | 1.523(6) |
| O(2) | C(17) | 1.432(5) |  | C(6) | C(5) | 1.537(6) |
| O(5) | C(18) | 1.452(5) |  | C(6) | C(7) | 1.521(7) |
| O(5) | C(14) | 1.429(5) |  | C(6) | C(8) | 1.519(7) |
| O(4) | C(18) | 1.422(5) |  | C(13) | C(14) | 1.523(6) |
| O(4) | C(13) | 1.430(5) |  | C(5) | C(4) | 1.514(7) |
| N(1) | C(6) | 1.501(6) |  | C(3) | C(2) | 1.539(6) |
| N(1) | C(2) | 1.498(6) |  | C(3) | C(4) | 1.527(7) |
| C(12) | C(11) | 1.525(6) |  | C(15) | C(16) | 1.308(6) |
| C(12) | C(13) | 1.511(6) |  | C(15) | C(14) | 1.508(6) |
| C(18) | C(19) | 1.505(7) |  | C(2) | C(9) | 1.518(7) |
| C(18) | C(20) | 1.515(7) |  | C(2) | C(1) | 1.526(8) |

Table 5 Bond Angles for QXB\_CPH\_Ra-2182018.

| Atom | Atom | Atom | Angle/˚ |  | Atom | Atom | Atom | Angle/˚ |
| --- | --- | --- | --- | --- | --- | --- | --- | --- |
| C(10) | O(1) | N(1) | 113.3(3) |  | N(1) | C(6) | C(7) | 114.4(4) |
| C(14) | O(5) | C(18) | 108.1(3) |  | N(1) | C(6) | C(8) | 107.5(4) |
| C(18) | O(4) | C(13) | 106.6(3) |  | C(7) | C(6) | C(5) | 110.3(4) |
| O(1) | N(1) | C(6) | 106.3(3) |  | C(8) | C(6) | C(5) | 108.1(4) |
| O(1) | N(1) | C(2) | 106.7(3) |  | C(8) | C(6) | C(7) | 108.6(4) |
| C(2) | N(1) | C(6) | 118.6(4) |  | O(4) | C(13) | C(12) | 111.6(3) |
| O(3) | C(12) | C(11) | 112.1(3) |  | O(4) | C(13) | C(14) | 100.8(3) |
| O(3) | C(12) | C(13) | 108.8(3) |  | C(12) | C(13) | C(14) | 115.0(4) |
| C(13) | C(12) | C(11) | 115.0(4) |  | C(4) | C(5) | C(6) | 113.8(4) |
| O(5) | C(18) | C(19) | 110.1(4) |  | C(4) | C(3) | C(2) | 113.0(4) |
| O(5) | C(18) | C(20) | 107.0(4) |  | C(16) | C(15) | C(10) | 121.6(4) |
| O(4) | C(18) | O(5) | 105.5(3) |  | C(16) | C(15) | C(14) | 123.3(4) |
| O(4) | C(18) | C(19) | 108.7(4) |  | C(14) | C(15) | C(10) | 115.1(4) |
| O(4) | C(18) | C(20) | 111.3(4) |  | N(1) | C(2) | C(3) | 107.4(4) |
| C(19) | C(18) | C(20) | 113.9(4) |  | N(1) | C(2) | C(9) | 116.6(4) |
| O(2) | C(17) | C(11) | 110.7(4) |  | N(1) | C(2) | C(1) | 106.7(4) |
| C(12) | C(11) | C(10) | 108.7(3) |  | C(9) | C(2) | C(3) | 110.6(4) |
| C(17) | C(11) | C(12) | 114.3(4) |  | C(9) | C(2) | C(1) | 109.2(5) |
| C(17) | C(11) | C(10) | 112.7(4) |  | C(1) | C(2) | C(3) | 105.8(4) |
| O(1) | C(10) | C(11) | 106.7(3) |  | O(5) | C(14) | C(13) | 102.0(3) |
| O(1) | C(10) | C(15) | 111.1(3) |  | O(5) | C(14) | C(15) | 112.3(4) |
| C(15) | C(10) | C(11) | 109.6(3) |  | C(15) | C(14) | C(13) | 114.0(4) |
| N(1) | C(6) | C(5) | 107.6(4) |  | C(5) | C(4) | C(3) | 107.3(4) |

Table 6 Hydrogen Atom Coordinates (Å×104) and Isotropic Displacement Parameters (Å2×103) for QXB\_CPH\_Ra-2182018.

| Atom | *x* | *y* | *z* | U(eq) |
| --- | --- | --- | --- | --- |
| H(3) | 5465.97 | 2862.03 | 9181.81 | 67 |
| H(2) | 5871.3 | 3817.88 | 1622.31 | 69 |
| H(12) | 4249.6 | 2404.23 | 8108.32 | 44 |
| H(17A) | 5832.43 | 3031.92 | 4043.81 | 48 |
| H(17B) | 5074.98 | 2256.4 | 2771.74 | 48 |
| H(11) | 4778.33 | 3373.53 | 5416.45 | 44 |
| H(10) | 3657.23 | 2623.72 | 2818.63 | 41 |
| H(13) | 4136.82 | 1067.73 | 8302.49 | 47 |
| H(5A) | 2164.14 | 4287.05 | 4689.95 | 63 |
| H(5B) | 1880.28 | 3567.31 | 2924.25 | 63 |
| H(3A) | 800.29 | 2039.71 | 3689.3 | 60 |
| H(3B) | 367.27 | 1766.53 | 5949.34 | 60 |
| H(16A) | 2854.71 | 427.89 | 1642.61 | 56 |
| H(16B) | 3040.13 | 1360.14 | 739.34 | 56 |
| H(14) | 2874.88 | 726.83 | 6589.83 | 48 |
| H(4A) | 696.42 | 3207.75 | 5186.94 | 65 |
| H(4B) | 1250.61 | 3221.08 | 7169.66 | 65 |
| H(19A) | 4091.24 | -457.02 | 2252.1 | 88 |
| H(19B) | 5068.16 | 244.56 | 2726.84 | 88 |
| H(19C) | 4424.73 | 536.26 | 1824.46 | 88 |
| H(9A) | 2109.58 | 1781.46 | 8716.98 | 95 |
| H(9B) | 1094.81 | 1401.89 | 8806.6 | 95 |
| H(9C) | 1732.18 | 2415.57 | 8931.87 | 95 |
| H(7A) | 2621.17 | 3676.22 | 8257.5 | 88 |
| H(7B) | 3361.92 | 4540.15 | 7225.65 | 88 |
| H(7C) | 3527.23 | 3762.17 | 7700.36 | 88 |
| H(8A) | 3999.2 | 4000.12 | 3884.52 | 75 |
| H(8B) | 3636.63 | 4631.13 | 3387.23 | 75 |
| H(8C) | 3305.35 | 3766.67 | 2070.74 | 75 |
| H(1A) | 1401.36 | 1095.52 | 3408.39 | 102 |
| H(1B) | 753.47 | 637.18 | 5309.66 | 102 |
| H(1C) | 1724.14 | 840.14 | 5471.42 | 102 |
| H(20A) | 4100.54 | -306.55 | 7948.91 | 87 |
| H(20B) | 4862.52 | -292.68 | 6631.11 | 87 |
| H(20C) | 3887.79 | -990.12 | 6120.25 | 87 |

Table 7 Solvent masks information for QXB\_CPH\_Ra-2182018.

| Number | X | Y | Z | Volume | Electron count | Content |
| --- | --- | --- | --- | --- | --- | --- |
| 1 | 0.333 | 0.667 | -0.566 | 234.9 | 53.8 | ? |

Experimental

Single crystals of C20H35NO5
[QXB\_CPH\_Ra-2182018]
were
[].
A suitable crystal was selected and
[]
on a
ROD, Synergy Custom system, HyPix
diffractometer. The crystal was kept at 220.00(10) K during data collection.
Using Olex2 [1], the structure was solved with the
Unknown
[2] structure solution program using
Unknown
and refined with the
Unknown
[3] refinement package using
Unknown
minimisation.

1. Dolomanov, O.V., Bourhis, L.J., Gildea, R.J, Howard, J.A.K. & Puschmann, H.
   (2009), J. Appl. Cryst. 42, 339-341.

Crystal structure determination of
[QXB\_CPH\_Ra-2182018]

**Crystal Data**
for C20H35NO5 (*M*=369.49 g/mol):
trigonal, space group P32 (no. 145),
*a* = 17.6413(14) Å, *c* = 6.2712(6) Å,
*V*= 1690.2(3) Å3,
*Z* = 3,
*T* = 220.00(10) K,
μ(CuKα) = 0.624 mm-1,
*Dcalc* = 1.089 g/cm3,
5103 reflections measured (5.784° ≤ 2Θ ≤ 145.096°),
3347 unique (*R*int = 0.0478, Rsigma = 0.0866) which were used in all calculations.
The final *R*1 was 0.0475
(I > 2σ(I)) and *wR*2 was 0.1205 (all data).

Refinement model description

Number of restraints - 1,
number of constraints - unknown.

Details:

```
1.a Ternary CH refined with riding coordinates:
```

This report has been created with Olex2, compiled on
2022.04.07 svn.rca3783a0 for OlexSys. Please
let us know
if there are any errors or if you would like to have additional features.
