# Supplementary material for: Total syntheses of Tetrodotoxin and 9-epiTetrodotoxin
Source: Nat Commun. 2024 Jan 23;15:679. doi: 10.1038/s41467-024-45037-0 (PMC10806222; doi:10.1038/s41467-024-45037-0)
Supplement: Supplementary file 4 — Source Data [file 41467_2024_45037_MOESM4_ESM.zip › Surce Data 20231213/Surce Data New/Crystal Structure Source Data/CCDC-2184298/20200811d_tables.html]

20200811d


# 20200811d

Table 1 Crystal data and structure refinement for 20200811d.

| Identification code | 20200811d |
| Empirical formula | C27H38O7Si |
| Formula weight | 502.66 |
| Temperature/K | 100.00(10) |
| Crystal system | monoclinic |
| Space group | C2 |
| a/Å | 21.9882(2) |
| b/Å | 7.80324(9) |
| c/Å | 16.22556(19) |
| α/° | 90 |
| β/° | 91.3178(11) |
| γ/° | 90 |
| Volume/Å3 | 2783.23(5) |
| Z | 4 |
| ρcalcg/cm3 | 1.200 |
| μ/mm‑1 | 1.085 |
| F(000) | 1080.0 |
| Crystal size/mm3 | 0.2 × 0.1 × 0.02 |
| Radiation | CuKα (λ = 1.54184) |
| 2Θ range for data collection/° | 8.044 to 151.292 |
| Index ranges | -22 ≤ h ≤ 27, -9 ≤ k ≤ 9, -20 ≤ l ≤ 20 |
| Reflections collected | 15417 |
| Independent reflections | 5558 [Rint = 0.0276, Rsigma = 0.0300] |
| Data/restraints/parameters | 5558/49/326 |
| Goodness-of-fit on F2 | 1.057 |
| Final R indexes [I>=2σ (I)] | R1 = 0.0330, wR2 = 0.0816 |
| Final R indexes [all data] | R1 = 0.0337, wR2 = 0.0821 |
| Largest diff. peak/hole / e Å-3 | 0.25/-0.19 || Flack parameter | 0.017(11) |

Table 2 Fractional Atomic Coordinates (×104) and Equivalent Isotropic Displacement Parameters (Å2×103) for 20200811d. Ueq is defined as 1/3 of of the trace of the orthogonalised UIJ tensor.

| Atom | *x* | *y* | *z* | U(eq) |
| Si12 | 2861.2(3) | 7595.8(9) | 2042.9(4) | 31.31(17) |
| O6 | 3981.1(6) | 9936.3(19) | 6260.4(9) | 19.2(3) |
| O20 | 3140.8(6) | 7805(2) | 5255.6(9) | 23.3(3) |
| O21 | 4308.6(7) | 12142.5(19) | 4314.6(9) | 21.2(3) |
| O4 | 4336.1(6) | 7419.6(18) | 5739.9(8) | 18.9(3) |
| O18 | 4505.2(7) | 13932(2) | 5741.2(11) | 26.2(3) |
| O19 | 2868.5(7) | 11128(2) | 5246.6(10) | 24.7(3) |
| O11 | 3004.1(8) | 7897(2) | 3030.0(10) | 32.1(4) |
| C17 | 3900.3(10) | 13373(3) | 5552.1(14) | 22.7(4) |
| C8 | 3940.7(9) | 11792(3) | 5011.4(13) | 18.5(4) |
| C9 | 3320.6(9) | 11296(3) | 4627.0(13) | 21.3(4) |
| C7 | 4244.3(9) | 10294(3) | 5477.7(12) | 16.7(4) |
| C5 | 4224.8(10) | 8330(3) | 6507.3(13) | 20.3(4) |
| C3 | 4199.1(9) | 8544(3) | 5062.0(12) | 17.0(4) |
| C22 | 4832.1(10) | 8534(3) | 6968.0(14) | 25.6(5) |
| C30 | 2037.2(11) | 8096(4) | 1812.1(14) | 32.2(6) |
| C23 | 3767.4(10) | 7364(3) | 7006.1(14) | 27.0(5) |
| C33 | 793.4(12) | 8814(4) | 1533.0(16) | 36.1(6) |
| C1 | 3393.6(9) | 9679(3) | 4104.2(13) | 20.6(4) |
| C29 | 3278.9(13) | 9077(6) | 538(2) | 57.4(10) |
| C24 | 3358.8(11) | 8983(4) | 1389.7(17) | 41.4(7) |
| C10 | 2845.7(10) | 9298(3) | 3542.8(14) | 26.8(5) |
| C2 | 3583.9(9) | 8147(3) | 4642.4(13) | 19.5(4) |
| C35 | 1843.5(12) | 9318(4) | 1232.5(15) | 37.0(6) |
| C31 | 1584.9(12) | 7272(4) | 2264.3(16) | 35.5(6) |
| C34 | 1232.2(13) | 9668(4) | 1090.6(16) | 38.5(6) |
| C32 | 972.6(11) | 7636(4) | 2127.8(16) | 37.4(6) |
| C13 | 3095.1(13) | 5282(4) | 1902.6(16) | 40.4(7) |
| C14 | 3767.3(18) | 5123(5) | 2148(3) | 68.0(11) |
| C25 | 3851.9(12) | 9865(4) | 1740(2) | 42.4(7) |
| C28 | 3667.4(14) | 10045(7) | 52(2) | 72.6(13) |
| C26 | 4248.0(13) | 10808(5) | 1256(2) | 56.6(9) |
| C27 | 4152.1(14) | 10910(6) | 421(3) | 70.8(12) |
| C16 | 3013(2) | 4744(8) | 1006(2) | 83.5(15) |
| C15 | 2734(3) | 4104(5) | 2445(4) | 97.7(19) |

Table 3 Anisotropic Displacement Parameters (Å2×103) for 20200811d. The Anisotropic displacement factor exponent takes the form: -2π2[h2a\*2U11+2hka\*b\*U12+…].

| Atom | U11 | U22 | U33 | U23 | U13 | U12 |
| Si12 | 28.7(3) | 46.8(4) | 18.1(3) | 5.5(3) | -6.5(2) | -16.1(3) |
| O6 | 18.6(7) | 19.1(7) | 20.0(7) | 0.7(6) | -0.6(5) | 2.7(6) |
| O20 | 16.5(7) | 30.3(8) | 22.9(7) | 2.4(6) | -1.9(5) | -8.1(6) |
| O21 | 19.4(7) | 20.8(7) | 23.5(7) | 1.0(6) | 0.5(6) | -2.0(6) |
| O4 | 21.4(7) | 15.4(7) | 19.8(7) | -1.1(6) | -3.0(5) | 2.3(6) |
| O18 | 24.2(8) | 16.3(7) | 37.8(9) | -4.6(6) | -4.9(6) | 2.3(6) |
| O19 | 15.6(7) | 32.1(9) | 26.5(8) | 3.5(7) | 1.8(6) | 5.0(6) |
| O11 | 40.7(9) | 33.4(9) | 21.9(8) | 2.1(7) | -10.1(7) | -10.7(7) |
| C17 | 19.6(10) | 19.2(10) | 29.1(11) | -0.8(9) | -2.6(8) | 4.7(8) |
| C8 | 16.7(10) | 18.9(10) | 19.9(10) | 1.0(8) | -0.7(8) | 2.1(7) |
| C9 | 15.8(9) | 25.8(11) | 22.3(11) | 3.8(9) | -1.3(8) | 3.3(8) |
| C7 | 14.2(9) | 17.2(9) | 18.7(9) | -1.3(8) | -1.4(7) | 0.8(7) |
| C5 | 22.3(10) | 18.4(10) | 20.1(10) | -1.5(8) | -2.4(8) | 2.8(8) |
| C3 | 16.9(9) | 14.9(9) | 19.1(10) | 1.3(7) | -1.1(7) | 0.1(7) |
| C22 | 25.0(11) | 26.7(11) | 24.6(11) | -1.7(9) | -7.1(8) | 5.2(9) |
| C30 | 30.4(11) | 48.9(15) | 17.0(10) | -0.2(10) | -4.5(9) | -15.5(11) |
| C23 | 28.5(11) | 27.2(11) | 25.4(10) | 3.8(9) | 1.7(8) | 0.1(10) |
| C33 | 28.6(12) | 49.0(16) | 30.7(13) | -4.4(11) | -3.5(10) | -10.5(11) |
| C1 | 16.1(9) | 25.5(10) | 20.1(10) | 2.0(8) | -2.6(8) | -1.6(8) |
| C29 | 28.1(13) | 107(3) | 37.2(15) | 30.8(17) | -3.0(11) | -5.1(16) |
| C24 | 25.4(12) | 63.3(18) | 35.4(14) | 22.0(13) | -4.1(10) | -6.8(13) |
| C10 | 24.1(10) | 32.4(12) | 23.5(11) | 3.0(9) | -6.2(8) | -6.0(9) |
| C2 | 16.7(9) | 22.0(10) | 19.7(9) | -0.4(8) | -0.5(8) | -3.8(7) |
| C35 | 34.0(13) | 54.0(17) | 22.8(12) | 4.5(11) | -1.7(10) | -15.9(12) |
| C31 | 35.1(13) | 43.6(15) | 27.6(12) | 0.7(11) | -2.3(10) | -16.3(12) |
| C34 | 38.0(14) | 53.6(17) | 23.6(12) | 3.7(11) | -2.5(10) | -8.4(12) |
| C32 | 33.5(12) | 45.5(15) | 33.3(12) | -2.9(12) | 3.3(10) | -16.4(12) |
| C13 | 38.9(14) | 56.1(18) | 26.0(12) | -5.7(12) | -3.0(11) | -16.1(13) |
| C14 | 59(2) | 55(2) | 88(3) | 1(2) | -35(2) | -5.2(17) |
| C25 | 24.7(12) | 50.4(16) | 51.8(16) | 23.1(14) | -8.1(11) | -7.3(11) |
| C28 | 33.4(15) | 130(3) | 54(2) | 54(2) | 2.6(14) | 3.6(19) |
| C26 | 26.1(13) | 64(2) | 79(2) | 38.3(18) | -5.9(14) | -8.4(13) |
| C27 | 28.2(14) | 101(3) | 84(3) | 63(2) | 6.0(15) | -3.6(16) |
| C16 | 71(2) | 128(4) | 50(2) | -44(2) | -26.8(19) | 35(3) |
| C15 | 128(4) | 37.9(19) | 131(4) | -9(2) | 81(3) | -19(2) |

Table 4 Bond Lengths for 20200811d.

| Atom | Atom | Length/Å |  | Atom | Atom | Length/Å |
| Si12 | O11 | 1.6420(16) |  | C5 | C23 | 1.507(3) |
| Si12 | C30 | 1.882(3) |  | C3 | C2 | 1.532(3) |
| Si12 | C24 | 1.883(3) |  | C30 | C35 | 1.399(4) |
| Si12 | C13 | 1.893(3) |  | C30 | C31 | 1.405(3) |
| O6 | C7 | 1.435(2) |  | C33 | C34 | 1.386(4) |
| O6 | C5 | 1.417(2) |  | C33 | C32 | 1.384(4) |
| O20 | C2 | 1.433(2) |  | C1 | C10 | 1.523(3) |
| O21 | C8 | 1.432(3) |  | C1 | C2 | 1.533(3) |
| O4 | C5 | 1.459(2) |  | C29 | C24 | 1.391(4) |
| O4 | C3 | 1.433(2) |  | C29 | C28 | 1.397(5) |
| O18 | C17 | 1.427(3) |  | C24 | C25 | 1.394(4) |
| O19 | C9 | 1.436(3) |  | C35 | C34 | 1.386(4) |
| O11 | C10 | 1.422(3) |  | C31 | C32 | 1.389(4) |
| C17 | C8 | 1.518(3) |  | C13 | C14 | 1.527(4) |
| C8 | C9 | 1.536(3) |  | C13 | C16 | 1.521(4) |
| C8 | C7 | 1.536(3) |  | C13 | C15 | 1.510(5) |
| C9 | C1 | 1.531(3) |  | C25 | C26 | 1.396(4) |
| C7 | C3 | 1.526(3) |  | C28 | C27 | 1.386(6) |
| C5 | C22 | 1.524(3) |  | C26 | C27 | 1.369(6) |

Table 5 Bond Angles for 20200811d.

| Atom | Atom | Atom | Angle/˚ |  | Atom | Atom | Atom | Angle/˚ |
| O11 | Si12 | C30 | 109.03(10) |  | C7 | C3 | C2 | 115.16(17) |
| O11 | Si12 | C24 | 111.47(12) |  | C35 | C30 | Si12 | 123.50(19) |
| O11 | Si12 | C13 | 101.98(11) |  | C35 | C30 | C31 | 117.1(2) |
| C30 | Si12 | C24 | 109.74(12) |  | C31 | C30 | Si12 | 119.4(2) |
| C30 | Si12 | C13 | 115.91(12) |  | C32 | C33 | C34 | 119.3(2) |
| C24 | Si12 | C13 | 108.53(14) |  | C9 | C1 | C2 | 110.96(17) |
| C5 | O6 | C7 | 105.35(15) |  | C10 | C1 | C9 | 113.62(18) |
| C3 | O4 | C5 | 108.70(15) |  | C10 | C1 | C2 | 113.00(18) |
| C10 | O11 | Si12 | 129.56(15) |  | C24 | C29 | C28 | 121.6(3) |
| O18 | C17 | C8 | 107.84(17) |  | C29 | C24 | Si12 | 121.8(2) |
| O21 | C8 | C17 | 110.05(17) |  | C29 | C24 | C25 | 117.3(3) |
| O21 | C8 | C9 | 103.88(16) |  | C25 | C24 | Si12 | 120.7(2) |
| O21 | C8 | C7 | 106.70(16) |  | O11 | C10 | C1 | 107.39(19) |
| C17 | C8 | C9 | 112.11(17) |  | O20 | C2 | C3 | 109.66(16) |
| C17 | C8 | C7 | 111.36(17) |  | O20 | C2 | C1 | 111.08(17) |
| C9 | C8 | C7 | 112.33(17) |  | C3 | C2 | C1 | 108.82(16) |
| O19 | C9 | C8 | 111.17(17) |  | C34 | C35 | C30 | 121.7(2) |
| O19 | C9 | C1 | 113.31(18) |  | C32 | C31 | C30 | 121.2(3) |
| C1 | C9 | C8 | 109.23(17) |  | C35 | C34 | C33 | 120.2(3) |
| O6 | C7 | C8 | 113.85(16) |  | C33 | C32 | C31 | 120.4(2) |
| O6 | C7 | C3 | 101.16(15) |  | C14 | C13 | Si12 | 108.1(2) |
| C3 | C7 | C8 | 116.12(17) |  | C16 | C13 | Si12 | 110.6(3) |
| O6 | C5 | O4 | 105.02(16) |  | C16 | C13 | C14 | 108.6(3) |
| O6 | C5 | C22 | 111.56(17) |  | C15 | C13 | Si12 | 111.3(2) |
| O6 | C5 | C23 | 109.96(17) |  | C15 | C13 | C14 | 108.5(4) |
| O4 | C5 | C22 | 107.90(17) |  | C15 | C13 | C16 | 109.7(4) |
| O4 | C5 | C23 | 110.00(17) |  | C24 | C25 | C26 | 121.3(3) |
| C23 | C5 | C22 | 112.14(18) |  | C27 | C28 | C29 | 119.6(3) |
| O4 | C3 | C7 | 101.43(15) |  | C27 | C26 | C25 | 120.2(3) |
| O4 | C3 | C2 | 112.67(16) |  | C26 | C27 | C28 | 119.9(3) |

Table 6 Torsion Angles for 20200811d.

| A | B | C | D | Angle/˚ |  | A | B | C | D | Angle/˚ |
| Si12 | O11 | C10 | C1 | -138.63(17) |  | C7 | C3 | C2 | O20 | -73.3(2) |
| Si12 | C30 | C35 | C34 | -178.9(2) |  | C7 | C3 | C2 | C1 | 48.4(2) |
| Si12 | C30 | C31 | C32 | 178.4(2) |  | C5 | O6 | C7 | C8 | 167.77(16) |
| Si12 | C24 | C25 | C26 | 175.8(3) |  | C5 | O6 | C7 | C3 | 42.48(18) |
| O6 | C7 | C3 | O4 | -38.30(17) |  | C5 | O4 | C3 | C7 | 21.10(19) |
| O6 | C7 | C3 | C2 | 83.66(19) |  | C5 | O4 | C3 | C2 | -102.57(19) |
| O21 | C8 | C9 | O19 | -172.02(16) |  | C3 | O4 | C5 | O6 | 4.2(2) |
| O21 | C8 | C9 | C1 | 62.2(2) |  | C3 | O4 | C5 | C22 | -114.96(18) |
| O21 | C8 | C7 | O6 | 171.56(15) |  | C3 | O4 | C5 | C23 | 122.43(17) |
| O21 | C8 | C7 | C3 | -71.5(2) |  | C30 | Si12 | O11 | C10 | -46.9(2) |
| O4 | C3 | C2 | O20 | 42.4(2) |  | C30 | Si12 | C24 | C29 | -52.6(3) |
| O4 | C3 | C2 | C1 | 164.05(16) |  | C30 | Si12 | C24 | C25 | 132.1(3) |
| O18 | C17 | C8 | O21 | -53.3(2) |  | C30 | Si12 | C13 | C14 | -178.0(2) |
| O18 | C17 | C8 | C9 | -168.37(17) |  | C30 | Si12 | C13 | C16 | 63.2(3) |
| O18 | C17 | C8 | C7 | 64.8(2) |  | C30 | Si12 | C13 | C15 | -59.0(3) |
| O19 | C9 | C1 | C10 | 68.5(2) |  | C30 | C35 | C34 | C33 | 0.7(4) |
| O19 | C9 | C1 | C2 | -60.1(2) |  | C30 | C31 | C32 | C33 | 0.7(4) |
| O11 | Si12 | C30 | C35 | 122.8(2) |  | C29 | C24 | C25 | C26 | 0.2(5) |
| O11 | Si12 | C30 | C31 | -54.2(2) |  | C29 | C28 | C27 | C26 | 0.1(7) |
| O11 | Si12 | C24 | C29 | -173.5(3) |  | C24 | Si12 | O11 | C10 | 74.4(2) |
| O11 | Si12 | C24 | C25 | 11.2(3) |  | C24 | Si12 | C30 | C35 | 0.4(3) |
| O11 | Si12 | C13 | C14 | -59.7(3) |  | C24 | Si12 | C30 | C31 | -176.5(2) |
| O11 | Si12 | C13 | C16 | -178.5(3) |  | C24 | Si12 | C13 | C14 | 58.1(3) |
| O11 | Si12 | C13 | C15 | 59.3(3) |  | C24 | Si12 | C13 | C16 | -60.7(3) |
| C17 | C8 | C9 | O19 | -53.2(2) |  | C24 | Si12 | C13 | C15 | 177.1(3) |
| C17 | C8 | C9 | C1 | -179.00(18) |  | C24 | C29 | C28 | C27 | 1.3(7) |
| C17 | C8 | C7 | O6 | 51.5(2) |  | C24 | C25 | C26 | C27 | 1.2(6) |
| C17 | C8 | C7 | C3 | 168.35(17) |  | C10 | C1 | C2 | O20 | -69.4(2) |
| C8 | C9 | C1 | C10 | -166.98(18) |  | C10 | C1 | C2 | C3 | 169.81(18) |
| C8 | C9 | C1 | C2 | 64.4(2) |  | C2 | C1 | C10 | O11 | -59.2(2) |
| C8 | C7 | C3 | O4 | -162.06(16) |  | C35 | C30 | C31 | C32 | 1.2(4) |
| C8 | C7 | C3 | C2 | -40.1(2) |  | C31 | C30 | C35 | C34 | -1.9(4) |
| C9 | C8 | C7 | O6 | -75.2(2) |  | C34 | C33 | C32 | C31 | -1.9(4) |
| C9 | C8 | C7 | C3 | 41.7(2) |  | C32 | C33 | C34 | C35 | 1.2(4) |
| C9 | C1 | C10 | O11 | 173.26(17) |  | C13 | Si12 | O11 | C10 | -169.93(19) |
| C9 | C1 | C2 | O20 | 59.6(2) |  | C13 | Si12 | C30 | C35 | -122.9(2) |
| C9 | C1 | C2 | C3 | -61.2(2) |  | C13 | Si12 | C30 | C31 | 60.1(2) |
| C7 | O6 | C5 | O4 | -29.83(19) |  | C13 | Si12 | C24 | C29 | 75.0(3) |
| C7 | O6 | C5 | C22 | 86.80(19) |  | C13 | Si12 | C24 | C25 | -100.4(3) |
| C7 | O6 | C5 | C23 | -148.13(17) |  | C25 | C26 | C27 | C28 | -1.3(6) |
| C7 | C8 | C9 | O19 | 73.1(2) |  | C28 | C29 | C24 | Si12 | -176.9(3) |
| C7 | C8 | C9 | C1 | -52.7(2) |  | C28 | C29 | C24 | C25 | -1.4(6) |

Table 7 Hydrogen Atom Coordinates (Å×104) and Isotropic Displacement Parameters (Å2×103) for 20200811d.

| Atom | *x* | *y* | *z* | U(eq) |
| H20 | 2866 | 7220 | 5054 | 35 |
| H21 | 4590 | 12774 | 4455 | 32 |
| H18 | 4517 | 14982 | 5736 | 39 |
| H19 | 2870 | 10144 | 5424 | 37 |
| H17A | 3676 | 14270 | 5264 | 27 |
| H17B | 3691 | 13102 | 6055 | 27 |
| H9 | 3192 | 12226 | 4256 | 26 |
| H7 | 4675 | 10569 | 5570 | 20 |
| H3 | 4520 | 8445 | 4655 | 20 |
| H22A | 5089 | 9291 | 6666 | 38 |
| H22B | 5025 | 7435 | 7024 | 38 |
| H22C | 4765 | 9004 | 7505 | 38 |
| H23A | 3713 | 7938 | 7523 | 41 |
| H23B | 3912 | 6220 | 7106 | 41 |
| H23C | 3386 | 7319 | 6707 | 41 |
| H33 | 383 | 9032 | 1431 | 43 |
| H1 | 3734 | 9901 | 3740 | 25 |
| H29 | 2959 | 8480 | 285 | 69 |
| H10A | 2496 | 9006 | 3868 | 32 |
| H10B | 2744 | 10294 | 3209 | 32 |
| H2 | 3626 | 7133 | 4292 | 23 |
| H35 | 2132 | 9910 | 935 | 44 |
| H31 | 1698 | 6468 | 2662 | 43 |
| H34 | 1116 | 10478 | 697 | 46 |
| H32 | 681 | 7084 | 2438 | 45 |
| H14A | 4000 | 5915 | 1830 | 102 |
| H14B | 3903 | 3976 | 2043 | 102 |
| H14C | 3821 | 5378 | 2723 | 102 |
| H25 | 3918 | 9824 | 2308 | 51 |
| H28 | 3601 | 10108 | -515 | 87 |
| H26 | 4578 | 11370 | 1502 | 68 |
| H27 | 4412 | 11558 | 102 | 85 |
| H16A | 2595 | 4890 | 836 | 125 |
| H16B | 3126 | 3562 | 948 | 125 |
| H16C | 3267 | 5440 | 668 | 125 |
| H15A | 2764 | 4496 | 3005 | 147 |
| H15B | 2895 | 2962 | 2411 | 147 |
| H15C | 2316 | 4105 | 2263 | 147 |

Table 8 Solvent masks information for 20200811d.

| Number | X | Y | Z | Volume | Electron count | Content |
| 1 | 0.000 | 0.177 | 0.000 | 95.6 | 10.7 | ? |
| 2 | 0.500 | 0.677 | 0.000 | 95.6 | 10.7 | ? |

Experimental

Single crystals of C27H38O7Si
[20200811d]
were
[].
A suitable crystal was selected and
[]
on a
XtaLAB Synergy R, DW system, HyPix
diffractometer. The crystal was kept at 100.00(10) K during data collection.
Using Olex2 [1], the structure was solved with the
ShelXT
[2] structure solution program using
Intrinsic Phasing
and refined with the
ShelXL
[3] refinement package using
Least Squares
minimisation.

1. Dolomanov, O.V., Bourhis, L.J., Gildea, R.J, Howard, J.A.K. & Puschmann, H.
   (2009), J. Appl. Cryst. 42, 339-341.
2. Sheldrick, G.M. (2015). Acta Cryst. A71, 3-8.
3. Sheldrick, G.M. (2015). Acta Cryst. C71, 3-8.

Crystal structure determination of
[20200811d]

**Crystal Data**
for C27H38O7Si (*M*=502.66 g/mol):
monoclinic, space group C2 (no. 5),
*a* = 21.9882(2) Å, *b* = 7.80324(9) Å, *c* = 16.22556(19) Å, *β* = 91.3178(11)°,
*V*= 2783.23(5) Å3,
*Z* = 4,
*T* = 100.00(10) K,
μ(CuKα) = 1.085 mm-1,
*Dcalc* = 1.200 g/cm3,
15417 reflections measured (8.044° ≤ 2Θ ≤ 151.292°),
5558 unique (*R*int = 0.0276, Rsigma = 0.0300) which were used in all calculations.
The final *R*1 was 0.0330
(I > 2σ(I)) and *wR*2 was 0.0821 (all data).

Refinement model description

Number of restraints - 49,
number of constraints - unknown.

Details:

```
1. Fixed Uiso
```

This report has been created with Olex2, compiled on
2018.05.29 svn.r3508 for OlexSys. Please
let us know
if there are any errors or if you would like to have additional features.
