# Supplementary material for: Total syntheses of Tetrodotoxin and 9-epiTetrodotoxin
Source: Nat Commun. 2024 Jan 23;15:679. doi: 10.1038/s41467-024-45037-0 (PMC10806222; doi:10.1038/s41467-024-45037-0)

checkCIF/PLATON report

Structure factors have been supplied for datablock(s) 20200811d

THIS REPORT IS FOR GUIDANCE ONLY. IF USED AS PART OF A REVIEW PROCEDURE FOR PUBLICATION, IT SHOULD NOT REPLACE THE EXPERTISE OF AN EXPERIENCED CRYSTALLOGRAPHIC REFEREE.

No syntax errors found. CIF dictionary Interpreting this report

Datablock: 20200811d

```
Bond precision:   C-C = 0.0039 A          Wavelength=1.54184

Cell:            a=21.9882(2)    b=7.80324(9)        c=16.22556(19)
                 alpha=90        beta=91.3178(11)    gamma=90
Temperature:     100 K

                 Calculated                   Reported
Volume           2783.23(5)                   2783.23(5)
Space group      C 2                          C 1 2 1
Hall group       C 2y                         C 2y
Moiety formula   C27 H38 O7 Si [+ solvent]    C27 H38 O7 Si
Sum formula      C27 H38 O7 Si [+ solvent]    C27 H38 O7 Si
Mr               502.66                       502.66
Dx,g cm-3        1.200                        1.200
Z                4                            4
Mu (mm-1)        1.085                        1.085
F000             1080.0                       1080.0
F000'            1084.23
h,k,lmax         27,9,20                      27,9,20
Nref             5791[ 3116]                  5558
Tmin,Tmax        0.878,0.979                  0.923,1.000
Tmin'            0.805

Correction method= # Reported T Limits: Tmin=0.923 Tmax=1.000
AbsCorr = MULTI-SCAN

Data completeness= 1.78/0.96        Theta(max)= 75.646

R(reflections)= 0.0330( 5430)                          wR2(reflections)=
                                                       0.0821( 5558)
S = 1.057                    Npar= 326
```

The following ALERTS were generated. Each ALERT has the format
test-name_ALERT_alert-type_alert-level.
Click on the hyperlinks for more details of the test.

Alert level C

```
PLAT220_ALERT_2_C NonSolvent   Resd 1  C   Ueq(max)/Ueq(min) Range        5.9 Ratio
PLAT222_ALERT_3_C NonSolvent Resd 1  H   Uiso(max)/Uiso(min) Range        7.4 Ratio
PLAT242_ALERT_2_C Low    'MainMol' Ueq as Compared to Neighbors of        C13 Check
```

Alert level G

```
PLAT003_ALERT_2_G Number of Uiso or Uij Restrained non-H Atoms ...           8 Report
PLAT007_ALERT_5_G Number of Unrefined Donor-H Atoms .............            4 Report
PLAT186_ALERT_4_G The CIF-Embedded .res File Contains ISOR Records           1 Report
PLAT398_ALERT_2_G Deviating  C-O-C   Angle From 120 for O4       .       108.7 Degree
PLAT398_ALERT_2_G Deviating  C-O-C   Angle From 120 for O6       .       105.3 Degree
PLAT605_ALERT_4_G Largest Solvent Accessible VOID in the Structure         86 A**3
PLAT791_ALERT_4_G Model has Chirality at C1          (Sohnke SpGr)           R Verify
PLAT791_ALERT_4_G Model has Chirality at C2          (Sohnke SpGr)           S Verify
PLAT791_ALERT_4_G Model has Chirality at C3          (Sohnke SpGr)           S Verify
PLAT791_ALERT_4_G Model has Chirality at C7          (Sohnke SpGr)           S Verify
PLAT791_ALERT_4_G Model has Chirality at C8          (Sohnke SpGr)           R Verify
PLAT791_ALERT_4_G Model has Chirality at C9          (Sohnke SpGr)           R Verify
PLAT860_ALERT_3_G Number of Least-Squares Restraints ............          49 Note
PLAT910_ALERT_3_G Missing # of FCF Reflection(s) Below Theta(Min).          1 Note
PLAT912_ALERT_4_G Missing # of FCF Reflections Above STh/L=  0.600         53 Note
PLAT978_ALERT_2_G Number C-C Bonds with Positive Residual Density.          7 Info
```

0 **ALERT level A** = Most likely a serious problem - resolve or explain
0 **ALERT level B** = A potentially serious problem, consider carefully
3 **ALERT level C** = Check. Ensure it is not caused by an omission or oversight
16 **ALERT level G** = General information/check it is not something unexpected

0 ALERT type 1 CIF construction/syntax error, inconsistent or missing data
6 ALERT type 2 Indicator that the structure model may be wrong or deficient
3 ALERT type 3 Indicator that the structure quality may be low
9 ALERT type 4 Improvement, methodology, query or suggestion
1 ALERT type 5 Informative message, check

It is advisable to attempt to resolve as many as possible of the alerts in all categories. Often the minor alerts point to easily fixed oversights, errors and omissions in your CIF or refinement strategy, so attention to these fine details can be worthwhile. In order to resolve some of the more serious problems it may be necessary to carry out additional measurements or structure refinements. However, the purpose of your study may justify the reported deviations and the more serious of these should normally be commented upon in the discussion or experimental section of a paper or in the "special_details" fields of the CIF. checkCIF was carefully designed to identify outliers and unusual parameters, but every test has its limitations and alerts that are not important in a particular case may appear. Conversely, the absence of alerts does not guarantee there are no aspects of the results needing attention. It is up to the individual to critically assess their own results and, if necessary, seek expert advice.

Publication of your CIF in IUCr journals

A basic structural check has been run on your CIF. These basic checks will be run on all CIFs submitted for publication in IUCr journals (*Acta Crystallographica*, *Journal of Applied Crystallography*, *Journal of Synchrotron Radiation*); however, if you intend to submit to *Acta Crystallographica Section C* or *E* or *IUCrData*, you should make sure that full publication checks are run on the final version of your CIF prior to submission.

Publication of your CIF in other journals

Please refer to the *Notes for Authors* of the relevant journal for any special instructions relating to CIF submission.

PLATON version of 18/05/2022; check.def file version of 17/05/2022

Datablock 20200811d - ellipsoid plot

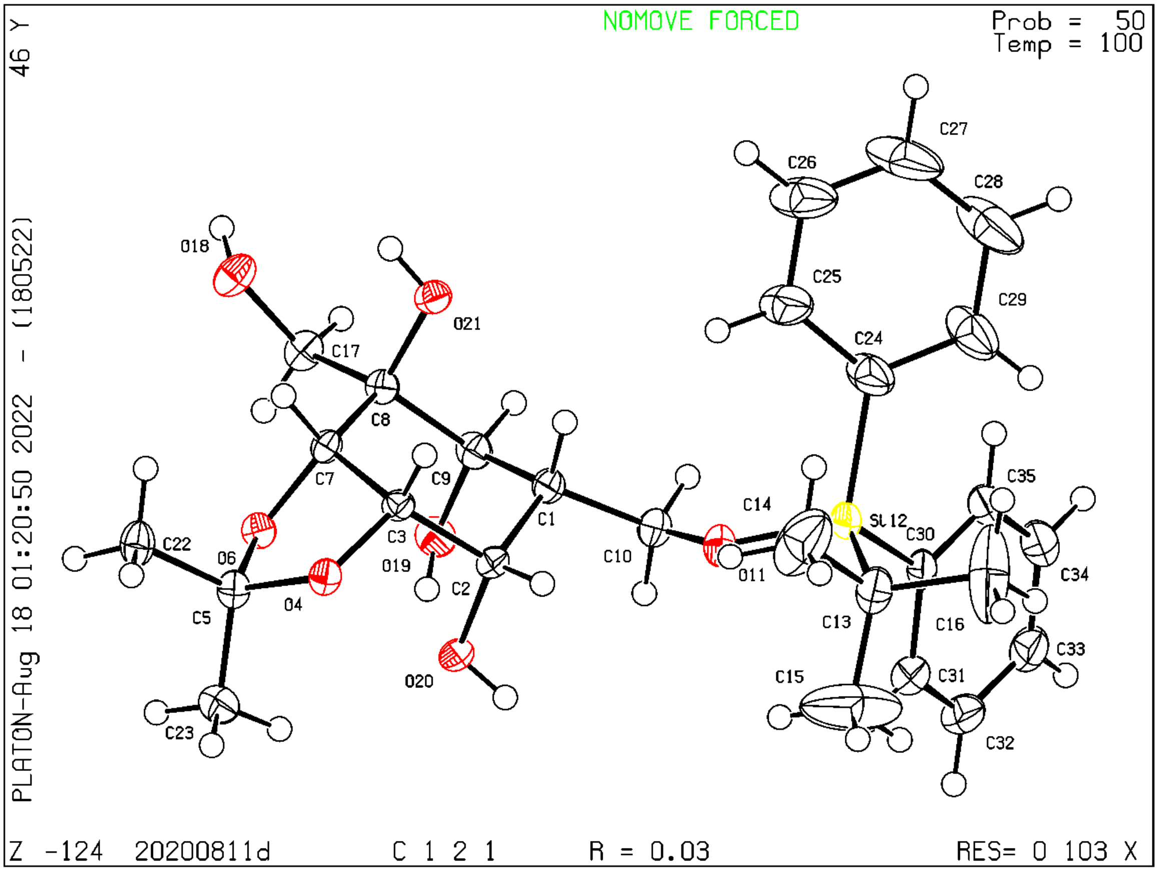

Supplement: Supplementary file 4 — Source Data [file 41467_2024_45037_MOESM4_ESM.zip › Surce Data 20231213/Surce Data New/Crystal Structure Source Data/CCDC-2184298/checkcif.pdf]
