# Supplementary material for: Total syntheses of Tetrodotoxin and 9-epiTetrodotoxin
Source: Nat Commun. 2024 Jan 23;15:679. doi: 10.1038/s41467-024-45037-0 (PMC10806222; doi:10.1038/s41467-024-45037-0)

checkCIF/PLATON report

Structure factors have been supplied for datablock(s) cu_20220427_cph_04_25_coome_0m

THIS REPORT IS FOR GUIDANCE ONLY. IF USED AS PART OF A REVIEW PROCEDURE FOR PUBLICATION, IT SHOULD NOT REPLACE THE EXPERTISE OF AN EXPERIENCED CRYSTALLOGRAPHIC REFEREE.

No syntax errors found.   CIF dictionary   Interpreting this report

Datablock: cu_20220427_cph_04_25_coome_0m

```
Bond precision:   C-C = 0.0039 A          Wavelength=1.54178

Cell:               a=6.0147(1)      b=17.5833(3)      c=23.3786(4)
                    alpha=90         beta=90           gamma=90
Temperature:        193 K

                 Calculated                    Reported
Volume           2472.48(7)                    2472.48(7)
Space group      P 21 21 21                    P 21 21 21
Hall group       P 2ac 2ab                     P 2ac 2ab
Moiety formula   C23 H30 O11 [+ solvent]       C23 H30 O11
Sum formula      C23 H30 O11 [+ solvent]       C23 H30 O11
Mr               482.47                        482.47
Dx,g cm-3        1.296                         1.296
Z                4                             4
Mu (mm-1)        0.879                         0.879
F000             1024.0                        1024.0
F000'            1027.73
h,k,lmax         7,21,28                       7,21,28
Nref             4525[ 2624]                   4521
Tmin,Tmax        0.869,0.892                   0.353,0.467
Tmin'            0.869

Correction method= # Reported T Limits: Tmin=0.353 Tmax=0.467
AbsCorr = MULTI-SCAN

Data completeness= 1.72/1.00       Theta(max)= 68.172

                                                      wR2(reflections)=
R(reflections)= 0.0421( 4310)                         0.1130( 4521)

S = 1.118                    Npar= 315
```

The following ALERTS were generated. Each ALERT has the format

test-name_ALERT_alert-type_alert-level.

Click on the hyperlinks for more details of the test.

Alert level C

```
PLAT911_ALERT_3_C Missing FCF Refl Between Thmin & STh/L=    0.600          3 Report
```

Alert level G

```
PLAT007_ALERT_5_G Number of Unrefined Donor-H Atoms .............           1 Report
PLAT398_ALERT_2_G Deviating  C-O-C   Angle From 120 for O1       .      108.3 Degree
PLAT398_ALERT_2_G Deviating  C-O-C   Angle From 120 for O2       .      108.2 Degree
PLAT398_ALERT_2_G Deviating  C-O-C   Angle From 120 for O3       .       97.8 Degree
PLAT398_ALERT_2_G Deviating  C-O-C   Angle From 120 for O10      .      105.9 Degree
PLAT605_ALERT_4_G Largest Solvent Accessible VOID in the Structure        123 A**3
PLAT791_ALERT_4_G Model has Chirality at C4          (Sohnke SpGr)          S Verify
PLAT791_ALERT_4_G Model has Chirality at C5          (Sohnke SpGr)          S Verify
PLAT791_ALERT_4_G Model has Chirality at C6          (Sohnke SpGr)          S Verify
PLAT791_ALERT_4_G Model has Chirality at C7          (Sohnke SpGr)          S Verify
PLAT791_ALERT_4_G Model has Chirality at C8          (Sohnke SpGr)          S Verify
PLAT791_ALERT_4_G Model has Chirality at C9          (Sohnke SpGr)          R Verify
PLAT791_ALERT_4_G Model has Chirality at C15         (Sohnke SpGr)          S Verify
PLAT791_ALERT_4_G Model has Chirality at C18         (Sohnke SpGr)          R Verify
PLAT909_ALERT_3_G Percentage of I>2sig(I) Data at Theta(Max) Still        86% Note
PLAT910_ALERT_3_G Missing # of FCF Reflection(s) Below Theta(Min).          1 Note
PLAT933_ALERT_2_G Number of HKL-OMIT Records in Embedded .res File          4 Note
PLAT978_ALERT_2_G Number C-C Bonds with Positive Residual Density.          2 Info
```

0 **ALERT level A** = Most likely a serious problem - resolve or explain
0 **ALERT level B** = A potentially serious problem, consider carefully
1 **ALERT level C** = Check. Ensure it is not caused by an omission or oversight
18 **ALERT level G** = General information/check it is not something unexpected

0 ALERT type 1 CIF construction/syntax error, inconsistent or missing data
6 ALERT type 2 Indicator that the structure model may be wrong or deficient
3 ALERT type 3 Indicator that the structure quality may be low
9 ALERT type 4 Improvement, methodology, query or suggestion
1 ALERT type 5 Informative message, check

It is advisable to attempt to resolve as many as possible of the alerts in all categories. Often the minor alerts point to easily fixed oversights, errors and omissions in your CIF or refinement strategy, so attention to these fine details can be worthwhile. In order to resolve some of the more serious problems it may be necessary to carry out additional measurements or structure refinements. However, the purpose of your study may justify the reported deviations and the more serious of these should normally be commented upon in the discussion or experimental section of a paper or in the "special_details" fields of the CIF. checkCIF was carefully designed to identify outliers and unusual parameters, but every test has its limitations and alerts that are not important in a particular case may appear. Conversely, the absence of alerts does not guarantee there are no aspects of the results needing attention. It is up to the individual to critically assess their own results and, if necessary, seek expert advice.

Publication of your CIF in IUCr journals

A basic structural check has been run on your CIF. These basic checks will be run on all CIFs submitted for publication in IUCr journals (*Acta Crystallographica*, *Journal of Applied Crystallography*, *Journal of Synchrotron Radiation*); however, if you intend to submit to *Acta Crystallographica Section C* or *E* or *IUCrData*, you should make sure that full publication checks are run on the final version of your CIF prior to submission.

Publication of your CIF in other journals

Please refer to the *Notes for Authors* of the relevant journal for any special instructions relating to CIF submission.

PLATON version of 19/02/2022; check.def file version of 19/02/2022

Datablock cu_20220427_cph_04_25_coome_0m - ellipsoid plot

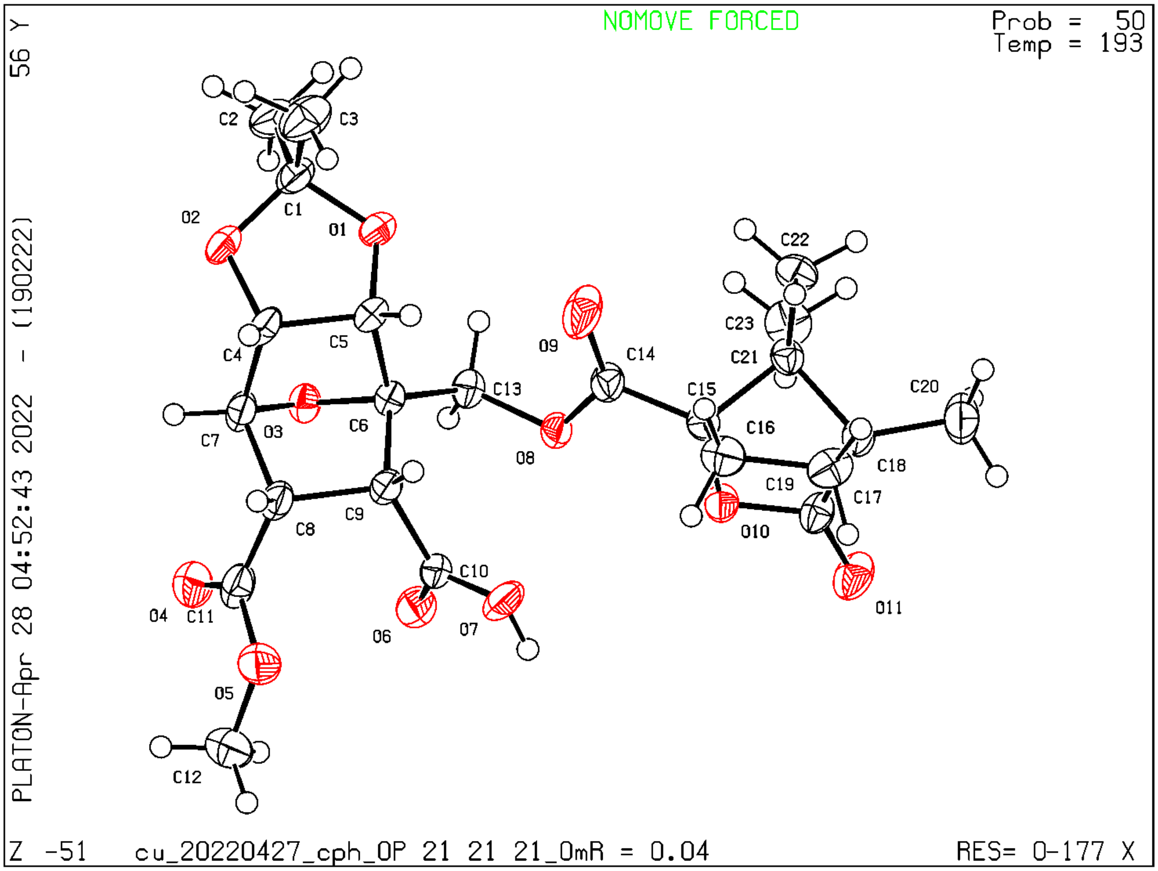

Supplement: Supplementary file 4 — Source Data [file 41467_2024_45037_MOESM4_ESM.zip › Surce Data 20231213/Surce Data New/Crystal Structure Source Data/CCDC-2184304/checkcif.pdf]
