# Supplementary material for: Total syntheses of Tetrodotoxin and 9-epiTetrodotoxin
Source: Nat Commun. 2024 Jan 23;15:679. doi: 10.1038/s41467-024-45037-0 (PMC10806222; doi:10.1038/s41467-024-45037-0)
Supplement: Supplementary file 4 — Source Data [file 41467_2024_45037_MOESM4_ESM.zip › Surce Data 20231213/Surce Data New/Crystal Structure Source Data/CCDC-2184304/cu_20220427_Cph_04_25_COOMe_0m_tables.html]

cu\_20220427\_Cph\_04\_25\_COOMe\_0m


# cu\_20220427\_Cph\_04\_25\_COOMe\_0m

Table 1 Crystal data and structure refinement for cu\_20220427\_Cph\_04\_25\_COOMe\_0m.

| Identification code | cu\_20220427\_Cph\_04\_25\_COOMe\_0m |
| Empirical formula | C23H30O11 |
| Formula weight | 482.47 |
| Temperature/K | 193.00 |
| Crystal system | orthorhombic |
| Space group | P212121 |
| a/Å | 6.01470(10) |
| b/Å | 17.5833(3) |
| c/Å | 23.3786(4) |
| α/° | 90 |
| β/° | 90 |
| γ/° | 90 |
| Volume/Å3 | 2472.48(7) |
| Z | 4 |
| ρcalcg/cm3 | 1.296 |
| μ/mm‑1 | 0.879 |
| F(000) | 1024.0 |
| Crystal size/mm3 | 0.16 × 0.15 × 0.13 |
| Radiation | CuKα (λ = 1.54178) |
| 2Θ range for data collection/° | 7.562 to 136.344 |
| Index ranges | -7 ≤ h ≤ 7, -21 ≤ k ≤ 21, -28 ≤ l ≤ 28 |
| Reflections collected | 46180 |
| Independent reflections | 4521 [Rint = 0.0611, Rsigma = 0.0282] |
| Data/restraints/parameters | 4521/0/315 |
| Goodness-of-fit on F2 | 1.118 |
| Final R indexes [I>=2σ (I)] | R1 = 0.0421, wR2 = 0.1115 |
| Final R indexes [all data] | R1 = 0.0437, wR2 = 0.1130 |
| Largest diff. peak/hole / e Å-3 | 0.27/-0.31 || Flack parameter | 0.07(5) |

Table 2 Fractional Atomic Coordinates (×104) and Equivalent Isotropic Displacement Parameters (Å2×103) for cu\_20220427\_Cph\_04\_25\_COOMe\_0m. Ueq is defined as 1/3 of the trace of the orthogonalised UIJ tensor.

| Atom | *x* | *y* | *z* | U(eq) |
| --- | --- | --- | --- | --- |
| O1 | 7174(3) | 2154.1(10) | 3541.1(8) | 31.1(4) |
| O2 | 6292(4) | 1301.2(10) | 2857.6(8) | 36.4(5) |
| O3 | 3420(3) | 2625.9(10) | 2875.8(8) | 27.5(4) |
| O4 | 683(4) | 2914.4(12) | 1800.7(10) | 44.4(5) |
| O5 | 2720(4) | 3770.1(12) | 1325.4(9) | 42.3(5) |
| O6 | 2303(4) | 4400.4(11) | 2480.0(9) | 37.5(5) |
| O7 | 5668(4) | 4927.1(10) | 2349.7(10) | 40.6(5) |
| O8 | 5483(3) | 4313.0(10) | 3589.8(7) | 26.4(4) |
| O9 | 8877(4) | 3858.5(13) | 3780.3(13) | 58.7(7) |
| O10 | 6453(3) | 5716.3(10) | 3820.4(8) | 30.0(4) |
| O11 | 5947(3) | 6904.2(12) | 4147.0(10) | 42.7(5) |
| C1 | 7397(5) | 1368.0(15) | 3399.7(12) | 32.5(6) |
| C2 | 6108(5) | 907.0(17) | 3834.0(14) | 40.7(7) |
| C3 | 9811(6) | 1141.2(19) | 3342.1(17) | 48.5(8) |
| C4 | 6579(5) | 2000.0(14) | 2553.3(12) | 29.7(6) |
| C5 | 7231(4) | 2582.5(14) | 3024.8(11) | 25.8(5) |
| C6 | 5230(4) | 3131.7(14) | 3001.3(11) | 23.8(5) |
| C7 | 4319(5) | 2296.4(14) | 2363.0(11) | 30.3(6) |
| C8 | 4640(5) | 3002.8(15) | 1983.3(11) | 30.4(6) |
| C9 | 5546(5) | 3596.0(13) | 2433.4(10) | 26.4(5) |
| C10 | 4294(5) | 4343.3(14) | 2426.9(11) | 28.6(5) |
| C11 | 2429(5) | 3210.6(15) | 1705.6(12) | 34.7(6) |
| C12 | 670(7) | 4026(2) | 1067.4(16) | 54.6(9) |
| C13 | 4611(4) | 3545.3(14) | 3545.3(11) | 26.9(5) |
| C14 | 7605(5) | 4381.5(15) | 3733.9(12) | 31.3(6) |
| C15 | 8331(4) | 5184.5(15) | 3850.5(11) | 26.6(5) |
| C16 | 10098(5) | 5507.7(17) | 3450.1(12) | 35.5(6) |
| C17 | 10822(5) | 6225.5(16) | 3775.8(13) | 34.6(6) |
| C18 | 9437(4) | 6208.5(15) | 4335.0(11) | 28.5(6) |
| C19 | 7110(4) | 6351.8(15) | 4115.2(12) | 28.8(6) |
| C20 | 10198(6) | 6739.4(19) | 4803.7(16) | 46.0(8) |
| C21 | 9278(4) | 5341.3(14) | 4459.2(11) | 25.3(5) |
| C22 | 11502(5) | 4964.1(17) | 4581.1(13) | 35.0(6) |
| C23 | 7635(5) | 5146.2(19) | 4940.8(13) | 40.6(7) |

Table 3 Anisotropic Displacement Parameters (Å2×103) for cu\_20220427\_Cph\_04\_25\_COOMe\_0m. The Anisotropic displacement factor exponent takes the form: -2π2[h2a\*2U11+2hka\*b\*U12+…].

| Atom | U11 | U22 | U33 | U23 | U13 | U12 |
| --- | --- | --- | --- | --- | --- | --- |
| O1 | 41.7(11) | 21.0(9) | 30.7(9) | 1.9(7) | -2.1(8) | 2.4(8) |
| O2 | 53.6(13) | 16.6(9) | 38.8(10) | 1.0(8) | -3.0(9) | -2.0(8) |
| O3 | 28.1(9) | 24.8(9) | 29.7(9) | -2.5(7) | 0.3(7) | -5.6(7) |
| O4 | 48.4(13) | 37.6(11) | 47.1(12) | 0.8(9) | -7.8(10) | -11.1(10) |
| O5 | 53.6(13) | 38.8(11) | 34.3(10) | 6.0(8) | -5.2(9) | -6.6(10) |
| O6 | 38.4(12) | 32.3(10) | 41.8(11) | -5.1(8) | -2.9(9) | 6.5(8) |
| O7 | 48.0(12) | 18.7(9) | 55.2(13) | 4.4(8) | -4.1(11) | -0.3(9) |
| O8 | 26.1(9) | 22.6(9) | 30.4(9) | -5.8(7) | -3.9(7) | 2.3(7) |
| O9 | 36.6(12) | 33.1(11) | 106(2) | -23.5(13) | -24.0(13) | 10.2(10) |
| O10 | 22.4(9) | 26.3(9) | 41.3(10) | -8.6(8) | -6.4(7) | 5.6(7) |
| O11 | 30.6(10) | 29.7(10) | 67.9(15) | -11.9(10) | -1.8(10) | 7.6(9) |
| C1 | 36.7(14) | 19.9(12) | 40.9(15) | 0.7(11) | -0.6(12) | 3.9(11) |
| C2 | 41.6(16) | 31.1(14) | 49.4(17) | 10.9(13) | 1.0(14) | -1.5(12) |
| C3 | 37.7(17) | 36.0(16) | 72(2) | 3.5(15) | 6.6(16) | 8.4(13) |
| C4 | 40.9(14) | 15.6(11) | 32.7(13) | -1.5(10) | 6.0(11) | -1.9(10) |
| C5 | 27.9(13) | 18.4(11) | 31.1(13) | 0.4(9) | 2.9(10) | 0.5(10) |
| C6 | 26.0(12) | 19.7(12) | 25.8(12) | -1.4(9) | 0.8(9) | -1.5(10) |
| C7 | 40.4(15) | 19.9(12) | 30.6(13) | -5.4(10) | 1.1(12) | -3.9(11) |
| C8 | 44.2(15) | 21.6(12) | 25.4(13) | -3.1(10) | 2.7(11) | -2.1(11) |
| C9 | 33.3(13) | 20.0(12) | 25.9(12) | -0.9(9) | 1.8(10) | -0.6(10) |
| C10 | 38.7(15) | 21.5(12) | 25.6(12) | -3.6(10) | -4.8(11) | 2.3(11) |
| C11 | 48.0(17) | 24.6(12) | 31.6(14) | -2.8(10) | 6.4(13) | -9.3(13) |
| C12 | 65(2) | 49.0(19) | 49.8(19) | 10.9(15) | -23.2(18) | -5.6(18) |
| C13 | 26.6(12) | 24.9(12) | 29.2(13) | -4.4(10) | 2.3(10) | -0.9(10) |
| C14 | 28.6(13) | 27.4(13) | 38.0(14) | -9.6(11) | -5.2(11) | 5.9(11) |
| C15 | 19.4(11) | 27.8(12) | 32.7(13) | -8.2(10) | -4.4(10) | 6.9(10) |
| C16 | 34.9(14) | 40.6(16) | 31.0(14) | -1.4(12) | 4.8(11) | 9.0(12) |
| C17 | 23.1(12) | 34.1(14) | 46.4(16) | 5.5(12) | 5.5(12) | 1.8(11) |
| C18 | 23.5(12) | 26.7(13) | 35.4(14) | -5.5(11) | -1.2(11) | -2.1(10) |
| C19 | 23.6(12) | 24.7(12) | 38.1(14) | -5.0(11) | 3.5(11) | -0.8(10) |
| C20 | 42.9(17) | 38.4(16) | 57(2) | -17.4(15) | -10.7(15) | -1.8(13) |
| C21 | 20.8(12) | 27.4(12) | 27.7(13) | -3.7(10) | -1.2(10) | 0.2(10) |
| C22 | 26.3(13) | 38.0(15) | 40.8(15) | 1.5(12) | -7.9(12) | 4.3(11) |
| C23 | 39.2(16) | 47.3(17) | 35.1(15) | 1.8(12) | 9.4(13) | -2.4(14) |

Table 4 Bond Lengths for cu\_20220427\_Cph\_04\_25\_COOMe\_0m.

| Atom | Atom | Length/Å |  | Atom | Atom | Length/Å |
| --- | --- | --- | --- | --- | --- | --- |
| O1 | C1 | 1.427(3) |  | C4 | C5 | 1.555(3) |
| O1 | C5 | 1.423(3) |  | C4 | C7 | 1.522(4) |
| O2 | C1 | 1.436(3) |  | C5 | C6 | 1.544(3) |
| O2 | C4 | 1.430(3) |  | C6 | C9 | 1.570(3) |
| O3 | C6 | 1.436(3) |  | C6 | C13 | 1.512(3) |
| O3 | C7 | 1.437(3) |  | C7 | C8 | 1.539(4) |
| O4 | C11 | 1.193(4) |  | C8 | C9 | 1.579(3) |
| O5 | C11 | 1.337(3) |  | C8 | C11 | 1.524(4) |
| O5 | C12 | 1.445(4) |  | C9 | C10 | 1.515(3) |
| O6 | C10 | 1.208(4) |  | C14 | C15 | 1.503(4) |
| O7 | C10 | 1.330(3) |  | C15 | C16 | 1.526(4) |
| O8 | C13 | 1.452(3) |  | C15 | C21 | 1.557(3) |
| O8 | C14 | 1.326(3) |  | C16 | C17 | 1.537(4) |
| O9 | C14 | 1.201(3) |  | C17 | C18 | 1.550(4) |
| O10 | C15 | 1.468(3) |  | C18 | C19 | 1.512(4) |
| O10 | C19 | 1.371(3) |  | C18 | C20 | 1.510(4) |
| O11 | C19 | 1.199(3) |  | C18 | C21 | 1.555(4) |
| C1 | C2 | 1.513(4) |  | C21 | C22 | 1.520(4) |
| C1 | C3 | 1.512(4) |  | C21 | C23 | 1.537(4) |

Table 5 Bond Angles for cu\_20220427\_Cph\_04\_25\_COOMe\_0m.

| Atom | Atom | Atom | Angle/˚ |  | Atom | Atom | Atom | Angle/˚ |
| --- | --- | --- | --- | --- | --- | --- | --- | --- |
| C5 | O1 | C1 | 108.29(19) |  | O6 | C10 | O7 | 124.4(2) |
| C4 | O2 | C1 | 108.2(2) |  | O6 | C10 | C9 | 124.3(3) |
| C6 | O3 | C7 | 97.75(18) |  | O7 | C10 | C9 | 111.2(2) |
| C11 | O5 | C12 | 113.3(3) |  | O4 | C11 | O5 | 124.1(3) |
| C14 | O8 | C13 | 116.8(2) |  | O4 | C11 | C8 | 125.7(2) |
| C19 | O10 | C15 | 105.86(19) |  | O5 | C11 | C8 | 110.2(2) |
| O1 | C1 | O2 | 103.9(2) |  | O8 | C13 | C6 | 114.7(2) |
| O1 | C1 | C2 | 108.4(2) |  | O8 | C14 | C15 | 114.3(2) |
| O1 | C1 | C3 | 111.5(3) |  | O9 | C14 | O8 | 124.5(3) |
| O2 | C1 | C2 | 108.2(2) |  | O9 | C14 | C15 | 121.2(2) |
| O2 | C1 | C3 | 110.1(3) |  | O10 | C15 | C14 | 111.5(2) |
| C3 | C1 | C2 | 114.2(3) |  | O10 | C15 | C16 | 105.6(2) |
| O2 | C4 | C5 | 104.1(2) |  | O10 | C15 | C21 | 102.27(18) |
| O2 | C4 | C7 | 109.4(2) |  | C14 | C15 | C16 | 116.1(2) |
| C7 | C4 | C5 | 101.9(2) |  | C14 | C15 | C21 | 116.0(2) |
| O1 | C5 | C4 | 104.26(19) |  | C16 | C15 | C21 | 103.9(2) |
| O1 | C5 | C6 | 110.0(2) |  | C15 | C16 | C17 | 101.5(2) |
| C6 | C5 | C4 | 101.0(2) |  | C16 | C17 | C18 | 104.5(2) |
| O3 | C6 | C5 | 102.17(19) |  | C17 | C18 | C21 | 102.1(2) |
| O3 | C6 | C9 | 103.95(19) |  | C19 | C18 | C17 | 102.0(2) |
| O3 | C6 | C13 | 106.5(2) |  | C19 | C18 | C21 | 99.8(2) |
| C5 | C6 | C9 | 105.1(2) |  | C20 | C18 | C17 | 115.9(2) |
| C13 | C6 | C5 | 117.6(2) |  | C20 | C18 | C19 | 115.1(2) |
| C13 | C6 | C9 | 119.4(2) |  | C20 | C18 | C21 | 119.3(2) |
| O3 | C7 | C4 | 103.3(2) |  | O10 | C19 | C18 | 107.6(2) |
| O3 | C7 | C8 | 101.7(2) |  | O11 | C19 | O10 | 121.5(2) |
| C4 | C7 | C8 | 109.5(2) |  | O11 | C19 | C18 | 130.8(3) |
| C7 | C8 | C9 | 101.1(2) |  | C18 | C21 | C15 | 91.46(19) |
| C11 | C8 | C7 | 109.3(2) |  | C22 | C21 | C15 | 114.6(2) |
| C11 | C8 | C9 | 115.3(2) |  | C22 | C21 | C18 | 114.1(2) |
| C6 | C9 | C8 | 100.27(19) |  | C22 | C21 | C23 | 109.3(2) |
| C10 | C9 | C6 | 113.5(2) |  | C23 | C21 | C15 | 113.2(2) |
| C10 | C9 | C8 | 113.2(2) |  | C23 | C21 | C18 | 113.3(2) |

Table 6 Torsion Angles for cu\_20220427\_Cph\_04\_25\_COOMe\_0m.

| A | B | C | D | Angle/˚ |  | A | B | C | D | Angle/˚ |
| --- | --- | --- | --- | --- | --- | --- | --- | --- | --- | --- |
| O1 | C5 | C6 | O3 | -74.5(2) |  | C7 | C8 | C9 | C6 | -7.7(3) |
| O1 | C5 | C6 | C9 | 177.20(19) |  | C7 | C8 | C9 | C10 | -129.0(2) |
| O1 | C5 | C6 | C13 | 41.6(3) |  | C7 | C8 | C11 | O4 | 5.6(4) |
| O2 | C4 | C5 | O1 | -1.7(3) |  | C7 | C8 | C11 | O5 | -173.2(2) |
| O2 | C4 | C5 | C6 | -115.8(2) |  | C8 | C9 | C10 | O6 | 54.4(3) |
| O2 | C4 | C7 | O3 | 78.0(2) |  | C8 | C9 | C10 | O7 | -125.0(2) |
| O2 | C4 | C7 | C8 | -174.2(2) |  | C9 | C6 | C13 | O8 | -31.8(3) |
| O3 | C6 | C9 | C8 | -27.1(2) |  | C9 | C8 | C11 | O4 | -107.4(3) |
| O3 | C6 | C9 | C10 | 94.0(2) |  | C9 | C8 | C11 | O5 | 73.9(3) |
| O3 | C6 | C13 | O8 | -148.9(2) |  | C11 | C8 | C9 | C6 | 109.9(2) |
| O3 | C7 | C8 | C9 | 40.4(3) |  | C11 | C8 | C9 | C10 | -11.4(3) |
| O3 | C7 | C8 | C11 | -81.5(2) |  | C12 | O5 | C11 | O4 | 4.4(4) |
| O8 | C14 | C15 | O10 | 4.4(3) |  | C12 | O5 | C11 | C8 | -176.8(2) |
| O8 | C14 | C15 | C16 | -116.7(3) |  | C13 | O8 | C14 | O9 | 6.7(4) |
| O8 | C14 | C15 | C21 | 120.9(2) |  | C13 | O8 | C14 | C15 | -172.1(2) |
| O9 | C14 | C15 | O10 | -174.5(3) |  | C13 | C6 | C9 | C8 | -145.5(2) |
| O9 | C14 | C15 | C16 | 64.4(4) |  | C13 | C6 | C9 | C10 | -24.4(3) |
| O9 | C14 | C15 | C21 | -58.0(4) |  | C14 | O8 | C13 | C6 | -78.0(3) |
| O10 | C15 | C16 | C17 | 69.2(2) |  | C14 | C15 | C16 | C17 | -166.7(2) |
| O10 | C15 | C21 | C18 | -52.7(2) |  | C14 | C15 | C21 | C18 | -174.3(2) |
| O10 | C15 | C21 | C22 | -170.2(2) |  | C14 | C15 | C21 | C22 | 68.3(3) |
| O10 | C15 | C21 | C23 | 63.5(3) |  | C14 | C15 | C21 | C23 | -58.1(3) |
| C1 | O1 | C5 | C4 | 21.6(3) |  | C15 | O10 | C19 | O11 | 177.1(3) |
| C1 | O1 | C5 | C6 | 129.1(2) |  | C15 | O10 | C19 | C18 | -0.3(3) |
| C1 | O2 | C4 | C5 | -18.7(3) |  | C15 | C16 | C17 | C18 | 2.0(3) |
| C1 | O2 | C4 | C7 | -127.0(2) |  | C16 | C15 | C21 | C18 | 57.0(2) |
| C4 | O2 | C1 | O1 | 32.2(3) |  | C16 | C15 | C21 | C22 | -60.5(3) |
| C4 | O2 | C1 | C2 | 147.2(2) |  | C16 | C15 | C21 | C23 | 173.2(2) |
| C4 | O2 | C1 | C3 | -87.3(3) |  | C16 | C17 | C18 | C19 | -68.6(3) |
| C4 | C5 | C6 | O3 | 35.2(2) |  | C16 | C17 | C18 | C20 | 165.6(2) |
| C4 | C5 | C6 | C9 | -73.1(2) |  | C16 | C17 | C18 | C21 | 34.3(3) |
| C4 | C5 | C6 | C13 | 151.3(2) |  | C17 | C18 | C19 | O10 | 70.2(2) |
| C4 | C7 | C8 | C9 | -68.4(3) |  | C17 | C18 | C19 | O11 | -106.8(3) |
| C4 | C7 | C8 | C11 | 169.7(2) |  | C17 | C18 | C21 | C15 | -53.9(2) |
| C5 | O1 | C1 | O2 | -33.4(3) |  | C17 | C18 | C21 | C22 | 64.0(3) |
| C5 | O1 | C1 | C2 | -148.3(2) |  | C17 | C18 | C21 | C23 | -170.1(2) |
| C5 | O1 | C1 | C3 | 85.2(3) |  | C19 | O10 | C15 | C14 | 159.9(2) |
| C5 | C4 | C7 | O3 | -31.7(2) |  | C19 | O10 | C15 | C16 | -73.1(2) |
| C5 | C4 | C7 | C8 | 76.0(2) |  | C19 | O10 | C15 | C21 | 35.3(2) |
| C5 | C6 | C9 | C8 | 79.9(2) |  | C19 | C18 | C21 | C15 | 50.7(2) |
| C5 | C6 | C9 | C10 | -159.0(2) |  | C19 | C18 | C21 | C22 | 168.6(2) |
| C5 | C6 | C13 | O8 | 97.4(3) |  | C19 | C18 | C21 | C23 | -65.5(3) |
| C6 | O3 | C7 | C4 | 54.8(2) |  | C20 | C18 | C19 | O10 | -163.5(3) |
| C6 | O3 | C7 | C8 | -58.7(2) |  | C20 | C18 | C19 | O11 | 19.5(4) |
| C6 | C9 | C10 | O6 | -59.1(3) |  | C20 | C18 | C21 | C15 | 176.9(2) |
| C6 | C9 | C10 | O7 | 121.5(2) |  | C20 | C18 | C21 | C22 | -65.3(3) |
| C7 | O3 | C6 | C5 | -55.8(2) |  | C20 | C18 | C21 | C23 | 60.7(4) |
| C7 | O3 | C6 | C9 | 53.4(2) |  | C21 | C15 | C16 | C17 | -38.0(2) |
| C7 | O3 | C6 | C13 | -179.7(2) |  | C21 | C18 | C19 | O10 | -34.5(3) |
| C7 | C4 | C5 | O1 | 112.1(2) |  | C21 | C18 | C19 | O11 | 148.5(3) |
| C7 | C4 | C5 | C6 | -2.1(2) |  |  |  |  |  |  |

Table 7 Hydrogen Atom Coordinates (Å×104) and Isotropic Displacement Parameters (Å2×103) for cu\_20220427\_Cph\_04\_25\_COOMe\_0m.

| Atom | *x* | *y* | *z* | U(eq) |
| --- | --- | --- | --- | --- |
| H7 | 4929.34 | 5331.59 | 2327.88 | 61 |
| H2A | 6802.53 | 961.08 | 4211.05 | 61 |
| H2B | 6115.48 | 370.12 | 3721.37 | 61 |
| H2C | 4571.57 | 1091.03 | 3851.07 | 61 |
| H3A | 9906.4 | 628.47 | 3179.49 | 73 |
| H3B | 10517.14 | 1146.69 | 3719.84 | 73 |
| H3C | 10574.76 | 1501.29 | 3089.58 | 73 |
| H4 | 7687.37 | 1965.76 | 2235.44 | 36 |
| H5 | 8692.16 | 2837.2 | 2953.92 | 31 |
| H7A | 3345.47 | 1898.27 | 2188.26 | 36 |
| H8 | 5787.66 | 2901.15 | 1683.53 | 36 |
| H9 | 7164.35 | 3690.03 | 2365.11 | 32 |
| H12A | 988.04 | 4434.73 | 794.48 | 82 |
| H12B | -330.84 | 4216.13 | 1365.81 | 82 |
| H12C | -39.48 | 3600.76 | 867.06 | 82 |
| H13A | 2969.77 | 3567.57 | 3572.52 | 32 |
| H13B | 5156.72 | 3246.05 | 3875.44 | 32 |
| H16A | 9466.61 | 5635.23 | 3071.01 | 43 |
| H16B | 11352.21 | 5149.04 | 3399.94 | 43 |
| H17A | 10491.61 | 6688.95 | 3550.9 | 41 |
| H17B | 12433.42 | 6211.24 | 3861 | 41 |
| H20A | 10124.03 | 7265.69 | 4666.98 | 69 |
| H20B | 11733.06 | 6616.72 | 4909.84 | 69 |
| H20C | 9231.63 | 6680.28 | 5138.17 | 69 |
| H22A | 12543.41 | 5079.47 | 4270.55 | 53 |
| H22B | 11297.02 | 4412.41 | 4608.27 | 53 |
| H22C | 12099.64 | 5157.38 | 4942.94 | 53 |
| H23A | 8252.23 | 5310.36 | 5308.54 | 61 |
| H23B | 7382.94 | 4595.7 | 4948.47 | 61 |
| H23C | 6221.59 | 5408.29 | 4872.39 | 61 |

Table 8 Solvent masks information for cu\_20220427\_Cph\_04\_25\_COOMe\_0m.

| Number | X | Y | Z | Volume | Electron count | Content |
| --- | --- | --- | --- | --- | --- | --- |
| 1 | -0.374 | 0.250 | 0.500 | 132.8 | 49.2 | ? |
| 2 | -0.392 | 0.750 | 0.000 | 132.8 | 49.2 | ? |

Experimental

Single crystals of C23H30O11
[cu\_20220427\_Cph\_04\_25\_COOMe\_0m]
were
[].
A suitable crystal was selected and
[]
on a
Bruker D8 VENTURE
diffractometer. The crystal was kept at 193.00 K during data collection.
Using Olex2 [1], the structure was solved with the
Unknown
[2] structure solution program using
Unknown
and refined with the
Unknown
[3] refinement package using
Unknown
minimisation.

1. Dolomanov, O.V., Bourhis, L.J., Gildea, R.J, Howard, J.A.K. & Puschmann, H.
   (2009), J. Appl. Cryst. 42, 339-341.

Crystal structure determination of
[cu\_20220427\_Cph\_04\_25\_COOMe\_0m]

**Crystal Data**
for C23H30O11 (*M*=482.47 g/mol):
orthorhombic, space group P212121 (no. 19),
*a* = 6.01470(10) Å, *b* = 17.5833(3) Å, *c* = 23.3786(4) Å,
*V*= 2472.48(7) Å3,
*Z* = 4,
*T* = 193.00 K,
μ(CuKα) = 0.879 mm-1,
*Dcalc* = 1.296 g/cm3,
46180 reflections measured (7.562° ≤ 2Θ ≤ 136.344°),
4521 unique (*R*int = 0.0611, Rsigma = 0.0282) which were used in all calculations.
The final *R*1 was 0.0421
(I > 2σ(I)) and *wR*2 was 0.1130 (all data).

Refinement model description

Number of restraints - 0,
number of constraints - unknown.

Details:

```
1.a Ternary CH refined with riding coordinates:
```

This report has been created with Olex2, compiled on
2022.04.07 svn.rca3783a0 for OlexSys. Please
let us know
if there are any errors or if you would like to have additional features.
