# Supplementary material for: Total syntheses of Tetrodotoxin and 9-epiTetrodotoxin
Source: Nat Commun. 2024 Jan 23;15:679. doi: 10.1038/s41467-024-45037-0 (PMC10806222; doi:10.1038/s41467-024-45037-0)

checkCIF/PLATON report

Structure factors have been supplied for datablock(s) cu_20220627_zh_wj_06_24_ome_0m

THIS REPORT IS FOR GUIDANCE ONLY. IF USED AS PART OF A REVIEW PROCEDURE FOR PUBLICATION, IT SHOULD NOT REPLACE THE EXPERTISE OF AN EXPERIENCED CRYSTALLOGRAPHIC REFEREE.

No syntax errors found. CIF dictionary Interpreting this report

Datablock: cu_20220627_zh_wj_06_24_ome_0m

```
Bond precision:   C-C = 0.0070 A          Wavelength=1.54178

Cell:           a=8.3171(2)       b=10.4531(3)      c=16.3414(5)
                alpha=84.815(2)   beta=83.036(2)    gamma=67.740(1)
Temperature:    198 K
```

	Calculated	Reported
Volume	1303.61(6)	1303.61(6)
Space group	P 1	P 1
Hall group	P 1	P 1
Moiety formula	C26 H33 N3 O8	C26 H33 N3 O8
Sum formula	C26 H33 N3 O8	C26 H33 N3 O8
Mr	515.55	515.55
Dx,g cm-3	1.313	1.313
Z	2	2
Mu (mm-1)	0.815	0.815
F000	548.0	548.0
F000'	549.83	
h,k,lmax	10,12,19	10,12,19
Nref	9580[4790]	28420
Tmin,Tmax	0.889,0.922	0.662,0.753
Tmin'	0.885	

```
Correction method= # Reported T Limits: Tmin=0.662 Tmax=0.753
AbsCorr = MULTI-SCAN

Data completeness= 5.93/2.97        Theta(max)= 68.383

R(reflections)= 0.0476( 24581)               wR2(reflections)= 0.1245( 28420)

S = 1.039                    Npar= 680
```

The following ALERTS were generated. Each ALERT has the format
test-name_ALERT_alert-type_alert-level.
Click on the hyperlinks for more details of the test.

Alert level C

```
PLAT089_ALERT_3_C Poor Data / Parameter Ratio (Zmax < 18) ........       7.01 Note
PLAT338_ALERT_4_C Small Aver Tau in Cyclohexane C4       -C11           34.12 Degree
PLAT338_ALERT_4_C Small Aver Tau in Cyclohexane C31      -C44           34.75 Degree
PLAT340_ALERT_3_C Low Bond Precision on  C-C Bonds ..............       0.007 Ang.
PLAT790_ALERT_4_C Centre of Gravity not Within Unit Cell: Resd.  #          1 Note
              C26 H33 N3 O8
PLAT911_ALERT_3_C Missing FCF Refl Between Thmin & STh/L=    0.600          9 Report
PLAT992_ALERT_5_C Repd & Actual _reflns_number_gt Values Differ by         15 Check
```

Alert level G

```
PLAT398_ALERT_2_G Deviating  C-O-C   Angle From 120 for O1       .      105.0 Degree
PLAT398_ALERT_2_G Deviating  C-O-C   Angle From 120 for O2       .      108.8 Degree
PLAT398_ALERT_2_G Deviating  C-O-C   Angle From 120 for O3       .      109.4 Degree
PLAT398_ALERT_2_G Deviating  C-O-C   Angle From 120 for O4       .      105.9 Degree
PLAT398_ALERT_2_G Deviating  C-O-C   Angle From 120 for O6       .      106.8 Degree
PLAT398_ALERT_2_G Deviating  C-O-C   Angle From 120 for O9       .      109.0 Degree
PLAT398_ALERT_2_G Deviating  C-O-C   Angle From 120 for O10      .      105.8 Degree
PLAT398_ALERT_2_G Deviating  C-O-C   Angle From 120 for O11      .      105.3 Degree
PLAT398_ALERT_2_G Deviating  C-O-C   Angle From 120 for O12      .      108.7 Degree
PLAT398_ALERT_2_G Deviating  C-O-C   Angle From 120 for O13      .      106.8 Degree
PLAT791_ALERT_4_G Model has Chirality at C4         (Sohnke SpGr)          S Verify
PLAT791_ALERT_4_G Model has Chirality at C5         (Sohnke SpGr)          S Verify
PLAT791_ALERT_4_G Model has Chirality at C6         (Sohnke SpGr)          R Verify
PLAT791_ALERT_4_G Model has Chirality at C11        (Sohnke SpGr)          R Verify
PLAT791_ALERT_4_G Model has Chirality at C12        (Sohnke SpGr)          R Verify
PLAT791_ALERT_4_G Model has Chirality at C13        (Sohnke SpGr)          R Verify
PLAT791_ALERT_4_G Model has Chirality at C14        (Sohnke SpGr)          R Verify
PLAT791_ALERT_4_G Model has Chirality at C17        (Sohnke SpGr)          S Verify
PLAT791_ALERT_4_G Model has Chirality at C31        (Sohnke SpGr)          R Verify
PLAT791_ALERT_4_G Model has Chirality at C32        (Sohnke SpGr)          S Verify
PLAT791_ALERT_4_G Model has Chirality at C33        (Sohnke SpGr)          S Verify
PLAT791_ALERT_4_G Model has Chirality at C37        (Sohnke SpGr)          R Verify
PLAT791_ALERT_4_G Model has Chirality at C38        (Sohnke SpGr)          R Verify
PLAT791_ALERT_4_G Model has Chirality at C41        (Sohnke SpGr)          S Verify
PLAT791_ALERT_4_G Model has Chirality at C43        (Sohnke SpGr)          R Verify
PLAT791_ALERT_4_G Model has Chirality at C44        (Sohnke SpGr)          R Verify
PLAT870_ALERT_4_G ALERTS Related to Twinning Effects Suppressed ..          ! Info
PLAT912_ALERT_4_G Missing # of FCF Reflections Above STh/L=  0.600         12 Note
PLAT913_ALERT_3_G Missing # of Very Strong Reflections in FCF ....          1 Note
PLAT916_ALERT_2_G Hooft y and Flack x Parameter Values Differ by .       0.18 Check
```

0 **ALERT level A** = Most likely a serious problem - resolve or explain
0 **ALERT level B** = A potentially serious problem, consider carefully
7 **ALERT level C** = Check. Ensure it is not caused by an omission or oversight
30 **ALERT level G** = General information/check it is not something unexpected

0 ALERT type 1 CIF construction/syntax error, inconsistent or missing data

```
11 ALERT type 2 Indicator that the structure model may be wrong or deficient
 4 ALERT type 3 Indicator that the structure quality may be low
21 ALERT type 4 Improvement, methodology, query or suggestion
 1 ALERT type 5 Informative message, check
```

It is advisable to attempt to resolve as many as possible of the alerts in all categories. Often the minor alerts point to easily fixed oversights, errors and omissions in your CIF or refinement strategy, so attention to these fine details can be worthwhile. In order to resolve some of the more serious problems it may be necessary to carry out additional measurements or structure refinements. However, the purpose of your study may justify the reported deviations and the more serious of these should normally be commented upon in the discussion or experimental section of a paper or in the "special_details" fields of the CIF. checkCIF was carefully designed to identify outliers and unusual parameters, but every test has its limitations and alerts that are not important in a particular case may appear. Conversely, the absence of alerts does not guarantee there are no aspects of the results needing attention. It is up to the individual to critically assess their own results and, if necessary, seek expert advice.

Publication of your CIF in IUCr journals

A basic structural check has been run on your CIF. These basic checks will be run on all CIFs submitted for publication in IUCr journals (*Acta Crystallographica*, *Journal of Applied Crystallography*, *Journal of Synchrotron Radiation*); however, if you intend to submit to *Acta Crystallographica Section C* or *E* or *IUCrData*, you should make sure that full publication checks are run on the final version of your CIF prior to submission.

Publication of your CIF in other journals

Please refer to the *Notes for Authors* of the relevant journal for any special instructions relating to CIF submission.

PLATON version of 18/05/2022; check.def file version of 17/05/2022

Datablock cu_20220627_zh_wj_06_24_ome_0m - ellipsoid plot

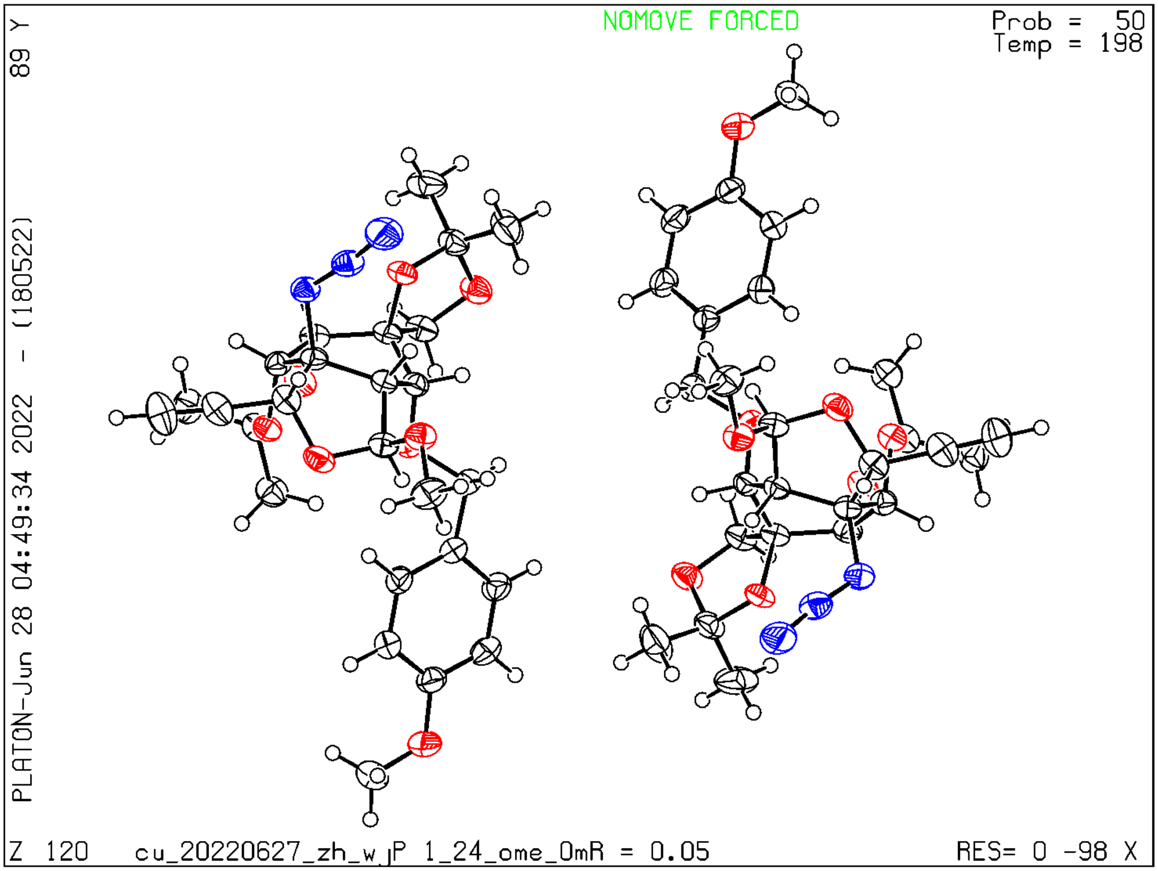

Supplement: Supplementary file 4 — Source Data [file 41467_2024_45037_MOESM4_ESM.zip › Surce Data 20231213/Surce Data New/Crystal Structure Source Data/CCDC-2184305/checkcif.pdf]
