# Supplementary material for: Total syntheses of Tetrodotoxin and 9-epiTetrodotoxin
Source: Nat Commun. 2024 Jan 23;15:679. doi: 10.1038/s41467-024-45037-0 (PMC10806222; doi:10.1038/s41467-024-45037-0)
Supplement: Supplementary file 4 — Source Data [file 41467_2024_45037_MOESM4_ESM.zip › Surce Data 20231213/Surce Data New/Crystal Structure Source Data/CCDC-2184305/cu_20220627_Zh_Wj_06_24_OMe_0m_tables.html]

cu\_20220627\_Zh\_Wj\_06\_24\_OMe\_0m


# cu\_20220627\_Zh\_Wj\_06\_24\_OMe\_0m

Table 1 Crystal data and structure refinement for cu\_20220627\_Zh\_Wj\_06\_24\_OMe\_0m.

| Identification code | cu\_20220627\_Zh\_Wj\_06\_24\_OMe\_0m |
| Empirical formula | C26H33N3O8 |
| Formula weight | 515.55 |
| Temperature/K | 198.00 |
| Crystal system | triclinic |
| Space group | P1 |
| a/Å | 8.3171(2) |
| b/Å | 10.4531(3) |
| c/Å | 16.3414(5) |
| α/° | 84.815(2) |
| β/° | 83.036(2) |
| γ/° | 67.7400(10) |
| Volume/Å3 | 1303.61(6) |
| Z | 2 |
| ρcalcg/cm3 | 1.313 |
| μ/mm‑1 | 0.815 |
| F(000) | 548.0 |
| Crystal size/mm3 | 0.15 × 0.12 × 0.1 |
| Radiation | CuKα (λ = 1.54178) |
| 2Θ range for data collection/° | 5.454 to 136.766 |
| Index ranges | -10 ≤ h ≤ 10, -12 ≤ k ≤ 12, -19 ≤ l ≤ 19 |
| Reflections collected | 28420 |
| Independent reflections | 9268 [Rint = 0.0498, Rsigma = 0.0490] |
| Data/restraints/parameters | 9268/3/679 |
| Goodness-of-fit on F2 | 1.034 |
| Final R indexes [I>=2σ (I)] | R1 = 0.0469, wR2 = 0.1185 |
| Final R indexes [all data] | R1 = 0.0518, wR2 = 0.1236 |
| Largest diff. peak/hole / e Å-3 | 0.41/-0.26 || Flack parameter | 0.01(8) |

Table 2 Fractional Atomic Coordinates (×104) and Equivalent Isotropic Displacement Parameters (Å2×103) for cu\_20220627\_Zh\_Wj\_06\_24\_OMe\_0m. Ueq is defined as 1/3 of the trace of the orthogonalised UIJ tensor.

| Atom | *x* | *y* | *z* | U(eq) |
| --- | --- | --- | --- | --- |
| O(1) | 4211(3) | 7808(3) | -3749.6(15) | 32.5(5) |
| O(2) | 2507(3) | 6545(3) | -3634.1(17) | 38.0(6) |
| O(3) | 5989(3) | 3485(2) | -2931.0(16) | 34.0(5) |
| O(4) | 3946(3) | 3172(3) | -1960.3(17) | 41.5(6) |
| O(5) | 3852(3) | 6803(2) | -1984.3(14) | 31.4(5) |
| O(6) | 7207(4) | 7952(3) | -2951.9(17) | 39.6(6) |
| O(7) | 8744(3) | 6483(3) | -1914.2(16) | 37.2(6) |
| O(8) | -700(4) | 12492(3) | -391.3(18) | 55.7(8) |
| N(1) | 8225(4) | 4811(3) | -4034(2) | 40.4(7) |
| N(2) | 9240(4) | 3845(4) | -3656(2) | 41.9(7) |
| N(3) | 10228(5) | 2878(4) | -3363(3) | 61.7(11) |
| C(1) | 2529(4) | 7882(4) | -3903(2) | 33.1(7) |
| C(2) | 2265(5) | 8153(5) | -4816(2) | 44.2(9) |
| C(3) | 1186(5) | 8965(4) | -3378(2) | 41.5(8) |
| C(4) | 5360(4) | 6458(4) | -3958(2) | 31.2(7) |
| C(5) | 4263(5) | 5561(4) | -3665(2) | 33.5(7) |
| C(6) | 4607(4) | 4816(4) | -2816(2) | 32.1(7) |
| C(7) | 3091(5) | 4432(4) | -2420(3) | 38.4(8) |
| C(8) | 5485(5) | 2433(4) | -2469(3) | 40.2(8) |
| C(9) | 5108(7) | 1574(5) | -3059(3) | 61.3(13) |
| C(10) | 6886(7) | 1605(5) | -1920(3) | 64.6(14) |
| C(11) | 7056(4) | 6038(4) | -3559(2) | 31.6(7) |
| C(12) | 6880(4) | 5800(4) | -2613(2) | 29.7(7) |
| C(13) | 5226(4) | 5561(3) | -2225(2) | 30.1(7) |
| C(14) | 7900(5) | 7148(4) | -3666(2) | 38.2(8) |
| C(15) | 7556(5) | 8074(4) | -4415(3) | 43.2(9) |
| C(16) | 7360(6) | 8808(5) | -5008(3) | 53.7(11) |
| C(17) | 7167(4) | 7011(4) | -2273(2) | 32.7(7) |
| C(18) | 9066(6) | 7486(5) | -1495(3) | 45.3(9) |
| C(19) | 3583(5) | 6970(4) | -1113(2) | 35.3(8) |
| C(20) | 2429(5) | 8446(4) | -948(2) | 32.2(7) |
| C(21) | 1212(5) | 8755(4) | -259(2) | 39.4(8) |
| C(22) | 192(6) | 10099(5) | -90(2) | 43.3(9) |
| C(23) | 372(5) | 11174(4) | -607(2) | 40.2(8) |
| C(24) | 1579(5) | 10893(4) | -1296(2) | 39.6(8) |
| C(25) | 2587(5) | 9525(4) | -1460(2) | 35.6(8) |
| C(26) | -473(9) | 13606(5) | -888(3) | 71.1(16) |
| O(9) | 3120(3) | 6587(2) | 2917.3(16) | 33.3(5) |
| O(10) | 1207(3) | 6902(3) | 1955.4(17) | 42.1(6) |
| O(11) | 5120(3) | 2300(2) | 3780.9(15) | 32.3(5) |
| O(12) | 2276(3) | 3496(3) | 3619.3(17) | 37.9(6) |
| O(13) | 8603(3) | 2215(3) | 3006.7(17) | 39.9(6) |
| O(14) | 9196(3) | 3595(3) | 1902.2(17) | 37.7(6) |
| O(15) | 4618(3) | 3254(2) | 1989.1(14) | 31.3(5) |
| O(16) | 6643(5) | -2499(3) | 366.0(19) | 56.8(8) |
| N(4) | 5950(4) | 5416(3) | 3996.7(19) | 35.6(7) |
| N(5) | 6355(4) | 6329(3) | 3618(2) | 39.4(7) |
| N(6) | 6651(7) | 7249(5) | 3321(3) | 63.3(11) |
| C(27) | 1829(5) | 7637(4) | 2450(2) | 37.8(8) |
| C(28) | 391(6) | 8523(5) | 3057(3) | 55.8(11) |
| C(29) | 2725(7) | 8437(5) | 1885(3) | 56.5(12) |
| C(30) | 1360(5) | 5627(4) | 2416(3) | 38.1(8) |
| C(31) | 3055(5) | 5263(4) | 2803(2) | 31.6(7) |
| C(32) | 3023(4) | 4518(4) | 3656(2) | 33.0(7) |
| C(33) | 4830(4) | 3678(4) | 3965(2) | 31.1(7) |
| C(34) | 3450(4) | 2196(4) | 3924(2) | 32.6(7) |
| C(35) | 2967(6) | 1983(5) | 4842(3) | 45.6(9) |
| C(36) | 3433(5) | 1062(4) | 3422(3) | 42.5(9) |
| C(37) | 6286(4) | 4133(4) | 3557(2) | 29.7(7) |
| C(38) | 8151(5) | 3096(4) | 3685(2) | 36.4(8) |
| C(39) | 8333(5) | 2280(4) | 4468(3) | 42.3(9) |
| C(40) | 8471(6) | 1677(6) | 5114(3) | 57.4(12) |
| C(41) | 7977(4) | 3074(4) | 2304(2) | 33.1(7) |
| C(42) | 10695(5) | 2564(5) | 1520(3) | 49.7(10) |
| C(43) | 6357(4) | 4308(4) | 2604(2) | 30.3(7) |
| C(44) | 4659(4) | 4519(4) | 2219(2) | 30.9(7) |
| C(45) | 4970(5) | 3064(4) | 1119(2) | 36.5(8) |
| C(46) | 5370(4) | 1582(4) | 961(2) | 31.9(7) |
| C(47) | 4808(5) | 1244(4) | 266(2) | 40.6(8) |
| C(48) | 5253(6) | -123(5) | 86(3) | 45.2(9) |
| C(49) | 6269(6) | -1178(4) | 593(2) | 40.3(8) |
| C(50) | 6829(5) | -867(4) | 1290(2) | 39.5(8) |
| C(51) | 6364(5) | 509(4) | 1467(2) | 35.3(8) |
| C(52) | 7786(10) | -3587(5) | 840(3) | 75.0(17) |

Table 3 Anisotropic Displacement Parameters (Å2×103) for cu\_20220627\_Zh\_Wj\_06\_24\_OMe\_0m. The Anisotropic displacement factor exponent takes the form: -2π2[h2a\*2U11+2hka\*b\*U12+…].

| Atom | U11 | U22 | U33 | U23 | U13 | U12 |
| --- | --- | --- | --- | --- | --- | --- |
| O(1) | 29.1(12) | 31.3(13) | 38.5(13) | 0.2(10) | -5.9(9) | -12.2(10) |
| O(2) | 29.0(12) | 34.1(14) | 52.6(15) | 6.3(11) | -11.1(11) | -13.3(11) |
| O(3) | 33.4(12) | 20.2(12) | 45.9(14) | -0.1(10) | 0.0(10) | -8.6(10) |
| O(4) | 42.9(14) | 30.6(14) | 47.5(15) | 1.7(11) | 6.2(11) | -13.5(11) |
| O(5) | 33.1(12) | 30.0(13) | 27.4(11) | -4.0(9) | -1.9(9) | -7.4(10) |
| O(6) | 47.4(15) | 29.5(14) | 45.8(14) | 2.2(11) | -13.5(11) | -16.8(11) |
| O(7) | 36.5(13) | 35.6(14) | 42.8(13) | -5.8(11) | -12.4(11) | -13.5(11) |
| O(8) | 68(2) | 38.2(17) | 44.5(16) | -9.2(13) | -1.6(14) | -1.4(15) |
| N(1) | 39.6(17) | 33.9(18) | 40.4(17) | -4.5(13) | -1.1(13) | -5.6(14) |
| N(2) | 35.7(16) | 41(2) | 44.2(18) | -10.0(15) | -0.6(14) | -7.6(15) |
| N(3) | 57(2) | 51(3) | 55(2) | -8.8(19) | -7.0(19) | 7(2) |
| C(1) | 29.2(17) | 30.0(19) | 41.4(19) | 1.9(14) | -7.8(14) | -12.1(14) |
| C(2) | 40(2) | 51(2) | 40(2) | 1.8(17) | -11.9(16) | -13.0(18) |
| C(3) | 34.2(18) | 42(2) | 44(2) | -1.1(16) | -6.7(15) | -8.4(16) |
| C(4) | 31.1(17) | 28.2(18) | 34.7(17) | -1.8(13) | -4.1(13) | -11.2(14) |
| C(5) | 34.4(17) | 28.2(19) | 40.2(19) | -2.8(14) | -8.0(14) | -12.5(15) |
| C(6) | 29.2(16) | 23.4(18) | 41.1(18) | -4.7(14) | -1.5(13) | -6.8(13) |
| C(7) | 35.4(18) | 28.3(19) | 51(2) | -1.1(15) | -2.7(16) | -11.8(15) |
| C(8) | 42(2) | 28(2) | 49(2) | 2.3(16) | 4.2(16) | -13.6(16) |
| C(9) | 70(3) | 41(3) | 79(3) | -21(2) | 18(3) | -32(2) |
| C(10) | 58(3) | 48(3) | 66(3) | 21(2) | 1(2) | -3(2) |
| C(11) | 27.9(16) | 30.4(19) | 35.6(18) | -4.4(14) | -2.2(13) | -9.5(14) |
| C(12) | 29.2(16) | 24.5(17) | 34.7(17) | -2.3(13) | -6.3(13) | -7.8(13) |
| C(13) | 30.0(16) | 24.4(18) | 33.2(17) | -0.2(13) | -5.1(13) | -6.6(13) |
| C(14) | 33.5(18) | 42(2) | 41(2) | 1.5(16) | -6.8(15) | -15.8(16) |
| C(15) | 37.8(19) | 43(2) | 54(2) | 6.2(18) | -7.7(17) | -22.4(17) |
| C(16) | 50(2) | 60(3) | 53(3) | 14(2) | -8.9(19) | -26(2) |
| C(17) | 27.6(16) | 28.2(18) | 44(2) | -6.9(14) | -2.1(14) | -10.9(14) |
| C(18) | 50(2) | 49(2) | 47(2) | -9.6(17) | -11.3(17) | -25.5(19) |
| C(19) | 41.2(19) | 34(2) | 31.3(18) | -2.0(14) | -2.8(14) | -14.7(16) |
| C(20) | 35.7(18) | 33(2) | 30.6(17) | -3.2(13) | -3.8(13) | -15.7(15) |
| C(21) | 48(2) | 41(2) | 32.4(18) | -3.2(15) | 3.0(15) | -22.8(18) |
| C(22) | 52(2) | 47(2) | 29.9(18) | -10.3(16) | 6.0(15) | -17.3(19) |
| C(23) | 45(2) | 36(2) | 34.3(18) | -9.5(15) | -7.4(15) | -6.8(17) |
| C(24) | 46(2) | 35(2) | 35.1(19) | 2.8(15) | -5.6(15) | -12.6(17) |
| C(25) | 36.2(18) | 37(2) | 32.5(17) | -0.1(14) | -2.0(14) | -13.8(16) |
| C(26) | 106(4) | 31(3) | 55(3) | 0(2) | -8(3) | -2(3) |
| O(9) | 33.4(12) | 21.5(12) | 44.7(14) | -2.4(10) | -11.2(10) | -7.5(10) |
| O(10) | 43.7(15) | 33.6(15) | 49.0(15) | 0.5(11) | -19.7(12) | -10.3(12) |
| O(11) | 30.2(12) | 27.9(13) | 39.5(13) | -0.9(10) | -2.9(10) | -11.8(10) |
| O(12) | 30.0(12) | 35.8(15) | 51.3(15) | 2.6(11) | -7.8(10) | -15.9(11) |
| O(13) | 36.0(13) | 26.7(13) | 49.6(15) | 0.6(11) | -0.3(11) | -4.9(11) |
| O(14) | 31.6(12) | 36.0(15) | 43.8(14) | -6.5(11) | 3.4(10) | -11.7(11) |
| O(15) | 36.1(12) | 30.2(13) | 29.9(12) | -6.4(9) | -3.3(9) | -14.0(10) |
| O(16) | 90(2) | 36.4(17) | 47.0(16) | -11.1(13) | -0.6(15) | -26.3(16) |
| N(4) | 40.4(16) | 31.1(17) | 38.4(16) | -4.9(13) | -6.8(13) | -15.1(13) |
| N(5) | 42.4(17) | 36.9(19) | 43.6(17) | -9.5(14) | -2.0(13) | -18.9(15) |
| N(6) | 92(3) | 49(2) | 64(2) | -8.4(19) | 3(2) | -44(2) |
| C(27) | 37.8(19) | 28(2) | 46(2) | 1.4(15) | -14.4(16) | -8.2(16) |
| C(28) | 46(2) | 39(2) | 72(3) | -14(2) | -15(2) | 0.0(19) |
| C(29) | 75(3) | 43(3) | 58(3) | 12(2) | -22(2) | -29(2) |
| C(30) | 33.0(18) | 29.3(19) | 53(2) | -4.5(15) | -10.8(16) | -9.6(15) |
| C(31) | 32.3(17) | 22.0(17) | 41.8(19) | -4.4(14) | -6.9(14) | -10.1(14) |
| C(32) | 29.5(17) | 31.5(19) | 39.3(18) | -5.8(14) | -0.2(14) | -12.7(14) |
| C(33) | 31.5(17) | 29.1(18) | 34.2(17) | -3.5(13) | -2.2(13) | -12.9(14) |
| C(34) | 29.3(16) | 30.6(19) | 39.0(19) | 2.1(14) | -3.5(13) | -13.2(14) |
| C(35) | 47(2) | 53(3) | 43(2) | -1.2(18) | 1.0(17) | -27(2) |
| C(36) | 45(2) | 39(2) | 49(2) | -1.5(17) | -7.0(17) | -20.9(17) |
| C(37) | 30.8(17) | 27.0(18) | 33.3(17) | -3.8(13) | -5.3(13) | -11.9(14) |
| C(38) | 31.4(18) | 38(2) | 42(2) | 2.4(15) | -6.7(14) | -15.0(16) |
| C(39) | 31.2(18) | 42(2) | 53(2) | 8.5(18) | -10.7(16) | -13.6(16) |
| C(40) | 49(2) | 63(3) | 61(3) | 21(2) | -19(2) | -23(2) |
| C(41) | 28.0(16) | 28.6(18) | 43.0(19) | -7.5(14) | -4.4(13) | -9.3(14) |
| C(42) | 34.5(19) | 56(3) | 51(2) | -14(2) | 6.2(17) | -9.0(18) |
| C(43) | 31.4(17) | 25.6(18) | 34.3(17) | -2.7(13) | -2.9(13) | -10.9(14) |
| C(44) | 32.7(17) | 25.1(18) | 34.4(17) | -1.3(13) | -3.6(13) | -10.2(14) |
| C(45) | 44(2) | 32.4(19) | 29.5(17) | -5.3(14) | -5.0(14) | -8.9(15) |
| C(46) | 31.9(17) | 33.7(19) | 30.1(17) | -5.4(13) | -1.4(13) | -12.0(15) |
| C(47) | 43(2) | 41(2) | 35.6(19) | -5.4(15) | -12.7(15) | -10.1(17) |
| C(48) | 57(2) | 46(2) | 35.7(19) | -12.0(16) | -7.5(17) | -19(2) |
| C(49) | 53(2) | 33(2) | 37.8(19) | -8.1(15) | 3.5(16) | -19.8(18) |
| C(50) | 48(2) | 35(2) | 34.4(19) | 0.8(15) | -3.6(15) | -14.9(17) |
| C(51) | 40.6(19) | 37(2) | 30.5(17) | -3.2(14) | -4.9(14) | -16.2(16) |
| C(52) | 131(5) | 31(3) | 52(3) | 1(2) | -5(3) | -19(3) |

Table 4 Bond Lengths for cu\_20220627\_Zh\_Wj\_06\_24\_OMe\_0m.

| Atom | Atom | Length/Å |  | Atom | Atom | Length/Å |
| --- | --- | --- | --- | --- | --- | --- |
| O(1) | C(1) | 1.423(4) |  | O(9) | C(27) | 1.447(4) |
| O(1) | C(4) | 1.417(4) |  | O(9) | C(31) | 1.435(4) |
| O(2) | C(1) | 1.433(4) |  | O(10) | C(27) | 1.424(5) |
| O(2) | C(5) | 1.431(4) |  | O(10) | C(30) | 1.439(5) |
| O(3) | C(6) | 1.440(4) |  | O(11) | C(33) | 1.422(4) |
| O(3) | C(8) | 1.441(4) |  | O(11) | C(34) | 1.423(4) |
| O(4) | C(7) | 1.429(5) |  | O(12) | C(32) | 1.433(4) |
| O(4) | C(8) | 1.427(5) |  | O(12) | C(34) | 1.427(4) |
| O(5) | C(13) | 1.416(4) |  | O(13) | C(38) | 1.428(5) |
| O(5) | C(19) | 1.431(4) |  | O(13) | C(41) | 1.414(5) |
| O(6) | C(14) | 1.431(5) |  | O(14) | C(41) | 1.396(4) |
| O(6) | C(17) | 1.421(5) |  | O(14) | C(42) | 1.422(5) |
| O(7) | C(17) | 1.396(4) |  | O(15) | C(44) | 1.421(4) |
| O(7) | C(18) | 1.424(4) |  | O(15) | C(45) | 1.433(4) |
| O(8) | C(23) | 1.377(5) |  | O(16) | C(49) | 1.372(5) |
| O(8) | C(26) | 1.417(6) |  | O(16) | C(52) | 1.414(7) |
| N(1) | N(2) | 1.218(5) |  | N(4) | N(5) | 1.223(5) |
| N(1) | C(11) | 1.498(5) |  | N(4) | C(37) | 1.495(4) |
| N(2) | N(3) | 1.142(5) |  | N(5) | N(6) | 1.134(5) |
| C(1) | C(2) | 1.519(5) |  | C(27) | C(28) | 1.520(6) |
| C(1) | C(3) | 1.500(5) |  | C(27) | C(29) | 1.508(6) |
| C(4) | C(5) | 1.550(5) |  | C(30) | C(31) | 1.516(5) |
| C(4) | C(11) | 1.520(5) |  | C(31) | C(32) | 1.537(5) |
| C(5) | C(6) | 1.530(5) |  | C(31) | C(44) | 1.528(5) |
| C(6) | C(7) | 1.520(5) |  | C(32) | C(33) | 1.545(5) |
| C(6) | C(13) | 1.538(5) |  | C(33) | C(37) | 1.523(5) |
| C(8) | C(9) | 1.509(6) |  | C(34) | C(35) | 1.524(5) |
| C(8) | C(10) | 1.504(7) |  | C(34) | C(36) | 1.506(5) |
| C(11) | C(12) | 1.542(5) |  | C(37) | C(38) | 1.546(5) |
| C(11) | C(14) | 1.555(5) |  | C(37) | C(43) | 1.548(5) |
| C(12) | C(13) | 1.542(5) |  | C(38) | C(39) | 1.463(5) |
| C(12) | C(17) | 1.535(5) |  | C(39) | C(40) | 1.174(6) |
| C(14) | C(15) | 1.476(5) |  | C(41) | C(43) | 1.535(5) |
| C(15) | C(16) | 1.167(6) |  | C(43) | C(44) | 1.546(5) |
| C(19) | C(20) | 1.505(5) |  | C(45) | C(46) | 1.497(5) |
| C(20) | C(21) | 1.392(5) |  | C(46) | C(47) | 1.399(5) |
| C(20) | C(25) | 1.380(5) |  | C(46) | C(51) | 1.386(5) |
| C(21) | C(22) | 1.371(6) |  | C(47) | C(48) | 1.383(6) |
| C(22) | C(23) | 1.387(6) |  | C(48) | C(49) | 1.384(6) |
| C(23) | C(24) | 1.387(6) |  | C(49) | C(50) | 1.389(6) |
| C(24) | C(25) | 1.388(6) |  | C(50) | C(51) | 1.388(5) |

Table 5 Bond Angles for cu\_20220627\_Zh\_Wj\_06\_24\_OMe\_0m.

| Atom | Atom | Atom | Angle/˚ |  | Atom | Atom | Atom | Angle/˚ |
| --- | --- | --- | --- | --- | --- | --- | --- | --- |
| C(4) | O(1) | C(1) | 105.1(3) |  | C(31) | O(9) | C(27) | 108.9(3) |
| C(5) | O(2) | C(1) | 109.0(3) |  | C(27) | O(10) | C(30) | 105.8(3) |
| C(6) | O(3) | C(8) | 109.3(3) |  | C(33) | O(11) | C(34) | 105.2(3) |
| C(8) | O(4) | C(7) | 105.6(3) |  | C(34) | O(12) | C(32) | 108.6(3) |
| C(13) | O(5) | C(19) | 114.0(3) |  | C(41) | O(13) | C(38) | 106.8(3) |
| C(17) | O(6) | C(14) | 106.8(3) |  | C(41) | O(14) | C(42) | 113.4(3) |
| C(17) | O(7) | C(18) | 113.4(3) |  | C(44) | O(15) | C(45) | 113.6(3) |
| C(23) | O(8) | C(26) | 117.0(4) |  | C(49) | O(16) | C(52) | 116.9(4) |
| N(2) | N(1) | C(11) | 117.7(3) |  | N(5) | N(4) | C(37) | 117.6(3) |
| N(3) | N(2) | N(1) | 174.1(4) |  | N(6) | N(5) | N(4) | 174.0(4) |
| O(1) | C(1) | O(2) | 104.4(3) |  | O(9) | C(27) | C(28) | 108.2(3) |
| O(1) | C(1) | C(2) | 111.5(3) |  | O(9) | C(27) | C(29) | 108.6(3) |
| O(1) | C(1) | C(3) | 108.3(3) |  | O(10) | C(27) | O(9) | 105.6(3) |
| O(2) | C(1) | C(2) | 109.9(3) |  | O(10) | C(27) | C(28) | 112.0(3) |
| O(2) | C(1) | C(3) | 109.7(3) |  | O(10) | C(27) | C(29) | 108.3(3) |
| C(3) | C(1) | C(2) | 112.7(3) |  | C(29) | C(27) | C(28) | 113.9(4) |
| O(1) | C(4) | C(5) | 103.1(3) |  | O(10) | C(30) | C(31) | 102.4(3) |
| O(1) | C(4) | C(11) | 111.2(3) |  | O(9) | C(31) | C(30) | 103.5(3) |
| C(11) | C(4) | C(5) | 114.5(3) |  | O(9) | C(31) | C(32) | 108.0(3) |
| O(2) | C(5) | C(4) | 103.1(3) |  | O(9) | C(31) | C(44) | 106.0(3) |
| O(2) | C(5) | C(6) | 109.4(3) |  | C(30) | C(31) | C(32) | 112.0(3) |
| C(6) | C(5) | C(4) | 115.3(3) |  | C(30) | C(31) | C(44) | 112.7(3) |
| O(3) | C(6) | C(5) | 108.2(3) |  | C(44) | C(31) | C(32) | 113.8(3) |
| O(3) | C(6) | C(7) | 102.6(3) |  | O(12) | C(32) | C(31) | 109.2(3) |
| O(3) | C(6) | C(13) | 106.2(3) |  | O(12) | C(32) | C(33) | 103.6(3) |
| C(5) | C(6) | C(13) | 114.1(3) |  | C(31) | C(32) | C(33) | 115.4(3) |
| C(7) | C(6) | C(5) | 112.6(3) |  | O(11) | C(33) | C(32) | 103.0(3) |
| C(7) | C(6) | C(13) | 112.2(3) |  | O(11) | C(33) | C(37) | 111.7(3) |
| O(4) | C(7) | C(6) | 102.7(3) |  | C(37) | C(33) | C(32) | 114.1(3) |
| O(3) | C(8) | C(9) | 109.3(3) |  | O(11) | C(34) | O(12) | 104.5(3) |
| O(3) | C(8) | C(10) | 109.0(3) |  | O(11) | C(34) | C(35) | 111.1(3) |
| O(4) | C(8) | O(3) | 105.1(3) |  | O(11) | C(34) | C(36) | 109.1(3) |
| O(4) | C(8) | C(9) | 110.9(3) |  | O(12) | C(34) | C(35) | 109.9(3) |
| O(4) | C(8) | C(10) | 108.4(4) |  | O(12) | C(34) | C(36) | 109.2(3) |
| C(10) | C(8) | C(9) | 113.7(4) |  | C(36) | C(34) | C(35) | 112.7(3) |
| N(1) | C(11) | C(4) | 102.2(3) |  | N(4) | C(37) | C(33) | 101.7(3) |
| N(1) | C(11) | C(12) | 115.9(3) |  | N(4) | C(37) | C(38) | 109.0(3) |
| N(1) | C(11) | C(14) | 108.3(3) |  | N(4) | C(37) | C(43) | 115.6(3) |
| C(4) | C(11) | C(12) | 114.7(3) |  | C(33) | C(37) | C(38) | 114.7(3) |
| C(4) | C(11) | C(14) | 114.3(3) |  | C(33) | C(37) | C(43) | 114.6(3) |
| C(12) | C(11) | C(14) | 101.8(3) |  | C(38) | C(37) | C(43) | 101.7(3) |
| C(13) | C(12) | C(11) | 116.7(3) |  | O(13) | C(38) | C(37) | 104.3(3) |
| C(17) | C(12) | C(11) | 105.1(3) |  | O(13) | C(38) | C(39) | 110.6(3) |
| C(17) | C(12) | C(13) | 114.7(3) |  | C(39) | C(38) | C(37) | 115.4(3) |
| O(5) | C(13) | C(6) | 110.2(3) |  | C(40) | C(39) | C(38) | 177.0(5) |
| O(5) | C(13) | C(12) | 112.6(3) |  | O(13) | C(41) | C(43) | 107.4(3) |
| C(6) | C(13) | C(12) | 110.9(3) |  | O(14) | C(41) | O(13) | 112.1(3) |
| O(6) | C(14) | C(11) | 104.5(3) |  | O(14) | C(41) | C(43) | 107.7(3) |
| O(6) | C(14) | C(15) | 109.3(3) |  | C(41) | C(43) | C(37) | 104.3(3) |
| C(15) | C(14) | C(11) | 116.5(3) |  | C(41) | C(43) | C(44) | 115.8(3) |
| C(16) | C(15) | C(14) | 176.9(4) |  | C(44) | C(43) | C(37) | 116.3(3) |
| O(6) | C(17) | C(12) | 106.9(3) |  | O(15) | C(44) | C(31) | 110.7(3) |
| O(7) | C(17) | O(6) | 111.7(3) |  | O(15) | C(44) | C(43) | 112.3(3) |
| O(7) | C(17) | C(12) | 108.1(3) |  | C(31) | C(44) | C(43) | 110.9(3) |
| O(5) | C(19) | C(20) | 109.0(3) |  | O(15) | C(45) | C(46) | 109.4(3) |
| C(21) | C(20) | C(19) | 120.5(3) |  | C(47) | C(46) | C(45) | 120.1(3) |
| C(25) | C(20) | C(19) | 120.9(3) |  | C(51) | C(46) | C(45) | 121.7(3) |
| C(25) | C(20) | C(21) | 118.5(4) |  | C(51) | C(46) | C(47) | 118.1(3) |
| C(22) | C(21) | C(20) | 121.0(4) |  | C(48) | C(47) | C(46) | 120.8(4) |
| C(21) | C(22) | C(23) | 120.0(3) |  | C(47) | C(48) | C(49) | 120.2(3) |
| O(8) | C(23) | C(22) | 116.2(4) |  | O(16) | C(49) | C(48) | 116.1(4) |
| O(8) | C(23) | C(24) | 123.7(4) |  | O(16) | C(49) | C(50) | 123.9(4) |
| C(24) | C(23) | C(22) | 120.1(4) |  | C(48) | C(49) | C(50) | 120.1(4) |
| C(23) | C(24) | C(25) | 119.0(4) |  | C(51) | C(50) | C(49) | 119.2(4) |
| C(20) | C(25) | C(24) | 121.4(3) |  | C(46) | C(51) | C(50) | 121.7(3) |

Table 6 Torsion Angles for cu\_20220627\_Zh\_Wj\_06\_24\_OMe\_0m.

| A | B | C | D | Angle/˚ |  | A | B | C | D | Angle/˚ |
| --- | --- | --- | --- | --- | --- | --- | --- | --- | --- | --- |
| O(1) | C(4) | C(5) | O(2) | 21.6(3) |  | O(9) | C(31) | C(32) | O(12) | 157.1(3) |
| O(1) | C(4) | C(5) | C(6) | -97.5(3) |  | O(9) | C(31) | C(32) | C(33) | -86.7(4) |
| O(1) | C(4) | C(11) | N(1) | -164.7(3) |  | O(9) | C(31) | C(44) | O(15) | -173.3(3) |
| O(1) | C(4) | C(11) | C(12) | 69.1(4) |  | O(9) | C(31) | C(44) | C(43) | 61.4(3) |
| O(1) | C(4) | C(11) | C(14) | -48.0(4) |  | O(10) | C(30) | C(31) | O(9) | 31.0(4) |
| O(2) | C(5) | C(6) | O(3) | 154.8(3) |  | O(10) | C(30) | C(31) | C(32) | 147.0(3) |
| O(2) | C(5) | C(6) | C(7) | 42.2(4) |  | O(10) | C(30) | C(31) | C(44) | -83.1(3) |
| O(2) | C(5) | C(6) | C(13) | -87.2(3) |  | O(11) | C(33) | C(37) | N(4) | -166.4(3) |
| O(3) | C(6) | C(7) | O(4) | 31.3(4) |  | O(11) | C(33) | C(37) | C(38) | -49.0(4) |
| O(3) | C(6) | C(13) | O(5) | -171.9(3) |  | O(11) | C(33) | C(37) | C(43) | 68.2(4) |
| O(3) | C(6) | C(13) | C(12) | 62.7(3) |  | O(12) | C(32) | C(33) | O(11) | 20.0(3) |
| O(5) | C(19) | C(20) | C(21) | 144.3(3) |  | O(12) | C(32) | C(33) | C(37) | 141.2(3) |
| O(5) | C(19) | C(20) | C(25) | -37.7(4) |  | O(13) | C(41) | C(43) | C(37) | 6.6(3) |
| O(8) | C(23) | C(24) | C(25) | -179.5(4) |  | O(13) | C(41) | C(43) | C(44) | -122.5(3) |
| N(1) | C(11) | C(12) | C(13) | -100.3(4) |  | O(14) | C(41) | C(43) | C(37) | -114.2(3) |
| N(1) | C(11) | C(12) | C(17) | 131.4(3) |  | O(14) | C(41) | C(43) | C(44) | 116.6(3) |
| N(1) | C(11) | C(14) | O(6) | -155.1(3) |  | O(15) | C(45) | C(46) | C(47) | 143.5(3) |
| N(1) | C(11) | C(14) | C(15) | 84.1(4) |  | O(15) | C(45) | C(46) | C(51) | -39.5(5) |
| N(2) | N(1) | C(11) | C(4) | -142.1(3) |  | O(16) | C(49) | C(50) | C(51) | -179.5(4) |
| N(2) | N(1) | C(11) | C(12) | -16.6(5) |  | N(4) | C(37) | C(38) | O(13) | -157.0(3) |
| N(2) | N(1) | C(11) | C(14) | 96.9(4) |  | N(4) | C(37) | C(38) | C(39) | 81.5(4) |
| C(1) | O(1) | C(4) | C(5) | -37.1(3) |  | N(4) | C(37) | C(43) | C(41) | 134.4(3) |
| C(1) | O(1) | C(4) | C(11) | -160.3(3) |  | N(4) | C(37) | C(43) | C(44) | -96.8(3) |
| C(1) | O(2) | C(5) | C(4) | 1.6(4) |  | N(5) | N(4) | C(37) | C(33) | -149.8(3) |
| C(1) | O(2) | C(5) | C(6) | 124.8(3) |  | N(5) | N(4) | C(37) | C(38) | 88.7(4) |
| C(4) | O(1) | C(1) | O(2) | 38.8(3) |  | N(5) | N(4) | C(37) | C(43) | -25.0(4) |
| C(4) | O(1) | C(1) | C(2) | -79.8(4) |  | C(27) | O(9) | C(31) | C(30) | -13.5(4) |
| C(4) | O(1) | C(1) | C(3) | 155.7(3) |  | C(27) | O(9) | C(31) | C(32) | -132.4(3) |
| C(4) | C(5) | C(6) | O(3) | -89.5(4) |  | C(27) | O(9) | C(31) | C(44) | 105.3(3) |
| C(4) | C(5) | C(6) | C(7) | 157.8(3) |  | C(27) | O(10) | C(30) | C(31) | -37.7(4) |
| C(4) | C(5) | C(6) | C(13) | 28.4(4) |  | C(30) | O(10) | C(27) | O(9) | 29.9(4) |
| C(4) | C(11) | C(12) | C(13) | 18.5(4) |  | C(30) | O(10) | C(27) | C(28) | -87.6(4) |
| C(4) | C(11) | C(12) | C(17) | -109.8(3) |  | C(30) | O(10) | C(27) | C(29) | 146.0(3) |
| C(4) | C(11) | C(14) | O(6) | 91.7(3) |  | C(30) | C(31) | C(32) | O(12) | 43.8(4) |
| C(4) | C(11) | C(14) | C(15) | -29.0(5) |  | C(30) | C(31) | C(32) | C(33) | 160.0(3) |
| C(5) | O(2) | C(1) | O(1) | -24.3(4) |  | C(30) | C(31) | C(44) | O(15) | -60.8(4) |
| C(5) | O(2) | C(1) | C(2) | 95.4(3) |  | C(30) | C(31) | C(44) | C(43) | 174.0(3) |
| C(5) | O(2) | C(1) | C(3) | -140.2(3) |  | C(31) | O(9) | C(27) | O(10) | -9.3(4) |
| C(5) | C(4) | C(11) | N(1) | 78.9(4) |  | C(31) | O(9) | C(27) | C(28) | 110.7(3) |
| C(5) | C(4) | C(11) | C(12) | -47.3(4) |  | C(31) | O(9) | C(27) | C(29) | -125.3(3) |
| C(5) | C(4) | C(11) | C(14) | -164.3(3) |  | C(31) | C(32) | C(33) | O(11) | -99.3(3) |
| C(5) | C(6) | C(7) | O(4) | 147.4(3) |  | C(31) | C(32) | C(33) | C(37) | 21.9(4) |
| C(5) | C(6) | C(13) | O(5) | 69.1(4) |  | C(32) | O(12) | C(34) | O(11) | -25.8(4) |
| C(5) | C(6) | C(13) | C(12) | -56.3(4) |  | C(32) | O(12) | C(34) | C(35) | 93.5(3) |
| C(6) | O(3) | C(8) | O(4) | -10.5(4) |  | C(32) | O(12) | C(34) | C(36) | -142.4(3) |
| C(6) | O(3) | C(8) | C(9) | 108.6(4) |  | C(32) | C(31) | C(44) | O(15) | 68.1(3) |
| C(6) | O(3) | C(8) | C(10) | -126.5(4) |  | C(32) | C(31) | C(44) | C(43) | -57.2(4) |
| C(7) | O(4) | C(8) | O(3) | 31.4(4) |  | C(32) | C(33) | C(37) | N(4) | 77.4(3) |
| C(7) | O(4) | C(8) | C(9) | -86.7(4) |  | C(32) | C(33) | C(37) | C(38) | -165.2(3) |
| C(7) | O(4) | C(8) | C(10) | 147.8(4) |  | C(32) | C(33) | C(37) | C(43) | -48.1(4) |
| C(7) | C(6) | C(13) | O(5) | -60.5(4) |  | C(33) | O(11) | C(34) | O(12) | 39.2(3) |
| C(7) | C(6) | C(13) | C(12) | 174.1(3) |  | C(33) | O(11) | C(34) | C(35) | -79.2(4) |
| C(8) | O(3) | C(6) | C(5) | -132.0(3) |  | C(33) | O(11) | C(34) | C(36) | 155.9(3) |
| C(8) | O(3) | C(6) | C(7) | -12.8(4) |  | C(33) | C(37) | C(38) | O(13) | 89.8(3) |
| C(8) | O(3) | C(6) | C(13) | 105.1(3) |  | C(33) | C(37) | C(38) | C(39) | -31.7(4) |
| C(8) | O(4) | C(7) | C(6) | -39.0(4) |  | C(33) | C(37) | C(43) | C(41) | -107.8(3) |
| C(11) | C(4) | C(5) | O(2) | 142.5(3) |  | C(33) | C(37) | C(43) | C(44) | 21.0(4) |
| C(11) | C(4) | C(5) | C(6) | 23.4(4) |  | C(34) | O(11) | C(33) | C(32) | -36.2(3) |
| C(11) | C(12) | C(13) | O(5) | -92.0(3) |  | C(34) | O(11) | C(33) | C(37) | -159.0(3) |
| C(11) | C(12) | C(13) | C(6) | 32.0(4) |  | C(34) | O(12) | C(32) | C(31) | 126.9(3) |
| C(11) | C(12) | C(17) | O(6) | 8.7(3) |  | C(34) | O(12) | C(32) | C(33) | 3.4(4) |
| C(11) | C(12) | C(17) | O(7) | -111.7(3) |  | C(37) | C(43) | C(44) | O(15) | -93.9(3) |
| C(12) | C(11) | C(14) | O(6) | -32.5(3) |  | C(37) | C(43) | C(44) | C(31) | 30.5(4) |
| C(12) | C(11) | C(14) | C(15) | -153.3(3) |  | C(38) | O(13) | C(41) | O(14) | 88.4(3) |
| C(13) | O(5) | C(19) | C(20) | 166.1(3) |  | C(38) | O(13) | C(41) | C(43) | -29.7(3) |
| C(13) | C(6) | C(7) | O(4) | -82.2(3) |  | C(38) | C(37) | C(43) | C(41) | 16.5(3) |
| C(13) | C(12) | C(17) | O(6) | -120.8(3) |  | C(38) | C(37) | C(43) | C(44) | 145.3(3) |
| C(13) | C(12) | C(17) | O(7) | 118.8(3) |  | C(41) | O(13) | C(38) | C(37) | 40.7(3) |
| C(14) | O(6) | C(17) | O(7) | 87.4(3) |  | C(41) | O(13) | C(38) | C(39) | 165.3(3) |
| C(14) | O(6) | C(17) | C(12) | -30.6(3) |  | C(41) | C(43) | C(44) | O(15) | 29.1(4) |
| C(14) | C(11) | C(12) | C(13) | 142.5(3) |  | C(41) | C(43) | C(44) | C(31) | 153.5(3) |
| C(14) | C(11) | C(12) | C(17) | 14.2(3) |  | C(42) | O(14) | C(41) | O(13) | 68.1(4) |
| C(17) | O(6) | C(14) | C(11) | 40.0(3) |  | C(42) | O(14) | C(41) | C(43) | -174.0(3) |
| C(17) | O(6) | C(14) | C(15) | 165.4(3) |  | C(43) | C(37) | C(38) | O(13) | -34.5(3) |
| C(17) | C(12) | C(13) | O(5) | 31.5(4) |  | C(43) | C(37) | C(38) | C(39) | -156.0(3) |
| C(17) | C(12) | C(13) | C(6) | 155.5(3) |  | C(44) | O(15) | C(45) | C(46) | 165.0(3) |
| C(18) | O(7) | C(17) | O(6) | 69.1(4) |  | C(44) | C(31) | C(32) | O(12) | -85.4(3) |
| C(18) | O(7) | C(17) | C(12) | -173.5(3) |  | C(44) | C(31) | C(32) | C(33) | 30.8(4) |
| C(19) | O(5) | C(13) | C(6) | 130.8(3) |  | C(45) | O(15) | C(44) | C(31) | 132.1(3) |
| C(19) | O(5) | C(13) | C(12) | -104.8(3) |  | C(45) | O(15) | C(44) | C(43) | -103.4(3) |
| C(19) | C(20) | C(21) | C(22) | 178.0(4) |  | C(45) | C(46) | C(47) | C(48) | 176.3(4) |
| C(19) | C(20) | C(25) | C(24) | -177.3(3) |  | C(45) | C(46) | C(51) | C(50) | -175.8(3) |
| C(20) | C(21) | C(22) | C(23) | -0.4(6) |  | C(46) | C(47) | C(48) | C(49) | -0.1(6) |
| C(21) | C(20) | C(25) | C(24) | 0.7(5) |  | C(47) | C(46) | C(51) | C(50) | 1.3(6) |
| C(21) | C(22) | C(23) | O(8) | -179.9(4) |  | C(47) | C(48) | C(49) | O(16) | 179.9(4) |
| C(21) | C(22) | C(23) | C(24) | 0.2(6) |  | C(47) | C(48) | C(49) | C(50) | 0.7(6) |
| C(22) | C(23) | C(24) | C(25) | 0.5(6) |  | C(48) | C(49) | C(50) | C(51) | -0.3(6) |
| C(23) | C(24) | C(25) | C(20) | -0.9(6) |  | C(49) | C(50) | C(51) | C(46) | -0.7(6) |
| C(25) | C(20) | C(21) | C(22) | 0.0(6) |  | C(51) | C(46) | C(47) | C(48) | -0.8(6) |
| C(26) | O(8) | C(23) | C(22) | 176.8(4) |  | C(52) | O(16) | C(49) | C(48) | 175.4(4) |
| C(26) | O(8) | C(23) | C(24) | -3.2(6) |  | C(52) | O(16) | C(49) | C(50) | -5.4(6) |

Table 7 Hydrogen Atom Coordinates (Å×104) and Isotropic Displacement Parameters (Å2×103) for cu\_20220627\_Zh\_Wj\_06\_24\_OMe\_0m.

| Atom | *x* | *y* | *z* | U(eq) |
| --- | --- | --- | --- | --- |
| H(2A) | 2441.68 | 9008.35 | -5013.21 | 66 |
| H(2B) | 1075.78 | 8248.7 | -4897.62 | 66 |
| H(2C) | 3105.42 | 7378.2 | -5125.2 | 66 |
| H(3A) | 20.86 | 8996.78 | -3454.74 | 62 |
| H(3B) | 1257.43 | 9869 | -3540.4 | 62 |
| H(3C) | 1403.43 | 8735.56 | -2796.96 | 62 |
| H(4) | 5621 | 6429.42 | -4571.51 | 37 |
| H(5) | 4440.37 | 4871.03 | -4085.71 | 40 |
| H(7A) | 2288.86 | 5155.56 | -2052.64 | 46 |
| H(7B) | 2428.44 | 4284.83 | -2842.34 | 46 |
| H(9A) | 4207.9 | 2177.86 | -3405.5 | 92 |
| H(9B) | 4696.26 | 894.5 | -2745.11 | 92 |
| H(9C) | 6176.21 | 1090.56 | -3408.95 | 92 |
| H(10A) | 7980 | 1148.09 | -2259.53 | 97 |
| H(10B) | 6536.35 | 904.77 | -1591.61 | 97 |
| H(10C) | 7054.49 | 2222.46 | -1550.28 | 97 |
| H(12) | 7895.7 | 4944.87 | -2475.68 | 36 |
| H(13) | 5537.73 | 4943.5 | -1717.93 | 36 |
| H(14) | 9190.32 | 6670.7 | -3651.37 | 46 |
| H(16) | 7199.79 | 9405.16 | -5490.45 | 64 |
| H(17) | 6187.22 | 7475.89 | -1850.89 | 39 |
| H(18A) | 9158.51 | 8222.93 | -1891.64 | 68 |
| H(18B) | 10159.44 | 7044.33 | -1235.54 | 68 |
| H(18C) | 8102.95 | 7882.36 | -1070.96 | 68 |
| H(19A) | 4717.56 | 6738.44 | -887.33 | 42 |
| H(19B) | 3025.93 | 6337.32 | -838.78 | 42 |
| H(21) | 1087.05 | 8024.44 | 99.59 | 47 |
| H(22) | -637.77 | 10293.85 | 380.54 | 52 |
| H(24) | 1714.1 | 11625.43 | -1649.51 | 47 |
| H(25) | 3403.47 | 9326.48 | -1935.95 | 43 |
| H(26A) | -591.6 | 13500.19 | -1464.73 | 107 |
| H(26B) | 691.38 | 13605.43 | -841.4 | 107 |
| H(26C) | -1359.69 | 14481.66 | -700.7 | 107 |
| H(28A) | -130.89 | 7939 | 3402.93 | 84 |
| H(28B) | -506.33 | 9234.4 | 2752.43 | 84 |
| H(28C) | 884.11 | 8968.29 | 3406.92 | 84 |
| H(29A) | 3208.19 | 8918.22 | 2215.41 | 85 |
| H(29B) | 1879.07 | 9116.28 | 1540.79 | 85 |
| H(29C) | 3670 | 7797.97 | 1531.1 | 85 |
| H(30A) | 1428.86 | 4901.24 | 2048.99 | 46 |
| H(30B) | 365.08 | 5761.77 | 2842.79 | 46 |
| H(32) | 2301.72 | 5204.36 | 4072.1 | 40 |
| H(33) | 4753.59 | 3740.65 | 4576.41 | 37 |
| H(35A) | 3797.76 | 1101.59 | 5050.01 | 68 |
| H(35B) | 1787.08 | 1969.61 | 4925.29 | 68 |
| H(35C) | 3006.91 | 2741.7 | 5139.69 | 68 |
| H(36A) | 3788.72 | 1230.47 | 2841.29 | 64 |
| H(36B) | 2252.85 | 1049.41 | 3472.91 | 64 |
| H(36C) | 4246.23 | 168.37 | 3625.59 | 64 |
| H(38) | 8954.41 | 3613.52 | 3638.58 | 44 |
| H(40) | 8581.77 | 1189.11 | 5636.73 | 69 |
| H(41) | 7653.7 | 2548.05 | 1912.83 | 40 |
| H(42A) | 11393.08 | 3008.94 | 1167.28 | 74 |
| H(42B) | 10330.09 | 2009.99 | 1184.22 | 74 |
| H(42C) | 11396.58 | 1962.79 | 1945.7 | 74 |
| H(43) | 6625.93 | 5157.38 | 2444.9 | 36 |
| H(44) | 4616.28 | 5126.51 | 1706.76 | 37 |
| H(45A) | 5974.39 | 3322.43 | 900.3 | 44 |
| H(45B) | 3942.53 | 3667.29 | 833.55 | 44 |
| H(47) | 4112.16 | 1961.54 | -85.75 | 49 |
| H(48) | 4861.08 | -337.45 | -386.99 | 54 |
| H(50) | 7522.4 | -1587.57 | 1641.75 | 47 |
| H(51) | 6735.68 | 720.21 | 1946.86 | 42 |
| H(52A) | 7879.93 | -4477.82 | 650.2 | 112 |
| H(52B) | 7328.65 | -3513.31 | 1423 | 112 |
| H(52C) | 8941.37 | -3525.21 | 776.66 | 112 |

Experimental

Single crystals of C26H33N3O8
[cu\_20220627\_Zh\_Wj\_06\_24\_OMe\_0m]
were
[].
A suitable crystal was selected and
[]
on a
Bruker D8 VENTURE
diffractometer. The crystal was kept at 198.00 K during data collection.
Using Olex2 [1], the structure was solved with the
SHELXT
[2] structure solution program using
Intrinsic Phasing
and refined with the
SHELXL
[3] refinement package using
Least Squares
minimisation.

1. Dolomanov, O.V., Bourhis, L.J., Gildea, R.J, Howard, J.A.K. & Puschmann, H.
   (2009), J. Appl. Cryst. 42, 339-341.
2. Sheldrick, G.M. (2015). Acta Cryst. A71, 3-8.
3. Sheldrick, G.M. (2015). Acta Cryst. C71, 3-8.

Crystal structure determination of
[cu\_20220627\_Zh\_Wj\_06\_24\_OMe\_0m]

**Crystal Data**
for C26H33N3O8 (*M*=515.55 g/mol):
triclinic, space group P1 (no. 1),
*a* = 8.3171(2) Å, *b* = 10.4531(3) Å, *c* = 16.3414(5) Å, *α* = 84.815(2)°, *β* = 83.036(2)°, *γ* = 67.7400(10)°,
*V*= 1303.61(6) Å3,
*Z* = 2,
*T* = 198.00 K,
μ(CuKα) = 0.815 mm-1,
*Dcalc* = 1.313 g/cm3,
28420 reflections measured (5.454° ≤ 2Θ ≤ 136.766°),
9268 unique (*R*int = 0.0498, Rsigma = 0.0490) which were used in all calculations.
The final *R*1 was 0.0469
(I > 2σ(I)) and *wR*2 was 0.1236 (all data).

Refinement model description

Number of restraints - 3,
number of constraints - unknown.

Details:

```
1. Fixed Uiso
```

This report has been created with Olex2, compiled on
2022.04.07 svn.rca3783a0 for OlexSys. Please
let us know
if there are any errors or if you would like to have additional features.
