# Supplementary material for: Total syntheses of Tetrodotoxin and 9-epiTetrodotoxin
Source: Nat Commun. 2024 Jan 23;15:679. doi: 10.1038/s41467-024-45037-0 (PMC10806222; doi:10.1038/s41467-024-45037-0)
Supplement: Supplementary file 4 — Source Data [file 41467_2024_45037_MOESM4_ESM.zip › Surce Data 20231213/Surce Data New/HPLC Source Data/TTX-Commercial-HPLC.pdf]

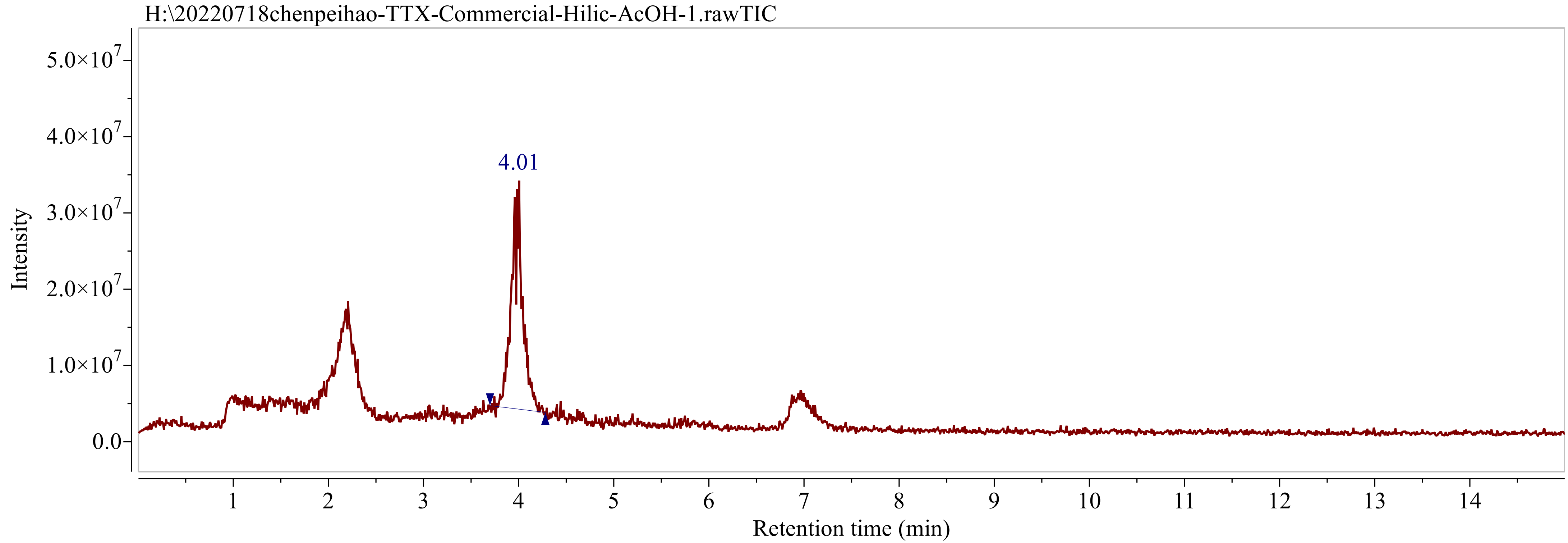
H:\20220718chenpeihao-TTX-Commercial-Hilic-AcOH-1.rawTIC
Intensity
5.0×10^7
4.0×10^7
3.0×10^7
2.0×10^7
1.0×10^7
0.0
4.01
1
2
3
4
5
6
7
8
9
10
11
12
13
14
Retention time (min)

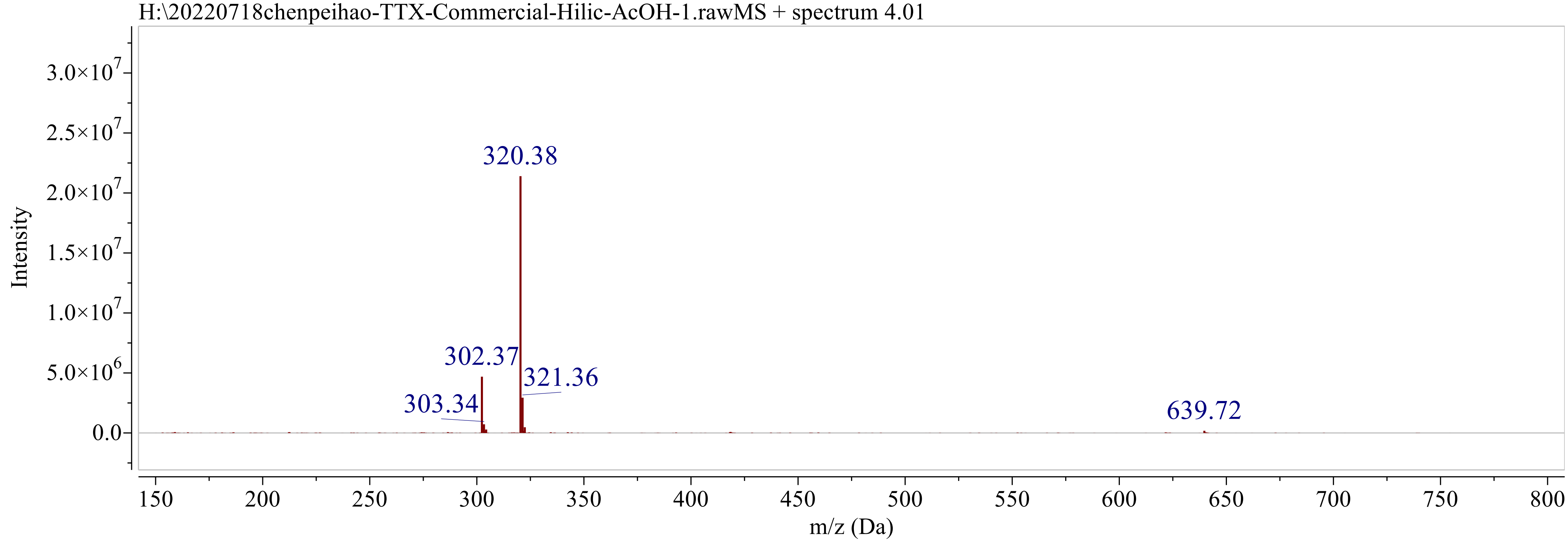
H:\20220718chenpeihao-TTX-Commercial-Hilic-AcOH-1.rawMS + spectrum 4.01
Intensity
3.0×10^7
2.5×10^7
2.0×10^7
1.5×10^7
1.0×10^7
5.0×10^6
0.0
320.38
302.37
321.36
303.34
639.72
150
200
250
300
350
400
450
500
550
600
650
700
750
800
m/z (Da)
