# Supplementary figures and images for: Total syntheses of Tetrodotoxin and 9-epiTetrodotoxin
Source: Nat Commun. 2024 Jan 23;15:679. doi: 10.1038/s41467-024-45037-0 (PMC10806222; doi:10.1038/s41467-024-45037-0)

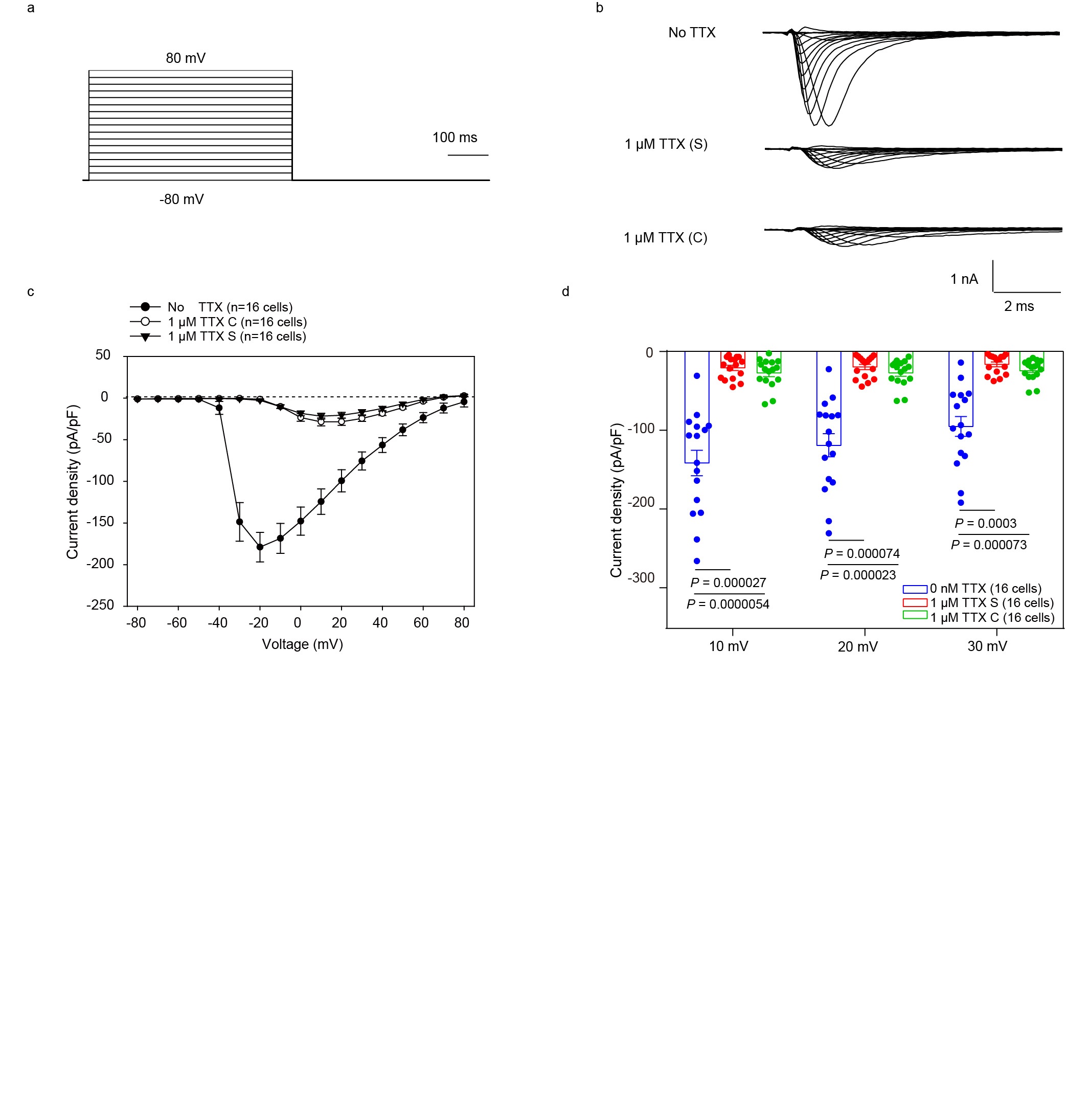

Supplement: Supplementary file 4 — Source Data [file 41467_2024_45037_MOESM4_ESM.zip › Surce Data 20231213/Surce Data New/TTX_normalized sodium currents Source Data/Supplementary Figure 4-ZSF-20231219.jpg]

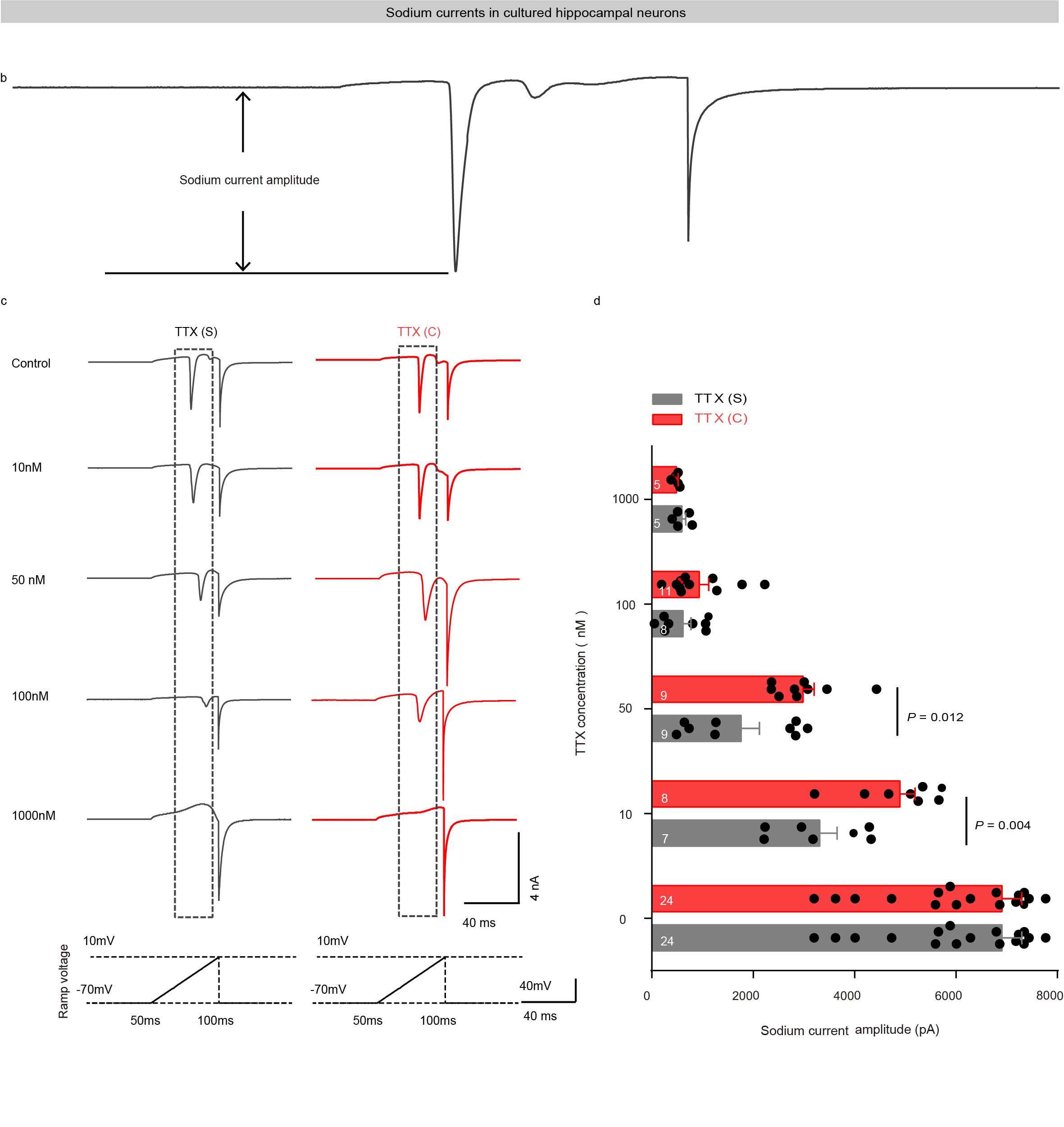

Supplement: Supplementary file 4 — Source Data [file 41467_2024_45037_MOESM4_ESM.zip › Surce Data 20231213/Surce Data New/TTX_normalized sodium currents Source Data/Supplementary Figure 5-ZSF-20231219.jpg]

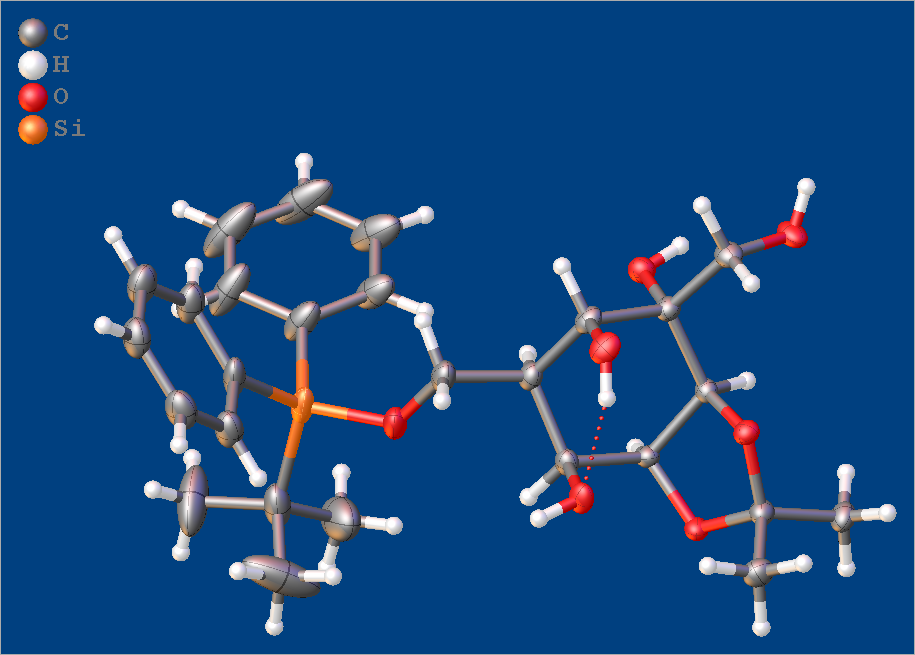

Supplement: Supplementary file 4 — Source Data [file 41467_2024_45037_MOESM4_ESM.zip › Surce Data 20231213/Surce Data New/Crystal Structure Source Data/CCDC-2184298/screenshot.png]

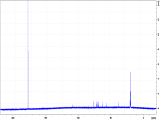

Supplement: Supplementary file 4 — Source Data [file 41467_2024_45037_MOESM4_ESM.zip › Surce Data 20231213/Surce Data New/NMR Source Data/Compound1(method A)-CNMR/pdata/1/thumb.png]

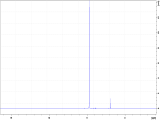

Supplement: Supplementary file 4 — Source Data [file 41467_2024_45037_MOESM4_ESM.zip › Surce Data 20231213/Surce Data New/NMR Source Data/Compound1(method A)-HNMR/pdata/1/thumb.png]
